# Spectrophotometric- and LC/MS-Based Lipidomics Analyses Revealed Changes in Lipid Profiles of Pike Eel (*Muraenesox cinereus*) Treated with Stable Chlorine Dioxides and Vacuum-Packed during Chilled Storage

**DOI:** 10.3390/foods12142791

**Published:** 2023-07-22

**Authors:** Shanshan Shui, Yingru Wu, Xiaonan Chen, Ruixue Li, Huicheng Yang, Baiyi Lu, Bin Zhang

**Affiliations:** 1 Department of Food Science and Nutrition, College of Biosystems Engineering and Food Science, Zhejiang University, Hangzhou 310058, China; shuiss@zjou.edu.cn (S.S.); bylu@zju.edu.cn (B.L.); 2 Key Laboratory of Health Risk Factors for Seafood of Zhejiang Province, College of Food Science and Pharmacy, Zhejiang Ocean University, Zhoushan 316022, China; yrwu9708@163.com (Y.W.); 2012111199@mail.hfut.edu.cn (X.C.); 3 Zhejiang Marine Development Research Institute, Zhoushan 316022, China; 4 Comprehensive Technical Service Center of Zhoushan Customs, Zhoushan 316000, China; lruixue2023@163.com

**Keywords:** pike eel muscle, lipid profiles, chlorine dioxides, vacuum-packaged, lipid oxidation

## Abstract

Spectrophotometric- and liquid chromatography/mass spectrometry (LC/MS)-based lipidomics analyses were performed to explore the changes of lipid profiles in pike eel (*Muraenesox cinereus*) under stable chlorine dioxides (ClO_2_) and vacuum-packed treatment during chilled storage. The peroxide value (PV) and malondialdehyde (MDA) content in ClO_2_ treated and vacuum-packaged (VP) samples were significantly reduced compared to simple-packaged (SP) samples during whole chilled storage. The LC/MS-based lipidomics analyses identified 2182 lipid species in the pike eel muscle classified into 39 subclasses, including 712 triglycerides (TGs), 310 phosphatidylcholines (PCs), 153 phosphatidylethanolamines (PEs), and 147 diglycerides (DGs), among others. Further, in comparison with fresh pike eel (FE) muscle, 354 and 164 higher and 420 and 193 lower abundant levels of differentially abundant lipids (DALs) were identified in SP samples and VP samples, respectively. Compared with the VP batch, 396 higher and 404 lower abundant levels of DALs were identified in the SP batch. Among these, PCs, PEs, TGs, and DGs were more easily oxidized/hydrolyzed, which could be used as biomarkers to distinguish FE, SP, and VP samples. This research provides a reference for controlling lipid oxidation in fatty fish.

## 1. Introduction

Pike eel (*Muraenesox cinereus*), as one of the main demersal marine resources, is widely spread in Southeast Asia, the Indian Ocean, Australia, and the Pacific Northwest. Pike eel is regarded as a functional food-fish variety with high polyunsaturated fatty acids (PUFAs), proteins, essential amino acids, and minerals [1]. Due to microbial growth and reproduction and the effects of endogenous proteases during processing, transportation, storage, and marketing, pike eel is prone to quality degradation [2]. Cryopreservation is often used to inhibit biochemical reactions in pike eel muscles and prolong the shelf life of pike eel. However, pike eel still suffers from soft muscle texture, color, flavor, and freshness loss during chilled storage, mainly resulting from denaturation and oxidation of muscle proteins and lipids [2]. Previous studies on the alterations of protein profiles in pike eel muscle during cold-temperature storage have been conducted [3]. The lipid oxidation and the alterations of pike eel muscle under cold stress should be investigated in depth.

Vacuum packaging, a nonthermal processing technique, can inhibit the growth of aerobic bacteria and reduce oxidation by decreasing the oxygen concentration in the package by 97–99%, which has been widely used in aquatic product preservation [4]. Chan et al. found that vacuum-packaged Atlantic salmon fillets presented better quality traits with a lower drip loss and a higher redness and yellowness than other fillets during refrigerated storage at 4 °C [5]. Tsoukalas et al. reported that European plaice fillets packaged in vacuum and stored at 4 °C lengthened the shelf life by shortening the lag time of psychrotrophic aerobic plate count and aerobic bacteria [6]. However, the survival and growth of microaerophilic and anaerobic psychrotrophic pathogens in vacuum-packaged aquatic products are worrisome. In order to improve the safety and quality characteristics of aquatic products, various intervention techniques have recently been adopted, e.g., the addition of chlorine dioxide. Stable chlorine dioxide (ClO_2_) is an effective disinfectant due to its strong oxidation capacity and biocidal activity [7]. According to the GB 2760-2014 of China National Standard, the maximum dose of ClO_2_ that can be used for aquatic products of fish processing is 0.05 g/kg. The combined treatment of erythorbate, gellan gum glazing, and ClO_2_ on peeled Pacific white shrimp could slow *Escherichia coli* and *Staphylococcus aureus* growth and quality deterioration of shrimp products [8]. ClO_2_ combined with slightly acidic electrolyzed water on inactivated *Shewanella putrefaciens* in large yellow croaker destroyed bacterial morphology [9]. Further, the effects of ClO_2_ and vacuum packaging on the flavor, texture properties, and protein stability of pike eel muscle during chilled storage have been studied [2], whereas their effects on lipid changes are not clear.

Lipidomics is a powerful emerging strategy that can describe comprehensive lipid profiles, their interactions, and metabolic pathways; it can also identify lipid biomarkers by distinguishing different lipid species and illuminating their functional roles in the organisms [10]. Liquid chromatography/mass spectrometry (LC/MS)-based lipidomics has been conducted to investigate the alternations of lipids in *Litopenaeus vannamei* muscle induced by hydroxyl radicals, which suggested CL(62:2), PC(38:3), and PE(34:9) as potential biomarkers for differentiating fresh and oxidized shrimp [11]. Yan et al. found that phosphatidylcholines (PCs), phosphatidylethanolamines (PEs), triglycerides (TGs), and diglycerides (DGs) were more easily oxidized/hydrolyzed in hairtail (*Trichiurus haumela*) during 6 d of chilled storage conducted by LC/MS-based lipidomics [12]. Fang et al. investigated that lipid side-chain modifications, backbone cleavage, and decomposition led to changes in lipid distribution in frozen hairtail muscle samples through LC/MS-based lipidomics [13]. Liao et al. reported that TG(18:4/18:3/18:4), TG(18:4/14:1/18:4), and PC(18:2e/10:3) were the top three oxidated lipids observed in hairtail samples after air-drying via LC/MS-based lipidomics [14].

In this current research, we explored the effects of ClO_2_ and vacuum-packaged treatment on the degree of lipid oxidation in pike eel muscle. Lipid compositions and biomarkers induced by cold stress and different treatments in pike eel muscle tissues were explored. The results enrich the data of lipid change and provide a reference for quality control of pike eel.

## 2. Materials and Methods

### 2.1. Chemical and Reagents

ClO_2_ (≥98%, solid) was obtained from Shandong Huashi Pharmaceutical Co., Ltd. (Weifang, China). Glacial acetic acid, chloroform, potassium iodide, sodium thiosulfate, petroleum ether, anhydrous sodium sulfate, and trichloroacetic acid (TCA) were provided by Sinopharm Chemical Reagent Co., Ltd. (Suzhou, China). Potassium dichromate, isooctane, disodium ethylenediamine tetraacetate, and thiobarbituric acid (TBA) were obtained from Sangon Biotech Co., Ltd. (Shanghai, China). Formic acid was purchased from Anpu Scientific Instrument Co., Ltd. (Shanghai, China). Methanol and acetonitrile were purchased from Thermo (Waltham, MA, USA). Methyl *tert*-butyl ether (MTBE), 2-propanol, and ammonium acetate were supplied by Sigma-Aldrich Co., LLC (St.Louis, MO, USA).

### 2.2. Pike Eel Samples and Treatments

Fresh pike eels (FE, average body weight of 1.4–1.6 kg, length of 65–70 cm, and K-value of 2.0–2.5%) were obtained from the aquatic market (Zhoushan, China). Pike eel samples were placed in foam boxes filled with ice and delivered to the lab within 30 min. Upon arrival, pike eel samples were rinsed with ice water, and the head and viscera were removed. After thoroughly cleaning with distilled water, the pike eels were cut into 6 cm × 6 cm × 2 cm fillets. The obtained pike eel fillets were randomly divided into two groups. SP (simple-packaged) group: soaking the fillets in distilled water for 10 min, wiping off the moisture, and packaging directly in polyethylene bags. VP (vacuum-packaged) group: soaking the fillets in 0.05% ClO_2_ solution for 10 min, wiping off the moisture, and vacuum packaging (−0.02 MPa) in polyethylene bags. Finally, the packed fillets were stored at 4 °C for 10 d. Peroxide value (PV) and malondialdehyde (MDA) analyses of triplicate samples from two groups were performed at 2 d intervals during chilled storage. LC/MS-based lipidomics analyses of six parallel samples from FE, SP, and VP groups were conducted on day 8 of chilled storage.

### 2.3. Peroxide Value (PV) and Malondialdehyde (MDA) Analyses

Lipids were extracted from pike eel muscle tissues according to the experimental method reported by Zhang et al. [15]. The PV of pike eel muscle was determined according to the GB 5009.227-2016 [16]. of the Chinese national standard [17]. The prepared lipids (2 g) were dissolved in 30 mL trichloromethane and glacial acetic acid solution, which was thoroughly mixed and reacted with 1 mL potassium iodide solution for 3 min. Next, the mixture was added with 100 mL distilled water, and the precipitated iodine was titrated with 0.01 mol/L sodium thiosulfate solution. The value of PV was expressed as the mass fraction of peroxide equivalent to iodine.

The MDA content of pike eel muscle was determined according to the GB 5009.181-2016 of the Chinese national standard [18]. The prepared lipids (2 g) were added with 20 mL 7.5% (*w*/*v*) TCA and then reacted at 50 °C for 30 min. After cooling to room temperature, 5 mL 0.02 mol/L TBA was added to the mixture and reacted at 90 °C for 30 min. MDA was extracted by TCA solution and acted with TBA to produce the colored compound. The absorbance value of MDA at 532 nm was determined. The absolute difference between two independent measurements obtained under repeatability conditions must not exceed 10% of the arithmetic mean. The limit of detection was 0.05 mg/kg. The limit of quantification was 0.10 mg/kg.

### 2.4. Liquid Chromatography/Mass Spectrometry (LC/MS)-Based Lipidomics Analysis

#### 2.4.1. Lipid Extraction

A total of 50 mg of pike eel muscle tissue was mixed with 280 μL pre-cooled methanol/water (2:5, *v*/*v*) solution and 400 µL MTBE. Next, the sample was ground in a frozen tissue grinder for 6 min (−10 °C, 50 Hz). Then, the mixture was extracted with ultrasonic for 30 min (5 °C, 40 kHz) and stored for 30 min (−20 °C). After centrifugation at 13,000× *g* for 15 min (4 °C), the supernatant was collected and dried with nitrogen. Afterward, 100 µL isopropyl alcohol/acetonitrile solution (1:1, *v*/*v*) was added to the supernatant for resolution. The solution was swirled for 30 s, ultrasonically extracted for 5 min (5 °C, 40 kHz), and centrifuged for 10 min (4 °C) at 13,000× *g*. The supernatant was collected as lipids for the following LC/MS detection.

#### 2.4.2. Liquid Chromatography/Mass Spectrometry (LC/MS) Analysis

LC/MS analysis of lipid extracts was performed on an ultra-high performance liquid chromatography-tandem Fourier transform mass spectrometry (UHPLC-Q Exactive HF-X; Thermo, Waltham, MA, USA) system equipped with an Accucor C30 column (100 mm × 2.1 mm, 2.6 μm; Thermo, Bremen, Germany). The injection volume, column temperature, and flow rate were set as 2 μL, 40 °C, and 0.4 mL/min, respectively. The mobile phase consisted of solvent I [acetonitrile/water, 1:1 (*v*/*v*); containing 0.1% formic acid and 10 mmol/L ammonium acetate] and solvent II [acetonitrile/isopropyl alcohol/water, 10:88:2 (*v*/*v*); containing 0.02% formic acid and 2 mmol/L ammonium acetate]. Elution separation was performed according to the following gradient: 0–4 min, I/II (65:35, *v*/*v*); 4–12 min, I/II (40:60, *v*/*v*); 12–15 min, I/II (15:85, *v*/*v*); 15–18 min, I/II (0:100, *v*/*v*); and 18–20 min, I/II (65:35, *v*/*v*).

Afterward, the samples were ionized by electrospray, and the MS signals were collected by positive and negative ion scanning modes. Parameters are as follows: scan type, 200–2000 *m*/*z*; sheath gas flow rate, 60 psi; aux gas flow rate, 20 psi; aux gas heater temperature, 370 °C; ion spray voltage floating in the positive mode, 3 kV; ion spray voltage floating in the negative mode, −3 kV; and normalized collision energy, 20–40–60 V.

### 2.5. Data Analysis

PV and MDA were determined in triplicate (n = 3). The statistical analysis was performed using SPSS 20.0 (SPSS Inc., Chicago, IL, USA) and Origin 2016 (OriginLab Inc., Northampton, MA, USA). The raw data followed by UPLC-MS/MS analyses were channeled into the LipidSearch 4.2.21 (Thermo, CA, USA) for peak detection, comparison, and identification. The lipids were identified by MS/MS fragments. Mass tolerance for both precursor and product tolls was set to 5.0 ppm. The m-score threshold was set to 2.0. The production of intensity threshold was set to 1.0%. The preprocessing results generated MS/MS data matrices that consisted of characteristic peak search database identification, retention time, mass-to-charge ratio values, and peak intensity. Partial least squares discriminant analysis (PLS-DA) was implemented by SIMCA 14.1 (MKS Data Analytics Solutions, Umea, Sweden) to distinguish FE, SP, and VP samples. The selected lipids with variable importance in projection (VIP) scores of ≥1 in the PLS-DA model and *p* values ≤ 0.05 in the statistical difference analysis were identified as differentially abundant lipids (DALs).

## 3. Results and Discussion

### 3.1. Peroxide Value (PV) and Malondialdehyde (MDA) Content Analyses

**Figure 1:**
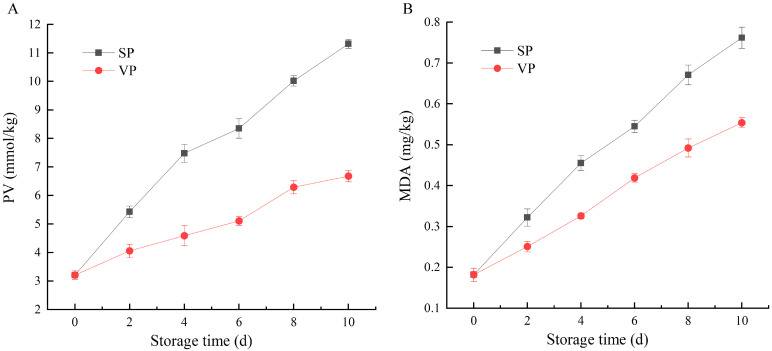
Changes in (**A**) peroxide value (PV) and (**B**) malondialdehyde (MDA) content of simple-packaged (SP) and vacuum-packaged (VP) pike eels during chilled storage (n = 3).

PV and MDA are important indicators to characterize the lipid oxidation degree of fish, which can be used to judge fish rancidity [19]. The changes in PV and MDA content in pike eel muscles during 10 days of chilled storage are shown in Figure 1. The initial PV and MDA levels were 3.21 mmol/kg fat and 0.18 mg/kg muscle, respectively, indicating that the pike eel was fresh with good quality. The PV and MDA content in all the pike eel samples showed the same trend, which increased gradually with the extension of chilled storage. Lipid oxidation is highly likely to occur in oily fish species during processing, transportation, marketing, and/or storage, resulting in rapid increases in PV and MDA content of fish muscle [20]. Lipids, such as glycerides and phospholipids in fish muscle, are firstly hydrolyzed by lipases to free fatty acids, which are oxidated to produce peroxides. As unstable compounds, peroxides decompose into short-chain hydrocarbons, such as aldehydes, ketones, and alcohols. These oxidative products can adversely affect the texture, color, and flavor of fish, which can degrade the quality of the fish [21,22]. Compared with SP samples, PV and MDA content of VP samples were significantly lower during the entire storage period, pointing out that the treatment of ClO_2_ combined with vacuum packaging notably inhibited lipid oxidation in pike eel. This result was likely due to the synergistic effect of the limited oxygen molecule of vacuum packaging and the oxidative properties of ClO_2_ [23,24].

Complex biochemical processes are involved in the lipid oxidation of pike eel muscles under ClO_2_ and vacuum packaging treatment during chilled storage, and the conventional PV and MDA investigations provide limited information on lipid oxidation. Thus, the relationships between the oxidation and lipid profiles in pike eel muscle tissues need to be explored in depth.

### 3.2. Lipid Composition Analysis

**Figure 2:**
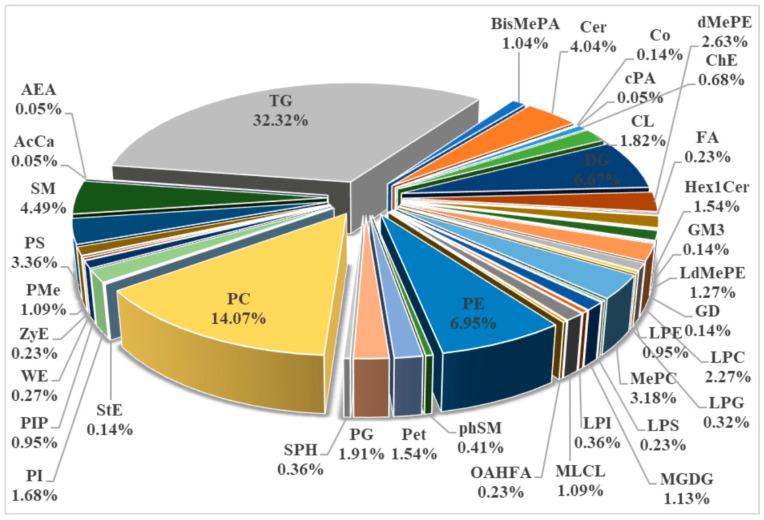
Lipid classification and composition in fresh (FE), simple-packaged (SP), and vacuum-packaged (VP) pike eels via LC/MS-based lipidomics analysis.

Lipid classification and composition in FE, SP, and VP samples were recognized and categorized using LC/MS-based lipidomics analysis (Figure 2 and Supplementary Table S1). A total of 2182 lipid species were identified in the pike eel muscle, which could be classified into five categories, including 21 fatty acyls (FAs, 0.96%), 884 glycerolipids (GLs, 40.51%), 1010 glycerophospholipids (GPs, 50.05%), 244 sphingolipids (SPs, 11.18%), and 23 sterol lipids (STs, 1.05%). These lipid categories could be further divided into 39 subclasses, including 712 TGs, 310 PCs, 153 PEs, 147 DGs, 99 sphingomyelins (SMs), 87 ceramides (Cers), 74 phosphatidylserines (PSs), 70 methyl-PCs (MePCs), 58 dimethyl-PEs (dMePEs), 50 lysophosphatidylcholines (LPCs), and other lipids.

TGs and DGs, as neutral lipids, belong to the GL category. TGs play important roles in energy supplements and fatty acid transportation for organisms. As reported, TGs and DGs (intermediate products of TGs), accounting for more than 80%, were important abundant lipid classes in the muscle, head, and viscera of *Oreochromis niloticus* [25]. Cheng et al. found that TGs were the most abundant lipids in extracted oil from *Trachinotus ovatus oil*. The oil also contained a small number of DGs, PCs, and PEs [26].

PCs, PEs, PSs, LPCs, MePCs, and dMePEs, as phospholipids and their derivatized lipids, belong to the GP category. Phospholipids, a class of polar lipids, are the main constituents of biological membranes and participate in several biological processes [27]. PEs and PCs were dominant phospholipids in *Scomberomorus niphonius*, *Scophthalmus maximus*, *Oncorhynchus keta,* and *Ctenopharyngodon idellus*. LPCs, lysophosphatidylethanolamines (LPEs), PSs, phosphatidylglycerols (PGs), and phosphatidylinositols (PIs) were also detected, although their abundances were much lower [28].

SP is closely involved in regulating the biological processes of cells from growth to death and senescence [29]. SMs and Cers belong to the SP category, while their abundances in pike eel muscles were much lower than GL and phospholipid category, accounting for less than 10%. Shi et al. reported that SMs and Cers showed relatively low abundance compared to TGs, LPCs, PCs, PEs, and DGs in raw, thermal-processed tilapia fillets [30]. The above reports were in agreement with our conclusions obtained in this present work.

### 3.3. Partial Least Squares Discriminant Analysis (PLS-DA)

**Figure 3:**
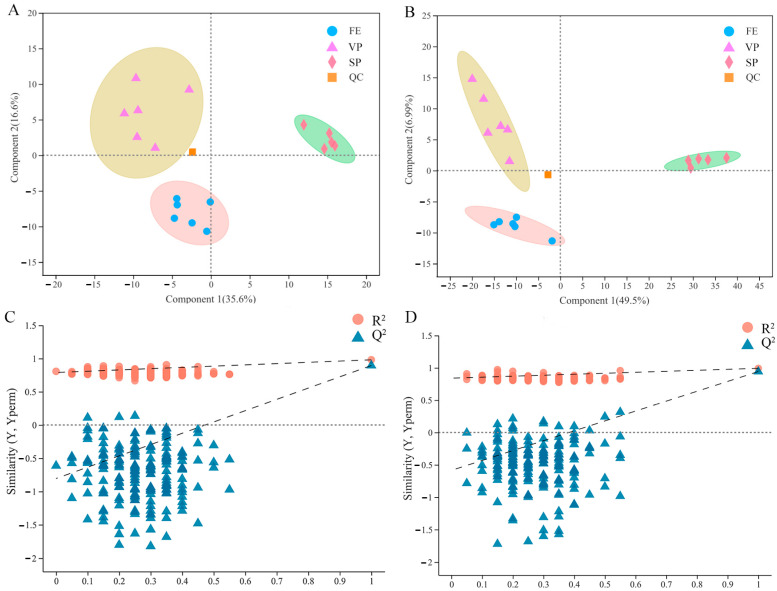
Partial least squares discriminant analysis (PLS-DA) and permutation score plots of PLS-DA obtained from fresh (FE), simple-packaged (SP), and vacuum-packaged (VP) pike eels via LC/MS-based lipidomics analysis. (**A**,**C**) negative ion mode. (**B**,**D**) positive ion mode.

In this work, the PLS-DA model was performed to compare the overall difference of the lipid composition in FE, SP, and VP pike eel samples. As shown in Figure 3A,B, six replicated quality control (QC) samples were closely clustered together and effectively distinguished from FE, SP, and VP groups, verifying the accuracy and stability of instrument platform detection. The FE, SP, and VP samples were clearly represented in the scatter plots as three well-separated clusters. The parameters of R^2^X, R^2^Y, and Q^2^ were calculated as 0.758, 0.981, and 0.895 in negative ion mode and 0.775, 0.992, and 0.942 in positive ion mode, respectively. The relatively high levels of the R^2^ factor and Q^2^ coefficient indicated that the PLS-DA models constructed in this current study were stable and reliable with good predictive ability [31]. In addition, the robustness of PLS-DA models was further evaluated by the permutation score plots (Figure 3C,D). The intercepts of R^2^ (0.0, 0.7905) and Q^2^ = (0.0, −0.8047) in negative ion mode, and R^2^ = (0.0, 0.84) and Q^2^ = (0.0, −0.5852) in positive ion mode confirmed the models were statistically valid without overfitting. In conclusion, the apparent separations among FE, SP, and VP samples in PLS-DA models further demonstrated significant alterations in the lipid profiles of pike eel muscles under cold stress and different treatment.

### 3.4. Differentially Abundant Lipids (DALs) Analysis

**Figure 4:**
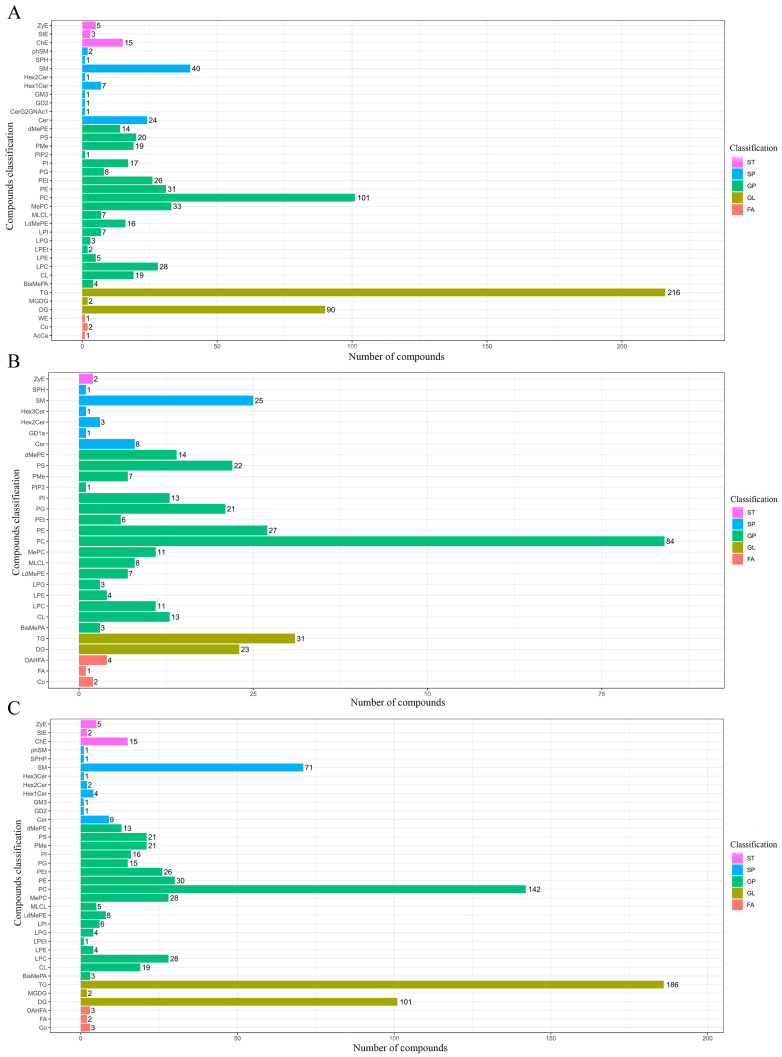
Composition and number of the differentially abundant lipids (DALs; *p* ≤ 0.05 and VIP ≥ 1 of lipid species) in pike eel samples. (**A**) simple-packaged (SP) vs. fresh (FE) pike eels. (**B**) vacuum-packaged (VP) vs. fresh (FE) pike eels. (**C**) simple-packaged (SP) vs. vacuum-packaged (VP) pike eels.

The *p* values (≤0.05) obtained by statistical analysis and the VIP values (≥1) resulting from PLS-DA models were performed to identify the differentially abundant lipids (DALs) detected in pike eel samples. The composition and number of DALs between SP and FE samples are shown in Figure 4A. The full names of the compound classification are shown in Table S2. The detected 774 DALs of five categories were assigned to 37 subclasses, including 216 TGs, 101 PCs, 90 DGs, 40 SMs, 33 MePCs, 31 PEs, 28 LPCs, 26 phosphatidylethanol (PEts), 24 Cers, and 20 PSs, among others. At the same time, 357 DALs between VP and FE samples (Figure 4B) were detected and classified into 29 subclasses, including 84 PCs, 31 TGs, 27 PEs, 25 SMs, 23 DGs, 22 PSs, and 21 PGs, among others. As reported in Figure 4C, 800 lipid species in SP and VP samples were deemed to be DALs and categorized into 36 subclasses, including 186 TGs, 142 PCs, 101 DGs, 71 SMs, 30 PEs, 28 MePCs, 28 LPCs, 26 PEts, 21 PSs, and 21 phosphatidylmethanol (PMes), among others.

**Figure 5:**
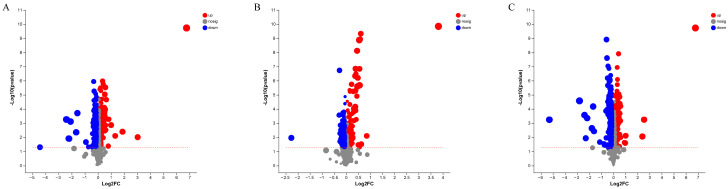
Volcano plot of lipid profiles identified in pike eel samples. (**A**) simple-packaged (SP) vs. fresh (FE) pike eels. (**B**) vacuum-packaged (VP) vs. fresh (FE) pike eels. (**C**) simple-packaged (SP) vs. vacuum-packaged (VP) pike eels.

Additionally, the differences in the abundance levels of lipid species between pike eel samples, as well as the statistical significance, were visualized using volcano plots (Figure 5). Each dot in the volcano plot represented a lipid compound. Compared with FE samples, 354 DALs marked with red dots, e.g., MePC(16:0e/20:4), LdMePE(20:0e), TG(16:0/11:3/20:2), TG(14:0/10:2/14:0), ZyE(20:5), LdMePE(18:0e), PC(14:0e/17:1), and LPC(20:0e), were significant with higher abundance levels in SP samples (Figure 5A and Table S3). While 420 DALs marked blue dots, e.g., DG(18:3/22:6), dMePE(22:6/22:6), MePC(18:4/18:3), LPE(20:5), PE(20:1/22:6), PE(14:0/22:6), PE(20:4/20:4), and TG(8:0/11:2/22:6), were significant with lower abundance levels in SP samples (Figure 5A and Table S4). The difference in lipid compositions between VP and FE samples are shown in Figure 5B and Tables S5 and S6. DALs of 164 were detected at higher abundances in the VP batch, including PG(15:0/16:1), PS(16:1/18:3), PMe(16:1/18:1), PC(11:0/18:2), MePC(8:0/22:5), PE(17:1/16:1), dMePE(16:1/14:1), and PE(16:1/14:0). DALs of 193 were explored at lower levels in the VP group, which included CL(20:5/20:4/22:4/24:1), CL(24:0/16:0/16:0/20:0), dMePE(16:0/22:6), dMePE(16:0/20:5), PS(22:6/23:0), PC(16:0/18:2), PC(24:1/22:6), and PC(14:0/20:4). Differential lipid changes between SP and VP samples are presented in Figure 5C and Tables S7 and S8. MePC(16:0e/20:4), TG(16:0/11:3/20:2), LdMePE(20:0e), TG(11:0/18:1/20:5), SM(d18:1/23:5), PC(16:0/13:0), dMePE(14:0/14:0), PS(18:0/18:1), and other metabolites formed 396 DALs that had higher levels in SP samples than those in VP samples. At the same time, PMe(16:1/18:1), PS(16:1/18:3), Co(Q9), MePC(8:0/22:5), PE(16:1/20:4), PMe(16:1/16:1), MePC(22:6/12:3), PE(16:1/20:5), and other metabolites constituted 404 DALs with lower abundances in SP samples.

As the largest category among these above lipid classes, GPs are critical in maintaining membrane integrity and cell homeostasis, as well as participating in cell growth and differentiation [32]. PCs occupy the highest proportion of GPs and can maintain the normal permeability of cell membranes. PEs, as the substrate for synthesizing PSs, can also be produced by the decarboxylation of PS in mitochondria [33]. Polyunsaturated fatty acids (PUFAs), such as docosahexaenoic acid (DHA), docosapentaenoic acid (DPA), and eicosapentaenoic acid (EPA), were mainly accumulated in PCs and PEs [34]. Low/high temperature and oxygen-induced lipid oxidation may be responsible for decreasing PCs and PEs in muscle tissue [35]. The abundances of PCs and PEs were the highest in FE samples, followed by VP samples, and the least in SP samples. Yu et al. reported that chilled storage notably reduced the abundant levels of PCs and PEs in whelks, consistent with our findings [36]. These results were possibly due to oxidative reactions occurring in muscle tissues caused by the cleavage of C-C species near functional groups and hydrogen rearrangement of α-bonds on the PC/PE chain [37]. PCs also have an emulsification function, which can fully mix moisture and protease in the organism to avoid rough aging and discharge excess lipids into smaller droplets [38]. This also explained that the decreased PC abundances were also accompanied by increased PV in pike eel muscle.

In this current study, SP and VP batches showed higher TGs abundant levels than the FE batch, and the abundances of TG in the SP batches were higher than those in the VP batches, suggesting that a large number of lipids in SP and VP samples hydrolyzed to produce TGs. TGs in the organism are broken down by a series of lipases to generate free fatty acids and glycerol. Glycerol can be involved in glucose metabolism or other metabolic pathways through enzyme-catalyzed reactions [39]. Free fatty acids are easily oxidized into hydrogen peroxide by enzymatic degradation, forming aldehydes, ketones, and other substances, which confirmed the increase in MDA value in pike eel muscle tissues [40].

### 3.5. Hierarchical Cluster and Potential Biomarkers Analyses

**Figure 6:**
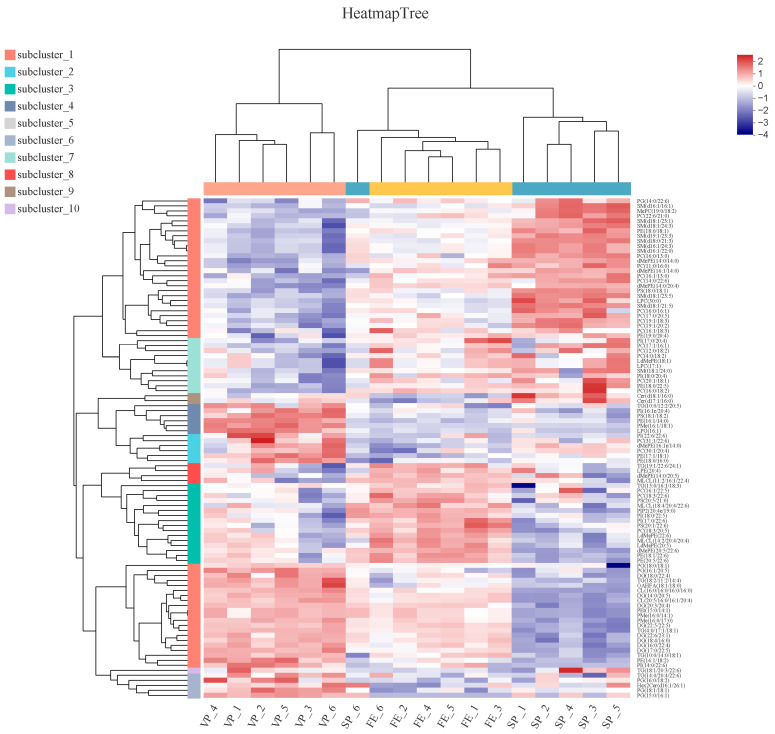
Hierarchical cluster and heat map analyses of the DALs (top 100 species) in fresh (FE), simple-packaged (SP), and vacuum-packaged (VP) pike eels. Each color block on the heat map corresponds to the abundance of different lipid species. Red (2) represents a relatively high abundance of lipids, and blue (−4) indicates a relatively low abundance of lipids.

The heat map visualization and hierarchical analysis of the identified DALs (the top 100 species) further clarified differences in lipid species and identified potential biomarkers among FE, SP, and VP samples (Figure 6). In general, FE, SP, and VP samples of six parallel were well distinguished, which validated the abovementioned PLS-DA analyses, suggesting the good stability, reliability, and repeatability of performed experimental procedures and analysis. However, SP6 did not cluster well with other SP samples. On the one hand, this might be caused by individual differences; on the other hand, the top 100 species of DALs were screened, which would have higher requirements for clustering. These clear separations of the three groups also indicated that the chilled storage and ClO_2_ combined with vacuum packaging had notably changed substantial DALs in pike eel muscle. The blue and red blocks in the plot represented relatively low and high abundance among FE, SP, and VP samples, respectively. The colored scales could be used as fingerprints to identify fresh or chilled simple-packaged or chilled vacuum-packaged pike eels according to the abundance of the DAL species. In our study, DALs, which were concentrated in PCs, PEs, TGs, and DGs, could be used as satisfactory biomarkers to evaluate the effect of ClO_2_ combined with vacuum packaging and distinguish the chilled stored sample from the fresh pike eel samples.

The noticeable lipid alterations between the different groups might be due to lipolysis, side-chain modification, backbone breakage, and/or lipid decomposition during chilled storage, resulting in the production of new intermediates and metabolites and the loss of several lipid profiles [41]. Whereas, the results obtained in this present research are still inadequate, and the detailed changes and mechanisms of lipid oxidation/hydrolysis and its intermediate and end products remain unclear and need further study.

## 4. Conclusions

Spectrophotometric- and LC/MS-based lipidomics analyses were conducted to illustrate the alterations in lipid profiles of FE, SP, and VP samples. Chemical results showed that ClO_2_ combined with vacuum packaging dramatically inhibited the increase in PV and MDA contents of lipids in pike eel muscle during chilled storage. The lipidomics analyses displayed that 354 higher and 420 lower abundant levels of DALs were identified in the SP vs. FE pike eels, 164 higher and 193 lower abundant levels of DALs were identified in the VP vs. FE pike eels, and 396 higher and 404 lower abundant levels of DALs were identified in the SP vs. VP pike eels, respectively. Among these DALs, several PCs, PEs, TGs, and DGs were more easily oxidized/hydrolyzed, which could be used as biomarkers to distinguish FE, SP, and VP pike eels. This work reveals the lipid composition and differences of pike eels under different storage methods, which provides a reference for controlling lipid oxidation in fatty fish.

## Data Availability

Data is contained within the article or supplementary material.

## Supplementary Materials

The following supporting information can be downloaded at https://www.mdpi.com/article/10.3390/foods12142791/s1: Table S1: Lipid classification and composition in fresh (FE), simple-packaged (SP), and vacuum-packaged (VP) pike eels via LC-MS/MS analysis; Table S2: The full names of the compound classification corresponding to Figure 4; Table S3: Higher abundant levels of lipid species in simple-packaged (SP) pike eels in comparison with fresh (FE) pike eels via LC-MS/MS analysis; Table S4: Lower abundant levels of lipid species in simple-packaged (SP) pike eels in comparison with fresh (FE) pike eels via LC-MS/MS analysis; Table S5: Higher abundant levels of lipid species in vacuum-packaged (VP) pike eels in comparison with fresh (FE) pike eels via LC-MS/MS analysis; Table S6: Lower abundant levels of lipid species in vacuum-packaged (VP) pike eels in comparison with fresh (FE) pike eels via LC-MS/MS analysis; Table S7: Higher abundant levels of lipid species in simple-packaged (SP) pike eels in comparison with vacuum-packaged (VP) pike eels via LC-MS/MS analysis; and Table S8: Lower abundant levels of lipid species in simple-packaged (SP) pike eels in comparison with vacuum-packaged (VP) pike eels via LC-MS/MS analysis.
