# Supplementary material for: Spectrophotometric- and LC/MS-Based Lipidomics Analyses Revealed Changes in Lipid Profiles of Pike Eel (Muraenesox cinereus) Treated with Stable Chlorine Dioxides and Vacuum-Packed during Chilled Storage
Source: Foods. 2023 Jul 22;12(14):2791. doi: 10.3390/foods12142791 (PMC10379090; doi:10.3390/foods12142791)
Supplement: Supplementary file 1 [file foods-12-02791-s001.zip › foods-2516484-supplementary.pdf]

Supplementary Material

Spectrophotometric- and LC/MS-Based Lipidomics Analyses Revealed Changes in Lipid Profiles of Pike Eel (*Muraenesox cinereus*) Treated with Stable Chlorine Dioxides and Vacuum-Packed during Chilled Storage

Shanshan Shui [1,2,3], Yingru Wu [2], Xiaonan Chen [2], Ruixue Li [4], Huicheng Yang [3,*], Baiyi Lu [1] and Bin Zhang [2,*]

1 Department of Food Science and Nutrition, College of Biosystems Engineering and Food Science, Zhejiang University, Hangzhou 310058, China
2 Key Laboratory of Health Risk Factors for Seafood of Zhejiang Province, College of Food Science and Pharmacy, Zhejiang Ocean University, Zhoushan 316022, China
3 Zhejiang Marine Development Research Institute, Zhoushan 316022, China
4 Comprehensive Technical Service Center of Zhoushan Customs, Zhoushan 316000, China
* Correspondence: huichengy@163.com (H.Y.); zhangbin@zjou.edu.cn or zhangbin_ouc@163.com (B.Z.); Tel.: +86-0580-255-4781 (B.Z.)

1 Supplementary Tables

1.1 Table S1. Lipid classification and composition in fresh (FE), simple-packaged (SP), and vacuum-packaged (VP) pike eels via LC-MS/MS analysis.

ID	Lipids	Mode	M/Z	Formula
pos_1	AEA(15:1)	pos	301.2850	C17 H37 O2 N2
pos_2	AcCa(13:0)	pos	358.2952	C20 H40 O4 N1
pos_4	BisMePA(10:1e/18:1)	pos	625.4204	C33 H63 O7 N0 P1 Na1
pos_6	BisMePA(16:2e/17:1)	pos	688.5276	C38 H75 O7 N1 P1
pos_7	BisMePA(16:2e/18:1)	pos	702.5432	C39 H77 O7 N1 P1
pos_8	BisMePA(12:0e/22:6)	pos	701.4517	C39 H67 O7 N0 P1 Na1
pos_9	BisMePA(18:2e/17:1)	pos	716.5589	C40 H79 O7 N1 P1
pos_10	BisMePA(18:3e/17:1)	pos	714.5432	C40 H77 O7 N1 P1
pos_11	BisMePA(18:2e/18:1)	pos	730.5745	C41 H81 O7 N1 P1

pos_12	BisMePA(18:3e/18:1)	pos	728.5589	C41 H79 O7 N1 P1
pos_13	BisMePA(16:0/20:5)	pos	740.5225	C41 H75 O8 N1 P1
pos_14	BisMePA(16:2e/20:4)	pos	724.5276	C41 H75 O7 N1 P1
pos_15	BisMePA(18:2e/20:0)	pos	760.6215	C43 H87 O7 N1 P1
pos_16	BisMePA(18:0/20:5)	pos	768.5538	C43 H79 O8 N1 P1
pos_18	BisMePA(16:2e/22:4)	pos	752.5589	C43 H79 O7 N1 P1
pos_19	BisMePA(18:2e/20:4)	pos	752.5589	C43 H79 O7 N1 P1
pos_20	BisMePA(18:3e/20:4)	pos	750.5432	C43 H77 O7 N1 P1
pos_21	BisMePA(20:5/20:5)	pos	786.5068	C45 H73 O8 N1 P1
pos_22	BisMePA(18:2e/22:4)	pos	780.5902	C45 H83 O7 N1 P1
pos_23	BisMePA(20:2e/20:4)	pos	780.5902	C45 H83 O7 N1 P1
pos_24	BisMePA(18:3e/22:4)	pos	778.5745	C45 H81 O7 N1 P1
pos_25	BisMePA(18:3e/22:5)	pos	776.5589	C45 H79 O7 N1 P1
pos_26	BisMePA(22:5/22:6)	pos	840.5538	C49 H79 O8 N1 P1
pos_27	BisMePA(22:6/22:6)	pos	838.5381	C49 H77 O8 N1 P1
pos_28	BisMePA(30:0/15:0)	pos	876.7416	C50 H103 O8 N1 P1
pos_29	CL(20:5/20:4/22:4/24:1)	pos	1634.1047	C95 H159 O17 P2
pos_30	Cer(d16:0/12:0)	pos	456.4411	C28 H58 O3 N1
pos_31	Cer(d12:0/16:0)	pos	456.4411	C28 H58 O3 N1
pos_33	Cer(d12:0/18:0)	pos	484.4724	C30 H62 O3 N1
pos_34	Cer(d16:1/14:0)	pos	464.4462	C30 H58 O2 N1
pos_35	Cer(d16:0/16:0)	pos	512.5037	C32 H66 O3 N1
pos_36	Cer(d16:1/16:0)	pos	492.4775	C32 H62 O2 N1

pos_37	Cer(d17:1/16:0)	pos	506.4932	C33 H64 O2 N1
pos_38	Cer(d16:0/18:0)	pos	540.5350	C34 H70 O3 N1
pos_39	Cer(d16:0/18:0+O)	pos	556.5299	C34 H70 O4 N1
pos_40	Cer(d16:0/18:1)	pos	538.5194	C34 H68 O3 N1
pos_41	Cer(d18:1/16:0)	pos	538.5194	C34 H68 O3 N1
pos_42	Cer(d16:0/18:2)	pos	536.5037	C34 H66 O3 N1
pos_43	Cer(d18:0/18:0)	pos	568.5663	C36 H74 O3 N1
pos_45	Cer(d16:0/20:1)	pos	566.5507	C36 H72 O3 N1
pos_46	Cer(d16:1/20:0)	pos	566.5507	C36 H72 O3 N1
pos_47	Cer(d20:0/18:0)	pos	596.5976	C38 H78 O3 N1
pos_48	Cer(d16:1/22:0)	pos	594.5820	C38 H76 O3 N1
pos_49	Cer(d16:1/22:1)	pos	592.5663	C38 H74 O3 N1
pos_50	Cer(d16:0/22:6)	pos	584.5037	C38 H66 O3 N1
pos_51	Cer(d16:1/23:0)	pos	608.5976	C39 H78 O3 N1
pos_52	Cer(d16:1/23:1)	pos	588.5714	C39 H74 O2 N1
pos_53	Cer(d22:0/18:0)	pos	624.6289	C40 H82 O3 N1
pos_54	Cer(d16:0/24:1)	pos	622.6133	C40 H80 O3 N1
pos_55	Cer(d18:1/22:0)	pos	622.6133	C40 H80 O3 N1
pos_56	Cer(d16:1/24:1)	pos	602.5871	C40 H76 O2 N1
pos_57	Cer(d16:1/24:2)	pos	618.5820	C40 H76 O3 N1
pos_58	Cer(d17:1/23:3)	pos	616.5663	C40 H74 O3 N1
pos_59	Cer(d19:1/22:0)	pos	636.6289	C41 H82 O3 N1
pos_60	Cer(d18:1/23:0)	pos	618.6184	C41 H80 O2 N1

pos_62	Cer(d19:1/23:0)	pos	632.6340	C42 H82 O2 N1
pos_63	Cer(d18:1/24:0)	pos	650.6446	C42 H84 O3 N1
pos_64	Cer(d16:1/26:1)	pos	630.6184	C42 H80 O2 N1
pos_65	Cer(d18:1/24:1)	pos	648.6289	C42 H82 O3 N1
pos_66	Cer(d18:2/24:1)	pos	628.6027	C42 H78 O2 N1
pos_67	Cer(d19:1/23:3)	pos	644.5976	C42 H78 O3 N1
pos_68	Cer(d17:1/26:1)	pos	662.6446	C43 H84 O3 N1
pos_69	Cer(d19:2/24:1)	pos	660.6289	C43 H82 O3 N1
pos_70	Cer(d18:1/26:1)	pos	658.6497	C44 H84 O2 N1
pos_71	Cer(m18:0/16:0)	pos	524.5401	C34 H70 O2 N1
pos_72	Cer(m18:0/20:0)	pos	580.6027	C38 H78 O2 N1
pos_73	Cer(m18:0/22:0)	pos	608.6340	C40 H82 O2 N1
pos_74	Cer(m17:0/24:1)	pos	620.6340	C41 H82 O2 N1
pos_75	Cer(m18:0/24:0)	pos	636.6653	C42 H86 O2 N1
pos_76	Cer(m18:0/24:1)	pos	634.6497	C42 H84 O2 N1
pos_77	Cer(t16:0/14:0)	pos	464.4462	C30 H58 O2 N1
pos_78	Cer(t16:0/16:0)	pos	528.4986	C32 H66 O4 N1
pos_79	Cer(t17:0/16:0)	pos	506.4932	C33 H64 O2 N1
pos_80	Cer(t18:0/16:0)	pos	556.5299	C34 H70 O4 N1
pos_81	Cer(t20:0/16:0)	pos	584.5612	C36 H74 O4 N1
pos_82	Cer(t16:0/20:0)	pos	548.5401	C36 H70 O2 N1
pos_83	Cer(t18:0/18:0+O)	pos	600.5562	C36 H74 O5 N1
pos_84	Cer(t18:0/18:1)	pos	582.5456	C36 H72 O4 N1

pos_85	Cer(t16:0/21:0)	pos	562.5558	C37 H72 O2 N1
pos_86	Cer(t16:0/22:0)	pos	594.5820	C38 H76 O3 N1
pos_87	Cer(t16:0/22:1)	pos	574.5558	C38 H72 O2 N1
pos_88	Cer(t16:0/22:3)	pos	588.5350	C38 H70 O3 N1
pos_89	Cer(t16:0/23:0)	pos	608.5976	C39 H78 O3 N1
pos_90	Cer(t16:0/23:5)	pos	598.5194	C39 H68 O3 N1
pos_91	Cer(t16:0/24:0)	pos	622.6133	C40 H80 O3 N1
pos_92	Cer(t16:0/24:1)	pos	620.5976	C40 H78 O3 N1
pos_93	Cer(t17:0/23:3)	pos	616.5663	C40 H74 O3 N1
pos_94	Cer(t17:0/24:0)	pos	618.6184	C41 H80 O2 N1
pos_95	Cer(t18:0/23:3)	pos	630.5820	C41 H76 O3 N1
pos_96	Cer(t18:0/24:3)	pos	644.5976	C42 H78 O3 N1
pos_99	ChE(16:0)	pos	642.6184	C43 H80 O2 N1
pos_102	ChE(18:0)	pos	670.6497	C45 H84 O2 N1
pos_103	ChE(18:1)	pos	668.6340	C45 H82 O2 N1
pos_105	ChE(19:1)	pos	682.6497	C46 H84 O2 N1
pos_106	ChE(20:1)	pos	696.6653	C47 H86 O2 N1
pos_107	ChE(20:2)	pos	694.6497	C47 H84 O2 N1
pos_110	ChE(20:5)	pos	671.5762	C47 H75 O2
pos_111	ChE(22:1)	pos	724.6966	C49 H90 O2 N1
pos_112	ChE(22:2)	pos	722.6810	C49 H88 O2 N1
pos_113	ChE(22:4)	pos	718.6497	C49 H84 O2 N1
pos_114	ChE(22:5)	pos	716.6340	C49 H82 O2 N1

pos_115	ChE(22:6)	pos	697.5918	C49 H77 O2
pos_116	ChE(24:1)	pos	752.7279	C51 H94 O2 N1
pos_117	ChE(24:2)	pos	750.7123	C51 H92 O2 N1
pos_118	ChE(30:5)	pos	828.7592	C57 H98 O2 N1
pos_119	Co(Q10)	pos	863.6912	C59 H91 O4
pos_120	Co(Q8)	pos	727.5660	C49 H75 O4
pos_121	Co(Q9)	pos	795.6286	C54 H83 O4
pos_122	DG(16:1/12:0)	pos	533.4176	C31 H58 O5 Na1
pos_123	DG(16:0/14:0)	pos	558.5092	C33 H68 O5 N1
pos_124	DG(16:1/14:0)	pos	556.4936	C33 H66 O5 N1
pos_125	DG(16:1/14:1)	pos	554.4779	C33 H64 O5 N1
pos_126	DG(18:4/12:0)	pos	555.4020	C33 H56 O5 Na1
pos_130	DG(16:0/16:0)	pos	586.5405	C35 H72 O5 N1
pos_131	DG(18:1/14:0)	pos	584.5249	C35 H70 O5 N1
pos_132	DG(16:0/16:1)	pos	584.5249	C35 H70 O5 N1
pos_133	DG(16:1e/16:0)	pos	575.5010	C35 H68 O4 Na1
pos_134	DG(16:1/16:1)	pos	582.5092	C35 H68 O5 N1
pos_135	DG(14:0/18:3)	pos	563.4670	C35 H63 O5
pos_136	DG(18:4/14:0)	pos	578.4779	C35 H64 O5 N1
pos_137	DG(12:0/20:5)	pos	581.4176	C35 H58 O5 Na1
pos_138	DG(10:0/22:6)	pos	574.4466	C35 H60 O5 N1
pos_139	DG(16:0/17:0)	pos	600.5562	C36 H74 O5 N1
pos_140	DG(15:0/18:1)	pos	598.5405	C36 H72 O5 N1

pos_141	DG(17:1/16:0)	pos	598.5405	C36 H72 O5 N1
pos_142	DG(17:1/16:1)	pos	596.5249	C36 H70 O5 N1
pos_143	DG(15:0/18:3)	pos	594.5092	C36 H68 O5 N1
pos_144	DG(18:4/15:0)	pos	592.4936	C36 H66 O5 N1
pos_148	DG(18:0/16:1)	pos	612.5562	C37 H74 O5 N1
pos_149	DG(16:1/18:1)	pos	610.5405	C37 H72 O5 N1
pos_150	DG(16:1/18:2)	pos	608.5249	C37 H70 O5 N1
pos_151	DG(16:0/18:3)	pos	591.4983	C37 H67 O5
pos_152	DG(16:1/18:3)	pos	606.5092	C37 H68 O5 N1
pos_153	DG(18:4/16:0)	pos	589.4827	C37 H65 O5
pos_154	DG(18:3e/16:1)	pos	575.5034	C37 H67 O4
pos_155	DG(18:4/16:1)	pos	587.4670	C37 H63 O5
pos_156	DG(14:0/20:5)	pos	587.4670	C37 H63 O5
pos_157	DG(20:5/14:1)	pos	602.4779	C37 H64 O5 N1
pos_158	DG(12:0/22:6)	pos	602.4779	C37 H64 O5 N1
pos_160	DG(19:1/16:0)	pos	626.5718	C38 H76 O5 N1
pos_161	DG(17:0/18:1)	pos	626.5718	C38 H76 O5 N1
pos_162	DG(17:1/18:1)	pos	624.5562	C38 H74 O5 N1
pos_163	DG(17:1/18:2)	pos	622.5405	C38 H72 O5 N1
pos_164	DG(17:0/18:3)	pos	605.5140	C38 H69 O5
pos_165	DG(15:0/20:4)	pos	620.5249	C38 H70 O5 N1
pos_167	DG(22:6/13:0)	pos	616.4936	C38 H66 O5 N1
pos_168	DG(18:0/18:0)	pos	642.6031	C39 H80 O5 N1

pos_169	DG(18:0/18:1)	pos	645.5428	C39 H74 O5 Na1
pos_170	DG(16:0/20:1)	pos	640.5875	C39 H78 O5 N1
pos_171	DG(18:1/18:1)	pos	643.5272	C39 H72 O5 Na1
pos_172	DG(18:0/18:2)	pos	638.5718	C39 H76 O5 N1
pos_173	DG(18:1/18:2)	pos	636.5562	C39 H74 O5 N1
pos_178	DG(18:3e/18:1)	pos	603.5347	C39 H71 O4
pos_180	DG(16:0/20:5)	pos	632.5249	C39 H70 O5 N1
pos_181	DG(18:3e/18:2)	pos	601.5190	C39 H69 O4
pos_183	DG(22:5/14:1)	pos	635.4646	C39 H64 O5 Na1
pos_184	DG(16:1/20:5)	pos	630.5092	C39 H68 O5 N1
pos_185	DG(18:4/18:3)	pos	628.4936	C39 H66 O5 N1
pos_186	DG(22:6/14:1)	pos	628.4936	C39 H66 O5 N1
pos_187	DG(18:4/18:4)	pos	609.4514	C39 H61 O5
pos_188	DG(22:6/14:2)	pos	626.4779	C39 H64 O5 N1
pos_189	DG(22:6/14:3)	pos	624.4623	C39 H62 O5 N1
pos_190	DG(19:0/18:1)	pos	654.6031	C40 H80 O5 N1
pos_191	DG(17:0/20:1)	pos	654.6031	C40 H80 O5 N1
pos_193	DG(17:1/20:2)	pos	650.5718	C40 H76 O5 N1
pos_194	DG(17:0/20:3)	pos	633.5453	C40 H73 O5
pos_195	DG(15:0/22:4)	pos	648.5562	C40 H74 O5 N1
pos_197	DG(17:1/20:4)	pos	646.5405	C40 H72 O5 N1
pos_198	DG(15:0/22:5)	pos	629.5140	C40 H69 O5
pos_199	DG(17:0/20:5)	pos	646.5405	C40 H72 O5 N1

pos_200	DG(17:1/20:5)	pos	644.5249	C40 H70 O5 N1
pos_201	DG(15:0/22:6)	pos	644.5249	C40 H70 O5 N1
pos_202	DG(16:0/22:1)	pos	673.5741	C41 H78 O5 Na1
pos_203	DG(20:1/18:1)	pos	666.6031	C41 H80 O5 N1
pos_204	DG(16:1e/22:1)	pos	657.5792	C41 H78 O4 Na1
pos_205	DG(18:1/20:2)	pos	664.5875	C41 H78 O5 N1
pos_206	DG(18:1/20:3)	pos	645.5453	C41 H73 O5
pos_207	DG(16:0/22:4)	pos	662.5718	C41 H76 O5 N1
pos_208	DG(18:0/20:4)	pos	662.5718	C41 H76 O5 N1
pos_210	DG(18:0/20:5)	pos	660.5562	C41 H74 O5 N1
pos_211	DG(16:0/22:5)	pos	643.5296	C41 H71 O5
pos_212	DG(20:4e/18:1)	pos	629.5503	C41 H73 O4
pos_213	DG(18:1/20:5)	pos	641.5140	C41 H69 O5
pos_214	DG(16:1/22:5)	pos	641.5140	C41 H69 O5
pos_215	DG(16:0/22:6)	pos	658.5405	C41 H72 O5 N1
pos_216	DG(18:3/20:4)	pos	639.4983	C41 H67 O5
pos_217	DG(16:1/22:6)	pos	639.4983	C41 H67 O5
pos_218	DG(18:3/20:5)	pos	637.4827	C41 H65 O5
pos_219	DG(18:4/20:5)	pos	635.4670	C41 H63 O5
pos_220	DG(17:1/22:1)	pos	680.6188	C42 H82 O5 N1
pos_221	DG(17:1/22:2)	pos	678.6031	C42 H80 O5 N1
pos_222	DG(19:0/20:3)	pos	678.6031	C42 H80 O5 N1
pos_224	DG(19:0/20:4)	pos	676.5875	C42 H78 O5 N1

pos_225	DG(17:0/22:5)	pos	674.5718	C42 H76 O5 N1
pos_226	DG(17:1/22:4)	pos	657.5453	C42 H73 O5
pos_227	DG(17:1/22:5)	pos	672.5562	C42 H74 O5 N1
pos_228	DG(17:0/22:6)	pos	672.5562	C42 H74 O5 N1
pos_229	DG(17:1/22:6)	pos	670.5405	C42 H72 O5 N1
pos_231	DG(18:4/22:6)	pos	661.4827	C43 H65 O5
pos_232	DG(18:1/22:1)	pos	694.6344	C43 H84 O5 N1
pos_233	DG(18:1/22:2)	pos	692.6188	C43 H82 O5 N1
pos_234	DG(18:3/22:1)	pos	690.6031	C43 H80 O5 N1
pos_235	DG(18:0/22:4)	pos	690.6031	C43 H80 O5 N1
pos_236	DG(20:0/20:4)	pos	690.6031	C43 H80 O5 N1
pos_237	DG(18:1/22:4)	pos	688.5875	C43 H78 O5 N1
pos_238	DG(20:1/20:4)	pos	671.5609	C43 H75 O5
pos_239	DG(18:0/22:5)	pos	688.5875	C43 H78 O5 N1
pos_240	DG(18:1/22:5)	pos	669.5453	C43 H73 O5
pos_241	DG(18:0/22:6)	pos	686.5718	C43 H76 O5 N1
pos_242	DG(20:3/20:4)	pos	689.5115	C43 H70 O5 Na1
pos_243	DG(22:5/18:2)	pos	667.5296	C43 H71 O5
pos_244	DG(18:1/22:6)	pos	684.5562	C43 H74 O5 N1
pos_245	DG(18:3/22:5)	pos	682.5405	C43 H72 O5 N1
pos_246	DG(18:2/22:6)	pos	682.5405	C43 H72 O5 N1
pos_247	DG(18:2e/22:6)	pos	651.5347	C43 H71 O4
pos_248	DG(18:3/22:6)	pos	680.5249	C43 H70 O5 N1

pos_249	DG(19:0/22:5)	pos	702.6031	C44 H80 O5 N1
pos_250	DG(19:1/22:5)	pos	700.5875	C44 H78 O5 N1
pos_251	DG(19:0/22:6)	pos	700.5875	C44 H78 O5 N1
pos_252	DG(19:1/22:6)	pos	698.5718	C44 H76 O5 N1
pos_254	DG(20:5/22:6)	pos	687.4983	C45 H67 O5
pos_256	DG(24:1/18:2)	pos	720.6501	C45 H86 O5 N1
pos_258	DG(20:1/22:4)	pos	716.6188	C45 H82 O5 N1
pos_260	DG(20:0/22:5)	pos	716.6188	C45 H82 O5 N1
pos_261	DG(20:1/22:5)	pos	697.5766	C45 H77 O5
pos_262	DG(20:0/22:6)	pos	714.6031	C45 H80 O5 N1
pos_264	DG(20:1/22:6)	pos	712.5875	C45 H78 O5 N1
pos_266	DG(20:2/22:6)	pos	710.5718	C45 H76 O5 N1
pos_267	DG(22:5/20:4)	pos	708.5562	C45 H74 O5 N1
pos_269	DG(22:5/21:1)	pos	728.6188	C46 H82 O5 N1
pos_270	DG(22:6/21:0)	pos	728.6188	C46 H82 O5 N1
pos_271	DG(22:6/21:1)	pos	726.6031	C46 H80 O5 N1
pos_272	DG(22:5/22:5)	pos	717.5453	C47 H73 O5
pos_274	DG(22:5/22:6)	pos	715.5296	C47 H71 O5
pos_275	DG(22:6/22:6)	pos	713.5140	C47 H69 O5
pos_276	DG(22:1/22:4)	pos	744.6501	C47 H86 O5 N1
pos_277	DG(20:4e/24:1)	pos	713.6442	C47 H85 O4
pos_279	DG(22:0/22:6)	pos	742.6344	C47 H84 O5 N1
pos_281	DG(22:1/22:6)	pos	740.6188	C47 H82 O5 N1

pos_282	DG(22:4/22:4)	pos	738.6031	C47 H80 O5 N1
pos_284	DG(22:4/22:5)	pos	736.5875	C47 H78 O5 N1
pos_285	DG(22:6/23:1)	pos	754.6344	C48 H84 O5 N1
pos_286	DG(24:1/22:5)	pos	770.6657	C49 H88 O5 N1
pos_287	DG(24:1/22:6)	pos	768.6501	C49 H86 O5 N1
pos_288	DG(24:2/22:6)	pos	766.6344	C49 H84 O5 N1
pos_289	DG(35:0/16:0)	pos	873.7672	C54 H106 O5 K1
pos_290	DG(33:0/18:1)	pos	850.8222	C54 H108 O5 N1
pos_291	DG(36:0/16:0)	pos	887.7828	C55 H108 O5 K1
pos_292	DG(37:0/16:0)	pos	901.7985	C56 H110 O5 K1
pos_293	DG(37:1/16:0)	pos	899.7828	C56 H108 O5 K1
pos_294	DG(38:0/16:0)	pos	915.8141	C57 H112 O5 K1
pos_295	DG(38:1/16:0)	pos	913.7985	C57 H110 O5 K1
pos_296	DG(34:1/20:4)	pos	889.7619	C57 H102 O5 Na1
pos_298	DG(33:1/24:1)	pos	932.9005	C60 H118 O5 N1
pos_299	Hex1Cer(d16:1/16:0)	pos	672.5409	C38 H74 O8 N1
pos_301	Hex1Cer(d16:1/22:0)	pos	756.6348	C44 H86 O8 N1
pos_302	Hex1Cer(d18:1/22:0)	pos	784.6661	C46 H90 O8 N1
pos_303	Hex1Cer(d16:1/24:1)	pos	782.6504	C46 H88 O8 N1
pos_304	Hex1Cer(d16:1/24:2)	pos	780.6348	C46 H86 O8 N1
pos_305	Hex1Cer(d17:1/24:1)	pos	778.6555	C47 H88 O7 N1
pos_306	Hex1Cer(d18:1/24:0)	pos	812.6974	C48 H94 O8 N1
pos_307	Hex1Cer(d16:1/26:1)	pos	810.6817	C48 H92 O8 N1

pos_308	Hex1Cer(d17:1/26:1)	pos	824.6974	C49 H94 O8 N1
pos_309	Hex1Cer(d18:1/26:1)	pos	838.7130	C50 H96 O8 N1
pos_310	Hex1Cer(t16:0/22:1)	pos	754.6191	C44 H84 O8 N1
pos_311	Hex1Cer(t16:0/24:1)	pos	782.6504	C46 H88 O8 N1
pos_312	Hex1Cer(t16:0/24:2)	pos	780.6348	C46 H86 O8 N1
pos_313	Hex1Cer(t16:0/26:1)	pos	810.6817	C48 H92 O8 N1
pos_314	Hex1Cer(t16:0/26:2)	pos	808.6661	C48 H90 O8 N1
pos_315	Hex1Cer(t18:0/24:3)	pos	806.6504	C48 H88 O8 N1
pos_318	Hex1Cer(t18:1/26:3)	pos	832.6661	C50 H90 O8 N1
pos_319	Hex1Cer(t20:0/26:4)	pos	860.6974	C52 H94 O8 N1
pos_320	Hex2Cer(d16:1/26:1)	pos	972.7346	C54 H102 O13 N1
pos_322	LPC(14:0e)	pos	454.3292	C22 H49 O6 N1 P1
pos_323	LPC(14:1e)	pos	452.3136	C22 H47 O6 N1 P1
pos_324	LPC(15:0)	pos	482.3241	C23 H49 O7 N1 P1
pos_325	LPC(16:0)	pos	496.3398	C24 H51 O7 N1 P1
pos_326	LPC(16:0e)	pos	482.3605	C24 H53 O6 N1 P1
pos_327	LPC(16:1)	pos	494.3241	C24 H49 O7 N1 P1
pos_328	LPC(16:1e)	pos	480.3449	C24 H51 O6 N1 P1
pos_329	LPC(16:2e)	pos	478.3292	C24 H49 O6 N1 P1
pos_330	LPC(17:0)	pos	510.3554	C25 H53 O7 N1 P1
pos_331	LPC(17:1)	pos	508.3398	C25 H51 O7 N1 P1
pos_333	LPC(18:0e)	pos	510.3918	C26 H57 O6 N1 P1
pos_334	LPC(18:1)	pos	522.3554	C26 H53 O7 N1 P1

pos_335	LPC(18:1e)	pos	508.3762	C26 H55 O6 N1 P1
pos_336	LPC(18:2)	pos	520.3398	C26 H51 O7 N1 P1
pos_337	LPC(18:2e)	pos	506.3605	C26 H53 O6 N1 P1
pos_338	LPC(18:3)	pos	540.3061	C26 H48 O7 N1 P1 Na1
pos_339	LPC(18:3e)	pos	504.3449	C26 H51 O6 N1 P1
pos_340	LPC(18:4)	pos	516.3085	C26 H47 O7 N1 P1
pos_341	LPC(19:0)	pos	538.3867	C27 H57 O7 N1 P1
pos_342	LPC(19:1)	pos	536.3711	C27 H55 O7 N1 P1
pos_343	LPC(20:0)	pos	552.4024	C28 H59 O7 N1 P1
pos_344	LPC(20:0e)	pos	538.4231	C28 H61 O6 N1 P1
pos_345	LPC(20:1)	pos	572.3687	C28 H56 O7 N1 P1 Na1
pos_346	LPC(20:1e)	pos	536.4075	C28 H59 O6 N1 P1
pos_347	LPC(20:2)	pos	570.3530	C28 H54 O7 N1 P1 Na1
pos_349	LPC(20:3)	pos	546.3554	C28 H53 O7 N1 P1
pos_350	LPC(20:3e)	pos	532.3762	C28 H55 O6 N1 P1
pos_351	LPC(20:4)	pos	566.3217	C28 H50 O7 N1 P1 Na1
pos_352	LPC(20:4e)	pos	530.3605	C28 H53 O6 N1 P1
pos_353	LPC(20:5)	pos	564.3061	C28 H48 O7 N1 P1 Na1
pos_355	LPC(22:0)	pos	580.4337	C30 H63 O7 N1 P1
pos_356	LPC(22:1)	pos	578.4180	C30 H61 O7 N1 P1
pos_358	LPC(22:3)	pos	574.3867	C30 H57 O7 N1 P1
pos_359	LPC(22:4)	pos	594.3530	C30 H54 O7 N1 P1 Na1
pos_360	LPC(22:5)	pos	592.3374	C30 H52 O7 N1 P1 Na1

pos_361	LPC(22:6)	pos	590.3217	C30 H50 O7 N1 P1 Na1
pos_362	LPC(23:0)	pos	594.4493	C31 H65 O7 N1 P1
pos_363	LPC(23:1)	pos	592.4337	C31 H63 O7 N1 P1
pos_364	LPC(24:0)	pos	608.4650	C32 H67 O7 N1 P1
pos_366	LPC(24:2)	pos	604.4337	C32 H63 O7 N1 P1
pos_367	LPC(26:1)	pos	634.4806	C34 H69 O7 N1 P1
pos_368	LPC(28:0)	pos	664.5276	C36 H75 O7 N1 P1
pos_369	LPC(28:1)	pos	662.5119	C36 H73 O7 N1 P1
pos_370	LPC(30:0)	pos	714.5408	C38 H78 O7 N1 P1 Na1
pos_371	LPC(30:1)	pos	712.5252	C38 H76 O7 N1 P1 Na1
pos_372	LPC(31:0)	pos	728.5565	C39 H80 O7 N1 P1 Na1
pos_373	LPC(32:0)	pos	742.5721	C40 H82 O7 N1 P1 Na1
pos_374	LPC(32:1)	pos	740.5565	C40 H80 O7 N1 P1 Na1
pos_375	LPC(33:1)	pos	754.5721	C41 H82 O7 N1 P1 Na1
pos_376	LPC(34:1)	pos	768.5878	C42 H84 O7 N1 P1 Na1
pos_377	LPE(16:0)	pos	476.2748	C21 H44 O7 N1 P1 Na1
pos_378	LPE(16:1)	pos	474.2591	C21 H42 O7 N1 P1 Na1
pos_379	LPE(16:1e)	pos	460.2798	C21 H44 O6 N1 P1 Na1
pos_380	LPE(18:0)	pos	504.3061	C23 H48 O7 N1 P1 Na1
pos_382	LPE(18:1e)	pos	488.3111	C23 H48 O6 N1 P1 Na1
pos_383	LPE(18:2e)	pos	486.2955	C23 H46 O6 N1 P1 Na1
pos_384	LPE(20:4)	pos	524.2748	C25 H44 O7 N1 P1 Na1
pos_386	LPE(22:6)	pos	548.2748	C27 H44 O7 N1 P1 Na1

pos_387	LPG(18:1)	pos	533.2850	C24 H47 O9 N0 P1 Na1
pos_388	LPS(18:1)	pos	524.2983	C24 H47 O9 N1 P1
pos_389	LPS(22:6)	pos	592.2646	C28 H44 O9 N1 P1 Na1
pos_390	LdMePE(18:1)	pos	508.3398	C25 H51 O7 N1 P1
pos_393	MePC(8:1e/8:0)	pos	525.3663	C25 H54 O7 N2 P1
pos_394	MePC(8:0/11:2)	pos	584.3323	C28 H52 O8 N1 P1 Na1
pos_395	MePC(8:0/11:3)	pos	582.3166	C28 H50 O8 N1 P1 Na1
pos_396	MePC(8:0e/11:4)	pos	566.3217	C28 H50 O7 N1 P1 Na1
pos_397	MePC(6:0/14:3)	pos	596.3323	C29 H52 O8 N1 P1 Na1
pos_398	MePC(8:0e/12:3)	pos	582.3530	C29 H54 O7 N1 P1 Na1
pos_400	MePC(11:0/18:2)	pos	747.5647	C40 H80 O8 N2 P1
pos_401	MePC(18:0e/11:4)	pos	729.5541	C40 H78 O7 N2 P1
pos_402	MePC(12:0/18:2)	pos	761.5803	C41 H82 O8 N2 P1
pos_403	MePC(10:0e/20:4)	pos	720.4939	C39 H72 O7 N1 P1 Na1
pos_404	MePC(8:0/22:5)	pos	732.4575	C39 H68 O8 N1 P1 Na1
pos_405	MePC(11:0/20:2)	pos	775.5960	C42 H84 O8 N2 P1
pos_406	MePC(11:0/20:3)	pos	773.5803	C42 H82 O8 N2 P1
pos_407	MePC(11:0/20:4)	pos	771.5647	C42 H80 O8 N2 P1
pos_408	MePC(20:0e/11:4)	pos	757.5854	C42 H82 O7 N2 P1
pos_409	MePC(16:0/16:1)	pos	763.5960	C41 H84 O8 N2 P1
pos_410	MePC(16:1/16:1)	pos	789.6116	C43 H86 O8 N2 P1
pos_411	MePC(14:0e/18:2)	pos	752.5565	C41 H80 O7 N1 P1 Na1
pos_412	MePC(10:0/22:3)	pos	759.5647	C41 H80 O8 N2 P1

pos_414	MePC(12:0/20:4)	pos	762.5044	C41 H74 O8 N1 P1 Na1
pos_415	MePC(12:0e/20:4)	pos	748.5252	C41 H76 O7 N1 P1 Na1
pos_416	MePC(12:1e/20:4)	pos	746.5095	C41 H74 O7 N1 P1 Na1
pos_417	MePC(10:0e/22:6)	pos	744.4939	C41 H72 O7 N1 P1 Na1
pos_418	MePC(15:0/18:2)	pos	775.5960	C42 H84 O8 N2 P1
pos_419	MePC(15:0/18:3)	pos	773.5803	C42 H82 O8 N2 P1
pos_420	MePC(20:4e/13:0)	pos	757.5854	C42 H82 O7 N2 P1
pos_421	MePC(11:0/22:5)	pos	797.5803	C44 H82 O8 N2 P1
pos_423	MePC(16:0/18:2)	pos	817.6429	C45 H90 O8 N2 P1
pos_424	MePC(16:1/18:2)	pos	815.6273	C45 H88 O8 N2 P1
pos_426	MePC(16:1/18:3)	pos	790.5357	C43 H78 O8 N1 P1 Na1
pos_427	MePC(14:0e/20:4)	pos	776.5565	C43 H80 O7 N1 P1 Na1
pos_429	MePC(14:1e/20:4)	pos	774.5408	C43 H78 O7 N1 P1 Na1
pos_430	MePC(12:0/22:6)	pos	786.5044	C43 H74 O8 N1 P1 Na1
pos_431	MePC(12:0e/22:6)	pos	772.5252	C43 H76 O7 N1 P1 Na1
pos_432	MePC(20:4/14:3)	pos	784.4888	C43 H72 O8 N1 P1 Na1
pos_434	MePC(20:4e/14:4)	pos	763.5385	C43 H76 O7 N2 P1
pos_435	MePC(22:6/12:3)	pos	780.4575	C43 H68 O8 N1 P1 Na1
pos_437	MePC(17:1/18:2)	pos	829.6429	C46 H90 O8 N2 P1
pos_438	MePC(15:0/20:5)	pos	797.5803	C44 H82 O8 N2 P1
pos_440	MePC(18:1/18:2)	pos	820.5827	C45 H84 O8 N1 P1 Na1
pos_441	MePC(18:1e/18:2)	pos	806.6034	C45 H86 O7 N1 P1 Na1
pos_442	MePC(16:0e/20:4)	pos	804.5878	C45 H84 O7 N1 P1 Na1

pos_443	MePC(16:1/20:4)	pos	816.5514	C45 H80 O8 N1 P1 Na1
pos_444	MePC(16:1e/20:4)	pos	802.5721	C45 H82 O7 N1 P1 Na1
pos_445	MePC(16:1/20:5)	pos	814.5357	C45 H78 O8 N1 P1 Na1
pos_446	MePC(14:0e/22:6)	pos	800.5565	C45 H80 O7 N1 P1 Na1
pos_447	MePC(18:4/18:3)	pos	812.5201	C45 H76 O8 N1 P1 Na1
pos_448	MePC(14:1e/22:6)	pos	798.5408	C45 H78 O7 N1 P1 Na1
pos_449	MePC(22:6/14:2)	pos	810.5044	C45 H74 O8 N1 P1 Na1
pos_450	MePC(19:0/18:2)	pos	836.6140	C46 H88 O8 N1 P1 Na1
pos_451	MePC(19:1/18:2)	pos	857.6742	C48 H94 O8 N2 P1
pos_454	MePC(18:0e/20:4)	pos	832.6191	C47 H88 O7 N1 P1 Na1
pos_455	MePC(18:1/20:4)	pos	844.5827	C47 H84 O8 N1 P1 Na1
pos_456	MePC(16:0/22:6)	pos	842.5670	C47 H82 O8 N1 P1 Na1
pos_457	MePC(16:0e/22:6)	pos	828.5878	C47 H84 O7 N1 P1 Na1
pos_458	MePC(16:1/22:6)	pos	840.5514	C47 H80 O8 N1 P1 Na1
pos_459	MePC(18:4/20:4)	pos	838.5357	C47 H78 O8 N1 P1 Na1
pos_460	MePC(16:2e/22:6)	pos	824.5565	C47 H80 O7 N1 P1 Na1
pos_461	MePC(18:4/20:5)	pos	836.5201	C47 H76 O8 N1 P1 Na1
pos_463	MePC(17:0/22:6)	pos	879.6586	C50 H92 O8 N2 P1
pos_465	MePC(20:1/20:4)	pos	872.6140	C49 H88 O8 N1 P1 Na1
pos_466	MePC(18:0/22:6)	pos	870.5983	C49 H86 O8 N1 P1 Na1
pos_467	MePC(18:1/22:6)	pos	868.5827	C49 H84 O8 N1 P1 Na1
pos_468	MePC(18:2/22:6)	pos	866.5670	C49 H82 O8 N1 P1 Na1
pos_469	MePC(18:3e/22:6)	pos	850.5721	C49 H82 O7 N1 P1 Na1

pos_470	MePC(20:4e/21:0)	pos	874.6660	C50 H94 O7 N1 P1 Na1
pos_471	MePC(20:0/22:6)	pos	898.6296	C51 H90 O8 N1 P1 Na1
pos_472	MePC(20:1/22:6)	pos	896.6140	C51 H88 O8 N1 P1 Na1
pos_473	MePC(20:2/22:6)	pos	894.5983	C51 H86 O8 N1 P1 Na1
pos_474	MePC(20:3/22:6)	pos	892.5827	C51 H84 O8 N1 P1 Na1
pos_475	PC(8:1e/6:0)	pos	466.2928	C22 H45 O7 N1 P1
pos_476	PC(12:0e/6:0)	pos	546.3530	C26 H54 O7 N1 P1 Na1
pos_477	PC(12:1e/6:0)	pos	544.3374	C26 H52 O7 N1 P1 Na1
pos_478	PC(8:0/11:2)	pos	548.3347	C27 H51 O8 N1 P1
pos_479	PC(8:0e/11:2)	pos	534.3554	C27 H53 O7 N1 P1
pos_480	PC(8:0e/11:3)	pos	532.3398	C27 H51 O7 N1 P1
pos_481	PC(8:0e/11:4)	pos	530.3241	C27 H49 O7 N1 P1
pos_482	PC(8:1e/11:4)	pos	528.3085	C27 H47 O7 N1 P1
pos_484	PC(14:1e/6:0)	pos	572.3687	C28 H56 O7 N1 P1 Na1
pos_485	PC(6:0/14:2)	pos	562.3503	C28 H53 O8 N1 P1
pos_486	PC(6:0/14:3)	pos	560.3347	C28 H51 O8 N1 P1
pos_487	PC(8:0e/13:0)	pos	566.4180	C29 H61 O7 N1 P1
pos_488	PC(10:0/11:2)	pos	576.3660	C29 H55 O8 N1 P1
pos_489	PC(10:0e/11:3)	pos	560.3711	C29 H55 O7 N1 P1
pos_490	PC(10:0/11:4)	pos	572.3347	C29 H51 O8 N1 P1
pos_492	PC(10:1e/11:4)	pos	556.3398	C29 H51 O7 N1 P1
pos_493	PC(4:0/18:2)	pos	590.3816	C30 H57 O8 N1 P1
pos_494	PC(4:0/18:3)	pos	588.3660	C30 H55 O8 N1 P1

pos_495	PC(8:1e/15:0)	pos	592.4337	C31 H63 O7 N1 P1
pos_496	PC(12:0e/11:4)	pos	586.3867	C31 H57 O7 N1 P1
pos_497	PC(12:1e/11:4)	pos	584.3711	C31 H55 O7 N1 P1
pos_498	PC(12:0e/12:0)	pos	608.4650	C32 H67 O7 N1 P1
pos_499	PC(10:1e/14:4)	pos	598.3867	C32 H57 O7 N1 P1
pos_500	PC(8:1e/18:2)	pos	630.4493	C34 H65 O7 N1 P1
pos_501	PC(11:0/16:0)	pos	664.4912	C35 H71 O8 N1 P1
pos_502	PC(11:0/16:1)	pos	662.4755	C35 H69 O8 N1 P1
pos_503	PC(9:0/18:2)	pos	660.4599	C35 H67 O8 N1 P1
pos_504	PC(14:0/14:0)	pos	700.4888	C36 H72 O8 N1 P1 Na1
pos_505	PC(16:0/12:0)	pos	678.5068	C36 H73 O8 N1 P1
pos_506	PC(16:0/13:0)	pos	692.5225	C37 H75 O8 N1 P1
pos_507	PC(15:0/14:0)	pos	714.5044	C37 H74 O8 N1 P1 Na1
pos_508	PC(8:0e/21:0)	pos	678.5432	C37 H77 O7 N1 P1
pos_509	PC(16:1/13:0)	pos	690.5068	C37 H73 O8 N1 P1
pos_510	PC(8:1e/21:0)	pos	676.5276	C37 H75 O7 N1 P1
pos_511	PC(11:0/18:2)	pos	688.4912	C37 H71 O8 N1 P1
pos_512	PC(16:0/14:0)	pos	706.5381	C38 H77 O8 N1 P1
pos_513	PC(14:0e/16:0)	pos	692.5589	C38 H79 O7 N1 P1
pos_514	PC(16:1/14:0)	pos	726.5044	C38 H74 O8 N1 P1 Na1
pos_515	PC(16:0/14:1)	pos	704.5225	C38 H75 O8 N1 P1
pos_516	PC(12:0e/18:1)	pos	690.5432	C38 H77 O7 N1 P1
pos_517	PC(14:0e/16:1)	pos	690.5432	C38 H77 O7 N1 P1

pos_518	PC(12:0/18:2)	pos	702.5068	C38 H73 O8 N1 P1
pos_519	PC(16:1/14:1)	pos	702.5068	C38 H73 O8 N1 P1
pos_520	PC(14:1e/16:1)	pos	688.5276	C38 H75 O7 N1 P1
pos_521	PC(12:0e/18:2)	pos	688.5276	C38 H75 O7 N1 P1
pos_522	PC(8:0/22:3)	pos	700.4912	C38 H71 O8 N1 P1
pos_523	PC(15:0/16:0)	pos	720.5538	C39 H79 O8 N1 P1
pos_524	PC(17:0/14:0)	pos	720.5538	C39 H79 O8 N1 P1
pos_525	PC(8:0e/23:0)	pos	706.5745	C39 H81 O7 N1 P1
pos_526	PC(15:0/16:1)	pos	718.5381	C39 H77 O8 N1 P1
pos_528	PC(14:0e/17:1)	pos	704.5589	C39 H79 O7 N1 P1
pos_530	PC(17:1/14:1)	pos	716.5225	C39 H75 O8 N1 P1
pos_531	PC(16:2e/15:0)	pos	702.5432	C39 H77 O7 N1 P1
pos_532	PC(11:0/20:3)	pos	714.5068	C39 H73 O8 N1 P1
pos_533	PC(20:5/11:4)	pos	702.4129	C39 H61 O8 N1 P1
pos_534	PC(16:0/16:0)	pos	734.5694	C40 H81 O8 N1 P1
pos_536	PC(16:0/16:1)	pos	754.5357	C40 H78 O8 N1 P1 Na1
pos_537	PC(18:0/14:1)	pos	732.5538	C40 H79 O8 N1 P1
pos_538	PC(14:0e/18:1)	pos	718.5745	C40 H81 O7 N1 P1
pos_539	PC(16:1e/16:0)	pos	718.5745	C40 H81 O7 N1 P1
pos_540	PC(16:1/16:1)	pos	752.5201	C40 H76 O8 N1 P1 Na1
pos_541	PC(14:0e/18:2)	pos	716.5589	C40 H79 O7 N1 P1
pos_542	PC(16:1e/16:1)	pos	716.5589	C40 H79 O7 N1 P1
pos_543	PC(10:0/22:3)	pos	728.5225	C40 H75 O8 N1 P1

pos_544	PC(14:1e/18:2)	pos	714.5432	C40 H77 O7 N1 P1
pos_546	PC(12:0e/20:4)	pos	712.5276	C40 H75 O7 N1 P1
pos_547	PC(12:1e/20:4)	pos	710.5119	C40 H73 O7 N1 P1
pos_548	PC(10:0e/22:6)	pos	708.4963	C40 H71 O7 N1 P1
pos_550	PC(18:0/15:0)	pos	748.5851	C41 H83 O8 N1 P1
pos_551	PC(16:0/17:0)	pos	748.5851	C41 H83 O8 N1 P1
pos_554	PC(17:1/16:0)	pos	768.5514	C41 H80 O8 N1 P1 Na1
pos_555	PC(19:0/14:1)	pos	746.5694	C41 H81 O8 N1 P1
pos_556	PC(16:0e/17:1)	pos	732.5902	C41 H83 O7 N1 P1
pos_557	PC(15:0/18:2)	pos	744.5538	C41 H79 O8 N1 P1
pos_558	PC(16:2e/17:0)	pos	730.5745	C41 H81 O7 N1 P1
pos_559	PC(15:0/18:3)	pos	742.5381	C41 H77 O8 N1 P1
pos_560	PC(11:0/22:4)	pos	740.5225	C41 H75 O8 N1 P1
pos_561	PC(20:4/13:0)	pos	740.5225	C41 H75 O8 N1 P1
pos_563	PC(11:0/22:5)	pos	738.5068	C41 H73 O8 N1 P1
pos_564	PC(20:5/13:0)	pos	738.5068	C41 H73 O8 N1 P1
pos_565	PC(11:0/22:6)	pos	736.4912	C41 H71 O8 N1 P1
pos_567	PC(18:0e/16:0)	pos	748.6215	C42 H87 O7 N1 P1
pos_569	PC(16:1e/18:0)	pos	746.6058	C42 H85 O7 N1 P1
pos_572	PC(16:0/18:2)	pos	780.5514	C42 H80 O8 N1 P1 Na1
pos_573	PC(16:0e/18:2)	pos	744.5902	C42 H83 O7 N1 P1
pos_574	PC(16:1e/18:1)	pos	744.5902	C42 H83 O7 N1 P1
pos_576	PC(16:0/18:3)	pos	778.5357	C42 H78 O8 N1 P1 Na1

pos_577	PC(18:3e/16:0)	pos	742.5745	C42 H81 O7 N1 P1
pos_578	PC(16:1e/18:2)	pos	742.5745	C42 H81 O7 N1 P1
pos_579	PC(16:1/18:3)	pos	754.5381	C42 H77 O8 N1 P1
pos_581	PC(14:0e/20:4)	pos	740.5589	C42 H79 O7 N1 P1
pos_582	PC(18:4/16:1)	pos	752.5225	C42 H75 O8 N1 P1
pos_583	PC(14:0/20:5)	pos	774.5044	C42 H74 O8 N1 P1 Na1
pos_584	PC(20:4/14:1)	pos	752.5225	C42 H75 O8 N1 P1
pos_585	PC(12:0e/22:5)	pos	760.5252	C42 H76 O7 N1 P1 Na1
pos_586	PC(14:1e/20:4)	pos	738.5432	C42 H77 O7 N1 P1
pos_588	PC(12:0e/22:6)	pos	736.5276	C42 H75 O7 N1 P1
pos_589	PC(14:1e/20:5)	pos	736.5276	C42 H75 O7 N1 P1
pos_590	PC(12:1e/22:6)	pos	734.5119	C42 H73 O7 N1 P1
pos_591	PC(18:0/17:0)	pos	776.6164	C43 H87 O8 N1 P1
pos_592	PC(16:0/19:0)	pos	776.6164	C43 H87 O8 N1 P1
pos_593	PC(16:1/19:0)	pos	774.6007	C43 H85 O8 N1 P1
pos_595	PC(17:0/18:2)	pos	772.5851	C43 H83 O8 N1 P1
pos_596	PC(16:2e/19:0)	pos	758.6058	C43 H85 O7 N1 P1
pos_598	PC(17:0/18:3)	pos	770.5694	C43 H81 O8 N1 P1
pos_599	PC(16:2e/19:1)	pos	756.5902	C43 H83 O7 N1 P1
pos_601	PC(17:1/18:3)	pos	768.5538	C43 H79 O8 N1 P1
pos_602	PC(20:4e/15:0)	pos	754.5745	C43 H81 O7 N1 P1
pos_603	PC(15:0/20:5)	pos	766.5381	C43 H77 O8 N1 P1
pos_604	PC(22:6/13:0)	pos	764.5225	C43 H75 O8 N1 P1

pos_605	PC(20:0/16:0)	pos	790.6320	C44 H89 O8 N1 P1
pos_606	PC(18:0/18:0)	pos	790.6320	C44 H89 O8 N1 P1
pos_607	PC(18:0/18:1)	pos	788.6164	C44 H87 O8 N1 P1
pos_608	PC(16:0/20:1)	pos	788.6164	C44 H87 O8 N1 P1
pos_609	PC(18:0e/18:1)	pos	774.6371	C44 H89 O7 N1 P1
pos_610	PC(18:1/18:1)	pos	808.5827	C44 H84 O8 N1 P1 Na1
pos_612	PC(18:0e/18:2)	pos	772.6215	C44 H87 O7 N1 P1
pos_613	PC(16:0e/20:2)	pos	794.6034	C44 H86 O7 N1 P1 Na1
pos_614	PC(18:1/18:2)	pos	784.5851	C44 H83 O8 N1 P1
pos_615	PC(16:0/20:3)	pos	784.5851	C44 H83 O8 N1 P1
pos_616	PC(18:3e/18:0)	pos	770.6058	C44 H85 O7 N1 P1
pos_617	PC(18:1e/18:2)	pos	770.6058	C44 H85 O7 N1 P1
pos_619	PC(18:4/18:0)	pos	782.5694	C44 H81 O8 N1 P1
pos_620	PC(16:0e/20:4)	pos	768.5902	C44 H83 O7 N1 P1
pos_621	PC(16:2e/20:2)	pos	790.5721	C44 H82 O7 N1 P1 Na1
pos_622	PC(16:1/20:4)	pos	780.5538	C44 H79 O8 N1 P1
pos_623	PC(16:0/20:5)	pos	802.5357	C44 H78 O8 N1 P1 Na1
pos_624	PC(16:1e/20:4)	pos	766.5745	C44 H81 O7 N1 P1
pos_625	PC(16:2e/20:3)	pos	788.5565	C44 H80 O7 N1 P1 Na1
pos_626	PC(14:0e/22:5)	pos	788.5565	C44 H80 O7 N1 P1 Na1
pos_627	PC(14:1e/22:4)	pos	766.5745	C44 H81 O7 N1 P1
pos_628	PC(16:1/20:5)	pos	778.5381	C44 H77 O8 N1 P1
pos_629	PC(14:0/22:6)	pos	800.5201	C44 H76 O8 N1 P1 Na1

pos_632	PC(18:4/18:3)	pos	776.5225	C44 H75 O8 N1 P1
pos_633	PC(22:6/14:1)	pos	776.5225	C44 H75 O8 N1 P1
pos_634	PC(14:1e/22:6)	pos	762.5432	C44 H77 O7 N1 P1
pos_636	PC(16:0/21:0)	pos	804.6477	C45 H91 O8 N1 P1
pos_637	PC(16:1/21:0)	pos	802.6320	C45 H89 O8 N1 P1
pos_638	PC(19:0/18:2)	pos	800.6164	C45 H87 O8 N1 P1
pos_639	PC(19:1/18:2)	pos	798.6007	C45 H85 O8 N1 P1
pos_641	PC(18:3e/19:0)	pos	806.6034	C45 H86 O7 N1 P1 Na1
pos_642	PC(17:0/20:4)	pos	796.5851	C45 H83 O8 N1 P1
pos_643	PC(19:1/18:3)	pos	796.5851	C45 H83 O8 N1 P1
pos_644	PC(15:0/22:4)	pos	796.5851	C45 H83 O8 N1 P1
pos_645	PC(20:4e/17:0)	pos	782.6058	C45 H85 O7 N1 P1
pos_646	PC(17:1/20:4)	pos	794.5694	C45 H81 O8 N1 P1
pos_647	PC(15:0/22:5)	pos	816.5514	C45 H80 O8 N1 P1 Na1
pos_648	PC(17:0/20:5)	pos	816.5514	C45 H80 O8 N1 P1 Na1
pos_649	PC(20:4e/17:1)	pos	780.5902	C45 H83 O7 N1 P1
pos_650	PC(15:0/22:6)	pos	792.5538	C45 H79 O8 N1 P1
pos_651	PC(17:1/20:5)	pos	814.5357	C45 H78 O8 N1 P1 Na1
pos_653	PC(16:0/22:1)	pos	816.6477	C46 H91 O8 N1 P1
pos_654	PC(20:0/18:1)	pos	838.6296	C46 H90 O8 N1 P1 Na1
pos_655	PC(20:0e/18:1)	pos	802.6684	C46 H93 O7 N1 P1
pos_656	PC(20:1/18:1)	pos	836.6140	C46 H88 O8 N1 P1 Na1
pos_658	PC(20:0e/18:2)	pos	800.6528	C46 H91 O7 N1 P1

pos_659	PC(20:1/18:2)	pos	812.6164	C46 H87 O8 N1 P1
pos_662	PC(16:0/22:4)	pos	832.5827	C46 H84 O8 N1 P1 Na1
pos_663	PC(16:0e/22:4)	pos	796.6215	C46 H87 O7 N1 P1
pos_664	PC(18:2e/20:2)	pos	818.6034	C46 H86 O7 N1 P1 Na1
pos_665	PC(18:0e/20:4)	pos	796.6215	C46 H87 O7 N1 P1
pos_666	PC(18:1/20:4)	pos	808.5851	C46 H83 O8 N1 P1
pos_667	PC(16:0/22:5)	pos	808.5851	C46 H83 O8 N1 P1
pos_668	PC(18:0/20:5)	pos	830.5670	C46 H82 O8 N1 P1 Na1
pos_669	PC(16:2e/22:3)	pos	816.5878	C46 H84 O7 N1 P1 Na1
pos_670	PC(18:2e/20:3)	pos	816.5878	C46 H84 O7 N1 P1 Na1
pos_671	PC(16:0e/22:5)	pos	816.5878	C46 H84 O7 N1 P1 Na1
pos_673	PC(16:0/22:6)	pos	806.5694	C46 H81 O8 N1 P1
pos_674	PC(16:1/22:5)	pos	806.5694	C46 H81 O8 N1 P1
pos_675	PC(18:1/20:5)	pos	828.5514	C46 H80 O8 N1 P1 Na1
pos_676	PC(16:2e/22:4)	pos	814.5721	C46 H82 O7 N1 P1 Na1
pos_677	PC(16:0e/22:6)	pos	792.5902	C46 H83 O7 N1 P1
pos_679	PC(20:5/18:2)	pos	826.5357	C46 H78 O8 N1 P1 Na1
pos_681	PC(16:2e/22:5)	pos	812.5565	C46 H80 O7 N1 P1 Na1
pos_682	PC(18:3e/20:4)	pos	812.5565	C46 H80 O7 N1 P1 Na1
pos_684	PC(18:3/20:5)	pos	824.5201	C46 H76 O8 N1 P1 Na1
pos_685	PC(16:2e/22:6)	pos	788.5589	C46 H79 O7 N1 P1
pos_686	PC(18:4/20:5)	pos	822.5044	C46 H74 O8 N1 P1 Na1
pos_687	PC(16:1/23:0)	pos	830.6633	C47 H93 O8 N1 P1

pos_688	PC(15:0/24:2)	pos	828.6477	C47 H91 O8 N1 P1
pos_689	PC(17:0/22:3)	pos	826.6320	C47 H89 O8 N1 P1
pos_690	PC(19:1/20:2)	pos	826.6320	C47 H89 O8 N1 P1
pos_691	PC(19:0/20:4)	pos	824.6164	C47 H87 O8 N1 P1
pos_692	PC(19:1/20:3)	pos	824.6164	C47 H87 O8 N1 P1
pos_693	PC(20:4e/19:0)	pos	832.6191	C47 H88 O7 N1 P1 Na1
pos_694	PC(19:1/20:4)	pos	822.6007	C47 H85 O8 N1 P1
pos_695	PC(17:1/22:4)	pos	844.5827	C47 H84 O8 N1 P1 Na1
pos_696	PC(17:0/22:5)	pos	844.5827	C47 H84 O8 N1 P1 Na1
pos_697	PC(20:4e/19:1)	pos	808.6215	C47 H87 O7 N1 P1
pos_698	PC(17:0/22:6)	pos	820.5851	C47 H83 O8 N1 P1
pos_699	PC(17:1/22:5)	pos	842.5670	C47 H82 O8 N1 P1 Na1
pos_700	PC(17:1/22:6)	pos	818.5694	C47 H81 O8 N1 P1
pos_701	PC(26:1/14:0)	pos	844.6790	C48 H95 O8 N1 P1
pos_703	PC(20:5/20:5)	pos	848.5201	C48 H76 O8 N1 P1 Na1
pos_704	PC(18:4/22:6)	pos	826.5381	C48 H77 O8 N1 P1
pos_705	PC(16:0/24:2)	pos	842.6633	C48 H93 O8 N1 P1
pos_706	PC(16:1/24:2)	pos	840.6477	C48 H91 O8 N1 P1
pos_708	PC(20:0/20:4)	pos	838.6320	C48 H89 O8 N1 P1
pos_709	PC(20:0e/20:4)	pos	824.6528	C48 H91 O7 N1 P1
pos_710	PC(20:2e/20:2)	pos	846.6347	C48 H90 O7 N1 P1 Na1
pos_711	PC(20:1/20:4)	pos	836.6164	C48 H87 O8 N1 P1
pos_714	PC(18:3e/22:2)	pos	844.6191	C48 H88 O7 N1 P1 Na1

pos_715	PC(20:1e/20:4)	pos	822.6371	C48 H89 O7 N1 P1
pos_716	PC(18:2e/22:3)	pos	844.6191	C48 H88 O7 N1 P1 Na1
pos_717	PC(18:0/22:6)	pos	834.6007	C48 H85 O8 N1 P1
pos_718	PC(18:1/22:5)	pos	834.6007	C48 H85 O8 N1 P1
pos_719	PC(18:0e/22:6)	pos	820.6215	C48 H87 O7 N1 P1
pos_720	PC(18:2e/22:4)	pos	842.6034	C48 H86 O7 N1 P1 Na1
pos_721	PC(18:1/22:6)	pos	832.5851	C48 H83 O8 N1 P1
pos_722	PC(18:3e/22:4)	pos	840.5878	C48 H84 O7 N1 P1 Na1
pos_723	PC(18:1e/22:6)	pos	818.6058	C48 H85 O7 N1 P1
pos_724	PC(18:2/22:6)	pos	830.5694	C48 H81 O8 N1 P1
pos_726	PC(20:4e/20:4)	pos	816.5902	C48 H83 O7 N1 P1
pos_727	PC(18:2e/22:6)	pos	816.5902	C48 H83 O7 N1 P1
pos_728	PC(18:3e/22:5)	pos	838.5721	C48 H82 O7 N1 P1 Na1
pos_729	PC(20:5/20:4)	pos	850.5357	C48 H78 O8 N1 P1 Na1
pos_730	PC(18:3/22:6)	pos	828.5538	C48 H79 O8 N1 P1
pos_732	PC(18:3e/22:6)	pos	814.5745	C48 H81 O7 N1 P1
pos_733	PC(17:1/24:2)	pos	854.6633	C49 H93 O8 N1 P1
pos_735	PC(19:0/22:5)	pos	850.6320	C49 H89 O8 N1 P1
pos_736	PC(19:1/22:5)	pos	870.5983	C49 H86 O8 N1 P1 Na1
pos_737	PC(19:0/22:6)	pos	848.6164	C49 H87 O8 N1 P1
pos_738	PC(19:1/22:6)	pos	846.6007	C49 H85 O8 N1 P1
pos_740	PC(20:4/22:6)	pos	854.5694	C50 H81 O8 N1 P1
pos_741	PC(20:5/22:5)	pos	876.5514	C50 H80 O8 N1 P1 Na1

pos_742	PC(20:4e/22:6)	pos	840.5902	C50 H83 O7 N1 P1
pos_743	PC(20:5/22:6)	pos	874.5357	C50 H78 O8 N1 P1 Na1
pos_744	PC(26:1/16:1)	pos	870.6946	C50 H97 O8 N1 P1
pos_745	PC(20:0/22:4)	pos	866.6633	C50 H93 O8 N1 P1
pos_747	PC(20:0/22:5)	pos	864.6477	C50 H91 O8 N1 P1
pos_748	PC(31:1/11:4)	pos	864.6477	C50 H91 O8 N1 P1
pos_750	PC(20:0/22:6)	pos	862.6320	C50 H89 O8 N1 P1
pos_751	PC(20:0e/22:6)	pos	848.6528	C50 H91 O7 N1 P1
pos_752	PC(20:2e/22:4)	pos	870.6347	C50 H90 O7 N1 P1 Na1
pos_753	PC(20:1/22:6)	pos	860.6164	C50 H87 O8 N1 P1
pos_754	PC(20:1e/22:6)	pos	846.6371	C50 H89 O7 N1 P1
pos_755	PC(22:4/20:4)	pos	858.6007	C50 H85 O8 N1 P1
pos_756	PC(20:2/22:6)	pos	858.6007	C50 H85 O8 N1 P1
pos_757	PC(20:2e/22:6)	pos	844.6215	C50 H87 O7 N1 P1
pos_758	PC(20:3/22:6)	pos	856.5851	C50 H83 O8 N1 P1
pos_759	PC(22:5/20:4)	pos	878.5670	C50 H82 O8 N1 P1 Na1
pos_760	PC(20:3e/22:6)	pos	842.6058	C50 H85 O7 N1 P1
pos_761	PC(22:6/21:0)	pos	876.6477	C51 H91 O8 N1 P1
pos_762	PC(22:6/21:1)	pos	874.6320	C51 H89 O8 N1 P1
pos_763	PC(22:4/22:6)	pos	882.6007	C52 H85 O8 N1 P1
pos_764	PC(22:5/22:6)	pos	880.5851	C52 H83 O8 N1 P1
pos_765	PC(22:6/22:6)	pos	900.5514	C52 H80 O8 N1 P1 Na1
pos_766	PC(24:0/20:4)	pos	894.6946	C52 H97 O8 N1 P1

pos_768	PC(20:3e/24:2)	pos	878.6997	C52 H97 O7 N1 P1
pos_769	PC(22:0/22:6)	pos	890.6633	C52 H93 O8 N1 P1
pos_770	PC(20:4e/24:2)	pos	876.6841	C52 H95 O7 N1 P1
pos_771	PC(22:1/22:6)	pos	888.6477	C52 H91 O8 N1 P1
pos_774	PC(24:1/22:6)	pos	916.6790	C54 H95 O8 N1 P1
pos_775	PE(8:0e/8:0)	pos	454.2928	C21 H45 O7 N1 P1
pos_776	PE(8:1e/9:0)	pos	466.2928	C22 H45 O7 N1 P1
pos_778	PE(16:1/14:1)	pos	660.4599	C35 H67 O8 N1 P1
pos_779	PE(16:0/16:1)	pos	690.5068	C37 H73 O8 N1 P1
pos_780	PE(16:0p/16:1)	pos	674.5119	C37 H73 O7 N1 P1
pos_781	PE(16:1/16:1)	pos	710.4731	C37 H70 O8 N1 P1 Na1
pos_782	PE(16:0p/17:1)	pos	688.5276	C38 H75 O7 N1 P1
pos_783	PE(17:1/16:1)	pos	702.5068	C38 H73 O8 N1 P1
pos_784	PE(16:1e/17:1)	pos	710.5095	C38 H74 O7 N1 P1 Na1
pos_785	PE(16:0/18:1)	pos	740.5201	C39 H76 O8 N1 P1 Na1
pos_786	PE(16:0p/18:1)	pos	702.5432	C39 H77 O7 N1 P1
pos_787	PE(16:1/18:1)	pos	716.5225	C39 H75 O8 N1 P1
pos_788	PE(16:1e/18:1)	pos	724.5252	C39 H76 O7 N1 P1 Na1
pos_790	PE(16:1/18:2)	pos	736.4888	C39 H72 O8 N1 P1 Na1
pos_791	PE(14:0p/20:4)	pos	696.4963	C39 H71 O7 N1 P1
pos_792	PE(14:0/20:5)	pos	710.4755	C39 H69 O8 N1 P1
pos_793	PE(14:0p/20:5)	pos	694.4806	C39 H69 O7 N1 P1
pos_794	PE(18:0p/17:1)	pos	716.5589	C40 H79 O7 N1 P1

pos_795	PE(18:1e/17:1)	pos	738.5408	C40 H78 O7 N1 P1 Na1
pos_796	PE(18:1p/17:1)	pos	714.5432	C40 H77 O7 N1 P1
pos_797	PE(17:0/19:0)	pos	770.5670	C41 H82 O8 N1 P1 Na1
pos_798	PE(18:0/18:1)	pos	768.5514	C41 H80 O8 N1 P1 Na1
pos_799	PE(18:0p/18:1)	pos	730.5745	C41 H81 O7 N1 P1
pos_800	PE(18:1e/18:1)	pos	752.5565	C41 H80 O7 N1 P1 Na1
pos_801	PE(18:1p/18:1)	pos	728.5589	C41 H79 O7 N1 P1
pos_802	PE(16:0/20:4)	pos	762.5044	C41 H74 O8 N1 P1 Na1
pos_803	PE(16:0p/20:4)	pos	724.5276	C41 H75 O7 N1 P1
pos_804	PE(16:0/20:5)	pos	760.4888	C41 H72 O8 N1 P1 Na1
pos_805	PE(16:1e/20:4)	pos	746.5095	C41 H74 O7 N1 P1 Na1
pos_806	PE(16:1p/20:4)	pos	722.5119	C41 H73 O7 N1 P1
pos_807	PE(16:0p/20:5)	pos	722.5119	C41 H73 O7 N1 P1
pos_808	PE(16:1/20:5)	pos	736.4912	C41 H71 O8 N1 P1
pos_809	PE(14:0e/22:6)	pos	744.4939	C41 H72 O7 N1 P1 Na1
pos_810	PE(14:0p/22:6)	pos	720.4963	C41 H71 O7 N1 P1
pos_811	PE(14:1e/22:6)	pos	742.4782	C41 H70 O7 N1 P1 Na1
pos_812	PE(18:0/20:4)	pos	768.5538	C43 H79 O8 N1 P1
pos_813	PE(16:0p/22:4)	pos	752.5589	C43 H79 O7 N1 P1
pos_814	PE(18:0p/20:4)	pos	752.5589	C43 H79 O7 N1 P1
pos_815	PE(18:1p/20:4)	pos	750.5432	C43 H77 O7 N1 P1
pos_816	PE(18:0p/20:5)	pos	750.5432	C43 H77 O7 N1 P1
pos_817	PE(16:0/22:6)	pos	786.5044	C43 H74 O8 N1 P1 Na1

pos_818	PE(16:0e/22:6)	pos	772.5252	C43 H76 O7 N1 P1 Na1
pos_819	PE(18:1p/20:5)	pos	748.5276	C43 H75 O7 N1 P1
pos_820	PE(16:0p/22:6)	pos	748.5276	C43 H75 O7 N1 P1
pos_821	PE(16:1/22:6)	pos	762.5068	C43 H73 O8 N1 P1
pos_823	PE(16:1p/22:6)	pos	746.5119	C43 H73 O7 N1 P1
pos_824	PE(16:1/24:2)	pos	820.5827	C45 H84 O8 N1 P1 Na1
pos_825	PE(18:0p/22:4)	pos	780.5902	C45 H83 O7 N1 P1
pos_826	PE(20:0p/20:4)	pos	780.5902	C45 H83 O7 N1 P1
pos_827	PE(18:0/22:5)	pos	794.5694	C45 H81 O8 N1 P1
pos_828	PE(20:1e/20:4)	pos	802.5721	C45 H82 O7 N1 P1 Na1
pos_829	PE(18:1p/22:4)	pos	778.5745	C45 H81 O7 N1 P1
pos_830	PE(18:0p/22:5)	pos	778.5745	C45 H81 O7 N1 P1
pos_831	PE(18:0/22:6)	pos	792.5538	C45 H79 O8 N1 P1
pos_832	PE(18:2e/22:4)	pos	778.5745	C45 H81 O7 N1 P1
pos_833	PE(18:1p/22:5)	pos	776.5589	C45 H79 O7 N1 P1
pos_834	PE(18:0p/22:6)	pos	776.5589	C45 H79 O7 N1 P1
pos_835	PE(18:1/22:6)	pos	812.5201	C45 H76 O8 N1 P1 Na1
pos_836	PE(18:1e/22:6)	pos	798.5408	C45 H78 O7 N1 P1 Na1
pos_837	PE(18:2/22:6)	pos	810.5044	C45 H74 O8 N1 P1 Na1
pos_838	PE(18:2p/22:6)	pos	772.5276	C45 H75 O7 N1 P1
pos_839	PE(18:3/22:6)	pos	808.4888	C45 H72 O8 N1 P1 Na1
pos_840	PE(20:4/22:6)	pos	812.5225	C47 H75 O8 N1 P1
pos_841	PE(20:5/22:6)	pos	810.5068	C47 H73 O8 N1 P1

pos_842	PE(20:3/22:5)	pos	838.5357	C47 H78 O8 N1 P1 Na1
pos_843	PE(20:3/22:6)	pos	836.5201	C47 H76 O8 N1 P1 Na1
pos_844	PE(22:4/22:6)	pos	840.5538	C49 H79 O8 N1 P1
pos_845	PE(22:5/22:6)	pos	838.5381	C49 H77 O8 N1 P1
pos_846	PE(22:6/22:6)	pos	836.5225	C49 H75 O8 N1 P1
pos_847	PEt(15:0/12:0)	pos	607.4333	C32 H64 O8 N0 P1
pos_848	PEt(15:0/13:0)	pos	621.4490	C33 H66 O8 N0 P1
pos_849	PEt(15:0/14:0)	pos	635.4646	C34 H68 O8 N0 P1
pos_850	PEt(15:0/14:1)	pos	633.4490	C34 H66 O8 N0 P1
pos_852	PEt(15:0/16:1)	pos	661.4803	C36 H70 O8 N0 P1
pos_853	PEt(15:0/17:0)	pos	677.5116	C37 H74 O8 N0 P1
pos_854	PEt(17:1/15:0)	pos	675.4959	C37 H72 O8 N0 P1
pos_855	PEt(18:0/15:0)	pos	691.5272	C38 H76 O8 N0 P1
pos_856	PEt(15:0/18:1)	pos	689.5116	C38 H74 O8 N0 P1
pos_857	PEt(15:0/18:2)	pos	687.4959	C38 H72 O8 N0 P1
pos_858	PEt(15:0/19:0)	pos	705.5429	C39 H78 O8 N0 P1
pos_859	PEt(19:1/15:0)	pos	703.5272	C39 H76 O8 N0 P1
pos_860	PEt(20:0/15:0)	pos	719.5585	C40 H80 O8 N0 P1
pos_862	PEt(15:0/20:1)	pos	717.5429	C40 H78 O8 N0 P1
pos_863	PEt(15:0/20:2)	pos	715.5272	C40 H76 O8 N0 P1
pos_864	PEt(15:0/21:0)	pos	733.5742	C41 H82 O8 N0 P1
pos_865	PEt(15:0/22:0)	pos	747.5898	C42 H84 O8 N0 P1
pos_866	PEt(15:0/22:1)	pos	745.5742	C42 H82 O8 N0 P1

pos_867	PEt(15:0/22:2)	pos	743.5585	C42 H80 O8 N0 P1
pos_868	PEt(15:0/22:5)	pos	737.5116	C42 H74 O8 N0 P1
pos_869	PEt(15:0/22:6)	pos	735.4959	C42 H72 O8 N0 P1
pos_870	PEt(15:0/24:1)	pos	773.6055	C44 H86 O8 N0 P1
pos_871	PG(14:0/14:0)	pos	667.4545	C34 H68 O10 N0 P1
pos_872	PG(16:1/14:0)	pos	693.4701	C36 H70 O10 N0 P1
pos_873	PG(16:1/16:1)	pos	741.4677	C38 H71 O10 N0 P1 Na1
pos_874	PG(18:0/14:4)	pos	732.4810	C38 H71 O10 N1 P1
pos_875	PG(16:0/18:2)	pos	769.4990	C40 H75 O10 N0 P1 Na1
pos_877	PG(28:0/16:0)	pos	908.7314	C50 H103 O10 N1 P1
pos_878	PG(30:0/16:0)	pos	936.7627	C52 H107 O10 N1 P1
pos_879	PG(30:1/16:0)	pos	917.7205	C52 H102 O10 N0 P1
pos_880	PI(18:0/20:4)	pos	904.5910	C47 H87 O13 N1 P1
pos_881	PI(16:0/22:6)	pos	900.5597	C47 H83 O13 N1 P1
pos_882	PI(16:1/22:6)	pos	898.5440	C47 H81 O13 N1 P1
pos_883	PI(18:0/22:6)	pos	928.5910	C49 H87 O13 N1 P1
pos_884	PI(18:1/22:6)	pos	926.5753	C49 H85 O13 N1 P1
pos_886	PI(22:6/22:6)	pos	972.5597	C53 H83 O13 N1 P1
pos_887	PMe(16:0/12:0)	pos	607.4333	C32 H64 O8 N0 P1
pos_889	PMe(16:0/14:0)	pos	635.4646	C34 H68 O8 N0 P1
pos_890	PMe(16:0/14:1)	pos	633.4490	C34 H66 O8 N0 P1
pos_891	PMe(16:0/16:0)	pos	663.4959	C36 H72 O8 N0 P1
pos_892	PMe(16:0/16:1)	pos	661.4803	C36 H70 O8 N0 P1

pos_893	PMe(16:0/17:0)	pos	677.5116	C37 H74 O8 N0 P1
pos_894	PMe(17:1/16:0)	pos	675.4959	C37 H72 O8 N0 P1
pos_895	PMe(18:0/16:0)	pos	691.5272	C38 H76 O8 N0 P1
pos_896	PMe(16:0/18:1)	pos	689.5116	C38 H74 O8 N0 P1
pos_897	PMe(16:0/18:2)	pos	687.4959	C38 H72 O8 N0 P1
pos_899	PMe(19:1/16:0)	pos	703.5272	C39 H76 O8 N0 P1
pos_900	PMe(20:0/16:0)	pos	719.5585	C40 H80 O8 N0 P1
pos_901	PMe(18:0/18:1)	pos	717.5429	C40 H78 O8 N0 P1
pos_902	PMe(16:0/20:1)	pos	717.5429	C40 H78 O8 N0 P1
pos_903	PMe(16:0/20:2)	pos	715.5272	C40 H76 O8 N0 P1
pos_904	PMe(16:0/20:5)	pos	709.4803	C40 H70 O8 N0 P1
pos_905	PMe(16:0/21:0)	pos	733.5742	C41 H82 O8 N0 P1
pos_906	PMe(16:0/22:0)	pos	747.5898	C42 H84 O8 N0 P1
pos_907	PMe(16:0/22:1)	pos	745.5742	C42 H82 O8 N0 P1
pos_908	PMe(16:0/22:2)	pos	743.5585	C42 H80 O8 N0 P1
pos_909	PMe(16:0/22:6)	pos	735.4959	C42 H72 O8 N0 P1
pos_910	PMe(16:0/24:1)	pos	773.6055	C44 H86 O8 N0 P1
pos_911	PS(18:0/18:1)	pos	790.5593	C42 H81 O10 N1 P1
pos_912	PS(16:0/22:6)	pos	808.5123	C44 H75 O10 N1 P1
pos_913	PS(16:1/22:6)	pos	806.4967	C44 H73 O10 N1 P1
pos_914	PS(18:0/22:5)	pos	838.5593	C46 H81 O10 N1 P1
pos_915	PS(18:0/22:6)	pos	836.5436	C46 H79 O10 N1 P1
pos_916	PS(18:1/22:6)	pos	834.5280	C46 H77 O10 N1 P1

pos_917	PS(22:5/22:6)	pos	882.5280	C50 H77 O10 N1 P1
pos_918	PS(22:6/22:6)	pos	880.5123	C50 H75 O10 N1 P1
pos_919	SM(d14:0/16:0)	pos	649.5279	C35 H74 O6 N2 P1
pos_920	SM(d18:1/12:0)	pos	647.5123	C35 H72 O6 N2 P1
pos_921	SM(d18:1/13:0)	pos	661.5279	C36 H74 O6 N2 P1
pos_922	SM(d14:0/18:0)	pos	677.5592	C37 H78 O6 N2 P1
pos_923	SM(d16:1/16:0)	pos	697.5255	C37 H75 O6 N2 P1 Na1
pos_924	SM(d14:0/18:1)	pos	675.5436	C37 H76 O6 N2 P1
pos_925	SM(d16:1/16:1)	pos	673.5279	C37 H74 O6 N2 P1
pos_926	SM(d14:0/18:4)	pos	669.4966	C37 H70 O6 N2 P1
pos_927	SM(d17:0/16:0)	pos	691.5749	C38 H80 O6 N2 P1
pos_928	SM(d17:1/16:0)	pos	689.5592	C38 H78 O6 N2 P1
pos_930	SM(d16:1/17:1)	pos	687.5436	C38 H76 O6 N2 P1
pos_931	SM(d18:2/15:1)	pos	685.5279	C38 H74 O6 N2 P1
pos_932	SM(d18:0/16:0)	pos	705.5905	C39 H82 O6 N2 P1
pos_933	SM(d18:1/16:0)	pos	703.5749	C39 H80 O6 N2 P1
pos_934	SM(d18:2/16:0)	pos	701.5592	C39 H78 O6 N2 P1
pos_935	SM(d16:1/18:2)	pos	699.5436	C39 H76 O6 N2 P1
pos_936	SM(d16:1/18:3)	pos	697.5279	C39 H74 O6 N2 P1
pos_937	SM(d17:0/18:1)	pos	717.5905	C40 H82 O6 N2 P1
pos_938	SM(d17:1/18:1)	pos	715.5749	C40 H80 O6 N2 P1
pos_939	SM(d18:2/17:1)	pos	713.5592	C40 H78 O6 N2 P1
pos_940	SM(d14:0/21:4)	pos	711.5436	C40 H76 O6 N2 P1

pos_941	SM(d18:0/18:0)	pos	733.6218	C41 H86 O6 N2 P1
pos_944	SM(d18:2/18:1)	pos	727.5749	C41 H80 O6 N2 P1
pos_945	SM(d18:1/18:3)	pos	725.5592	C41 H78 O6 N2 P1
pos_946	SM(d18:2/18:3)	pos	723.5436	C41 H76 O6 N2 P1
pos_947	SM(d19:0/18:1)	pos	745.6218	C42 H86 O6 N2 P1
pos_948	SM(d19:1/18:1)	pos	743.6062	C42 H84 O6 N2 P1
pos_949	SM(d18:2/19:1)	pos	741.5905	C42 H82 O6 N2 P1
pos_950	SM(d14:0/23:4)	pos	739.5749	C42 H80 O6 N2 P1
pos_951	SM(d16:1/21:3)	pos	739.5749	C42 H80 O6 N2 P1
pos_952	SM(d14:0/23:6)	pos	735.5436	C42 H76 O6 N2 P1
pos_953	SM(d20:0/18:0)	pos	761.6531	C43 H90 O6 N2 P1
pos_954	SM(d20:0/18:1)	pos	759.6375	C43 H88 O6 N2 P1
pos_955	SM(d16:1/22:0)	pos	781.6194	C43 H87 O6 N2 P1 Na1
pos_956	SM(d20:1/18:1)	pos	757.6218	C43 H86 O6 N2 P1
pos_957	SM(d18:1/20:2)	pos	755.6062	C43 H84 O6 N2 P1
pos_958	SM(d16:1/22:2)	pos	755.6062	C43 H84 O6 N2 P1
pos_959	SM(d18:1/20:3)	pos	753.5905	C43 H82 O6 N2 P1
pos_961	SM(d18:2/20:5)	pos	747.5436	C43 H76 O6 N2 P1
pos_962	SM(d18:1/21:0)	pos	773.6531	C44 H90 O6 N2 P1
pos_963	SM(d18:0/21:2)	pos	771.6375	C44 H88 O6 N2 P1
pos_964	SM(d16:1/23:1)	pos	771.6375	C44 H88 O6 N2 P1
pos_965	SM(d18:1/21:2)	pos	769.6218	C44 H86 O6 N2 P1
pos_966	SM(d18:0/21:3)	pos	769.6218	C44 H86 O6 N2 P1

pos_968	SM(d18:1/21:5)	pos	763.5749	C44 H80 O6 N2 P1
pos_970	SM(d18:1/22:0)	pos	787.6688	C45 H92 O6 N2 P1
pos_971	SM(d16:1/24:1)	pos	807.6350	C45 H89 O6 N2 P1 Na1
pos_972	SM(d18:1/22:1)	pos	785.6531	C45 H90 O6 N2 P1
pos_973	SM(d18:1/22:2)	pos	783.6375	C45 H88 O6 N2 P1
pos_974	SM(d18:1/22:3)	pos	781.6218	C45 H86 O6 N2 P1
pos_975	SM(d16:1/24:3)	pos	781.6218	C45 H86 O6 N2 P1
pos_977	SM(d16:1/24:4)	pos	779.6062	C45 H84 O6 N2 P1
pos_978	SM(d18:1/22:5)	pos	777.5905	C45 H82 O6 N2 P1
pos_979	SM(d20:1/20:5)	pos	777.5905	C45 H82 O6 N2 P1
pos_980	SM(d18:1/22:6)	pos	775.5749	C45 H80 O6 N2 P1
pos_982	SM(d18:1/23:1)	pos	799.6688	C46 H92 O6 N2 P1
pos_983	SM(d18:1/23:2)	pos	797.6531	C46 H90 O6 N2 P1
pos_984	SM(d20:0/21:3)	pos	797.6531	C46 H90 O6 N2 P1
pos_985	SM(d18:1/23:3)	pos	795.6375	C46 H88 O6 N2 P1
pos_986	SM(d18:1/23:4)	pos	793.6218	C46 H86 O6 N2 P1
pos_987	SM(d18:1/23:5)	pos	791.6062	C46 H84 O6 N2 P1
pos_988	SM(d18:1/24:0)	pos	815.7001	C47 H96 O6 N2 P1
pos_989	SM(d18:1/24:1)	pos	813.6844	C47 H94 O6 N2 P1
pos_990	SM(d18:1/24:2)	pos	811.6688	C47 H92 O6 N2 P1
pos_991	SM(d18:1/24:3)	pos	809.6531	C47 H90 O6 N2 P1
pos_992	SM(d19:1/23:3)	pos	809.6531	C47 H90 O6 N2 P1
pos_993	SM(d18:2/24:3)	pos	807.6375	C47 H88 O6 N2 P1

pos_994	SM(d18:1/24:5)	pos	805.6218	C47 H86 O6 N2 P1
pos_995	SM(d19:0/24:2)	pos	827.7001	C48 H96 O6 N2 P1
pos_996	SM(d19:0/24:3)	pos	825.6844	C48 H94 O6 N2 P1
pos_997	SM(d19:0/24:4)	pos	823.6688	C48 H92 O6 N2 P1
pos_998	SM(d19:0/24:5)	pos	821.6531	C48 H90 O6 N2 P1
pos_999	SM(d19:0/24:6)	pos	819.6375	C48 H88 O6 N2 P1
pos_1000	SM(d19:1/24:6)	pos	817.6218	C48 H86 O6 N2 P1
pos_1001	SM(d20:0/24:2)	pos	841.7157	C49 H98 O6 N2 P1
pos_1002	SM(d20:0/24:3)	pos	839.7001	C49 H96 O6 N2 P1
pos_1004	SM(d20:1/24:3)	pos	837.6844	C49 H94 O6 N2 P1
pos_1005	SM(d20:0/24:5)	pos	835.6688	C49 H92 O6 N2 P1
pos_1006	SM(d20:0/24:6)	pos	833.6531	C49 H90 O6 N2 P1
pos_1007	SM(t18:0/14:0)	pos	693.5541	C37 H78 O7 N2 P1
pos_1008	SM(t18:1/14:0)	pos	691.5385	C37 H76 O7 N2 P1
pos_1009	SM(t18:0/16:0)	pos	721.5854	C39 H82 O7 N2 P1
pos_1010	SM(t18:1/21:5)	pos	779.5698	C44 H80 O7 N2 P1
pos_1011	SM(t18:1/22:0)	pos	803.6637	C45 H92 O7 N2 P1
pos_1012	SM(t18:1/22:1)	pos	801.6480	C45 H90 O7 N2 P1
pos_1013	SM(t18:1/22:2)	pos	799.6324	C45 H88 O7 N2 P1
pos_1014	SM(t18:1/22:3)	pos	797.6167	C45 H86 O7 N2 P1
pos_1015	SM(t18:1/22:6)	pos	797.5779	C45 H79 O7 N2 P1 Li1
pos_1016	SM(t18:1/23:6)	pos	805.5854	C46 H82 O7 N2 P1
pos_1017	SM(t18:1/24:0)	pos	831.6950	C47 H96 O7 N2 P1

pos_1019	SPH(d16:1)	pos	272.2584	C16 H34 O2 N1
pos_1020	SPH(d18:1)	pos	300.2897	C18 H38 O2 N1
pos_1021	SPH(d20:1)	pos	328.3210	C20 H42 O2 N1
pos_1022	SPH(d22:1)	pos	356.3523	C22 H46 O2 N1
pos_1024	SPH(t16:0)	pos	290.2690	C16 H36 O3 N1
pos_1025	SPH(t20:0)	pos	346.3316	C20 H44 O3 N1
pos_1026	SPHP(d18:0)	pos	364.2611	C18 H39 O4 N1 P1
pos_1027	SPHP(d20:0)	pos	392.2924	C20 H43 O4 N1 P1
pos_1028	StE(22:3)	pos	746.6810	C51 H88 O2 N1
pos_1029	StE(22:4)	pos	744.6653	C51 H86 O2 N1
pos_1030	StE(30:5)	pos	854.7749	C59 H100 O2 N1
pos_1031	TG(4:0/6:0/12:4)	pos	457.2561	C25 H38 O6 Na1
pos_1032	TG(6:0/9:0/10:1)	pos	598.4915	C34 H66 O6 N2
pos_1033	TG(9:0/10:0/10:0)	pos	579.4021	C32 H60 O6 K1
pos_1035	TG(6:0/13:0/13:0)	pos	621.4491	C35 H66 O6 K1
pos_1036	TG(8:0/10:2/14:0)	pos	579.4619	C35 H63 O6
pos_1037	TG(8:0/11:2/14:0)	pos	593.4776	C36 H65 O6
pos_1038	TG(4:0/15:0/15:0)	pos	649.4804	C37 H70 O6 K1
pos_1040	TG(16:1/6:0/12:2)	pos	605.4776	C37 H65 O6
pos_1041	TG(14:1e/9:0/11:3)	pos	589.4827	C37 H65 O5
pos_1042	TG(12:0e/6:0/17:1)	pos	626.5718	C38 H76 O5 N1
pos_1043	TG(12:1e/6:0/17:0)	pos	626.5718	C38 H76 O5 N1
pos_1044	TG(16:0/8:0/11:2)	pos	621.5089	C38 H69 O6

pos_1045	TG(16:0/8:0/11:3)	pos	619.4932	C38 H67 O6
pos_1046	TG(16:1/8:0/11:2)	pos	619.4932	C38 H67 O6
pos_1047	TG(14:0/10:3/11:2)	pos	615.4619	C38 H63 O6
pos_1048	TG(15:0/6:0/15:0)	pos	645.5640	C39 H74 O6 Li1
pos_1049	TG(16:0/10:0/10:2)	pos	635.5245	C39 H71 O6
pos_1050	TG(9:0/9:0/18:2)	pos	635.5245	C39 H71 O6
pos_1051	TG(12:1e/6:0/18:1)	pos	621.5453	C39 H73 O5
pos_1052	TG(16:1/10:0/10:2)	pos	633.5089	C39 H69 O6
pos_1053	TG(18:3e/8:0/10:0)	pos	619.5296	C39 H71 O5
pos_1054	TG(12:0e/6:0/18:3)	pos	619.5296	C39 H71 O5
pos_1055	TG(14:0/10:1/12:4)	pos	651.4595	C39 H64 O6 Na1
pos_1056	TG(14:0/11:3/11:3)	pos	627.4619	C39 H63 O6
pos_1057	TG(10:0/10:0/17:1)	pos	689.5117	C40 H74 O6 K1
pos_1058	TG(12:0e/10:1/15:0)	pos	654.6031	C40 H80 O5 N1
pos_1059	TG(16:0/10:0/11:2)	pos	649.5402	C40 H73 O6
pos_1060	TG(18:0/8:0/11:2)	pos	649.5402	C40 H73 O6
pos_1061	TG(14:1e/6:0/17:1)	pos	657.5428	C40 H74 O5 Na1
pos_1062	TG(8:0/11:2/18:1)	pos	647.5245	C40 H71 O6
pos_1064	TG(14:1e/11:3/12:3)	pos	625.4827	C40 H65 O5
pos_1065	TG(4:0/17:1/17:1)	pos	669.5640	C41 H74 O6 Li1
pos_1066	TG(16:0/11:1/11:1)	pos	663.5558	C41 H75 O6
pos_1067	TG(14:0/10:2/14:0)	pos	669.5640	C41 H74 O6 Li1
pos_1068	TG(12:1e/8:0/18:1)	pos	649.5766	C41 H77 O5

pos_1069	TG(18:1/10:1/10:1)	pos	661.5402	C41 H73 O6
pos_1070	TG(12:1e/6:0/20:2)	pos	647.5609	C41 H75 O5
pos_1071	TG(18:2e/10:1/10:1)	pos	645.5453	C41 H73 O5
pos_1072	TG(12:1e/10:4/16:0)	pos	660.5562	C41 H74 O5 N1
pos_1073	TG(12:0e/6:0/20:5)	pos	643.5296	C41 H71 O5
pos_1075	TG(16:1/10:1/12:4)	pos	677.4752	C41 H66 O6 Na1
pos_1077	TG(16:1/10:3/12:4)	pos	651.4619	C41 H63 O6
pos_1078	TG(14:0/10:4/14:4)	pos	651.4619	C41 H63 O6
pos_1079	TG(20:4e/8:0/10:4)	pos	637.4827	C41 H65 O5
pos_1080	TG(15:0/6:0/18:1)	pos	717.5430	C42 H78 O6 K1
pos_1081	TG(9:0/13:0/17:1)	pos	717.5430	C42 H78 O6 K1
pos_1082	TG(16:1/9:0/14:0)	pos	794.7106	C48 H94 O6 N2
pos_1083	TG(18:3/10:3/11:4)	pos	667.4544	C42 H60 O6 Li1
pos_1084	TG(4:0/17:1/18:1)	pos	715.5273	C42 H76 O6 K1
pos_1085	TG(20:1/8:0/11:2)	pos	675.5558	C42 H75 O6
pos_1086	TG(6:0/13:0/20:4)	pos	711.4960	C42 H72 O6 K1
pos_1087	TG(18:1/10:1/11:3)	pos	671.5245	C42 H71 O6
pos_1088	TG(18:1/10:1/11:4)	pos	669.5089	C42 H69 O6
pos_1089	TG(8:0/11:2/20:5)	pos	667.4932	C42 H67 O6
pos_1091	TG(12:0/14:0/14:0)	pos	712.6450	C43 H86 O6 N1
pos_1092	TG(16:1/10:0/14:0)	pos	710.6293	C43 H84 O6 N1
pos_1093	TG(18:0/11:1/11:1)	pos	691.5871	C43 H79 O6
pos_1095	TG(16:0/6:0/18:2)	pos	697.5953	C43 H78 O6 Li1

pos_1096	TG(18:1/11:1/11:1)	pos	689.5715	C43 H77 O6
pos_1097	TG(12:0e/6:0/22:4)	pos	673.5766	C43 H77 O5
pos_1098	TG(18:0/10:4/12:1)	pos	685.5402	C43 H73 O6
pos_1099	TG(12:0e/6:0/22:5)	pos	671.5609	C43 H75 O5
pos_1100	TG(12:0e/6:0/22:6)	pos	669.5453	C43 H73 O5
pos_1101	TG(16:1/10:3/14:4)	pos	679.4932	C43 H67 O6
pos_1102	TG(18:4/10:2/12:2)	pos	679.4932	C43 H67 O6
pos_1104	TG(9:0/14:0/18:1)	pos	822.7419	C50 H98 O6 N2
pos_1105	TG(20:3/10:3/11:4)	pos	695.4857	C44 H64 O6 Li1
pos_1106	TG(20:4/10:4/11:3)	pos	693.4701	C44 H62 O6 Li1
pos_1109	TG(16:1/11:3/14:0)	pos	816.6950	C50 H92 O6 N2
pos_1110	TG(8:0/11:2/22:5)	pos	695.5245	C44 H71 O6
pos_1111	TG(8:0/11:2/22:6)	pos	693.5089	C44 H69 O6
pos_1112	TG(17:1/10:4/14:4)	pos	691.4932	C44 H67 O6
pos_1113	TG(14:0/14:0/14:0)	pos	740.6763	C45 H90 O6 N1
pos_1114	TG(16:0/10:0/16:0)	pos	838.7732	C51 H102 O6 N2
pos_1115	TG(10:0/14:0/18:1)	pos	759.5899	C45 H84 O6 K1
pos_1116	TG(16:1/12:0/14:0)	pos	738.6606	C45 H88 O6 N1
pos_1117	TG(16:1/12:0/14:1)	pos	736.6450	C45 H86 O6 N1
pos_1119	TG(20:4e/6:0/16:0)	pos	701.6079	C45 H81 O5
pos_1120	TG(18:0/10:3/14:2)	pos	713.5715	C45 H77 O6
pos_1121	TG(10:0/12:2/20:4)	pos	711.5558	C45 H75 O6
pos_1122	TG(12:1e/8:0/22:5)	pos	697.5766	C45 H77 O5

pos_1123	TG(12:1e/10:0/20:5)	pos	697.5766	C45 H77 O5
pos_1124	TG(14:0e/6:0/22:6)	pos	697.5766	C45 H77 O5
pos_1125	TG(16:0/12:3/14:4)	pos	709.5402	C45 H73 O6
pos_1126	TG(10:0/12:2/20:5)	pos	709.5402	C45 H73 O6
pos_1127	TG(12:1e/8:0/22:6)	pos	695.5609	C45 H75 O5
pos_1128	TG(10:0/10:2/22:6)	pos	707.5245	C45 H71 O6
pos_1129	TG(15:0/14:0/14:0)	pos	754.6919	C46 H92 O6 N1
pos_1130	TG(11:0/16:0/16:0)	pos	852.7889	C52 H104 O6 N2
pos_1131	TG(9:0/10:0/24:1)	pos	773.6056	C46 H86 O6 K1
pos_1132	TG(16:1/13:0/14:0)	pos	752.6763	C46 H90 O6 N1
pos_1133	TG(16:0/11:1/16:0)	pos	850.7732	C52 H102 O6 N2
pos_1134	TG(22:6/10:1/11:4)	pos	715.4932	C46 H67 O6
pos_1138	TG(16:0/11:2/16:0)	pos	848.7576	C52 H100 O6 N2
pos_1139	TG(15:0/10:2/18:1)	pos	769.5743	C46 H82 O6 K1
pos_1140	TG(16:0/11:2/16:1)	pos	846.7419	C52 H98 O6 N2
pos_1141	TG(16:1/11:2/16:1)	pos	844.7263	C52 H96 O6 N2
pos_1142	TG(16:1/11:3/16:1)	pos	842.7106	C52 H94 O6 N2
pos_1143	TG(12:1e/9:0/22:6)	pos	709.5766	C46 H77 O5
pos_1144	TG(18:2/11:2/14:4)	pos	721.5402	C46 H73 O6
pos_1146	TG(18:0/10:0/16:0)	pos	866.8045	C53 H106 O6 N2
pos_1147	TG(16:1/14:0/14:0)	pos	771.6473	C47 H88 O6 Na1
pos_1148	TG(16:0/10:0/18:1)	pos	864.7889	C53 H104 O6 N2
pos_1149	TG(16:1/14:0/14:1)	pos	769.6317	C47 H86 O6 Na1

pos_1150	TG(16:1/10:0/18:1)	pos	862.7732	C53 H102 O6 N2
pos_1152	TG(16:0/14:0/14:3)	pos	762.6606	C47 H88 O6 N1
pos_1153	TG(16:1/14:0/14:3)	pos	760.6450	C47 H86 O6 N1
pos_1156	TG(16:1/14:0/14:4)	pos	856.7263	C53 H96 O6 N2
pos_1157	TG(16:0/10:4/18:2)	pos	854.7106	C53 H94 O6 N2
pos_1158	TG(12:1e/10:4/22:1)	pos	725.6079	C47 H81 O5
pos_1159	TG(22:5/11:1/11:1)	pos	737.5715	C47 H77 O6
pos_1161	TG(22:6/11:1/11:1)	pos	735.5558	C47 H75 O6
pos_1162	TG(12:0e/10:2/22:6)	pos	721.5766	C47 H77 O5
pos_1163	TG(15:0/14:0/16:0)	pos	782.7232	C48 H96 O6 N1
pos_1164	TG(15:0/14:0/16:1)	pos	780.7076	C48 H94 O6 N1
pos_1168	TG(16:1/12:1/17:1)	pos	776.6763	C48 H90 O6 N1
pos_1170	TG(16:0/11:2/18:1)	pos	874.7732	C54 H102 O6 N2
pos_1171	TG(18:4/13:0/14:0)	pos	774.6606	C48 H88 O6 N1
pos_1172	TG(15:0/14:3/16:1)	pos	774.6606	C48 H88 O6 N1
pos_1173	TG(16:1/11:2/18:1)	pos	872.7576	C54 H100 O6 N2
pos_1175	TG(16:1/9:0/20:4)	pos	870.7419	C54 H98 O6 N2
pos_1178	TG(16:0e/14:0/16:0)	pos	782.7596	C49 H100 O5 N1
pos_1179	TG(16:0/14:0/16:1)	pos	799.6786	C49 H92 O6 Na1
pos_1180	TG(16:1/14:0/16:1)	pos	797.6630	C49 H90 O6 Na1
pos_1181	TG(16:0/12:1/18:1)	pos	890.8045	C55 H106 O6 N2
pos_1182	TG(16:1/14:1/16:1)	pos	795.6473	C49 H88 O6 Na1
pos_1183	TG(16:0/14:2/16:1)	pos	790.6919	C49 H92 O6 N1

pos_1184	TG(16:1/12:0/18:3)	pos	788.6763	C49 H90 O6 N1
pos_1185	TG(16:0/14:3/16:1)	pos	788.6763	C49 H90 O6 N1
pos_1187	TG(12:0/14:0/20:5)	pos	791.6160	C49 H84 O6 Na1
pos_1189	TG(16:1/10:0/20:5)	pos	789.6004	C49 H82 O6 Na1
pos_1191	TG(16:1/12:4/18:1)	pos	882.7419	C55 H98 O6 N2
pos_1192	TG(18:0/10:4/18:3)	pos	765.6028	C49 H81 O6
pos_1193	TG(16:1/8:0/22:6)	pos	782.6293	C49 H84 O6 N1
pos_1198	TG(16:0/14:0/17:1)	pos	813.6943	C50 H94 O6 Na1
pos_1199	TG(15:0/16:1/16:1)	pos	806.7232	C50 H96 O6 N1
pos_1200	TG(16:1/14:0/17:1)	pos	811.6786	C50 H92 O6 Na1
pos_1201	TG(11:0/18:1/18:1)	pos	904.8202	C56 H108 O6 N2
pos_1202	TG(16:1/14:1/17:1)	pos	804.7076	C50 H94 O6 N1
pos_1203	TG(11:0/16:0/20:3)	pos	902.8045	C56 H106 O6 N2
pos_1204	TG(16:1/13:0/18:3)	pos	802.6919	C50 H92 O6 N1
pos_1206	TG(18:4/13:0/16:1)	pos	800.6763	C50 H90 O6 N1
pos_1207	TG(14:0/13:0/20:5)	pos	800.6763	C50 H90 O6 N1
pos_1208	TG(9:0/18:1/20:4)	pos	898.7732	C56 H102 O6 N2
pos_1209	TG(18:1/11:3/18:1)	pos	898.7732	C56 H102 O6 N2
pos_1210	TG(16:0/11:3/20:2)	pos	789.6579	C50 H86 O6 Li1
pos_1211	TG(11:0/14:0/22:6)	pos	798.6606	C50 H88 O6 N1
pos_1212	TG(16:1/9:0/22:5)	pos	896.7576	C56 H100 O6 N2
pos_1213	TG(16:1/11:1/20:4)	pos	896.7576	C56 H100 O6 N2
pos_1214	TG(18:2/11:3/18:2)	pos	894.7419	C56 H98 O6 N2

pos_1215	TG(18:3/11:2/18:3)	pos	892.7263	C56 H96 O6 N2
pos_1216	TG(18:0/14:0/16:0)	pos	824.7702	C51 H102 O6 N1
pos_1217	TG(16:0/16:0/16:0)	pos	824.7702	C51 H102 O6 N1
pos_1218	TG(18:0e/14:0/16:0)	pos	810.7909	C51 H104 O5 N1
pos_1220	TG(16:0/16:0/16:1)	pos	827.7099	C51 H96 O6 Na1
pos_1221	TG(16:1e/16:0/16:0)	pos	808.7753	C51 H102 O5 N1
pos_1222	TG(16:1/14:0/18:1)	pos	825.6943	C51 H94 O6 Na1
pos_1224	TG(16:0e/16:1/16:1)	pos	806.7596	C51 H100 O5 N1
pos_1226	TG(16:1/16:1/16:1)	pos	823.6786	C51 H92 O6 Na1
pos_1227	TG(16:0/14:0/18:3)	pos	823.6786	C51 H92 O6 Na1
pos_1229	TG(16:1/14:0/18:3)	pos	821.6630	C51 H90 O6 Na1
pos_1230	TG(18:4/14:0/16:0)	pos	821.6630	C51 H90 O6 Na1
pos_1231	TG(18:4/14:0/16:1)	pos	819.6473	C51 H88 O6 Na1
pos_1232	TG(14:0/14:0/20:5)	pos	819.6473	C51 H88 O6 Na1
pos_1233	TG(16:1/14:3/18:1)	pos	912.7889	C57 H104 O6 N2
pos_1234	TG(16:0/14:4/18:1)	pos	912.7889	C57 H104 O6 N2
pos_1235	TG(16:1/12:0/20:5)	pos	817.6317	C51 H86 O6 Na1
pos_1236	TG(12:0/14:0/22:6)	pos	817.6317	C51 H86 O6 Na1
pos_1237	TG(18:4/14:1/16:1)	pos	795.6497	C51 H87 O6
pos_1238	TG(16:0/10:0/22:6)	pos	910.7732	C57 H102 O6 N2
pos_1239	TG(16:1/10:0/22:6)	pos	810.6606	C51 H88 O6 N1
pos_1240	TG(16:0/10:1/22:6)	pos	810.6606	C51 H88 O6 N1
pos_1242	TG(14:0/14:3/20:5)	pos	808.6450	C51 H86 O6 N1

pos_1243	TG(16:0/10:2/22:6)	pos	808.6450	C51 H86 O6 N1
pos_1244	TG(18:4/14:3/16:1)	pos	791.6184	C51 H83 O6
pos_1245	TG(15:0/11:2/22:6)	pos	906.7419	C57 H98 O6 N2
pos_1246	TG(16:0/16:0/17:0)	pos	838.7858	C52 H104 O6 N1
pos_1247	TG(18:0/15:0/16:0)	pos	838.7858	C52 H104 O6 N1
pos_1248	TG(16:0/16:1/17:0)	pos	836.7702	C52 H102 O6 N1
pos_1249	TG(15:0/16:0/18:1)	pos	841.7256	C52 H98 O6 Na1
pos_1251	TG(16:1e/16:0/17:0)	pos	822.7909	C52 H104 O5 N1
pos_1252	TG(16:0/16:1/17:1)	pos	839.7099	C52 H96 O6 Na1
pos_1254	TG(16:1/16:1/17:1)	pos	837.6943	C52 H94 O6 Na1
pos_1255	TG(18:1/13:0/18:2)	pos	930.8358	C58 H110 O6 N2
pos_1256	TG(15:0/16:1/18:3)	pos	830.7232	C52 H96 O6 N1
pos_1257	TG(15:0/14:0/20:4)	pos	830.7232	C52 H96 O6 N1
pos_1258	TG(11:0/16:0/22:4)	pos	928.8202	C58 H108 O6 N2
pos_1259	TG(18:1/11:1/20:2)	pos	928.8202	C58 H108 O6 N2
pos_1261	TG(15:0/14:0/20:5)	pos	833.6630	C52 H90 O6 Na1
pos_1263	TG(11:0/18:1/20:4)	pos	926.8045	C58 H106 O6 N2
pos_1264	TG(16:1/13:0/20:5)	pos	826.6919	C52 H92 O6 N1
pos_1265	TG(14:0/13:0/22:6)	pos	826.6919	C52 H92 O6 N1
pos_1267	TG(11:0/18:1/20:5)	pos	815.6735	C52 H88 O6 Li1
pos_1268	TG(11:0/16:1/22:6)	pos	824.6763	C52 H90 O6 N1
pos_1269	TG(18:1/11:2/20:4)	pos	922.7732	C58 H102 O6 N2
pos_1270	TG(15:0/12:2/22:6)	pos	822.6606	C52 H88 O6 N1

pos_1271	TG(16:1/11:2/22:5)	pos	920.7576	C58 H100 O6 N2
pos_1274	TG(16:0/16:0/18:1)	pos	850.7858	C53 H104 O6 N1
pos_1276	TG(18:1/10:3/22:6)	pos	930.7419	C59 H98 O6 N2
pos_1277	TG(16:1/14:4/20:5)	pos	930.7419	C59 H98 O6 N2
pos_1278	TG(16:0e/16:0/18:1)	pos	819.7800	C53 H103 O5
pos_1280	TG(16:0e/16:1/18:1)	pos	834.7909	C53 H104 O5 N1
pos_1281	TG(16:1e/16:0/18:1)	pos	834.7909	C53 H104 O5 N1
pos_1282	TG(18:1e/16:0/16:1)	pos	834.7909	C53 H104 O5 N1
pos_1284	TG(16:0/14:0/20:3)	pos	829.7280	C53 H97 O6
pos_1285	TG(16:1/16:1/18:1)	pos	851.7099	C53 H96 O6 Na1
pos_1287	TG(16:1e/16:1/18:1)	pos	832.7753	C53 H102 O5 N1
pos_1288	TG(16:0/14:0/20:4)	pos	849.6943	C53 H94 O6 Na1
pos_1289	TG(16:0/16:1/18:3)	pos	827.7123	C53 H95 O6
pos_1290	TG(18:1/14:2/18:1)	pos	942.8358	C59 H110 O6 N2
pos_1291	TG(16:0/12:0/22:4)	pos	942.8358	C59 H110 O6 N2
pos_1292	TG(16:1/14:0/20:4)	pos	847.6786	C53 H92 O6 Na1
pos_1294	TG(18:4/16:0/16:1)	pos	825.6967	C53 H93 O6
pos_1296	TG(18:4/16:1/16:1)	pos	845.6630	C53 H90 O6 Na1
pos_1297	TG(16:1/14:0/20:5)	pos	845.6630	C53 H90 O6 Na1
pos_1298	TG(16:1/14:1/20:5)	pos	843.6473	C53 H88 O6 Na1
pos_1299	TG(16:1/12:0/22:6)	pos	843.6473	C53 H88 O6 Na1
pos_1300	TG(14:0/14:1/22:6)	pos	838.6919	C53 H92 O6 N1
pos_1301	TG(10:0/18:1/22:6)	pos	936.7889	C59 H104 O6 N2

pos_1302	TG(12:0/18:3/20:5)	pos	836.6763	C53 H90 O6 N1
pos_1303	TG(16:0/14:3/20:5)	pos	836.6763	C53 H90 O6 N1
pos_1306	TG(16:0/12:3/22:6)	pos	932.7576	C59 H100 O6 N2
pos_1308	TG(18:0/16:0/17:0)	pos	866.8171	C54 H108 O6 N1
pos_1309	TG(16:0/17:0/18:1)	pos	864.8015	C54 H106 O6 N1
pos_1310	TG(16:0/11:1/24:0)	pos	962.8984	C60 H118 O6 N2
pos_1313	TG(16:0/17:1/18:1)	pos	867.7412	C54 H100 O6 Na1
pos_1315	TG(16:1/17:1/18:1)	pos	865.7256	C54 H98 O6 Na1
pos_1316	TG(18:1/11:1/22:1)	pos	958.8671	C60 H114 O6 N2
pos_1317	TG(15:0/16:0/20:4)	pos	841.7280	C54 H97 O6
pos_1318	TG(16:0/13:0/22:4)	pos	956.8515	C60 H112 O6 N2
pos_1319	TG(16:1/17:1/18:3)	pos	845.7205	C54 H94 O6 Li1
pos_1320	TG(15:0/14:0/22:5)	pos	839.7123	C54 H95 O6
pos_1321	TG(15:0/16:0/20:5)	pos	861.6943	C54 H94 O6 Na1
pos_1322	TG(18:1/13:0/20:4)	pos	954.8358	C60 H110 O6 N2
pos_1324	TG(14:0/17:1/20:5)	pos	859.6786	C54 H92 O6 Na1
pos_1325	TG(15:0/14:0/22:6)	pos	859.6786	C54 H92 O6 Na1
pos_1326	TG(11:0/18:1/22:5)	pos	952.8202	C60 H108 O6 N2
pos_1328	TG(20:5/14:1/17:1)	pos	852.7076	C54 H94 O6 N1
pos_1329	TG(18:0/11:1/22:6)	pos	950.8045	C60 H106 O6 N2
pos_1330	TG(20:5/14:2/17:1)	pos	850.6919	C54 H92 O6 N1
pos_1331	TG(18:3/13:0/20:5)	pos	850.6919	C54 H92 O6 N1
pos_1333	TG(18:0/16:0/18:0)	pos	880.8328	C55 H110 O6 N1

pos_1334	TG(18:0e/16:0/18:0)	pos	866.8535	C55 H112 O5 N1
pos_1336	TG(18:4/14:1/20:5)	pos	865.6317	C55 H86 O6 Na1
pos_1337	TG(16:0/14:4/22:6)	pos	860.6763	C55 H90 O6 N1
pos_1339	TG(18:0e/16:0/18:1)	pos	847.8113	C55 H107 O5
pos_1340	TG(16:0/18:1/18:1)	pos	876.8015	C55 H106 O6 N1
pos_1342	TG(16:0e/18:1/18:1)	pos	845.7957	C55 H105 O5
pos_1343	TG(18:1e/16:0/18:1)	pos	862.8222	C55 H108 O5 N1
pos_1344	TG(16:0/14:0/22:3)	pos	857.7593	C55 H101 O6
pos_1345	TG(16:1/18:1/18:1)	pos	879.7412	C55 H100 O6 Na1
pos_1346	TG(18:1e/16:1/18:1)	pos	860.8066	C55 H106 O5 N1
pos_1348	TG(16:1e/18:1/18:1)	pos	860.8066	C55 H106 O5 N1
pos_1349	TG(16:1/18:1/18:2)	pos	855.7436	C55 H99 O6
pos_1350	TG(16:0/16:0/20:4)	pos	877.7256	C55 H98 O6 Na1
pos_1351	TG(16:0/18:1/18:3)	pos	855.7436	C55 H99 O6
pos_1352	TG(16:0e/16:0/20:4)	pos	858.7909	C55 H104 O5 N1
pos_1353	TG(16:0/16:1/20:4)	pos	875.7099	C55 H96 O6 Na1
pos_1354	TG(16:0/16:0/20:5)	pos	875.7099	C55 H96 O6 Na1
pos_1355	TG(16:0e/14:0/22:5)	pos	856.7753	C55 H102 O5 N1
pos_1358	TG(16:1/14:0/22:5)	pos	868.7389	C55 H98 O6 N1
pos_1359	TG(16:1/16:1/20:4)	pos	873.6943	C55 H94 O6 Na1
pos_1362	TG(16:1/14:1/22:6)	pos	869.6630	C55 H90 O6 Na1
pos_1365	TG(18:4/16:1/18:4)	pos	862.6919	C55 H92 O6 N1
pos_1367	TG(18:0/17:0/18:0)	pos	894.8484	C56 H112 O6 N1

pos_1368	TG(18:0/16:0/19:0)	pos	894.8484	C56 H112 O6 N1
pos_1369	TG(16:0/18:1/19:0)	pos	881.8144	C56 H106 O6 Li1
pos_1370	TG(18:0/17:0/18:1)	pos	892.8328	C56 H110 O6 N1
pos_1371	TG(20:5/13:0/20:5)	pos	879.6473	C56 H88 O6 Na1
pos_1374	TG(20:4/11:2/22:6)	pos	968.7576	C62 H100 O6 N2
pos_1375	TG(20:5/11:3/22:5)	pos	966.7419	C62 H98 O6 N2
pos_1377	TG(17:0/18:1/18:1)	pos	890.8171	C56 H108 O6 N1
pos_1381	TG(18:1/17:1/18:3)	pos	873.7518	C56 H98 O6 Li1
pos_1383	TG(18:2/17:1/18:2)	pos	873.7518	C56 H98 O6 Li1
pos_1384	TG(18:1/13:0/22:4)	pos	982.8671	C62 H114 O6 N2
pos_1385	TG(18:4/17:1/18:1)	pos	871.7361	C56 H96 O6 Li1
pos_1386	TG(16:1/17:0/20:5)	pos	871.7361	C56 H96 O6 Li1
pos_1388	TG(14:0/17:1/22:5)	pos	882.7545	C56 H100 O6 N1
pos_1389	TG(16:0/17:1/20:5)	pos	887.7099	C56 H96 O6 Na1
pos_1390	TG(15:0/16:0/22:6)	pos	887.7099	C56 H96 O6 Na1
pos_1391	TG(18:1/13:0/22:5)	pos	980.8515	C62 H112 O6 N2
pos_1392	TG(16:1/17:1/20:5)	pos	869.7205	C56 H94 O6 Li1
pos_1393	TG(14:0/17:1/22:6)	pos	885.6943	C56 H94 O6 Na1
pos_1394	TG(18:4/17:1/18:3)	pos	878.7232	C56 H96 O6 N1
pos_1395	TG(22:6/14:1/17:1)	pos	878.7232	C56 H96 O6 N1
pos_1396	TG(18:4/15:0/20:5)	pos	881.6630	C56 H90 O6 Na1
pos_1398	TG(11:0/20:4/22:5)	pos	974.8045	C62 H106 O6 N2
pos_1399	TG(18:0/16:0/20:0)	pos	913.8195	C57 H110 O6 Na1

pos_1401	TG(18:0/18:0/18:1)	pos	906.8484	C57 H112 O6 N1
pos_1402	TG(18:0/16:0/20:1)	pos	906.8484	C57 H112 O6 N1
pos_1404	TG(18:4/14:0/22:6)	pos	893.6630	C57 H90 O6 Na1
pos_1405	TG(12:0/20:5/22:6)	pos	891.6473	C57 H88 O6 Na1
pos_1407	TG(10:0/22:6/22:6)	pos	889.6317	C57 H86 O6 Na1
pos_1409	TG(20:5/12:2/22:6)	pos	865.6341	C57 H85 O6
pos_1410	TG(18:0e/18:0/18:1)	pos	875.8426	C57 H111 O5
pos_1411	TG(16:0/18:1/20:1)	pos	887.8062	C57 H107 O6
pos_1412	TG(16:0e/18:1/20:1)	pos	890.8535	C57 H112 O5 N1
pos_1413	TG(18:1e/18:0/18:1)	pos	890.8535	C57 H112 O5 N1
pos_1414	TG(18:1/18:1/18:1)	pos	907.7725	C57 H104 O6 Na1
pos_1415	TG(18:1e/18:1/18:1)	pos	888.8379	C57 H110 O5 N1
pos_1417	TG(16:0/16:0/22:4)	pos	921.7308	C57 H102 O6 K1
pos_1419	TG(16:0/18:1/20:4)	pos	903.7412	C57 H100 O6 Na1
pos_1420	TG(16:0/16:1/22:4)	pos	887.7674	C57 H100 O6 Li1
pos_1422	TG(18:0/16:0/20:5)	pos	903.7412	C57 H100 O6 Na1
pos_1423	TG(16:0/16:0/22:5)	pos	898.7858	C57 H104 O6 N1
pos_1424	TG(16:0e/16:0/22:5)	pos	884.8066	C57 H106 O5 N1
pos_1426	TG(16:0/16:0/22:6)	pos	896.7702	C57 H102 O6 N1
pos_1428	TG(16:0/16:1/22:5)	pos	879.7436	C57 H99 O6
pos_1429	TG(16:1/18:1/20:4)	pos	901.7256	C57 H98 O6 Na1
pos_1432	TG(16:0/18:3/20:4)	pos	883.7361	C57 H96 O6 Li1
pos_1433	TG(16:1/16:1/22:5)	pos	877.7280	C57 H97 O6

pos_1435	TG(16:1/18:2/20:5)	pos	875.7123	C57 H95 O6
pos_1437	TG(16:1/18:3/20:5)	pos	895.6786	C57 H92 O6 Na1
pos_1438	TG(14:0/20:4/20:5)	pos	879.7048	C57 H92 O6 Li1
pos_1439	TG(16:2e/16:1/22:6)	pos	876.7440	C57 H98 O5 N1
pos_1440	TG(18:0/16:0/21:0)	pos	922.8797	C58 H116 O6 N1
pos_1441	TG(18:0/18:0/19:0)	pos	922.8797	C58 H116 O6 N1
pos_1442	TG(16:0/16:0/23:0)	pos	922.8797	C58 H116 O6 N1
pos_1443	TG(20:0/17:0/18:1)	pos	920.8641	C58 H114 O6 N1
pos_1444	TG(16:0/18:1/21:0)	pos	925.8195	C58 H110 O6 Na1
pos_1445	TG(18:0/16:0/21:1)	pos	920.8641	C58 H114 O6 N1
pos_1447	TG(18:4/15:0/22:6)	pos	891.7048	C58 H92 O6 Li1
pos_1449	TG(22:4/11:2/22:4)	pos	1000.8202	C64 H108 O6 N2
pos_1450	TG(20:5/13:0/22:6)	pos	889.6892	C58 H90 O6 Li1
pos_1452	TG(19:0/18:1/18:1)	pos	918.8484	C58 H112 O6 N1
pos_1453	TG(17:0/18:1/20:1)	pos	918.8484	C58 H112 O6 N1
pos_1454	TG(19:1/18:0/18:1)	pos	918.8484	C58 H112 O6 N1
pos_1457	TG(19:1/18:1/18:1)	pos	921.7882	C58 H106 O6 Na1
pos_1458	TG(16:0/17:0/22:4)	pos	914.8171	C58 H108 O6 N1
pos_1459	TG(16:0/17:0/22:5)	pos	912.8015	C58 H106 O6 N1
pos_1460	TG(19:1/18:2/18:2)	pos	901.7831	C58 H102 O6 Li1
pos_1461	TG(17:0/18:1/20:4)	pos	895.7749	C58 H103 O6
pos_1462	TG(16:1/17:1/22:4)	pos	910.7858	C58 H104 O6 N1
pos_1463	TG(16:0/17:1/22:5)	pos	910.7858	C58 H104 O6 N1

pos_1464	TG(16:0/17:0/22:6)	pos	893.7593	C58 H101 O6
pos_1465	TG(16:0/17:1/22:6)	pos	891.7436	C58 H99 O6
pos_1466	TG(19:1/14:0/22:6)	pos	897.7518	C58 H98 O6 Li1
pos_1467	TG(16:1/17:1/22:5)	pos	908.7702	C58 H102 O6 N1
pos_1469	TG(16:1/17:1/22:6)	pos	895.7361	C58 H96 O6 Li1
pos_1470	TG(18:3/17:1/20:4)	pos	889.7280	C58 H97 O6
pos_1471	TG(18:3/17:1/20:5)	pos	904.7389	C58 H98 O6 N1
pos_1472	TG(15:0/18:3/22:6)	pos	909.6943	C58 H94 O6 Na1
pos_1473	TG(11:0/22:4/22:5)	pos	1002.8358	C64 H110 O6 N2
pos_1474	TG(16:0/16:0/24:0)	pos	936.8954	C59 H118 O6 N1
pos_1476	TG(18:0/16:0/22:1)	pos	923.8613	C59 H112 O6 Li1
pos_1477	TG(16:0/16:1/24:0)	pos	939.8351	C59 H112 O6 Na1
pos_1478	TG(16:0/16:0/24:1)	pos	934.8797	C59 H116 O6 N1
pos_1479	TG(16:0/20:5/20:5)	pos	899.7123	C59 H95 O6
pos_1482	TG(14:0/20:4/22:6)	pos	899.7123	C59 H95 O6
pos_1483	TG(18:3e/16:1/22:6)	pos	902.7596	C59 H100 O5 N1
pos_1484	TG(18:4/16:1/22:6)	pos	903.7048	C59 H92 O6 Li1
pos_1486	TG(14:0/20:5/22:6)	pos	919.6786	C59 H92 O6 Na1
pos_1487	TG(20:5/14:1/22:6)	pos	917.6630	C59 H90 O6 Na1
pos_1488	TG(12:0/22:6/22:6)	pos	917.6630	C59 H90 O6 Na1
pos_1491	TG(20:5/14:3/22:6)	pos	908.6763	C59 H90 O6 N1
pos_1492	TG(22:5/12:3/22:6)	pos	891.6497	C59 H87 O6
pos_1494	TG(22:6/12:4/22:6)	pos	887.6184	C59 H83 O6

pos_1495	TG(18:0e/16:0/22:1)	pos	903.8739	C59 H115 O5
pos_1496	TG(20:0/18:1/18:1)	pos	932.8641	C59 H114 O6 N1
pos_1497	TG(16:0/18:1/22:1)	pos	915.8375	C59 H111 O6
pos_1498	TG(16:0/16:1/24:1)	pos	932.8641	C59 H114 O6 N1
pos_1499	TG(16:0e/18:1/22:1)	pos	918.8848	C59 H116 O5 N1
pos_1500	TG(20:1/18:1/18:1)	pos	935.8038	C59 H108 O6 Na1
pos_1503	TG(18:0/16:0/22:4)	pos	911.8062	C59 H107 O6
pos_1504	TG(18:0e/16:0/22:4)	pos	914.8535	C59 H112 O5 N1
pos_1505	TG(16:1/18:1/22:3)	pos	947.7464	C59 H104 O6 K1
pos_1506	TG(16:0/18:1/22:4)	pos	909.7906	C59 H105 O6
pos_1507	TG(18:0/16:0/22:5)	pos	926.8171	C59 H108 O6 N1
pos_1508	TG(18:0e/16:0/22:5)	pos	912.8379	C59 H110 O5 N1
pos_1509	TG(16:0/18:1/22:5)	pos	907.7749	C59 H103 O6
pos_1510	TG(18:0/16:0/22:6)	pos	929.7569	C59 H102 O6 Na1
pos_1512	TG(18:0e/16:0/22:6)	pos	910.8222	C59 H108 O5 N1
pos_1515	TG(18:1/18:1/20:5)	pos	927.7412	C59 H100 O6 Na1
pos_1518	TG(18:1/18:2/20:5)	pos	920.7702	C59 H102 O6 N1
pos_1519	TG(16:1/20:4/20:4)	pos	901.7280	C59 H97 O6
pos_1520	TG(16:0/20:4/20:5)	pos	923.7099	C59 H96 O6 Na1
pos_1522	TG(20:5/18:2/18:2)	pos	923.7099	C59 H96 O6 Na1
pos_1523	TG(18:3/18:2/20:4)	pos	918.7545	C59 H100 O6 N1
pos_1524	TG(18:0/19:0/20:0)	pos	950.9110	C60 H120 O6 N1
pos_1525	TG(18:0/16:0/23:0)	pos	950.9110	C60 H120 O6 N1

pos_1526	TG(16:0/17:0/24:1)	pos	948.8954	C60 H118 O6 N1
pos_1527	TG(16:0/18:1/23:0)	pos	953.8508	C60 H114 O6 Na1
pos_1529	TG(15:0/20:4/22:6)	pos	935.7099	C60 H96 O6 Na1
pos_1530	TG(20:5/17:1/20:5)	pos	928.7389	C60 H98 O6 N1
pos_1531	TG(18:4/17:1/22:6)	pos	933.6943	C60 H94 O6 Na1
pos_1534	TG(16:0e/18:1/23:0)	pos	934.9161	C60 H120 O5 N1
pos_1535	TG(16:0/18:1/23:1)	pos	951.8351	C60 H112 O6 Na1
pos_1536	TG(18:1/17:1/22:1)	pos	944.8641	C60 H114 O6 N1
pos_1537	TG(24:1/11:1/22:1)	pos	1042.9610	C66 H126 O6 N2
pos_1539	TG(16:0/19:0/22:4)	pos	942.8484	C60 H112 O6 N1
pos_1540	TG(17:0/18:1/22:4)	pos	940.8328	C60 H110 O6 N1
pos_1541	TG(18:0/17:0/22:5)	pos	940.8328	C60 H110 O6 N1
pos_1542	TG(17:0/18:1/22:5)	pos	938.8171	C60 H108 O6 N1
pos_1543	TG(18:0/17:0/22:6)	pos	921.7906	C60 H105 O6
pos_1544	TG(16:1/20:5/21:1)	pos	919.7749	C60 H103 O6
pos_1545	TG(19:1/16:1/22:5)	pos	936.8015	C60 H106 O6 N1
pos_1546	TG(18:1/17:1/22:5)	pos	936.8015	C60 H106 O6 N1
pos_1547	TG(17:0/18:1/22:6)	pos	941.7569	C60 H102 O6 Na1
pos_1548	TG(18:1/17:1/22:6)	pos	939.7412	C60 H100 O6 Na1
pos_1550	TG(18:3/17:1/22:5)	pos	932.7702	C60 H102 O6 N1
pos_1551	TG(18:2/17:1/22:6)	pos	937.7256	C60 H98 O6 Na1
pos_1552	TG(17:0/18:3/22:6)	pos	921.7518	C60 H98 O6 Li1
pos_1554	TG(16:0/18:1/24:0)	pos	962.9110	C61 H120 O6 N1

pos_1557	TG(18:1/18:3/22:6)	pos	949.7256	C61 H98 O6 Na1
pos_1558	TG(18:1/20:4/20:5)	pos	933.7518	C61 H98 O6 Li1
pos_1560	TG(16:0/20:5/22:6)	pos	925.7280	C61 H97 O6
pos_1561	TG(16:1/20:5/22:5)	pos	942.7545	C61 H100 O6 N1
pos_1562	TG(18:1/20:5/20:5)	pos	925.7280	C61 H97 O6
pos_1563	TG(14:0/22:6/22:6)	pos	923.7123	C61 H95 O6
pos_1564	TG(16:1/20:5/22:6)	pos	929.7205	C61 H94 O6 Li1
pos_1565	TG(22:6/14:1/22:6)	pos	943.6786	C61 H92 O6 Na1
pos_1566	TG(18:3e/20:5/20:5)	pos	924.7440	C61 H98 O5 N1
pos_1567	TG(22:6/14:2/22:6)	pos	936.7076	C61 H94 O6 N1
pos_1568	TG(22:6/14:3/22:6)	pos	934.6919	C61 H92 O6 N1
pos_1569	TG(16:0e/18:1/24:0)	pos	948.9318	C61 H122 O5 N1
pos_1572	TG(16:0/18:1/24:1)	pos	965.8508	C61 H114 O6 Na1
pos_1573	TG(16:1e/18:0/24:1)	pos	946.9161	C61 H120 O5 N1
pos_1574	TG(16:1/18:1/24:1)	pos	958.8797	C61 H116 O6 N1
pos_1576	TG(16:1e/18:1/24:1)	pos	944.9005	C61 H118 O5 N1
pos_1579	TG(16:0/20:4/22:0)	pos	956.8641	C61 H114 O6 N1
pos_1581	TG(18:0/18:1/22:4)	pos	937.8219	C61 H109 O6
pos_1584	TG(18:0/18:0/22:6)	pos	957.7882	C61 H106 O6 Na1
pos_1585	TG(20:1e/16:0/22:5)	pos	938.8535	C61 H112 O5 N1
pos_1586	TG(18:1/18:1/22:5)	pos	955.7725	C61 H104 O6 Na1
pos_1587	TG(18:0/18:1/22:6)	pos	950.8171	C61 H108 O6 N1
pos_1589	TG(18:1/18:1/22:6)	pos	931.7749	C61 H103 O6

pos_1590	TG(18:1/18:2/22:6)	pos	929.7593	C61 H101 O6
pos_1591	TG(16:1/20:4/22:4)	pos	929.7593	C61 H101 O6
pos_1592	TG(18:1/18:3/22:5)	pos	929.7593	C61 H101 O6
pos_1593	TG(16:0/20:3/22:6)	pos	929.7593	C61 H101 O6
pos_1594	TG(18:0/18:0/23:0)	pos	978.9423	C62 H124 O6 N1
pos_1595	TG(25:0/16:0/18:0)	pos	978.9423	C62 H124 O6 N1
pos_1596	TG(26:0/9:0/24:1)	pos	976.9267	C62 H122 O6 N1
pos_1597	TG(18:0/18:1/23:0)	pos	976.9267	C62 H122 O6 N1
pos_1598	TG(17:0/20:4/22:6)	pos	958.7858	C62 H104 O6 N1
pos_1599	TG(20:5/17:1/22:5)	pos	956.7702	C62 H102 O6 N1
pos_1600	TG(20:4/17:1/22:6)	pos	961.7256	C62 H98 O6 Na1
pos_1601	TG(15:0/22:5/22:6)	pos	961.7256	C62 H98 O6 Na1
pos_1602	TG(17:0/20:5/22:6)	pos	956.7702	C62 H102 O6 N1
pos_1603	TG(15:0/22:6/22:6)	pos	943.7361	C62 H96 O6 Li1
pos_1604	TG(20:5/17:1/22:6)	pos	959.7099	C62 H96 O6 Na1
pos_1605	TG(18:0e/18:1/23:0)	pos	962.9474	C62 H124 O5 N1
pos_1606	TG(18:1/18:1/23:0)	pos	979.8664	C62 H116 O6 Na1
pos_1607	TG(18:0e/18:1/23:1)	pos	960.9318	C62 H122 O5 N1
pos_1608	TG(18:1/17:1/24:1)	pos	972.8954	C62 H118 O6 N1
pos_1610	TG(16:0/21:0/22:4)	pos	970.8797	C62 H116 O6 N1
pos_1612	TG(16:0/21:0/22:5)	pos	968.8641	C62 H114 O6 N1
pos_1613	TG(16:0/20:5/23:0)	pos	968.8641	C62 H114 O6 N1
pos_1614	TG(19:1/18:1/22:4)	pos	966.8484	C62 H112 O6 N1

pos_1615	TG(19:0/18:1/22:5)	pos	966.8484	C62 H112 O6 N1
pos_1616	TG(16:0/21:0/22:6)	pos	966.8484	C62 H112 O6 N1
pos_1618	TG(19:1/18:1/22:5)	pos	964.8328	C62 H110 O6 N1
pos_1619	TG(19:0/18:1/22:6)	pos	964.8328	C62 H110 O6 N1
pos_1620	TG(19:1/18:1/22:6)	pos	962.8171	C62 H108 O6 N1
pos_1621	TG(20:2/17:1/22:6)	pos	960.8015	C62 H106 O6 N1
pos_1622	TG(20:0/16:0/24:0)	pos	992.9580	C63 H126 O6 N1
pos_1623	TG(18:0/18:1/24:0)	pos	990.9423	C63 H124 O6 N1
pos_1625	TG(18:1/20:3/22:6)	pos	955.7749	C63 H103 O6
pos_1627	TG(16:0/22:4/22:6)	pos	955.7749	C63 H103 O6
pos_1628	TG(18:0/20:4/22:6)	pos	972.8015	C63 H106 O6 N1
pos_1629	TG(18:1/20:4/22:5)	pos	955.7749	C63 H103 O6
pos_1630	TG(18:1/20:4/22:6)	pos	975.7412	C63 H100 O6 Na1
pos_1631	TG(16:0/22:5/22:6)	pos	953.7593	C63 H101 O6
pos_1632	TG(18:0/20:5/22:6)	pos	959.7674	C63 H100 O6 Li1
pos_1633	TG(16:0/22:6/22:6)	pos	951.7436	C63 H99 O6
pos_1634	TG(16:1/22:5/22:6)	pos	973.7256	C63 H98 O6 Na1
pos_1635	TG(18:1/20:5/22:6)	pos	973.7256	C63 H98 O6 Na1
pos_1636	TG(16:1/22:6/22:6)	pos	949.7280	C63 H97 O6
pos_1637	TG(18:3/20:4/22:6)	pos	971.7099	C63 H96 O6 Na1
pos_1638	TG(18:3/20:5/22:5)	pos	971.7099	C63 H96 O6 Na1
pos_1639	TG(20:5/18:2/22:6)	pos	971.7099	C63 H96 O6 Na1
pos_1640	TG(18:3/20:5/22:6)	pos	947.7123	C63 H95 O6

pos_1641	TG(16:2e/22:6/22:6)	pos	950.7596	C63 H100 O5 N1
pos_1642	TG(18:4/20:5/22:6)	pos	967.6786	C63 H92 O6 Na1
pos_1643	TG(20:5/20:5/20:5)	pos	951.7048	C63 H92 O6 Li1
pos_1644	TG(18:0e/18:1/24:0)	pos	976.9631	C63 H126 O5 N1
pos_1645	TG(18:1/18:1/24:0)	pos	993.8821	C63 H118 O6 Na1
pos_1646	TG(26:1/16:0/18:1)	pos	988.9267	C63 H122 O6 N1
pos_1647	TG(18:0e/18:1/24:1)	pos	974.9474	C63 H124 O5 N1
pos_1648	TG(18:1/18:1/24:1)	pos	991.8664	C63 H116 O6 Na1
pos_1649	TG(18:1/18:2/24:0)	pos	991.8664	C63 H116 O6 Na1
pos_1650	TG(24:0/12:2/24:1)	pos	969.8845	C63 H117 O6
pos_1652	TG(18:1/18:2/24:1)	pos	984.8954	C63 H118 O6 N1
pos_1653	TG(16:0/20:4/24:0)	pos	984.8954	C63 H118 O6 N1
pos_1654	TG(16:0/20:4/24:1)	pos	987.8351	C63 H112 O6 Na1
pos_1655	TG(16:0/20:5/24:0)	pos	987.8351	C63 H112 O6 Na1
pos_1656	TG(20:4e/16:0/24:1)	pos	951.8739	C63 H115 O5
pos_1658	TG(16:0/20:5/24:1)	pos	985.8195	C63 H110 O6 Na1
pos_1659	TG(16:0/22:0/22:6)	pos	963.8375	C63 H111 O6
pos_1660	TG(20:1/18:1/22:5)	pos	961.8219	C63 H109 O6
pos_1661	TG(16:0/22:1/22:6)	pos	961.8219	C63 H109 O6
pos_1662	TG(20:1/18:1/22:6)	pos	981.7882	C63 H106 O6 Na1
pos_1663	TG(18:0/20:3/22:6)	pos	974.8171	C63 H108 O6 N1
pos_1664	TG(18:0/20:5/22:4)	pos	957.7906	C63 H105 O6
pos_1665	TG(18:0/19:0/24:1)	pos	1004.9580	C64 H126 O6 N1

pos_1666	TG(16:0/22:1/23:0)	pos	1009.9134	C64 H122 O6 Na1
pos_1667	TG(19:0/20:4/22:6)	pos	986.8171	C64 H108 O6 N1
pos_1668	TG(17:0/22:5/22:6)	pos	984.8015	C64 H106 O6 N1
pos_1669	TG(22:5/17:1/22:6)	pos	987.7412	C64 H100 O6 Na1
pos_1670	TG(17:0/22:6/22:6)	pos	987.7412	C64 H100 O6 Na1
pos_1671	TG(22:6/17:1/22:6)	pos	980.7702	C64 H102 O6 N1
pos_1672	TG(25:0/18:1/18:1)	pos	1007.8977	C64 H120 O6 Na1
pos_1673	TG(19:1/18:1/24:1)	pos	1000.9267	C64 H122 O6 N1
pos_1674	TG(24:1/17:1/20:2)	pos	998.9110	C64 H120 O6 N1
pos_1677	TG(18:1/20:4/23:0)	pos	979.8688	C64 H115 O6
pos_1679	TG(16:0/22:5/23:1)	pos	994.8797	C64 H116 O6 N1
pos_1680	TG(16:0/22:6/23:0)	pos	977.8532	C64 H113 O6
pos_1681	TG(18:1/21:1/22:5)	pos	992.8641	C64 H114 O6 N1
pos_1682	TG(22:1/17:1/22:5)	pos	992.8641	C64 H114 O6 N1
pos_1683	TG(16:0/22:6/23:1)	pos	992.8641	C64 H114 O6 N1
pos_1684	TG(19:1/20:1/22:6)	pos	990.8484	C64 H112 O6 N1
pos_1685	TG(22:1/17:1/22:6)	pos	990.8484	C64 H112 O6 N1
pos_1686	TG(22:4/17:1/22:4)	pos	988.8328	C64 H110 O6 N1
pos_1687	TG(17:0/22:4/22:5)	pos	988.8328	C64 H110 O6 N1
pos_1688	TG(19:1/20:2/22:6)	pos	971.8062	C64 H107 O6
pos_1689	TG(16:0/22:1/24:0)	pos	1018.9736	C65 H128 O6 N1
pos_1690	TG(18:1/22:4/22:5)	pos	1000.8328	C65 H110 O6 N1
pos_1692	TG(18:0/22:4/22:6)	pos	1000.8328	C65 H110 O6 N1

pos_1694	TG(18:0/22:5/22:6)	pos	998.8171	C65 H108 O6 N1
pos_1695	TG(18:1/22:5/22:5)	pos	981.7906	C65 H105 O6
pos_1696	TG(18:1/22:5/22:6)	pos	1001.7569	C65 H102 O6 Na1
pos_1697	TG(18:0/22:6/22:6)	pos	979.7749	C65 H103 O6
pos_1698	TG(22:5/18:2/22:6)	pos	994.7858	C65 H104 O6 N1
pos_1699	TG(18:1/22:6/22:6)	pos	999.7412	C65 H100 O6 Na1
pos_1700	TG(18:3/22:5/22:6)	pos	992.7702	C65 H102 O6 N1
pos_1701	TG(18:2/22:6/22:6)	pos	997.7256	C65 H98 O6 Na1
pos_1702	TG(20:5/20:4/22:6)	pos	973.7280	C65 H97 O6
pos_1704	TG(18:3e/22:6/22:6)	pos	976.7753	C65 H102 O5 N1
pos_1705	TG(20:5/20:5/22:6)	pos	971.7123	C65 H95 O6
pos_1707	TG(16:0/22:1/24:1)	pos	1016.9580	C65 H126 O6 N1
pos_1708	TG(16:0e/22:1/24:1)	pos	1002.9787	C65 H128 O5 N1
pos_1709	TG(18:1/22:1/22:1)	pos	1019.8977	C65 H120 O6 Na1
pos_1710	TG(16:1e/22:1/24:1)	pos	1000.9631	C65 H126 O5 N1
pos_1711	TG(16:0/22:4/24:0)	pos	1012.9267	C65 H122 O6 N1
pos_1712	TG(16:0/22:4/24:1)	pos	1010.9110	C65 H120 O6 N1
pos_1713	TG(16:0/22:5/24:0)	pos	1015.8664	C65 H116 O6 Na1
pos_1714	TG(16:0/22:5/24:1)	pos	991.8688	C65 H115 O6
pos_1715	TG(16:0/22:6/24:0)	pos	991.8688	C65 H115 O6
pos_1716	TG(18:1/22:1/22:5)	pos	989.8532	C65 H113 O6
pos_1717	TG(16:0/22:6/24:1)	pos	1011.8351	C65 H112 O6 Na1
pos_1719	TG(18:1/22:2/22:6)	pos	1002.8484	C65 H112 O6 N1

pos_1720	TG(20:0/20:3/22:6)	pos	1002.8484	C65 H112 O6 N1
pos_1721	TG(20:1/20:2/22:6)	pos	985.8219	C65 H109 O6
pos_1722	TG(16:0/23:0/24:1)	pos	1032.9893	C66 H130 O6 N1
pos_1723	TG(19:0/22:4/22:6)	pos	1014.8484	C66 H112 O6 N1
pos_1724	TG(19:0/22:5/22:6)	pos	1012.8328	C66 H110 O6 N1
pos_1727	TG(19:1/22:6/22:6)	pos	1008.8015	C66 H106 O6 N1
pos_1728	TG(15:0/24:1/24:1)	pos	1019.9552	C66 H124 O6 Li1
pos_1729	TG(17:0/22:1/24:1)	pos	1030.9736	C66 H128 O6 N1
pos_1730	TG(24:1/17:1/22:1)	pos	1028.9580	C66 H126 O6 N1
pos_1731	TG(18:1/22:1/23:1)	pos	1033.9134	C66 H122 O6 Na1
pos_1732	TG(24:1/17:1/22:2)	pos	1026.9423	C66 H124 O6 N1
pos_1734	TG(27:1/12:2/24:1)	pos	1009.9158	C66 H121 O6
pos_1735	TG(17:0/22:4/24:1)	pos	1024.9267	C66 H122 O6 N1
pos_1736	TG(25:0/16:0/22:5)	pos	1024.9267	C66 H122 O6 N1
pos_1737	TG(24:1/17:1/22:4)	pos	1022.9110	C66 H120 O6 N1
pos_1738	TG(18:1/22:5/23:0)	pos	1022.9110	C66 H120 O6 N1
pos_1739	TG(18:0/22:6/23:0)	pos	1022.9110	C66 H120 O6 N1
pos_1740	TG(24:0/17:1/22:5)	pos	1005.8845	C66 H117 O6
pos_1742	TG(18:1/22:6/23:0)	pos	1020.8954	C66 H118 O6 N1
pos_1744	TG(24:1/17:1/22:6)	pos	1018.8797	C66 H116 O6 N1
pos_1745	TG(24:2/17:1/22:6)	pos	1016.8641	C66 H114 O6 N1
pos_1746	TG(20:2/21:1/22:6)	pos	999.8375	C66 H111 O6
pos_1748	TG(22:1/20:4/22:5)	pos	1028.8641	C67 H114 O6 N1

pos_1749	TG(20:0/22:4/22:6)	pos	1028.8641	C67 H114 O6 N1
pos_1751	TG(20:1/22:4/22:6)	pos	1026.8484	C67 H112 O6 N1
pos_1752	TG(20:0/22:5/22:6)	pos	1026.8484	C67 H112 O6 N1
pos_1753	TG(20:1/22:5/22:6)	pos	1024.8328	C67 H110 O6 N1
pos_1754	TG(20:0/22:6/22:6)	pos	1024.8328	C67 H110 O6 N1
pos_1755	TG(20:2/22:5/22:6)	pos	1022.8171	C67 H108 O6 N1
pos_1756	TG(20:1/22:6/22:6)	pos	1022.8171	C67 H108 O6 N1
pos_1757	TG(20:3/22:5/22:6)	pos	1020.8015	C67 H106 O6 N1
pos_1758	TG(20:2/22:6/22:6)	pos	1025.7569	C67 H102 O6 Na1
pos_1760	TG(20:3/22:6/22:6)	pos	1001.7593	C67 H101 O6
pos_1761	TG(20:5/22:5/22:6)	pos	999.7436	C67 H99 O6
pos_1762	TG(20:4/22:6/22:6)	pos	1016.7702	C67 H102 O6 N1
pos_1764	TG(18:0/22:1/24:1)	pos	1044.9893	C67 H130 O6 N1
pos_1765	TG(18:1/22:1/24:1)	pos	1047.9290	C67 H124 O6 Na1
pos_1766	TG(16:1e/24:1/24:1)	pos	1028.9944	C67 H130 O5 N1
pos_1767	TG(18:0/22:4/24:0)	pos	1040.9580	C67 H126 O6 N1
pos_1768	TG(18:0/22:4/24:1)	pos	1038.9423	C67 H124 O6 N1
pos_1769	TG(18:0/22:5/24:0)	pos	1038.9423	C67 H124 O6 N1
pos_1770	TG(18:1/22:4/24:1)	pos	1036.9267	C67 H122 O6 N1
pos_1771	TG(18:1/22:5/24:0)	pos	1041.8821	C67 H118 O6 Na1
pos_1772	TG(18:0/22:6/24:0)	pos	1036.9267	C67 H122 O6 N1
pos_1773	TG(18:1/22:5/24:1)	pos	1017.8845	C67 H117 O6
pos_1775	TG(18:1/22:6/24:0)	pos	1017.8845	C67 H117 O6

pos_1777	TG(24:1/18:2/22:5)	pos	1032.8954	C67 H118 O6 N1
pos_1778	TG(18:1/22:6/24:1)	pos	1037.8508	C67 H114 O6 Na1
pos_1779	TG(24:1/18:2/22:6)	pos	1030.8797	C67 H116 O6 N1
pos_1781	TG(20:1/22:2/22:6)	pos	1013.8532	C67 H113 O6
pos_1782	TG(20:2e/22:1/22:6)	pos	1005.8821	C67 H114 O5 Li1
pos_1784	TG(22:4/21:0/22:6)	pos	1042.8797	C68 H116 O6 N1
pos_1785	TG(20:4/22:6/23:0)	pos	1042.8797	C68 H116 O6 N1
pos_1788	TG(20:5/22:6/23:0)	pos	1040.8641	C68 H114 O6 N1
pos_1789	TG(20:5/22:6/23:1)	pos	1038.8484	C68 H112 O6 N1
pos_1790	TG(22:6/21:0/22:6)	pos	1038.8484	C68 H112 O6 N1
pos_1793	TG(18:1/23:0/24:1)	pos	1059.0049	C68 H132 O6 N1
pos_1794	TG(24:1/17:1/24:1)	pos	1061.9447	C68 H126 O6 Na1
pos_1795	TG(24:1/18:2/23:1)	pos	1054.9736	C68 H128 O6 N1
pos_1798	TG(26:0/17:0/22:5)	pos	1035.9314	C68 H123 O6
pos_1799	TG(25:0/18:0/22:5)	pos	1052.9580	C68 H126 O6 N1
pos_1800	TG(25:0/18:1/22:5)	pos	1050.9423	C68 H124 O6 N1
pos_1801	TG(25:1/18:1/22:5)	pos	1048.9267	C68 H122 O6 N1
pos_1802	TG(25:0/18:1/22:6)	pos	1048.9267	C68 H122 O6 N1
pos_1803	TG(19:1/22:6/24:1)	pos	1046.9110	C68 H120 O6 N1
pos_1804	TG(25:1/18:1/22:6)	pos	1046.9110	C68 H120 O6 N1
pos_1805	TG(22:4/20:4/23:1)	pos	1044.8954	C68 H118 O6 N1
pos_1806	TG(25:1/18:2/22:6)	pos	1044.8954	C68 H118 O6 N1
pos_1808	TG(24:1/20:4/22:5)	pos	1056.8954	C69 H118 O6 N1

pos_1809	TG(22:0/22:4/22:6)	pos	1056.8954	C69 H118 O6 N1
pos_1810	TG(24:0/20:4/22:6)	pos	1056.8954	C69 H118 O6 N1
pos_1811	TG(22:1/22:5/22:5)	pos	1054.8797	C69 H116 O6 N1
pos_1812	TG(22:0/22:5/22:6)	pos	1054.8797	C69 H116 O6 N1
pos_1813	TG(22:1/22:5/22:6)	pos	1052.8641	C69 H114 O6 N1
pos_1814	TG(22:0/22:6/22:6)	pos	1052.8641	C69 H114 O6 N1
pos_1815	TG(22:2/22:5/22:6)	pos	1050.8484	C69 H112 O6 N1
pos_1816	TG(22:1/22:6/22:6)	pos	1050.8484	C69 H112 O6 N1
pos_1817	TG(22:4/22:4/22:6)	pos	1048.8328	C69 H110 O6 N1
pos_1818	TG(22:2/22:6/22:6)	pos	1048.8328	C69 H110 O6 N1
pos_1819	TG(22:4/22:5/22:6)	pos	1046.8171	C69 H108 O6 N1
pos_1820	TG(22:5/22:5/22:6)	pos	1044.8015	C69 H106 O6 N1
pos_1821	TG(22:5/22:6/22:6)	pos	1042.7858	C69 H104 O6 N1
pos_1822	TG(22:6/22:6/22:6)	pos	1023.7436	C69 H99 O6
pos_1823	TG(18:1/24:0/24:1)	pos	1073.0206	C69 H134 O6 N1
pos_1824	TG(18:1/24:1/24:1)	pos	1071.0049	C69 H132 O6 N1
pos_1825	TG(18:1/24:1/24:2)	pos	1068.9893	C69 H130 O6 N1
pos_1826	TG(20:0/22:4/24:0)	pos	1068.9893	C69 H130 O6 N1
pos_1827	TG(20:1/22:4/24:0)	pos	1066.9736	C69 H128 O6 N1
pos_1829	TG(22:1/22:1/22:4)	pos	1064.9580	C69 H126 O6 N1
pos_1830	TG(26:1/18:0/22:5)	pos	1064.9580	C69 H126 O6 N1
pos_1831	TG(22:0/22:0/22:6)	pos	1064.9580	C69 H126 O6 N1
pos_1832	TG(22:1/22:1/22:5)	pos	1062.9423	C69 H124 O6 N1

pos_1833	TG(26:1/18:1/22:5)	pos	1062.9423	C69 H124 O6 N1
pos_1835	TG(20:1/22:5/24:1)	pos	1045.9158	C69 H121 O6
pos_1836	TG(24:1/20:2/22:5)	pos	1060.9267	C69 H122 O6 N1
pos_1837	TG(26:1/18:1/22:6)	pos	1065.8821	C69 H118 O6 Na1
pos_1838	TG(22:1/22:1/22:6)	pos	1043.9001	C69 H119 O6
pos_1839	TG(24:1/20:3/22:5)	pos	1058.9110	C69 H120 O6 N1
pos_1840	TG(24:1/20:2/22:6)	pos	1058.9110	C69 H120 O6 N1
pos_1841	TG(24:0/20:4/22:5)	pos	1058.9110	C69 H120 O6 N1
pos_1842	TG(24:0/20:5/22:4)	pos	1041.8845	C69 H117 O6
pos_1843	TG(22:1/22:2/22:6)	pos	1041.8845	C69 H117 O6
pos_1844	TG(22:4/22:6/23:0)	pos	1070.9110	C70 H120 O6 N1
pos_1845	TG(22:4/22:6/23:1)	pos	1068.8954	C70 H118 O6 N1
pos_1846	TG(22:5/22:6/23:0)	pos	1068.8954	C70 H118 O6 N1
pos_1847	TG(22:5/22:6/23:1)	pos	1066.8797	C70 H116 O6 N1
pos_1848	TG(22:6/22:6/23:0)	pos	1066.8797	C70 H116 O6 N1
pos_1849	TG(22:6/22:6/23:1)	pos	1064.8641	C70 H114 O6 N1
pos_1850	TG(25:0/18:1/24:1)	pos	1087.0362	C70 H136 O6 N1
pos_1851	TG(25:1/18:1/24:1)	pos	1085.0206	C70 H134 O6 N1
pos_1852	TG(24:1/20:2/23:1)	pos	1083.0049	C70 H132 O6 N1
pos_1855	TG(24:1/21:0/22:5)	pos	1078.9736	C70 H128 O6 N1
pos_1856	TG(22:0/22:6/23:0)	pos	1061.9471	C70 H125 O6
pos_1857	TG(24:0/21:0/22:6)	pos	1078.9736	C70 H128 O6 N1
pos_1858	TG(22:1/22:5/23:1)	pos	1076.9580	C70 H126 O6 N1

pos_1859	TG(22:1/22:6/23:0)	pos	1076.9580	C70 H126 O6 N1
pos_1860	TG(24:1/21:1/22:6)	pos	1074.9423	C70 H124 O6 N1
pos_1861	TG(22:1/22:6/23:1)	pos	1074.9423	C70 H124 O6 N1
pos_1862	TG(22:4/22:4/23:1)	pos	1072.9267	C70 H122 O6 N1
pos_1863	TG(24:1/22:4/22:5)	pos	1084.9267	C71 H122 O6 N1
pos_1864	TG(24:0/22:4/22:6)	pos	1084.9267	C71 H122 O6 N1
pos_1865	TG(24:1/22:4/22:6)	pos	1082.9110	C71 H120 O6 N1
pos_1867	TG(24:1/22:5/22:6)	pos	1080.8954	C71 H118 O6 N1
pos_1868	TG(24:0/22:6/22:6)	pos	1080.8954	C71 H118 O6 N1
pos_1869	TG(22:5/22:6/24:2)	pos	1078.8797	C71 H116 O6 N1
pos_1870	TG(24:1/22:6/22:6)	pos	1078.8797	C71 H116 O6 N1
pos_1872	TG(24:0/22:1/22:1)	pos	1101.0519	C71 H138 O6 N1
pos_1873	TG(24:1/22:1/22:1)	pos	1103.9916	C71 H132 O6 Na1
pos_1874	TG(24:1/22:1/22:2)	pos	1097.0206	C71 H134 O6 N1
pos_1875	TG(24:0/20:4/24:1)	pos	1095.0049	C71 H132 O6 N1
pos_1877	TG(24:1/22:1/22:4)	pos	1092.9893	C71 H130 O6 N1
pos_1879	TG(22:0/22:6/24:0)	pos	1075.9627	C71 H127 O6
pos_1880	TG(24:1/22:1/22:5)	pos	1090.9736	C71 H128 O6 N1
pos_1881	TG(22:0/22:6/24:1)	pos	1095.9290	C71 H124 O6 Na1
pos_1882	TG(24:1/22:1/22:6)	pos	1088.9580	C71 H126 O6 N1
pos_1883	TG(24:1/22:4/22:4)	pos	1086.9423	C71 H124 O6 N1
pos_1884	TG(24:1/22:2/22:6)	pos	1086.9423	C71 H124 O6 N1
pos_1885	TG(26:0/20:3/22:6)	pos	1069.9158	C71 H121 O6

pos_1886	TG(24:1/22:1/23:1)	pos	1113.0519	C72 H138 O6 N1
pos_1888	TG(24:1/22:5/23:0)	pos	1107.0049	C72 H132 O6 N1
pos_1889	TG(24:1/22:4/23:1)	pos	1089.9784	C72 H129 O6
pos_1890	TG(24:0/22:6/23:0)	pos	1107.0049	C72 H132 O6 N1
pos_1891	TG(24:1/22:5/23:1)	pos	1104.9893	C72 H130 O6 N1
pos_1892	TG(24:1/22:6/23:0)	pos	1104.9893	C72 H130 O6 N1
pos_1894	TG(26:1/22:4/22:5)	pos	1112.9580	C73 H126 O6 N1
pos_1896	TG(26:1/22:5/22:6)	pos	1108.9267	C73 H122 O6 N1
pos_1897	TG(26:0/22:6/22:6)	pos	1108.9267	C73 H122 O6 N1
pos_1898	TG(26:1/22:6/22:6)	pos	1106.9110	C73 H120 O6 N1
pos_1899	TG(24:1/22:1/24:1)	pos	1132.0229	C73 H136 O6 Na1
pos_1900	TG(24:1/22:2/24:1)	pos	1125.0519	C73 H138 O6 N1
pos_1904	TG(24:0/22:6/24:0)	pos	1121.0206	C73 H134 O6 N1
pos_1905	TG(24:1/22:5/24:1)	pos	1123.9603	C73 H128 O6 Na1
pos_1906	TG(24:0/22:6/24:1)	pos	1123.9603	C73 H128 O6 Na1
pos_1907	TG(24:1/22:5/24:2)	pos	1116.9893	C73 H130 O6 N1
pos_1908	TG(24:1/22:6/24:1)	pos	1121.9447	C73 H126 O6 Na1
pos_1909	TG(26:1/22:4/22:4)	pos	1114.9736	C73 H128 O6 N1
pos_1910	TG(24:1/22:6/24:2)	pos	1114.9736	C73 H128 O6 N1
pos_1912	TG(25:1/22:5/24:1)	pos	1133.0206	C74 H134 O6 N1
pos_1913	TG(25:0/22:6/24:1)	pos	1133.0206	C74 H134 O6 N1
pos_1914	TG(25:1/22:6/24:1)	pos	1131.0049	C74 H132 O6 N1
pos_1915	TG(28:0/22:5/22:5)	pos	1140.9893	C75 H130 O6 N1

pos_1917	TG(28:0/22:6/22:6)	pos	1136.9580	C75 H126 O6 N1
pos_1918	TG(28:1/22:6/22:6)	pos	1134.9423	C75 H124 O6 N1
pos_1920	TG(26:1/22:5/24:1)	pos	1147.0362	C75 H136 O6 N1
pos_1921	TG(26:0/22:6/24:1)	pos	1147.0362	C75 H136 O6 N1
pos_1922	TG(26:1/22:6/24:1)	pos	1145.0206	C75 H134 O6 N1
pos_1923	TG(26:1/22:6/24:2)	pos	1143.0049	C75 H132 O6 N1
pos_1925	TG(30:0/22:6/22:6)	pos	1164.9893	C77 H130 O6 N1
pos_1926	TG(30:0/22:1/22:6)	pos	1175.0675	C77 H140 O6 N1
pos_1927	TG(28:1/22:6/24:1)	pos	1173.0519	C77 H138 O6 N1
pos_1928	TG(30:1/22:6/24:1)	pos	1201.0832	C79 H142 O6 N1
pos_1929	WE(3:0/16:1)	pos	314.3054	H40 C19 O2 N1
pos_1930	WE(3:0/18:1)	pos	342.3367	H44 C21 O2 N1
pos_1931	WE(3:0/18:2)	pos	340.3210	H42 C21 O2 N1
pos_1932	WE(3:0/19:1)	pos	356.3523	H46 C22 O2 N1
pos_1933	WE(3:0/20:1)	pos	370.3680	H48 C23 O2 N1
pos_1934	WE(3:0/20:2)	pos	368.3523	H46 C23 O2 N1
pos_1935	ZyE(20:4)	pos	671.5762	C47 H75 O2
pos_1936	ZyE(20:5)	pos	669.5605	C47 H73 O2
pos_1937	ZyE(22:4)	pos	716.6340	C49 H82 O2 N1
pos_1938	ZyE(22:5)	pos	697.5918	C49 H77 O2
pos_1939	ZyE(22:6)	pos	695.5762	C49 H75 O2
pos_147	DG(16:0/18:1)	pos	617.5115	C37 H70 O5 Na1
neg_1	CL(14:0/16:0/16:0/18:0)	neg	675.4788	C73 H140 O17 P2

neg_2	CL(16:0/16:0/16:0/16:0)	neg	675.4788	C73 H140 O17 P2
neg_3	CL(14:0/16:0/16:1/18:1)	neg	673.4632	C73 H136 O17 P2
neg_4	CL(16:0/16:0/18:0/18:0)	neg	703.5101	C77 H148 O17 P2
neg_5	CL(16:0/16:0/16:0/20:0)	neg	703.5101	C77 H148 O17 P2
neg_6	CL(18:1/16:0/16:0/18:1)	neg	701.4945	C77 H144 O17 P2
neg_7	CL(18:1/16:1/16:1/18:1)	neg	699.4788	C77 H140 O17 P2
neg_8	CL(23:1/14:0/16:0/18:1)	neg	722.5180	C80 H150 O17 P2
neg_9	CL(24:0/14:0/16:0/18:0)	neg	731.5414	C81 H156 O17 P2
neg_10	CL(20:5/16:0/16:1/20:4)	neg	721.4632	C81 H136 O17 P2
neg_12	CL(14:0/18:0/20:4/20:4)	neg	723.4788	C81 H140 O17 P2
neg_14	CL(23:0/15:0/16:0/20:0)	neg	745.5571	C83 H160 O17 P2
neg_15	CL(24:0/15:0/16:0/20:4)	neg	748.5336	C84 H154 O17 P2
neg_16	CL(20:5/15:0/18:1/22:0)	neg	746.5180	C84 H150 O17 P2
neg_17	CL(21:0/16:0/16:0/22:6)	neg	746.5180	C84 H150 O17 P2
neg_18	CL(19:0/16:1/18:1/22:6)	neg	744.5023	C84 H146 O17 P2
neg_19	CL(24:0/16:0/16:0/20:0)	neg	759.5727	C85 H164 O17 P2
neg_20	CL(24:0/16:0/16:1/20:1)	neg	757.5571	C85 H160 O17 P2
neg_21	CL(23:1/14:0/16:0/24:1)	neg	764.5649	C86 H162 O17 P2
neg_22	CL(23:0/16:0/18:0/20:4)	neg	762.5493	C86 H158 O17 P2
neg_23	CL(23:0/16:0/16:0/22:6)	neg	760.5336	C86 H154 O17 P2
neg_24	CL(21:0/16:1/18:1/22:6)	neg	758.5180	C86 H150 O17 P2
neg_25	CL(22:4/16:0/16:1/24:1)	neg	767.5414	C87 H156 O17 P2
neg_26	CL(19:0/18:1/20:3/22:6)	neg	770.5180	C88 H150 O17 P2

neg_27	CL(21:0/22:4/16:0/20:0)	neg	776.5649	C88 H162 O17 P2
neg_28	CL(23:0/16:0/20:0/20:4)	neg	776.5649	C88 H162 O17 P2
neg_29	CL(22:6/15:0/20:0/22:0)	neg	774.5493	C88 H158 O17 P2
neg_30	CL(21:0/22:5/18:0/18:1)	neg	774.5493	C88 H158 O17 P2
neg_31	CL(23:0/16:0/18:0/22:6)	neg	1550.1058	C88 H159 O17 P2
neg_32	CL(21:0/18:0/18:0/22:6)	neg	774.5493	C88 H158 O17 P2
neg_33	CL(23:1/16:0/18:1/22:6)	neg	772.5336	C88 H154 O17 P2
neg_34	CL(21:0/16:0/22:5/22:6)	neg	1568.0589	C90 H153 O17 P2
neg_35	CL(23:0/18:1/18:1/22:3)	neg	789.5727	C90 H164 O17 P2
neg_36	CL(23:0/16:0/20:0/22:6)	neg	788.5649	C90 H162 O17 P2
neg_37	CL(20:5/16:0/22:4/24:1)	neg	791.5414	C91 H156 O17 P2
neg_38	CL(17:0/22:5/22:6/22:6)	neg	1583.9963	C92 H145 O17 P2
neg_39	CL(23:0/18:0/20:0/22:6)	neg	802.5806	C92 H166 O17 P2
neg_40	CL(23:0/16:0/22:2/22:6)	neg	800.5649	C92 H162 O17 P2
neg_41	CL(22:6/20:0/22:0/22:6)	neg	817.5571	C95 H160 O17 P2
neg_42	Cer(d18:0/16:0)	neg	584.5259	C35 H70 O5 N1
neg_43	Cer(d18:2/16:0)	neg	580.4946	C35 H66 O5 N1
neg_44	Cer(d16:2/19:1)	neg	592.4946	C36 H66 O5 N1
neg_46	Cer(d16:1/21:0)	neg	624.5572	C38 H74 O5 N1
neg_47	Cer(d16:2/21:1)	neg	620.5259	C38 H70 O5 N1
neg_49	Cer(d16:1/22:0+O)	neg	608.5623	C38 H74 O4 N1
neg_50	Cer(d16:2/23:1)	neg	648.5572	C40 H74 O5 N1
neg_51	Cer(d18:0/22:0)	neg	668.6198	C41 H82 O5 N1

neg_52	Cer(d16:1/24:0)	neg	620.5987	C40 H78 O3 N1
neg_54	Cer(d19:1/22:0+O)	neg	650.6093	C41 H80 O4 N1
neg_56	Cer(d19:2/22:1)	neg	676.5885	C42 H78 O5 N1
neg_57	Cer(d19:2/22:2)	neg	674.5729	C42 H76 O5 N1
neg_58	Cer(d18:0/24:1)	neg	694.6355	C43 H84 O5 N1
neg_59	Cer(d16:1/26:2)	neg	644.5987	C42 H78 O3 N1
neg_60	Cer(d16:2/26:2)	neg	688.5885	C43 H78 O5 N1
neg_61	Cer(d19:1/24:0)	neg	708.6511	C44 H86 O5 N1
neg_62	Cer(d19:1/24:1)	neg	706.6355	C44 H84 O5 N1
neg_63	Cer(d17:1/26:2)	neg	704.6198	C44 H82 O5 N1
neg_64	Cer(d19:2/24:2)	neg	702.6042	C44 H80 O5 N1
neg_65	Cer(m18:0/18:1+O)	neg	610.5416	C37 H72 O5 N1
neg_66	Cer(t16:1/24:1)	neg	680.5835	C41 H78 O6 N1
neg_67	Cer(t18:0/24:1)	neg	710.6304	C43 H84 O6 N1
neg_68	Cer(t16:0/26:2)	neg	662.6093	C42 H80 O4 N1
neg_69	CerG2GNAc1(d18:2/18:1)	neg	1087.6898	C56 H99 O18 N2
neg_70	CerG2GNAc1(d20:2/17:0)	neg	551.3569	C57 H102 O18 N2
neg_71	FA(18:4)	neg	275.2017	O2 H27 C18
neg_72	FA(20:4)	neg	303.2330	O2 H31 C20
neg_74	FA(22:4)	neg	331.2643	O2 H35 C22
neg_75	FA(22:5)	neg	329.2486	O2 H33 C22
neg_76	FA(22:6)	neg	327.2330	O2 H31 C22
neg_77	GD1a(m19:1/20:4)	neg	926.4735	C87 H144 O38 N4

neg_78	GD2(d19:0/22:6)	neg	866.4524	C83 H136 O34 N4
neg_79	GM3(m18:1/18:1)	neg	580.3597	C59 H104 O20 N2
neg_80	GM3(m17:1/19:1)	neg	580.3597	C59 H104 O20 N2
neg_81	GM3(m22:0/24:0)	neg	652.4536	C69 H128 O20 N2
neg_82	Hex1Cer(d17:0/16:1)	neg	730.5475	C40 H76 O10 N1
neg_83	Hex1Cer(d16:0/18:1)	neg	744.5631	C41 H78 O10 N1
neg_84	Hex1Cer(d21:0/17:1)	neg	800.6257	C45 H86 O10 N1
neg_85	Hex1Cer(d20:1/18:1)	neg	798.6101	C45 H84 O10 N1
neg_86	Hex1Cer(d21:0/18:1)	neg	814.6414	C46 H88 O10 N1
neg_87	Hex1Cer(d21:0/22:4)	neg	864.6570	C50 H90 O10 N1
neg_88	Hex1Cer(t20:1/18:1)	neg	814.6050	C45 H84 O11 N1
neg_89	Hex1Cer(t16:0/22:6)	neg	806.5424	C45 H76 O11 N1
neg_90	Hex1Cer(t17:0/22:6)	neg	774.5526	C45 H76 O9 N1
neg_91	Hex1Cer(t18:0/22:6)	neg	834.5737	C47 H80 O11 N1
neg_92	Hex2Cer(d12:0/16:0)	neg	778.5322	C40 H76 O13 N1
neg_93	Hex2Cer(d22:1/18:1)	neg	988.6942	C53 H98 O15 N1
neg_94	Hex2Cer(d24:1/18:1)	neg	1016.7255	C55 H102 O15 N1
neg_95	Hex2Cer(d24:0/22:6)	neg	1078.7411	C60 H104 O15 N1
neg_96	Hex3Cer(m21:1/22:6)	neg	1120.7153	C61 H102 O17 N1
neg_97	LPE(15:0)	neg	438.2626	C20 H41 O7 N1 P1
neg_98	LPE(17:0)	neg	466.2939	C22 H45 O7 N1 P1
neg_99	LPE(17:1)	neg	464.2783	C22 H43 O7 N1 P1
neg_100	LPE(18:2)	neg	476.2783	C23 H43 O7 N1 P1

neg_101	LPE(19:0)	neg	494.3252	C24 H49 O7 N1 P1
neg_102	LPE(20:0)	neg	508.3409	C25 H51 O7 N1 P1
neg_103	LPE(20:1)	neg	506.3252	C25 H49 O7 N1 P1
neg_104	LPE(20:3)	neg	502.2939	C25 H45 O7 N1 P1
neg_105	LPE(20:5)	neg	498.2626	C25 H41 O7 N1 P1
neg_106	LPE(22:4)	neg	528.3096	C27 H47 O7 N1 P1
neg_107	LPEt(18:1)	neg	463.2830	C23 H44 O7 N0 P1
neg_108	LPEt(20:4)	neg	485.2674	C25 H42 O7 N0 P1
neg_109	LPEt(22:6)	neg	509.2674	C27 H42 O7 N0 P1
neg_110	LPG(15:0)	neg	469.2572	C21 H42 O9 N0 P1
neg_111	LPG(16:0)	neg	483.2728	C22 H44 O9 N0 P1
neg_112	LPG(16:1)	neg	481.2572	C22 H42 O9 N0 P1
neg_113	LPG(20:4)	neg	531.2728	C26 H44 O9 N0 P1
neg_114	LPG(20:5)	neg	529.2572	C26 H42 O9 N0 P1
neg_115	LPG(22:6)	neg	555.2728	C28 H44 O9 N0 P1
neg_116	LPI(16:0)	neg	571.2889	C25 H48 O12 N0 P1
neg_117	LPI(18:0)	neg	599.3202	C27 H52 O12 N0 P1
neg_118	LPI(18:1)	neg	597.3045	C27 H50 O12 N0 P1
neg_119	LPI(20:1)	neg	625.3358	C29 H54 O12 N0 P1
neg_122	LPI(20:4)	neg	619.2889	C29 H48 O12 N0 P1
neg_123	LPI(20:5)	neg	617.2732	C29 H46 O12 N0 P1
neg_124	LPI(22:5)	neg	645.3045	C31 H50 O12 N0 P1
neg_125	LPI(22:6)	neg	643.2889	C31 H48 O12 N0 P1

neg_126	LPS(20:4)	neg	544.2681	C26 H43 O9 N1 P1
neg_127	LPS(22:4)	neg	572.2994	C28 H47 O9 N1 P1
neg_128	LPS(22:5)	neg	570.2837	C28 H45 O9 N1 P1
neg_129	LdMePE(14:0)	neg	452.2783	C21 H43 O7 N1 P1
neg_130	LdMePE(14:0e)	neg	438.2990	C21 H45 O6 N1 P1
neg_131	LdMePE(15:0)	neg	466.2939	C22 H45 O7 N1 P1
neg_132	LdMePE(16:0)	neg	480.3096	C23 H47 O7 N1 P1
neg_133	LdMePE(16:0e)	neg	466.3303	C23 H49 O6 N1 P1
neg_134	LdMePE(16:1)	neg	478.2939	C23 H45 O7 N1 P1
neg_135	LdMePE(16:1e)	neg	464.3147	C23 H47 O6 N1 P1
neg_136	LdMePE(17:0)	neg	494.3252	C24 H49 O7 N1 P1
neg_137	LdMePE(17:1)	neg	492.3096	C24 H47 O7 N1 P1
neg_138	LdMePE(18:0)	neg	508.3409	C25 H51 O7 N1 P1
neg_139	LdMePE(18:0e)	neg	494.3616	C25 H53 O6 N1 P1
neg_140	LdMePE(18:1e)	neg	492.3460	C25 H51 O6 N1 P1
neg_141	LdMePE(18:2)	neg	504.3096	C25 H47 O7 N1 P1
neg_142	LdMePE(18:3)	neg	502.2939	C25 H45 O7 N1 P1
neg_143	LdMePE(19:0)	neg	522.3565	C26 H53 O7 N1 P1
neg_144	LdMePE(19:1)	neg	520.3409	C26 H51 O7 N1 P1
neg_145	LdMePE(20:0)	neg	536.3722	C27 H55 O7 N1 P1
neg_146	LdMePE(20:0e)	neg	522.3929	C27 H57 O6 N1 P1
neg_147	LdMePE(20:1)	neg	534.3565	C27 H53 O7 N1 P1
neg_148	LdMePE(20:2)	neg	532.3409	C27 H51 O7 N1 P1

neg_149	LdMePE(20:3)	neg	530.3252	C27 H49 O7 N1 P1
neg_150	LdMePE(20:4)	neg	528.3096	C27 H47 O7 N1 P1
neg_151	LdMePE(20:5)	neg	526.2939	C27 H45 O7 N1 P1
neg_152	LdMePE(22:4)	neg	556.3409	C29 H51 O7 N1 P1
neg_153	LdMePE(22:5)	neg	554.3252	C29 H49 O7 N1 P1
neg_154	LdMePE(22:6)	neg	552.3096	C29 H47 O7 N1 P1
neg_155	LdMePE(24:1)	neg	590.4191	C31 H61 O7 N1 P1
neg_156	MGDG(12:0e/15:0)	neg	705.5158	C38 H73 O11
neg_157	MGDG(12:0e/16:0)	neg	719.5315	C39 H75 O11
neg_158	MGDG(14:0e/15:0)	neg	733.5471	C40 H77 O11
neg_159	MGDG(14:0e/16:0)	neg	747.5628	C41 H79 O11
neg_160	MGDG(14:0e/16:1)	neg	745.5471	C41 H77 O11
neg_161	MGDG(16:0e/15:0)	neg	761.5784	C42 H81 O11
neg_162	MGDG(12:0e/20:0)	neg	775.5941	C43 H83 O11
neg_164	MGDG(14:1e/18:1)	neg	771.5628	C43 H79 O11
neg_165	MGDG(16:0e/18:0)	neg	803.6254	C45 H87 O11
neg_166	MGDG(16:0e/18:1)	neg	801.6097	C45 H85 O11
neg_167	MGDG(18:1/16:0p)	neg	799.5941	C45 H83 O11
neg_168	MGDG(18:1e/16:1)	neg	799.5941	C45 H83 O11
neg_169	MGDG(18:0e/17:1)	neg	815.6254	C46 H87 O11
neg_170	MGDG(18:0e/18:1)	neg	815.6254	C46 H87 O11
neg_171	MGDG(12:0e/24:1)	neg	829.6410	C47 H89 O11
neg_172	MGDG(18:1e/18:1)	neg	827.6254	C47 H87 O11

neg_173	MGDG(20:3e/16:0)	neg	825.6097	C47 H85 O11
neg_174	MGDG(20:0e/17:1)	neg	843.6567	C48 H91 O11
neg_175	MGDG(20:2e/17:1)	neg	839.6254	C48 H87 O11
neg_176	MGDG(16:0/21:5)	neg	849.5734	C48 H81 O12
neg_177	MGDG(20:1e/18:0)	neg	857.6723	C49 H93 O11
neg_178	MGDG(20:1e/18:1)	neg	855.6567	C49 H91 O11
neg_179	MGDG(20:3e/18:0)	neg	853.6410	C49 H89 O11
neg_180	MGDG(20:4e/18:1)	neg	789.5886	C47 H81 O9
neg_182	MGMG(28:0)	neg	719.5315	C39 H75 O11
neg_183	MLCL(14:2/14:0/14:0)	neg	512.3014	C51 H94 O16 P2
neg_184	MLCL(14:2/15:0/15:0)	neg	526.3171	C53 H98 O16 P2
neg_185	MLCL(10:4/16:1/18:1)	neg	522.2858	C53 H90 O16 P2
neg_186	MLCL(10:1/15:1/20:3)	neg	530.3014	C54 H94 O16 P2
neg_187	MLCL(14:2/16:0/16:0)	neg	540.3327	C55 H102 O16 P2
neg_188	MLCL(14:2/16:1/16:1)	neg	538.3171	C55 H98 O16 P2
neg_190	MLCL(11:2/16:1/22:4)	neg	556.3171	C58 H98 O16 P2
neg_191	MLCL(14:2/18:0/18:0)	neg	568.3640	C59 H110 O16 P2
neg_192	MLCL(14:2/18:1/18:1)	neg	566.3484	C59 H106 O16 P2
neg_193	MLCL(14:2/18:2/18:2)	neg	564.3327	C59 H102 O16 P2
neg_194	MLCL(18:2/16:1/18:1)	neg	580.3640	C61 H110 O16 P2
neg_195	MLCL(11:3/20:4/22:6)	neg	578.3014	C62 H94 O16 P2
neg_196	MLCL(14:2/20:4/20:4)	neg	588.3327	C63 H102 O16 P2
neg_197	MLCL(14:2/20:0/20:0)	neg	596.3953	C63 H118 O16 P2

neg_198	MLCL(14:2/20:1/20:1)	neg	594.3797	C63 H114 O16 P2
neg_199	MLCL(20:2/14:0/20:4)	neg	592.3640	C63 H110 O16 P2
neg_200	MLCL(14:2/20:3/20:3)	neg	590.3484	C63 H106 O16 P2
neg_201	MLCL(14:2/22:4/22:4)	neg	616.3640	C67 H110 O16 P2
neg_202	MLCL(14:2/22:5/22:5)	neg	614.3484	C67 H106 O16 P2
neg_203	MLCL(14:2/22:6/22:6)	neg	612.3327	C67 H102 O16 P2
neg_204	MLCL(14:2/22:0/22:0)	neg	624.4266	C67 H126 O16 P2
neg_206	MLCL(18:4/20:4/22:6)	neg	626.3484	C69 H106 O16 P2
neg_207	MLCL(24:0/18:1/20:1)	neg	652.4579	C71 H134 O16 P2
neg_208	MLCL(14:2/24:1/24:1)	neg	650.4423	C71 H130 O16 P2
neg_209	OAHFA(18:1/18:0)	neg	563.5045	C36 H67 O4
neg_210	OAHFA(18:1/20:3)	neg	585.4888	C38 H65 O4
neg_211	OAHFA(20:4/20:3)	neg	607.4732	C40 H63 O4
neg_212	OAHFA(20:4/22:6)	neg	629.4575	C42 H61 O4
neg_213	OAHFA(22:5/22:4)	neg	659.5045	C44 H67 O4
neg_214	PC(14:0e/14:0)	neg	708.5185	C37 H75 O9 N1 P1
neg_215	PC(14:1e/14:0)	neg	706.5028	C37 H73 O9 N1 P1
neg_216	PC(14:0e/15:0)	neg	722.5341	C38 H77 O9 N1 P1
neg_217	PC(14:1e/15:0)	neg	720.5185	C38 H75 O9 N1 P1
neg_218	PC(16:0e/14:0)	neg	736.5498	C39 H79 O9 N1 P1
neg_219	PC(16:1e/14:0)	neg	734.5341	C39 H77 O9 N1 P1
neg_220	PC(16:0e/15:0)	neg	750.5654	C40 H81 O9 N1 P1
neg_222	PC(16:0e/16:1)	neg	762.5654	C41 H81 O9 N1 P1

neg_224	PC(17:1/16:1)	neg	788.5447	C42 H79 O10 N1 P1
neg_225	PC(16:1e/17:1)	neg	774.5654	C42 H81 O9 N1 P1
neg_226	PC(16:0e/18:1)	neg	790.5967	C43 H85 O9 N1 P1
neg_227	PC(14:0/20:4)	neg	798.5291	C43 H77 O10 N1 P1
neg_228	PC(14:0e/20:5)	neg	782.5341	C43 H77 O9 N1 P1
neg_229	PC(17:0/18:1)	neg	818.5917	C44 H85 O10 N1 P1
neg_230	PC(17:1/18:1)	neg	816.5760	C44 H83 O10 N1 P1
neg_231	PC(18:2e/17:1)	neg	800.5811	C44 H83 O9 N1 P1
neg_232	PC(20:1e/16:0)	neg	818.6280	C45 H89 O9 N1 P1
neg_233	PC(18:1e/18:1)	neg	756.5913	C43 H83 O7 N1 P1
neg_234	PC(18:2e/18:1)	neg	814.5967	C45 H85 O9 N1 P1
neg_235	PC(14:0e/22:4)	neg	812.5811	C45 H83 O9 N1 P1
neg_236	PC(16:0e/20:5)	neg	810.5654	C45 H81 O9 N1 P1
neg_237	PC(16:1e/20:5)	neg	808.5498	C45 H79 O9 N1 P1
neg_238	PC(19:0/18:1)	neg	846.6230	C46 H89 O10 N1 P1
neg_239	PC(19:1/18:1)	neg	844.6073	C46 H87 O10 N1 P1
neg_241	PC(18:2e/20:4)	neg	826.5523	C46 H82 O7 N1 P1 Cl1
neg_242	PC(18:1e/20:5)	neg	836.5811	C47 H83 O9 N1 P1
neg_243	PC(16:1e/22:5)	neg	836.5811	C47 H83 O9 N1 P1
neg_244	PC(17:0/22:4)	neg	868.6073	C48 H87 O10 N1 P1
neg_245	PC(19:0/20:5)	neg	866.5917	C48 H85 O10 N1 P1
neg_246	PC(16:0/24:1)	neg	888.6699	C49 H95 O10 N1 P1
neg_247	PC(18:1/22:0)	neg	888.6699	C49 H95 O10 N1 P1

neg_248	PC(18:1/22:1)	neg	886.6543	C49 H93 O10 N1 P1
neg_249	PC(20:0/20:5)	neg	880.6073	C49 H87 O10 N1 P1
neg_250	PC(18:0e/22:5)	neg	866.6280	C49 H89 O9 N1 P1
neg_251	PC(18:1e/22:5)	neg	864.6124	C49 H87 O9 N1 P1
neg_252	PC(20:1/22:5)	neg	906.6230	C51 H89 O10 N1 P1
neg_253	PC(24:0/20:5)	neg	936.6699	C53 H95 O10 N1 P1
neg_255	PC(22:4/22:5)	neg	928.6073	C53 H87 O10 N1 P1
neg_256	PC(24:0/22:6)	neg	962.6856	C55 H97 O10 N1 P1
neg_257	PC(30:1/17:1)	neg	924.7427	C54 H103 O8 N1 P1
neg_258	PC(32:1/17:1)	neg	952.7740	C56 H107 O8 N1 P1
neg_259	PC(30:1/20:4)	neg	960.7427	C57 H103 O8 N1 P1
neg_260	PC(30:1/20:5)	neg	958.7270	C57 H101 O8 N1 P1
neg_261	PC(28:1/22:6)	neg	956.7114	C57 H99 O8 N1 P1
neg_262	PC(29:1/22:6)	neg	970.7270	C58 H101 O8 N1 P1
neg_263	PC(30:1/22:4)	neg	988.7740	C59 H107 O8 N1 P1
neg_264	PC(32:1/20:4)	neg	988.7740	C59 H107 O8 N1 P1
neg_265	PC(30:1/22:5)	neg	986.7583	C59 H105 O8 N1 P1
neg_266	PC(32:1/20:5)	neg	986.7583	C59 H105 O8 N1 P1
neg_267	PC(30:1/22:6)	neg	984.7427	C59 H103 O8 N1 P1
neg_268	PC(31:1/22:6)	neg	998.7583	C60 H105 O8 N1 P1
neg_269	PC(32:1/22:4)	neg	1016.8053	C61 H111 O8 N1 P1
neg_270	PC(34:1/20:4)	neg	1016.8053	C61 H111 O8 N1 P1
neg_271	PC(32:1/22:5)	neg	1014.7896	C61 H109 O8 N1 P1

neg_273	PC(32:1/22:6)	neg	1012.7740	C61 H107 O8 N1 P1
neg_274	PC(33:1/22:6)	neg	1026.7896	C62 H109 O8 N1 P1
neg_275	PC(34:1/22:6)	neg	1040.8053	C63 H111 O8 N1 P1
neg_278	PE(16:1/14:0)	neg	660.4610	C35 H67 O8 N1 P1
neg_280	PE(14:1e/17:1)	neg	658.4817	C36 H69 O7 N1 P1
neg_281	PE(16:0/16:0)	neg	690.5079	C37 H73 O8 N1 P1
neg_282	PE(16:1e/16:0)	neg	674.5130	C37 H73 O7 N1 P1
neg_283	PE(14:1e/18:1)	neg	672.4974	C37 H71 O7 N1 P1
neg_284	PE(16:1e/16:1)	neg	672.4974	C37 H71 O7 N1 P1
neg_285	PE(17:1/16:0)	neg	702.5079	C38 H73 O8 N1 P1
neg_287	PE(18:0/16:0)	neg	718.5392	C39 H77 O8 N1 P1
neg_288	PE(18:0e/16:1)	neg	702.5443	C39 H77 O7 N1 P1
neg_290	PE(16:0/18:2)	neg	714.5079	C39 H73 O8 N1 P1
neg_291	PE(18:1e/16:1)	neg	700.5287	C39 H75 O7 N1 P1
neg_292	PE(18:2e/16:1)	neg	698.5130	C39 H73 O7 N1 P1
neg_293	PE(14:0/20:4)	neg	710.4766	C39 H69 O8 N1 P1
neg_294	PE(14:1e/20:4)	neg	694.4817	C39 H69 O7 N1 P1
neg_295	PE(14:1e/20:5)	neg	692.4661	C39 H67 O7 N1 P1
neg_296	PE(17:0/18:1)	neg	730.5392	C40 H77 O8 N1 P1
neg_297	PE(17:1/18:1)	neg	728.5236	C40 H75 O8 N1 P1
neg_298	PE(18:2e/17:1)	neg	712.5287	C40 H75 O7 N1 P1
neg_299	PE(18:3e/17:1)	neg	710.5130	C40 H73 O7 N1 P1
neg_300	PE(16:0/20:1)	neg	744.5549	C41 H79 O8 N1 P1

neg_301	PE(18:0e/18:1)	neg	730.5756	C41 H81 O7 N1 P1
neg_302	PE(18:1/18:1)	neg	742.5392	C41 H77 O8 N1 P1
neg_303	PE(18:0/18:2)	neg	742.5392	C41 H77 O8 N1 P1
neg_304	PE(18:1/18:2)	neg	740.5236	C41 H75 O8 N1 P1
neg_305	PE(16:0/20:3)	neg	740.5236	C41 H75 O8 N1 P1
neg_306	PE(18:2e/18:1)	neg	726.5443	C41 H77 O7 N1 P1
neg_307	PE(18:1e/18:2)	neg	726.5443	C41 H77 O7 N1 P1
neg_308	PE(16:0e/20:4)	neg	724.5287	C41 H75 O7 N1 P1
neg_309	PE(16:1/20:4)	neg	736.4923	C41 H71 O8 N1 P1
neg_310	PE(14:0/22:6)	neg	734.4766	C41 H69 O8 N1 P1
neg_311	PE(16:2e/20:4)	neg	720.4974	C41 H71 O7 N1 P1
neg_312	PE(16:1e/20:5)	neg	720.4974	C41 H71 O7 N1 P1
neg_314	PE(19:0/18:1)	neg	758.5705	C42 H81 O8 N1 P1
neg_315	PE(20:1e/17:1)	neg	742.5756	C42 H81 O7 N1 P1
neg_316	PE(17:1/20:2)	neg	754.5392	C42 H77 O8 N1 P1
neg_317	PE(20:2e/17:1)	neg	740.5600	C42 H79 O7 N1 P1
neg_318	PE(17:0/20:4)	neg	752.5236	C42 H75 O8 N1 P1
neg_319	PE(17:0/20:5)	neg	750.5079	C42 H73 O8 N1 P1
neg_321	PE(15:0/22:6)	neg	748.4923	C42 H71 O8 N1 P1
neg_322	PE(18:0/20:1)	neg	772.5862	C43 H83 O8 N1 P1
neg_323	PE(18:1e/20:0)	neg	758.6069	C43 H85 O7 N1 P1
neg_324	PE(20:1e/18:1)	neg	756.5913	C43 H83 O7 N1 P1
neg_325	PE(18:0/20:3)	neg	768.5549	C43 H79 O8 N1 P1

neg_326	PE(20:2e/18:1)	neg	754.5756	C43 H81 O7 N1 P1
neg_327	PE(16:0/22:4)	neg	766.5392	C43 H77 O8 N1 P1
neg_328	PE(18:0e/20:4)	neg	752.5600	C43 H79 O7 N1 P1
neg_329	PE(18:1/20:4)	neg	764.5236	C43 H75 O8 N1 P1
neg_330	PE(16:0/22:5)	neg	764.5236	C43 H75 O8 N1 P1
neg_331	PE(16:1e/22:4)	neg	750.5443	C43 H77 O7 N1 P1
neg_332	PE(18:1e/20:4)	neg	750.5443	C43 H77 O7 N1 P1
neg_333	PE(16:1/22:5)	neg	762.5079	C43 H73 O8 N1 P1
neg_334	PE(18:1/20:5)	neg	762.5079	C43 H73 O8 N1 P1
neg_335	PE(18:1e/20:5)	neg	748.5287	C43 H75 O7 N1 P1
neg_336	PE(16:1e/22:5)	neg	748.5287	C43 H75 O7 N1 P1
neg_337	PE(18:2e/20:5)	neg	746.5130	C43 H73 O7 N1 P1
neg_339	PE(16:2e/22:6)	neg	744.4974	C43 H71 O7 N1 P1
neg_340	PE(17:1/22:2)	neg	782.5705	C44 H81 O8 N1 P1
neg_342	PE(19:0/20:4)	neg	780.5549	C44 H79 O8 N1 P1
neg_343	PE(17:0/22:6)	neg	776.5236	C44 H75 O8 N1 P1
neg_344	PE(17:1/22:6)	neg	774.5079	C44 H73 O8 N1 P1
neg_345	PE(18:4/22:6)	neg	782.4766	C45 H69 O8 N1 P1
neg_346	PE(18:1/22:2)	neg	796.5862	C45 H83 O8 N1 P1
neg_347	PE(18:1/22:3)	neg	794.5705	C45 H81 O8 N1 P1
neg_348	PE(18:0/22:4)	neg	794.5705	C45 H81 O8 N1 P1
neg_349	PE(20:0/20:4)	neg	794.5705	C45 H81 O8 N1 P1
neg_350	PE(18:0e/22:4)	neg	780.5913	C45 H83 O7 N1 P1

neg_351	PE(18:1e/22:4)	neg	778.5756	C45 H81 O7 N1 P1
neg_352	PE(18:1/22:5)	neg	790.5392	C45 H77 O8 N1 P1
neg_353	PE(18:0e/22:6)	neg	776.5600	C45 H79 O7 N1 P1
neg_354	PE(18:1e/22:5)	neg	776.5600	C45 H79 O7 N1 P1
neg_355	PE(18:2e/22:5)	neg	774.5443	C45 H77 O7 N1 P1
neg_356	PE(20:4/20:4)	neg	786.5079	C45 H73 O8 N1 P1
neg_357	PE(18:2e/22:6)	neg	772.5287	C45 H75 O7 N1 P1
neg_358	PE(18:3e/22:6)	neg	770.5130	C45 H73 O7 N1 P1
neg_359	PE(19:0/22:6)	neg	804.5549	C46 H79 O8 N1 P1
neg_360	PE(19:1/22:6)	neg	802.5392	C46 H77 O8 N1 P1
neg_361	PE(20:0/22:5)	neg	820.5862	C47 H83 O8 N1 P1
neg_362	PE(20:0/22:6)	neg	818.5705	C47 H81 O8 N1 P1
neg_363	PE(20:0e/22:6)	neg	804.5913	C47 H83 O7 N1 P1
neg_364	PE(20:1/22:6)	neg	816.5549	C47 H79 O8 N1 P1
neg_365	PE(20:1e/22:6)	neg	802.5756	C47 H81 O7 N1 P1
neg_366	PE(20:2/22:6)	neg	814.5392	C47 H77 O8 N1 P1
neg_367	PE(20:2e/22:6)	neg	800.5600	C47 H79 O7 N1 P1
neg_368	PE(22:5/20:4)	neg	812.5236	C47 H75 O8 N1 P1
neg_370	PEt(16:0/18:1)	neg	701.5127	C39 H74 O8 N0 P1
neg_371	PEt(12:0e/22:6)	neg	677.4552	C39 H66 O7 N0 P1
neg_372	PEt(18:0/18:1)	neg	729.5440	C41 H78 O8 N0 P1
neg_373	PEt(18:1/18:1)	neg	727.5283	C41 H76 O8 N0 P1
neg_374	PEt(16:0e/20:4)	neg	709.5178	C41 H74 O7 N0 P1

neg_375	PEt(16:0/20:5)	neg	721.4814	C41 H70 O8 N0 P1
neg_376	PEt(16:1e/20:4)	neg	707.5021	C41 H72 O7 N0 P1
neg_378	PEt(16:1/22:6)	neg	745.4814	C43 H70 O8 N0 P1
neg_380	PEt(18:1/22:6)	neg	773.5127	C45 H74 O8 N0 P1
neg_382	PEt(20:5/22:6)	neg	793.4814	C47 H70 O8 N0 P1
neg_383	PEt(22:6/22:6)	neg	819.4970	C49 H72 O8 N0 P1
neg_384	PEt(22:2/22:6)	neg	827.5596	C49 H80 O8 N0 P1
neg_385	PG(15:0/13:0)	neg	665.4399	C34 H66 O10 N0 P1
neg_386	PG(15:0/15:0)	neg	693.4712	C36 H70 O10 N0 P1
neg_387	PG(15:0/16:0)	neg	707.4869	C37 H72 O10 N0 P1
neg_388	PG(15:0/16:1)	neg	705.4712	C37 H70 O10 N0 P1
neg_389	PG(16:0/16:1)	neg	719.4869	C38 H72 O10 N0 P1
neg_390	PG(12:1e/20:4)	neg	697.4450	C38 H66 O9 N0 P1
neg_392	PG(17:1/16:1)	neg	731.4869	C39 H72 O10 N0 P1
neg_393	PG(16:0/18:1)	neg	747.5182	C40 H76 O10 N0 P1
neg_395	PG(17:1/18:1)	neg	759.5182	C41 H76 O10 N0 P1
neg_396	PG(18:0/18:1)	neg	775.5495	C42 H80 O10 N0 P1
neg_397	PG(18:1/18:1)	neg	773.5338	C42 H78 O10 N0 P1
neg_398	PG(18:1/18:2)	neg	771.5182	C42 H76 O10 N0 P1
neg_399	PG(16:0/20:4)	neg	769.5025	C42 H74 O10 N0 P1
neg_400	PG(16:0/20:5)	neg	767.4869	C42 H72 O10 N0 P1
neg_401	PG(16:1/20:4)	neg	767.4869	C42 H72 O10 N0 P1
neg_402	PG(16:1/20:5)	neg	765.4712	C42 H70 O10 N0 P1

neg_403	PG(14:0/22:6)	neg	765.4712	C42 H70 O10 N0 P1
neg_404	PG(16:2e/20:4)	neg	751.4919	C42 H72 O9 N0 P1
neg_406	PG(18:0/20:4)	neg	797.5338	C44 H78 O10 N0 P1
neg_407	PG(18:1/20:4)	neg	795.5182	C44 H76 O10 N0 P1
neg_408	PG(16:1/22:5)	neg	793.5025	C44 H74 O10 N0 P1
neg_409	PG(18:1/20:5)	neg	793.5025	C44 H74 O10 N0 P1
neg_410	PG(16:0/22:6)	neg	793.5025	C44 H74 O10 N0 P1
neg_411	PG(16:0e/22:6)	neg	779.5232	C44 H76 O9 N0 P1
neg_412	PG(16:1/22:6)	neg	791.4869	C44 H72 O10 N0 P1
neg_413	PG(16:1e/22:6)	neg	777.5076	C44 H74 O9 N0 P1
neg_414	PG(18:0/22:6)	neg	821.5338	C46 H78 O10 N0 P1
neg_416	PG(18:2e/22:6)	neg	803.5232	C46 H76 O9 N0 P1
neg_417	PG(20:4/22:6)	neg	841.5025	C48 H74 O10 N0 P1
neg_418	PG(20:5/22:6)	neg	839.4869	C48 H72 O10 N0 P1
neg_419	PG(22:5/22:5)	neg	869.5338	C50 H78 O10 N0 P1
neg_420	PG(22:5/22:6)	neg	867.5182	C50 H76 O10 N0 P1
neg_421	PG(22:6/22:6)	neg	865.5025	C50 H74 O10 N0 P1
neg_422	PG(28:1/20:5)	neg	933.6590	C54 H94 O10 N0 P1
neg_423	PI(16:0/16:1)	neg	807.5029	C41 H76 O13 N0 P1
neg_424	PI(16:0/18:1)	neg	835.5342	C43 H80 O13 N0 P1
neg_425	PI(16:1/18:1)	neg	833.5186	C43 H78 O13 N0 P1
neg_426	PI(14:0/20:4)	neg	829.4873	C43 H74 O13 N0 P1
neg_427	PI(18:0/18:1)	neg	863.5655	C45 H84 O13 N0 P1

neg_428	PI(18:1/18:1)	neg	861.5499	C45 H82 O13 N0 P1
neg_429	PI(16:0/20:4)	neg	857.5186	C45 H78 O13 N0 P1
neg_430	PI(16:1/20:4)	neg	855.5029	C45 H76 O13 N0 P1
neg_431	PI(16:0/20:5)	neg	855.5029	C45 H76 O13 N0 P1
neg_432	PI(16:1e/20:4)	neg	841.5236	C45 H78 O12 N0 P1
neg_433	PI(16:1/20:5)	neg	853.4873	C45 H74 O13 N0 P1
neg_434	PI(14:0/22:6)	neg	853.4873	C45 H74 O13 N0 P1
neg_435	PI(17:0/20:4)	neg	871.5342	C46 H80 O13 N0 P1
neg_436	PI(17:1/20:4)	neg	869.5186	C46 H78 O13 N0 P1
neg_437	PI(15:0/22:6)	neg	867.5029	C46 H76 O13 N0 P1
neg_438	PI(18:1/20:4)	neg	883.5342	C47 H80 O13 N0 P1
neg_439	PI(18:0/20:5)	neg	883.5342	C47 H80 O13 N0 P1
neg_440	PI(16:1/22:5)	neg	881.5186	C47 H78 O13 N0 P1
neg_442	PI(16:0e/22:6)	neg	867.5393	C47 H80 O12 N0 P1
neg_443	PI(19:0/20:4)	neg	899.5655	C48 H84 O13 N0 P1
neg_444	PI(17:0/22:6)	neg	895.5342	C48 H80 O13 N0 P1
neg_445	PI(17:1/22:6)	neg	893.5186	C48 H78 O13 N0 P1
neg_446	PI(18:0/22:4)	neg	913.5812	C49 H86 O13 N0 P1
neg_447	PI(18:0/22:5)	neg	911.5655	C49 H84 O13 N0 P1
neg_448	PI(18:1/22:5)	neg	909.5499	C49 H82 O13 N0 P1
neg_450	PI(18:3/22:6)	neg	903.5029	C49 H76 O13 N0 P1
neg_451	PI(19:0/22:6)	neg	923.5655	C50 H84 O13 N0 P1
neg_452	PI(20:5/22:6)	neg	927.5029	C51 H76 O13 N0 P1

neg_453	PI(22:5/20:4)	neg	931.5342	C51 H80 O13 N0 P1
neg_454	PI(22:4/22:6)	neg	957.5499	C53 H82 O13 N0 P1
neg_455	PI(22:5/22:6)	neg	955.5342	C53 H80 O13 N0 P1
neg_457	PIP2(20:4e/19:0)	neg	1045.5189	C48 H88 O18 N0 P3
neg_458	PMe(16:1/16:1)	neg	657.4501	C36 H66 O8 N0 P1
neg_459	PMe(16:1/18:1)	neg	685.4814	C38 H70 O8 N0 P1
neg_460	PS(16:0/18:1)	neg	760.5134	C40 H75 O10 N1 P1
neg_461	PS(16:1/18:2)	neg	756.4821	C40 H71 O10 N1 P1
neg_462	PS(16:1/18:3)	neg	754.4665	C40 H69 O10 N1 P1
neg_463	PS(18:3e/17:1)	neg	754.5028	C41 H73 O9 N1 P1
neg_464	PS(18:1/18:1)	neg	786.5291	C42 H77 O10 N1 P1
neg_465	PS(18:1/18:2)	neg	784.5134	C42 H75 O10 N1 P1
neg_466	PS(16:0/20:4)	neg	782.4978	C42 H73 O10 N1 P1
neg_467	PS(14:0/22:6)	neg	778.4665	C42 H69 O10 N1 P1
neg_468	PS(18:0/19:0)	neg	804.5760	C43 H83 O10 N1 P1
neg_469	PS(19:0/18:1)	neg	802.5604	C43 H81 O10 N1 P1
neg_470	PS(20:3e/17:1)	neg	782.5341	C43 H77 O9 N1 P1
neg_471	PS(20:4e/17:1)	neg	780.5185	C43 H75 O9 N1 P1
neg_472	PS(18:0/20:0)	neg	818.5917	C44 H85 O10 N1 P1
neg_474	PS(18:1/20:2)	neg	812.5447	C44 H79 O10 N1 P1
neg_475	PS(18:0/20:4)	neg	810.5291	C44 H77 O10 N1 P1
neg_476	PS(18:0e/20:4)	neg	796.5498	C44 H79 O9 N1 P1
neg_477	PS(20:3e/18:1)	neg	796.5498	C44 H79 O9 N1 P1

neg_478	PS(16:0/22:5)	neg	808.5134	C44 H75 O10 N1 P1
neg_479	PS(18:0/20:5)	neg	808.5134	C44 H75 O10 N1 P1
neg_480	PS(18:0e/20:5)	neg	794.5341	C44 H77 O9 N1 P1
neg_481	PS(18:2/20:4)	neg	806.4978	C44 H73 O10 N1 P1
neg_482	PS(20:5/18:2)	neg	804.4821	C44 H71 O10 N1 P1
neg_483	PS(18:3e/20:4)	neg	790.5028	C44 H73 O9 N1 P1
neg_484	PS(18:3/20:5)	neg	802.4665	C44 H69 O10 N1 P1
neg_485	PS(18:1/21:0)	neg	830.5917	C45 H85 O10 N1 P1
neg_486	PS(19:0/20:4)	neg	824.5447	C45 H79 O10 N1 P1
neg_487	PS(19:0/20:5)	neg	822.5291	C45 H77 O10 N1 P1
neg_488	PS(17:0/22:6)	neg	820.5134	C45 H75 O10 N1 P1
neg_490	PS(18:0/22:4)	neg	838.5604	C46 H81 O10 N1 P1
neg_491	PS(20:0e/20:4)	neg	824.5811	C46 H83 O9 N1 P1
neg_492	PS(20:0/20:5)	neg	836.5447	C46 H79 O10 N1 P1
neg_493	PS(18:1/22:4)	neg	836.5447	C46 H79 O10 N1 P1
neg_494	PS(20:2/20:4)	neg	834.5291	C46 H77 O10 N1 P1
neg_496	PS(18:3e/22:4)	neg	818.5341	C46 H77 O9 N1 P1
neg_497	PS(20:3e/20:4)	neg	818.5341	C46 H77 O9 N1 P1
neg_498	PS(18:2/22:6)	neg	830.4978	C46 H73 O10 N1 P1
neg_499	PS(18:3e/22:5)	neg	816.5185	C46 H75 O9 N1 P1
neg_500	PS(20:3e/20:5)	neg	816.5185	C46 H75 O9 N1 P1
neg_501	PS(18:3/22:6)	neg	828.4821	C46 H71 O10 N1 P1
neg_502	PS(18:3e/22:6)	neg	814.5028	C46 H73 O9 N1 P1

neg_503	PS(20:4e/20:5)	neg	814.5028	C46 H73 O9 N1 P1
neg_504	PS(18:1/23:0)	neg	858.6230	C47 H89 O10 N1 P1
neg_505	PS(20:5/21:0)	neg	850.5604	C47 H81 O10 N1 P1
neg_506	PS(19:0/22:6)	neg	848.5447	C47 H79 O10 N1 P1
neg_507	PS(20:4/22:6)	neg	854.4978	C48 H73 O10 N1 P1
neg_508	PS(20:4e/22:6)	neg	840.5185	C48 H75 O9 N1 P1
neg_509	PS(20:5/22:6)	neg	852.4821	C48 H71 O10 N1 P1
neg_510	PS(20:0/22:6)	neg	862.5604	C48 H81 O10 N1 P1
neg_511	PS(20:2/22:4)	neg	862.5604	C48 H81 O10 N1 P1
neg_512	PS(20:0e/22:6)	neg	848.5811	C48 H83 O9 N1 P1
neg_513	PS(20:1/22:6)	neg	860.5447	C48 H79 O10 N1 P1
neg_514	PS(20:2/22:5)	neg	860.5447	C48 H79 O10 N1 P1
neg_515	PS(20:3e/22:4)	neg	846.5654	C48 H81 O9 N1 P1
neg_517	PS(20:2/22:6)	neg	858.5291	C48 H77 O10 N1 P1
neg_518	PS(20:4e/22:4)	neg	844.5498	C48 H79 O9 N1 P1
neg_519	PS(20:3e/22:5)	neg	844.5498	C48 H79 O9 N1 P1
neg_520	PS(20:5/22:4)	neg	856.5134	C48 H75 O10 N1 P1
neg_521	PS(20:3/22:6)	neg	856.5134	C48 H75 O10 N1 P1
neg_522	PS(20:4e/22:5)	neg	842.5341	C48 H77 O9 N1 P1
neg_523	PS(20:3e/22:6)	neg	842.5341	C48 H77 O9 N1 P1
neg_524	PS(22:5/21:0)	neg	878.5917	C49 H85 O10 N1 P1
neg_525	PS(22:6/21:0)	neg	876.5760	C49 H83 O10 N1 P1
neg_526	PS(22:6/23:0)	neg	904.6073	C51 H87 O10 N1 P1

neg_527	PS(25:0/22:6)	neg	932.6386	C53 H91 O10 N1 P1
neg_528	PS(27:0/22:6)	neg	960.6699	C55 H95 O10 N1 P1
neg_529	PS(32:1/22:6)	neg	1028.7325	C60 H103 O10 N1 P1
neg_530	SM(d18:2/14:0)	neg	717.5188	C38 H74 O8 N2 P1
neg_531	SM(d16:1/19:0)	neg	761.5814	C41 H82 O8 N2 P1
neg_532	SM(d16:1/20:0)	neg	775.5971	C42 H84 O8 N2 P1
neg_533	SM(d16:1/22:1)	neg	801.6127	C44 H86 O8 N2 P1
neg_534	SM(d16:1/23:0)	neg	817.6440	C45 H90 O8 N2 P1
neg_535	SM(d18:1/21:1)	neg	815.6284	C45 H88 O8 N2 P1
neg_536	SM(d16:1/24:2)	neg	827.6284	C46 H88 O8 N2 P1
neg_537	SM(d17:1/24:1)	neg	843.6597	C47 H92 O8 N2 P1
neg_538	SM(d20:0/24:1)	neg	887.7223	C50 H100 O8 N2 P1
neg_539	cPA(22:6)	neg	463.2255	C25 H36 O6 N0 P1
neg_540	dMePE(14:0/14:0)	neg	662.4766	C35 H69 O8 N1 P1
neg_542	dMePE(16:0/14:0)	neg	690.5079	C37 H73 O8 N1 P1
neg_543	dMePE(16:0e/14:0)	neg	676.5287	C37 H75 O7 N1 P1
neg_544	dMePE(16:1/14:0)	neg	688.4923	C37 H71 O8 N1 P1
neg_545	dMePE(16:1e/14:0)	neg	674.5130	C37 H73 O7 N1 P1
neg_546	dMePE(16:1/14:1)	neg	686.4766	C37 H69 O8 N1 P1
neg_547	dMePE(15:0/16:0)	neg	704.5236	C38 H75 O8 N1 P1
neg_549	dMePE(16:0/16:0)	neg	718.5392	C39 H77 O8 N1 P1
neg_550	dMePE(16:0e/16:0)	neg	704.5600	C39 H79 O7 N1 P1
neg_551	dMePE(16:0/16:1)	neg	716.5236	C39 H75 O8 N1 P1

neg_552	dMePE(16:0e/16:1)	neg	702.5443	C39 H77 O7 N1 P1
neg_555	dMePE(16:1e/16:1)	neg	700.5287	C39 H75 O7 N1 P1
neg_556	dMePE(17:1/16:0)	neg	730.5392	C40 H77 O8 N1 P1
neg_557	dMePE(16:0/18:1)	neg	744.5549	C41 H79 O8 N1 P1
neg_558	dMePE(16:0e/18:1)	neg	730.5756	C41 H81 O7 N1 P1
neg_559	dMePE(16:1/18:1)	neg	742.5392	C41 H77 O8 N1 P1
neg_560	dMePE(16:0/18:3)	neg	740.5236	C41 H75 O8 N1 P1
neg_561	dMePE(14:0/20:4)	neg	738.5079	C41 H73 O8 N1 P1
neg_562	dMePE(14:0/20:5)	neg	736.4923	C41 H71 O8 N1 P1
neg_563	dMePE(17:0/18:1)	neg	758.5705	C42 H81 O8 N1 P1
neg_564	dMePE(17:1/18:1)	neg	756.5549	C42 H79 O8 N1 P1
neg_565	dMePE(18:0/18:1)	neg	772.5862	C43 H83 O8 N1 P1
neg_566	dMePE(16:1e/20:0)	neg	758.6069	C43 H85 O7 N1 P1
neg_567	dMePE(18:1/18:1)	neg	770.5705	C43 H81 O8 N1 P1
neg_568	dMePE(18:0/18:2)	neg	770.5705	C43 H81 O8 N1 P1
neg_570	dMePE(16:0/20:4)	neg	766.5392	C43 H77 O8 N1 P1
neg_571	dMePE(16:0e/20:4)	neg	752.5600	C43 H79 O7 N1 P1
neg_572	dMePE(16:1/20:4)	neg	764.5236	C43 H75 O8 N1 P1
neg_573	dMePE(16:0/20:5)	neg	764.5236	C43 H75 O8 N1 P1
neg_574	dMePE(16:0e/20:5)	neg	750.5443	C43 H77 O7 N1 P1
neg_575	dMePE(16:1e/20:4)	neg	750.5443	C43 H77 O7 N1 P1
neg_576	dMePE(16:1/20:5)	neg	762.5079	C43 H73 O8 N1 P1
neg_577	dMePE(14:0/22:6)	neg	762.5079	C43 H73 O8 N1 P1

neg_578	dMePE(14:0e/22:6)	neg	748.5287	C43 H75 O7 N1 P1
neg_579	dMePE(16:1e/20:5)	neg	748.5287	C43 H75 O7 N1 P1
neg_580	dMePE(14:1e/22:6)	neg	746.5130	C43 H73 O7 N1 P1
neg_581	dMePE(16:0/22:4)	neg	794.5705	C45 H81 O8 N1 P1
neg_582	dMePE(18:0/20:4)	neg	794.5705	C45 H81 O8 N1 P1
neg_583	dMePE(18:0e/20:4)	neg	780.5913	C45 H83 O7 N1 P1
neg_584	dMePE(16:0/22:5)	neg	792.5549	C45 H79 O8 N1 P1
neg_585	dMePE(18:1/20:5)	neg	790.5392	C45 H77 O8 N1 P1
neg_586	dMePE(16:0/22:6)	neg	790.5392	C45 H77 O8 N1 P1
neg_587	dMePE(16:0e/22:6)	neg	776.5600	C45 H79 O7 N1 P1
neg_588	dMePE(16:1/22:6)	neg	788.5236	C45 H75 O8 N1 P1
neg_589	dMePE(16:1e/22:6)	neg	774.5443	C45 H77 O7 N1 P1
neg_590	dMePE(19:0/20:4)	neg	808.5862	C46 H83 O8 N1 P1
neg_591	dMePE(17:1/22:6)	neg	802.5392	C46 H77 O8 N1 P1
neg_592	dMePE(20:5/20:5)	neg	810.5079	C47 H73 O8 N1 P1
neg_593	dMePE(18:1/22:5)	neg	818.5705	C47 H81 O8 N1 P1
neg_594	dMePE(18:0/22:6)	neg	818.5705	C47 H81 O8 N1 P1
neg_595	dMePE(18:0e/22:6)	neg	804.5913	C47 H83 O7 N1 P1
neg_596	dMePE(18:1/22:6)	neg	816.5549	C47 H79 O8 N1 P1
neg_597	dMePE(18:2/22:6)	neg	814.5392	C47 H77 O8 N1 P1
neg_598	dMePE(20:5/20:4)	neg	812.5236	C47 H75 O8 N1 P1
neg_599	dMePE(20:5/22:5)	neg	838.5392	C49 H77 O8 N1 P1
neg_600	dMePE(20:4/22:6)	neg	838.5392	C49 H77 O8 N1 P1

neg_601	dMePE(20:5/22:6)	neg	836.5236	C49 H75 O8 N1 P1
neg_603	dMePE(22:6/22:6)	neg	862.5392	C51 H77 O8 N1 P1
neg_604	phSM(d20:0/14:0)	neg	765.5763	C40 H82 O9 N2 P1
neg_605	phSM(d19:0/20:4)	neg	841.6076	C46 H86 O9 N2 P1
neg_606	phSM(t18:1/16:0)	neg	769.5268	C39 H79 O8 N2 P1 Cl1
neg_607	phSM(t18:1/16:1)	neg	767.5112	C39 H77 O8 N2 P1 Cl1
neg_608	phSM(t18:1/18:0)	neg	797.5581	C41 H83 O8 N2 P1 Cl1
neg_609	phSM(t18:1/18:1)	neg	795.5425	C41 H81 O8 N2 P1 Cl1
neg_610	phSM(t18:1/20:4)	neg	817.5268	C43 H79 O8 N2 P1 Cl1
neg_611	phSM(t18:1/20:5)	neg	815.5112	C43 H77 O8 N2 P1 Cl1
neg_612	phSM(t18:1/22:6)	neg	841.5268	C45 H79 O8 N2 P1 Cl1

1.2 Table S2. The full names of the compound classification correspond to figure 4.

	Classkey	Full name
Figure 4A	ZyE	Zymosterol Ester
	StE	Stigmasterol Ester
	ChE	Cholesterol Ester
	phSM	sphingomyelin(phytosphingosine)
	SPH	Sphingosine
	SM	sphingomyelin
	Hex2Cer	dihexosylceramides
	Hex1Cer	hexaglycosylceramides
	GM3	monosialodihexosylceramide
	GD2	Disialoganglioside（GD2）
	CerG2GNAc1	dihexosyl N-acetylhexosyl ceramide
	Cer	ceramides
	dMePE	dimethyl-PE
	PS	phosphatidylserine
	PMe	phosphatidylmethanol
	PIP2	phosphatidylinositol bisphosphate
	PI	phosphatidylinositol
	PG	phosphatidylglycerol
	PEt	phosphatidylethanol

	PE	phosphatidylethanolamine
	PC	phosphatidylcholine
	MePC	methyl-PC
	MLCL	monolysocardiolipin
	LdMePE	lysodimethylphosphatidylethanolamine
	LPI	lysophosphatidylinositol
	LPG	lysophosphatidylglycerol
	LPEt	lysophosphatidylethanol
	LPE	lysophosphatidylethanolamine
	LPC	lysophosphatidylcholine
	CL	Cardiolipin
	BisMePA	Bis-methyl phosphatidic acid
	TG	triglyceride
	MGDG	Monogalactosyldiacylglycerol
	DG	diglyceride
	WE	wax ester
	Co	Coenzyme
	AcCa	acylcarnitine
Figure 4B	ZyE	Zymosterol Ester
	SPH	Sphingosine

SM	sphingomyelin
Hex3Cer	trihexosylceramides
Hex2Cer	dihexosylceramides
GD1a	Disialoganglioside（GD1a）
Cer	Ceramides
dMePE	dimethyl-PE
PS	phosphatidylserine
PMe	phosphatidylmethanol
PIP2	phosphatidylinositol bisphosphate
PI	phosphatidylinositol
PG	phosphatidylglycerol
PEt	phosphatidylethanol
PE	phosphatidylethanolamine
PC	phosphatidylcholine
MePC	methyl-PC
MLCL	monolysocardiolipin
LdMePE	lysodimethylphosphatidylethanolamine
LPG	lysophosphatidylglycerol
LPE	lysophosphatidylethanolamine
LPC	lysophosphatidylcholine
CL	Cardiolipin

	BisMePA	Bis-methyl phosphatidic acid
	TG	triglyceride
	DG	diglyceride
	OAHFA	(O-acyl) ω-hydroxy fatty acids
	FA	fatty acyl
	Co	Coenzyme
Figure 4C	ZyE	Zymosterol Ester
	StE	Stigmasterol Ester
	ChE	Cholesterol Ester
	PhSM	sphingomyelin(phytosphingosine)
	SPHP	Sphingosine phosphate
	SM	sphingomyelin
	Hex3Cer	trihexosylceramides
	Hex2Cer	dihexosylceramides
	Hex1Cer	hexaglycosylceramides
	GM3	monosialodihexosylceramide
	GD2	Disialoganglioside（GD2）
	Cer	Ceramides
	dMePE	dimethyl-PE
	PS	phosphatidylserine

PMe	phosphatidylmethanol
PI	phosphatidylinositol
PG	phosphatidylglycerol
PEt	phosphatidylethanol
PE	phosphatidylethanolamine
PC	phosphatidylcholine
MePC	methyl-PC
MLCL	monolysocardiolipin
LdMePE	lysodimethylphosphatidylethanolamine
LPI	lysophosphatidylinositol
LPG	lysophosphatidylglycerol
LPEt	lysophosphatidylethanol
LPE	lysophosphatidylethanolamine
LPC	lysophosphatidylcholine
CL	Cardiolipin
BisMePA	Bis-methyl phosphatidic acid
TG	triglyceride
MGDG	Monogalactosyldiacylglycerol
DG	diglyceride
OAHFA	(O-acyl) ω-hydroxy fatty acids
FA	fatty acyl

Co	Coenzyme

1.3 Table S3. Higher abundant levels of lipid species in simple-packaged (SP) pike eels in comparison with fresh (FE) pike eels via LC-MS/MS analysis.

ID	Lipids	Mode	Formula	VIP	FC	*P* value	FDR
pos_2	AcCa(13:0)	pos	C20 H40 O4 N1	1.0049	1.1158	0.0172	0.0367
pos_36	Cer(d16:1/16:0)	pos	C32 H62 O2 N1	1.2584	1.1894	0.0033	0.0129
pos_37	Cer(d17:1/16:0)	pos	C33 H64 O2 N1	0.9592	1.0976	0.0150	0.0333
pos_54	Cer(d16:0/24:1)	pos	C40 H80 O3 N1	1.1409	1.1225	0.0018	0.0095
pos_59	Cer(d19:1/22:0)	pos	C41 H82 O3 N1	1.3060	1.2294	0.0122	0.0288
pos_62	Cer(d19:1/23:0)	pos	C42 H82 O2 N1	1.5331	1.2687	0.0025	0.0109
pos_65	Cer(d18:1/24:1)	pos	C42 H82 O3 N1	0.9129	1.0661	0.0017	0.0095
pos_68	Cer(d17:1/26:1)	pos	C43 H84 O3 N1	0.9546	1.0858	0.0026	0.0111
pos_75	Cer(m18:0/24:0)	pos	C42 H86 O2 N1	1.3626	1.1597	0.0001	0.0052
pos_77	Cer(t16:0/14:0)	pos	C30 H58 O2 N1	1.1956	1.1199	0.0063	0.0186
pos_79	Cer(t17:0/16:0)	pos	C33 H64 O2 N1	1.2883	1.1225	0.0013	0.0080
pos_85	Cer(t16:0/21:0)	pos	C37 H72 O2 N1	0.9159	1.0718	0.0005	0.0064
pos_88	Cer(t16:0/22:3)	pos	C38 H70 O3 N1	0.9481	1.0883	0.0089	0.0230
pos_99	ChE(16:0)	pos	C43 H80 O2 N1	1.6544	1.2597	0.0006	0.0064
pos_102	ChE(18:0)	pos	C45 H84 O2 N1	1.6998	1.2879	0.0004	0.0063
pos_103	ChE(18:1)	pos	C45 H82 O2 N1	2.1196	1.3981	0.0001	0.0052
pos_105	ChE(19:1)	pos	C46 H84 O2 N1	1.6102	1.2589	0.0006	0.0064
pos_106	ChE(20:1)	pos	C47 H86 O2 N1	1.9066	1.3855	0.0006	0.0064
pos_107	ChE(20:2)	pos	C47 H84 O2 N1	1.5726	1.2298	0.0006	0.0064
pos_110	ChE(20:5)	pos	C47 H75 O2	1.8443	1.3518	0.0006	0.0064
pos_111	ChE(22:1)	pos	C49 H90 O2 N1	1.7979	1.3328	0.0002	0.0062

pos_112	ChE(22:2)	pos	C49 H88 O2 N1	1.4904	1.2206	0.0006	0.0064
pos_113	ChE(22:4)	pos	C49 H84 O2 N1	1.7741	1.3073	0.0004	0.0063
pos_114	ChE(22:5)	pos	C49 H82 O2 N1	1.7665	1.2805	0.0003	0.0063
pos_115	ChE(22:6)	pos	C49 H77 O2	1.6945	1.2822	0.0007	0.0064
pos_116	ChE(24:1)	pos	C51 H94 O2 N1	1.3268	1.1891	0.0020	0.0099
pos_117	ChE(24:2)	pos	C51 H92 O2 N1	1.6410	1.3111	0.0005	0.0064
pos_118	ChE(30:5)	pos	C57 H98 O2 N1	0.9858	1.1076	0.0052	0.0164
pos_119	Co(Q10)	pos	C59 H91 O4	0.9777	1.1009	0.0106	0.0262
pos_120	Co(Q8)	pos	C49 H75 O4	1.6634	1.2181	0.0026	0.0112
pos_139	DG(16:0/17:0)	pos	C36 H74 O5 N1	1.0160	1.0701	0.0001	0.0047
pos_213	DG(18:1/20:5)	pos	C41 H69 O5	1.2673	1.1925	0.0065	0.0189
pos_290	DG(33:0/18:1)	pos	C54 H108 O5 N1	1.2397	1.1268	0.0021	0.0102
pos_298	DG(33:1/24:1)	pos	C60 H118 O5 N1	0.9210	1.0685	0.0008	0.0067
pos_299	Hex1Cer(d16:1/16:0)	pos	C38 H74 O8 N1	1.1737	1.1400	0.0034	0.0132
pos_301	Hex1Cer(d16:1/22:0)	pos	C44 H86 O8 N1	1.0020	1.0872	0.0010	0.0072
pos_322	LPC(14:0e)	pos	C22 H49 O6 N1 P1	1.0877	1.0934	0.0007	0.0065
pos_323	LPC(14:1e)	pos	C22 H47 O6 N1 P1	0.9834	1.0927	0.0050	0.0161
pos_326	LPC(16:0e)	pos	C24 H53 O6 N1 P1	1.2507	1.1017	0.0001	0.0060
pos_328	LPC(16:1e)	pos	C24 H51 O6 N1 P1	1.0852	1.0961	0.0008	0.0066
pos_329	LPC(16:2e)	pos	C24 H49 O6 N1 P1	1.4743	1.2151	0.0010	0.0072
pos_330	LPC(17:0)	pos	C25 H53 O7 N1 P1	1.0724	1.0973	0.0023	0.0106
pos_333	LPC(18:0e)	pos	C26 H57 O6 N1 P1	1.5377	1.1743	0.0000	0.0040
pos_335	LPC(18:1e)	pos	C26 H55 O6 N1 P1	1.2344	1.1067	0.0004	0.0063

pos_337	LPC(18:2e)	pos	C26 H53 O6 N1 P1	1.0440	1.1110	0.0063	0.0186
pos_341	LPC(19:0)	pos	C27 H57 O7 N1 P1	1.2261	1.1671	0.0045	0.0154
pos_343	LPC(20:0)	pos	C28 H59 O7 N1 P1	1.3062	1.1333	0.0002	0.0061
pos_344	LPC(20:0e)	pos	C28 H61 O6 N1 P1	1.5429	1.1642	0.0000	0.0012
pos_346	LPC(20:1e)	pos	C28 H59 O6 N1 P1	1.4315	1.1749	0.0011	0.0073
pos_350	LPC(20:3e)	pos	C28 H55 O6 N1 P1	1.2819	1.1901	0.0035	0.0134
pos_352	LPC(20:4e)	pos	C28 H53 O6 N1 P1	1.3780	1.1863	0.0010	0.0071
pos_355	LPC(22:0)	pos	C30 H63 O7 N1 P1	1.2863	1.1616	0.0013	0.0080
pos_362	LPC(23:0)	pos	C31 H65 O7 N1 P1	1.2861	1.2064	0.0069	0.0195
pos_363	LPC(23:1)	pos	C31 H63 O7 N1 P1	1.4082	1.2006	0.0002	0.0062
pos_364	LPC(24:0)	pos	C32 H67 O7 N1 P1	1.1002	1.1277	0.0123	0.0288
pos_366	LPC(24:2)	pos	C32 H63 O7 N1 P1	1.1580	1.1357	0.0006	0.0064
pos_367	LPC(26:1)	pos	C34 H69 O7 N1 P1	1.2462	1.1826	0.0087	0.0226
pos_368	LPC(28:0)	pos	C36 H75 O7 N1 P1	0.8932	1.0571	0.0002	0.0061
pos_373	LPC(32:0)	pos	C40 H82 O7 N1 P1 Na1	0.9396	1.0709	0.0020	0.0099
pos_374	LPC(32:1)	pos	C40 H80 O7 N1 P1 Na1	0.9457	1.0716	0.0016	0.0089
pos_375	LPC(33:1)	pos	C41 H82 O7 N1 P1 Na1	1.1024	1.1182	0.0067	0.0194
pos_376	LPC(34:1)	pos	C42 H84 O7 N1 P1 Na1	1.0769	1.0861	0.0008	0.0067
pos_382	LPE(18:1e)	pos	C23 H48 O6 N1 P1 Na1	1.1591	1.0948	0.0002	0.0062
pos_394	MePC(8:0/11:2)	pos	C28 H52 O8 N1 P1 Na1	1.2238	1.1804	0.0420	0.0720
pos_396	MePC(8:0e/11:4)	pos	C28 H50 O7 N1 P1 Na1	1.3009	1.2039	0.0094	0.0240
pos_400	MePC(11:0/18:2)	pos	C40 H80 O8 N2 P1	1.5779	1.2711	0.0005	0.0064
pos_401	MePC(18:0e/11:4)	pos	C40 H78 O7 N2 P1	1.4365	1.2509	0.0034	0.0131

pos_402	MePC(12:0/18:2)	pos	C41 H82 O8 N2 P1	1.5782	1.3189	0.0007	0.0066
pos_405	MePC(11:0/20:2)	pos	C42 H84 O8 N2 P1	1.8050	1.3175	0.0003	0.0063
pos_406	MePC(11:0/20:3)	pos	C42 H82 O8 N2 P1	1.8481	1.4117	0.0003	0.0063
pos_407	MePC(11:0/20:4)	pos	C42 H80 O8 N2 P1	1.2817	1.1843	0.0016	0.0089
pos_408	MePC(20:0e/11:4)	pos	C42 H82 O7 N2 P1	1.8824	1.4472	0.0003	0.0063
pos_410	MePC(16:1/16:1)	pos	C43 H86 O8 N2 P1	1.7140	1.3813	0.0005	0.0064
pos_411	MePC(14:0e/18:2)	pos	C41 H80 O7 N1 P1 Na1	1.0491	1.1023	0.0024	0.0109
pos_418	MePC(15:0/18:2)	pos	C42 H84 O8 N2 P1	2.0873	1.4048	0.0003	0.0063
pos_419	MePC(15:0/18:3)	pos	C42 H82 O8 N2 P1	1.8488	1.4112	0.0003	0.0063
pos_420	MePC(20:4e/13:0)	pos	C42 H82 O7 N2 P1	1.8616	1.4905	0.0014	0.0084
pos_421	MePC(11:0/22:5)	pos	C44 H82 O8 N2 P1	1.3644	1.2039	0.0019	0.0099
pos_424	MePC(16:1/18:2)	pos	C45 H88 O8 N2 P1	2.3032	2.4903	0.0080	0.0216
pos_437	MePC(17:1/18:2)	pos	C46 H90 O8 N2 P1	1.7759	1.3630	0.0001	0.0060
pos_438	MePC(15:0/20:5)	pos	C44 H82 O8 N2 P1	1.3644	1.2038	0.0019	0.0099
pos_442	MePC(16:0e/20:4)	pos	C45 H84 O7 N1 P1 Na1	4.1363	#######	0.0000	0.0000
pos_451	MePC(19:1/18:2)	pos	C48 H94 O8 N2 P1	1.8894	1.3625	0.0001	0.0052
pos_463	MePC(17:0/22:6)	pos	C50 H92 O8 N2 P1	1.1930	1.1615	0.0045	0.0154
pos_470	MePC(20:4e/21:0)	pos	C50 H94 O7 N1 P1 Na1	1.0224	1.1148	0.0160	0.0349
pos_476	PC(12:0e/6:0)	pos	C26 H54 O7 N1 P1 Na1	1.0209	1.1075	0.0027	0.0113
pos_486	PC(6:0/14:3)	pos	C28 H51 O8 N1 P1	1.1618	1.1756	0.0191	0.0397
pos_487	PC(8:0e/13:0)	pos	C29 H61 O7 N1 P1	1.3458	1.1426	0.0496	0.0813
pos_489	PC(10:0e/11:3)	pos	C29 H55 O7 N1 P1	1.3124	1.1228	0.0003	0.0063
pos_494	PC(4:0/18:3)	pos	C30 H55 O8 N1 P1	1.0677	1.1664	0.0165	0.0356

pos_495	PC(8:1e/15:0)	pos	C31 H63 O7 N1 P1	1.4082	1.2006	0.0002	0.0062
pos_498	PC(12:0e/12:0)	pos	C32 H67 O7 N1 P1	1.1002	1.1276	0.0123	0.0288
pos_500	PC(8:1e/18:2)	pos	C34 H65 O7 N1 P1	1.0282	1.1494	0.0275	0.0522
pos_504	PC(14:0/14:0)	pos	C36 H72 O8 N1 P1 Na1	0.8971	1.0713	0.0022	0.0103
pos_506	PC(16:0/13:0)	pos	C37 H75 O8 N1 P1	1.2603	1.1289	0.0014	0.0085
pos_508	PC(8:0e/21:0)	pos	C37 H77 O7 N1 P1	1.0428	1.0937	0.0004	0.0064
pos_511	PC(11:0/18:2)	pos	C37 H71 O8 N1 P1	1.7068	1.1916	0.0010	0.0072
pos_516	PC(12:0e/18:1)	pos	C38 H77 O7 N1 P1	0.9699	1.0662	0.0001	0.0060
pos_522	PC(8:0/22:3)	pos	C38 H71 O8 N1 P1	0.8971	1.0713	0.0022	0.0103
pos_525	PC(8:0e/23:0)	pos	C39 H81 O7 N1 P1	0.9700	1.0829	0.0086	0.0225
pos_526	PC(15:0/16:1)	pos	C39 H77 O8 N1 P1	0.9854	1.1170	0.0204	0.0416
pos_528	PC(14:0e/17:1)	pos	C39 H79 O7 N1 P1	1.2179	1.0771	0.0000	0.0011
pos_533	PC(20:5/11:4)	pos	C39 H61 O8 N1 P1	1.4623	1.1994	0.0008	0.0066
pos_534	PC(16:0/16:0)	pos	C40 H81 O8 N1 P1	0.9790	1.0760	0.0001	0.0060
pos_542	PC(16:1e/16:1)	pos	C40 H79 O7 N1 P1	0.8923	1.0582	0.0005	0.0064
pos_544	PC(14:1e/18:2)	pos	C40 H77 O7 N1 P1	1.0501	1.1097	0.0398	0.0690
pos_550	PC(18:0/15:0)	pos	C41 H83 O8 N1 P1	1.0099	1.0783	0.0004	0.0063
pos_551	PC(16:0/17:0)	pos	C41 H83 O8 N1 P1	1.0247	1.0704	0.0001	0.0060
pos_556	PC(16:0e/17:1)	pos	C41 H83 O7 N1 P1	0.9308	1.0610	0.0007	0.0066
pos_569	PC(16:1e/18:0)	pos	C42 H85 O7 N1 P1	0.8970	1.0496	0.0003	0.0063
pos_573	PC(16:0e/18:2)	pos	C42 H83 O7 N1 P1	0.9478	1.0640	0.0010	0.0072
pos_574	PC(16:1e/18:1)	pos	C42 H83 O7 N1 P1	0.9410	1.0626	0.0007	0.0065
pos_578	PC(16:1e/18:2)	pos	C42 H81 O7 N1 P1	0.9358	1.0684	0.0014	0.0085

pos_591	PC(18:0/17:0)	pos	C43 H87 O8 N1 P1	0.9606	1.0679	0.0014	0.0084
pos_599	PC(16:2e/19:1)	pos	C43 H83 O7 N1 P1	0.9716	1.0748	0.0010	0.0070
pos_602	PC(20:4e/15:0)	pos	C43 H81 O7 N1 P1	0.9922	1.0872	0.0021	0.0099
pos_605	PC(20:0/16:0)	pos	C44 H89 O8 N1 P1	1.1457	1.1459	0.0211	0.0428
pos_607	PC(18:0/18:1)	pos	C44 H87 O8 N1 P1	1.0652	1.1074	0.0303	0.0559
pos_609	PC(18:0e/18:1)	pos	C44 H89 O7 N1 P1	1.0302	1.0875	0.0014	0.0084
pos_612	PC(18:0e/18:2)	pos	C44 H87 O7 N1 P1	0.9557	1.0622	0.0031	0.0126
pos_614	PC(18:1/18:2)	pos	C44 H83 O8 N1 P1	2.4224	2.0024	0.0014	0.0083
pos_615	PC(16:0/20:3)	pos	C44 H83 O8 N1 P1	1.5955	1.2605	0.0005	0.0064
pos_617	PC(18:1e/18:2)	pos	C44 H85 O7 N1 P1	0.9133	1.0582	0.0013	0.0080
pos_620	PC(16:0e/20:4)	pos	C44 H83 O7 N1 P1	1.5509	1.2509	0.0089	0.0229
pos_621	PC(16:2e/20:2)	pos	C44 H82 O7 N1 P1 Na1	0.9292	1.0625	0.0022	0.0103
pos_622	PC(16:1/20:4)	pos	C44 H79 O8 N1 P1	1.6736	1.2927	0.0004	0.0063
pos_624	PC(16:1e/20:4)	pos	C44 H81 O7 N1 P1	2.1210	1.5568	0.0000	0.0027
pos_628	PC(16:1/20:5)	pos	C44 H77 O8 N1 P1	1.6481	1.3100	0.0019	0.0099
pos_639	PC(19:1/18:2)	pos	C45 H85 O8 N1 P1	0.9565	1.0646	0.0005	0.0064
pos_642	PC(17:0/20:4)	pos	C45 H83 O8 N1 P1	1.8830	1.5072	0.0030	0.0124
pos_646	PC(17:1/20:4)	pos	C45 H81 O8 N1 P1	1.6748	1.3590	0.0013	0.0080
pos_650	PC(15:0/22:6)	pos	C45 H79 O8 N1 P1	1.3674	1.2324	0.0007	0.0066
pos_659	PC(20:1/18:2)	pos	C46 H87 O8 N1 P1	2.5145	1.7615	0.0005	0.0064
pos_663	PC(16:0e/22:4)	pos	C46 H87 O7 N1 P1	1.0227	1.0738	0.0006	0.0064
pos_664	PC(18:2e/20:2)	pos	C46 H86 O7 N1 P1 Na1	0.9725	1.0759	0.0020	0.0099
pos_666	PC(18:1/20:4)	pos	C46 H83 O8 N1 P1	1.9794	1.3824	0.0002	0.0061

pos_670	PC(18:2e/20:3)	pos	C46 H84 O7 N1 P1 Na1	1.0514	1.0945	0.0039	0.0143
pos_673	PC(16:0/22:6)	pos	C46 H81 O8 N1 P1	1.2027	1.1974	0.0275	0.0522
pos_676	PC(16:2e/22:4)	pos	C46 H82 O7 N1 P1 Na1	1.0553	1.0897	0.0034	0.0132
pos_689	PC(17:0/22:3)	pos	C47 H89 O8 N1 P1	0.9885	1.0717	0.0009	0.0070
pos_691	PC(19:0/20:4)	pos	C47 H87 O8 N1 P1	0.9121	1.0603	0.0013	0.0080
pos_692	PC(19:1/20:3)	pos	C47 H87 O8 N1 P1	1.0639	1.0888	0.0018	0.0095
pos_698	PC(17:0/22:6)	pos	C47 H83 O8 N1 P1	1.3108	1.2370	0.0076	0.0210
pos_709	PC(20:0e/20:4)	pos	C48 H91 O7 N1 P1	1.0368	1.0842	0.0018	0.0096
pos_710	PC(20:2e/20:2)	pos	C48 H90 O7 N1 P1 Na1	1.2207	1.1305	0.0003	0.0063
pos_711	PC(20:1/20:4)	pos	C48 H87 O8 N1 P1	1.5719	1.2438	0.0006	0.0064
pos_714	PC(18:3e/22:2)	pos	C48 H88 O7 N1 P1 Na1	0.9950	1.1094	0.0083	0.0221
pos_717	PC(18:0/22:6)	pos	C48 H85 O8 N1 P1	1.8540	1.3917	0.0007	0.0064
pos_720	PC(18:2e/22:4)	pos	C48 H86 O7 N1 P1 Na1	1.1567	1.1021	0.0008	0.0066
pos_721	PC(18:1/22:6)	pos	C48 H83 O8 N1 P1	1.9000	1.4255	0.0002	0.0062
pos_724	PC(18:2/22:6)	pos	C48 H81 O8 N1 P1	1.3297	1.2443	0.0084	0.0222
pos_752	PC(20:2e/22:4)	pos	C50 H90 O7 N1 P1 Na1	1.0360	1.0936	0.0005	0.0064
pos_760	PC(20:3e/22:6)	pos	C50 H85 O7 N1 P1	1.1569	1.1021	0.0008	0.0066
pos_762	PC(22:6/21:1)	pos	C51 H89 O8 N1 P1	1.1052	1.1494	0.0301	0.0557
pos_766	PC(24:0/20:4)	pos	C52 H97 O8 N1 P1	1.9824	1.7252	0.0416	0.0714
pos_768	PC(20:3e/24:2)	pos	C52 H97 O7 N1 P1	1.1301	1.1454	0.0048	0.0158
pos_770	PC(20:4e/24:2)	pos	C52 H95 O7 N1 P1	1.0448	1.1092	0.0043	0.0149
pos_779	PE(16:0/16:1)	pos	C37 H73 O8 N1 P1	1.8846	1.2325	0.0018	0.0095
pos_783	PE(17:1/16:1)	pos	C38 H73 O8 N1 P1	1.0707	1.1284	0.0021	0.0099

pos_788	PE(16:1e/18:1)	pos	C39 H76 O7 N1 P1 Na1	1.1502	1.1260	0.0115	0.0276
pos_871	PG(14:0/14:0)	pos	C34 H68 O10 N0 P1	1.6733	1.3141	0.0004	0.0063
pos_872	PG(16:1/14:0)	pos	C36 H70 O10 N0 P1	1.9663	1.4357	0.0002	0.0060
pos_911	PS(18:0/18:1)	pos	C42 H81 O10 N1 P1	1.3557	1.1608	0.0005	0.0064
pos_914	PS(18:0/22:5)	pos	C46 H81 O10 N1 P1	1.0231	1.1098	0.0189	0.0393
pos_920	SM(d18:1/12:0)	pos	C35 H72 O6 N2 P1	0.9631	1.0726	0.0006	0.0064
pos_921	SM(d18:1/13:0)	pos	C36 H74 O6 N2 P1	0.8952	1.0733	0.0047	0.0156
pos_923	SM(d16:1/16:0)	pos	C37 H75 O6 N2 P1 Na1	0.9458	1.0584	0.0006	0.0064
pos_924	SM(d14:0/18:1)	pos	C37 H76 O6 N2 P1	0.9651	1.0573	0.0004	0.0063
pos_925	SM(d16:1/16:1)	pos	C37 H74 O6 N2 P1	1.0495	1.0985	0.0013	0.0080
pos_926	SM(d14:0/18:4)	pos	C37 H70 O6 N2 P1	0.8980	1.0854	0.0063	0.0186
pos_931	SM(d18:2/15:1)	pos	C38 H74 O6 N2 P1	0.9345	1.0804	0.0031	0.0126
pos_934	SM(d18:2/16:0)	pos	C39 H78 O6 N2 P1	1.0008	1.0722	0.0004	0.0063
pos_936	SM(d16:1/18:3)	pos	C39 H74 O6 N2 P1	0.9458	1.0583	0.0006	0.0064
pos_944	SM(d18:2/18:1)	pos	C41 H80 O6 N2 P1	1.1293	1.1241	0.0111	0.0271
pos_948	SM(d19:1/18:1)	pos	C42 H84 O6 N2 P1	1.0118	1.1085	0.0135	0.0306
pos_949	SM(d18:2/19:1)	pos	C42 H82 O6 N2 P1	1.0099	1.0670	0.0003	0.0063
pos_950	SM(d14:0/23:4)	pos	C42 H80 O6 N2 P1	1.0676	1.0835	0.0102	0.0254
pos_951	SM(d16:1/21:3)	pos	C42 H80 O6 N2 P1	1.1369	1.1224	0.0039	0.0144
pos_954	SM(d20:0/18:1)	pos	C43 H88 O6 N2 P1	1.4712	1.1682	0.0115	0.0275
pos_958	SM(d16:1/22:2)	pos	C43 H84 O6 N2 P1	0.9963	1.0760	0.0006	0.0064
pos_961	SM(d18:2/20:5)	pos	C43 H76 O6 N2 P1	1.2647	1.1370	0.0010	0.0072
pos_965	SM(d18:1/21:2)	pos	C44 H86 O6 N2 P1	1.1630	1.0961	0.0272	0.0519

pos_966	SM(d18:0/21:3)	pos	C44 H86 O6 N2 P1	1.0636	1.0703	0.0000	0.0027
pos_968	SM(d18:1/21:5)	pos	C44 H80 O6 N2 P1	1.1792	1.1039	0.0003	0.0063
pos_973	SM(d18:1/22:2)	pos	C45 H88 O6 N2 P1	0.8803	1.0550	0.0009	0.0069
pos_974	SM(d18:1/22:3)	pos	C45 H86 O6 N2 P1	0.9760	1.0732	0.0005	0.0064
pos_977	SM(d16:1/24:4)	pos	C45 H84 O6 N2 P1	0.9065	1.0620	0.0011	0.0072
pos_979	SM(d20:1/20:5)	pos	C45 H82 O6 N2 P1	1.4347	1.1920	0.0018	0.0095
pos_980	SM(d18:1/22:6)	pos	C45 H80 O6 N2 P1	1.1231	1.1121	0.0405	0.0698
pos_986	SM(d18:1/23:4)	pos	C46 H86 O6 N2 P1	1.1691	1.1108	0.0007	0.0064
pos_987	SM(d18:1/23:5)	pos	C46 H84 O6 N2 P1	1.4228	1.1595	0.0000	0.0037
pos_991	SM(d18:1/24:3)	pos	C47 H90 O6 N2 P1	0.9775	1.0711	0.0003	0.0063
pos_1000	SM(d19:1/24:6)	pos	C48 H86 O6 N2 P1	1.4969	1.1797	0.0009	0.0070
pos_1005	SM(d20:0/24:5)	pos	C49 H92 O6 N2 P1	1.0861	1.1043	0.0455	0.0764
pos_1006	SM(d20:0/24:6)	pos	C49 H90 O6 N2 P1	1.0292	1.0804	0.0002	0.0061
pos_1007	SM(t18:0/14:0)	pos	C37 H78 O7 N2 P1	1.1038	1.1086	0.0007	0.0066
pos_1008	SM(t18:1/14:0)	pos	C37 H76 O7 N2 P1	1.1828	1.1491	0.0127	0.0294
pos_1009	SM(t18:0/16:0)	pos	C39 H82 O7 N2 P1	1.0832	1.1093	0.0042	0.0149
pos_1010	SM(t18:1/21:5)	pos	C44 H80 O7 N2 P1	1.5334	1.3347	0.0109	0.0267
pos_1012	SM(t18:1/22:1)	pos	C45 H90 O7 N2 P1	1.5476	1.2911	0.0006	0.0064
pos_1013	SM(t18:1/22:2)	pos	C45 H88 O7 N2 P1	2.5985	3.6649	0.0040	0.0145
pos_1015	SM(t18:1/22:6)	pos	C45 H79 O7 N2 P1 Li1	2.0091	1.4773	0.0001	0.0060
pos_1025	SPH(t20:0)	pos	C20 H44 O3 N1	0.9119	1.0860	0.0046	0.0155
pos_1028	StE(22:3)	pos	C51 H88 O2 N1	1.4360	1.2249	0.0044	0.0151
pos_1029	StE(22:4)	pos	C51 H86 O2 N1	1.6107	1.2540	0.0015	0.0087

pos_1030	StE(30:5)	pos	C59 H100 O2 N1	0.9386	1.0808	0.0018	0.0096
pos_1041	TG(14:1e/9:0/11:3)	pos	C37 H65 O5	1.2096	1.2090	0.0346	0.0619
pos_1045	TG(16:0/8:0/11:3)	pos	C38 H67 O6	1.3735	1.2034	0.0041	0.0146
pos_1067	TG(14:0/10:2/14:0)	pos	C41 H74 O6 Li1	2.3610	1.4789	0.0000	0.0010
pos_1083	TG(18:3/10:3/11:4)	pos	C42 H60 O6 Li1	1.8223	1.3555	0.0003	0.0063
pos_1095	TG(16:0/6:0/18:2)	pos	C43 H78 O6 Li1	1.6945	1.2811	0.0007	0.0064
pos_1105	TG(20:3/10:3/11:4)	pos	C44 H64 O6 Li1	1.5884	1.2693	0.0042	0.0147
pos_1106	TG(20:4/10:4/11:3)	pos	C44 H62 O6 Li1	1.9663	1.4357	0.0002	0.0060
pos_1112	TG(17:1/10:4/14:4)	pos	C44 H67 O6	1.5475	1.1980	0.0036	0.0136
pos_1119	TG(20:4e/6:0/16:0)	pos	C45 H81 O5	2.0964	1.2231	0.0002	0.0061
pos_1163	TG(15:0/14:0/16:0)	pos	C48 H96 O6 N1	1.1576	1.1559	0.0095	0.0243
pos_1178	TG(16:0e/14:0/16:0)	pos	C49 H100 O5 N1	1.4074	1.1906	0.0023	0.0106
pos_1179	TG(16:0/14:0/16:1)	pos	C49 H92 O6 Na1	0.9934	1.0783	0.0085	0.0224
pos_1198	TG(16:0/14:0/17:1)	pos	C50 H94 O6 Na1	1.1113	1.0984	0.0041	0.0147
pos_1200	TG(16:1/14:0/17:1)	pos	C50 H92 O6 Na1	1.0138	1.1026	0.0157	0.0344
pos_1210	TG(16:0/11:3/20:2)	pos	C50 H86 O6 Li1	2.2670	1.3144	0.0000	0.0009
pos_1218	TG(18:0e/14:0/16:0)	pos	C51 H104 O5 N1	1.4249	1.1753	0.0007	0.0066
pos_1220	TG(16:0/16:0/16:1)	pos	C51 H96 O6 Na1	1.1076	1.0925	0.0025	0.0109
pos_1224	TG(16:0e/16:1/16:1)	pos	C51 H100 O5 N1	1.0406	1.1506	0.0385	0.0672
pos_1233	TG(16:1/14:3/18:1)	pos	C57 H104 O6 N2	1.0574	1.1103	0.0499	0.0818
pos_1246	TG(16:0/16:0/17:0)	pos	C52 H104 O6 N1	1.6679	1.2622	0.0005	0.0064
pos_1249	TG(15:0/16:0/18:1)	pos	C52 H98 O6 Na1	1.0879	1.0873	0.0053	0.0166
pos_1252	TG(16:0/16:1/17:1)	pos	C52 H96 O6 Na1	1.0740	1.0880	0.0072	0.0202

pos_1254	TG(16:1/16:1/17:1)	pos	C52 H94 O6 Na1	1.0150	1.1126	0.0214	0.0432
pos_1267	TG(11:0/18:1/20:5)	pos	C52 H88 O6 Li1	1.9736	1.2424	0.0000	0.0027
pos_1281	TG(16:1e/16:0/18:1)	pos	C53 H104 O5 N1	1.3183	1.1452	0.0006	0.0064
pos_1282	TG(18:1e/16:0/16:1)	pos	C53 H104 O5 N1	1.4293	1.1881	0.0007	0.0065
pos_1288	TG(16:0/14:0/20:4)	pos	C53 H94 O6 Na1	1.4799	1.1739	0.0012	0.0076
pos_1313	TG(16:0/17:1/18:1)	pos	C54 H100 O6 Na1	1.0052	1.0748	0.0051	0.0163
pos_1319	TG(16:1/17:1/18:3)	pos	C54 H94 O6 Li1	1.2216	1.1616	0.0210	0.0428
pos_1333	TG(18:0/16:0/18:0)	pos	C55 H110 O6 N1	1.3343	1.1651	0.0012	0.0077
pos_1334	TG(18:0e/16:0/18:0)	pos	C55 H112 O5 N1	1.2764	1.1581	0.0020	0.0099
pos_1339	TG(18:0e/16:0/18:1)	pos	C55 H107 O5	1.0391	1.0905	0.0021	0.0102
pos_1343	TG(18:1e/16:0/18:1)	pos	C55 H108 O5 N1	1.1369	1.1209	0.0080	0.0217
pos_1346	TG(18:1e/16:1/18:1)	pos	C55 H106 O5 N1	1.1097	1.1318	0.0276	0.0523
pos_1352	TG(16:0e/16:0/20:4)	pos	C55 H104 O5 N1	1.2019	1.1308	0.0036	0.0135
pos_1353	TG(16:0/16:1/20:4)	pos	C55 H96 O6 Na1	1.0678	1.1203	0.0180	0.0380
pos_1377	TG(17:0/18:1/18:1)	pos	C56 H108 O6 N1	1.2027	1.1706	0.0245	0.0477
pos_1386	TG(16:1/17:0/20:5)	pos	C56 H96 O6 Li1	1.3049	1.1329	0.0209	0.0425
pos_1399	TG(18:0/16:0/20:0)	pos	C57 H110 O6 Na1	1.4975	1.1601	0.0274	0.0522
pos_1410	TG(18:0e/18:0/18:1)	pos	C57 H111 O5	0.9843	1.0844	0.0024	0.0108
pos_1412	TG(16:0e/18:1/20:1)	pos	C57 H112 O5 N1	0.9600	1.0710	0.0020	0.0099
pos_1415	TG(18:1e/18:1/18:1)	pos	C57 H110 O5 N1	1.0926	1.1237	0.0043	0.0149
pos_1423	TG(16:0/16:0/22:5)	pos	C57 H104 O6 N1	0.9956	1.1039	0.0203	0.0416
pos_1429	TG(16:1/18:1/20:4)	pos	C57 H98 O6 Na1	0.9845	1.0919	0.0049	0.0159
pos_1442	TG(16:0/16:0/23:0)	pos	C58 H116 O6 N1	1.1885	1.1666	0.0427	0.0727

pos_1452	TG(19:0/18:1/18:1)	pos	C58 H112 O6 N1	0.9872	1.0965	0.0080	0.0216
pos_1470	TG(18:3/17:1/20:4)	pos	C58 H97 O6	1.1071	1.1113	0.0159	0.0348
pos_1474	TG(16:0/16:0/24:0)	pos	C59 H118 O6 N1	1.0853	1.0908	0.0398	0.0690
pos_1499	TG(16:0e/18:1/22:1)	pos	C59 H116 O5 N1	1.0230	1.0678	0.0002	0.0060
pos_1508	TG(18:0e/16:0/22:5)	pos	C59 H110 O5 N1	0.9564	1.0893	0.0198	0.0410
pos_1510	TG(18:0/16:0/22:6)	pos	C59 H102 O6 Na1	0.9272	1.0659	0.0033	0.0130
pos_1515	TG(18:1/18:1/20:5)	pos	C59 H100 O6 Na1	0.9351	1.0654	0.0038	0.0142
pos_1529	TG(15:0/20:4/22:6)	pos	C60 H96 O6 Na1	1.2033	1.1318	0.0103	0.0255
pos_1534	TG(16:0e/18:1/23:0)	pos	C60 H120 O5 N1	1.0510	1.0855	0.0005	0.0064
pos_1548	TG(18:1/17:1/22:6)	pos	C60 H100 O6 Na1	1.0526	1.0830	0.0061	0.0183
pos_1562	TG(18:1/20:5/20:5)	pos	C61 H97 O6	1.0705	1.0976	0.0256	0.0494
pos_1563	TG(14:0/22:6/22:6)	pos	C61 H95 O6	1.5794	1.1794	0.0073	0.0206
pos_1589	TG(18:1/18:1/22:6)	pos	C61 H103 O6	1.0639	1.1476	0.0293	0.0546
pos_1592	TG(18:1/18:3/22:5)	pos	C61 H101 O6	2.4434	1.3183	0.0027	0.0114
pos_1601	TG(15:0/22:5/22:6)	pos	C62 H98 O6 Na1	0.9228	1.0902	0.0132	0.0302
pos_1630	TG(18:1/20:4/22:6)	pos	C63 H100 O6 Na1	0.9449	1.0904	0.0314	0.0574
pos_1636	TG(16:1/22:6/22:6)	pos	C63 H97 O6	1.7935	1.2249	0.0022	0.0103
pos_1662	TG(20:1/18:1/22:6)	pos	C63 H106 O6 Na1	0.9486	1.0743	0.0040	0.0145
pos_1669	TG(22:5/17:1/22:6)	pos	C64 H100 O6 Na1	0.9904	1.0842	0.0088	0.0227
pos_1670	TG(17:0/22:6/22:6)	pos	C64 H100 O6 Na1	1.1400	1.1201	0.0026	0.0112
pos_1695	TG(18:1/22:5/22:5)	pos	C65 H105 O6	0.9486	1.0743	0.0040	0.0145
pos_1728	TG(15:0/24:1/24:1)	pos	C66 H124 O6 Li1	1.9264	1.3653	0.0006	0.0064
pos_1737	TG(24:1/17:1/22:4)	pos	C66 H120 O6 N1	1.0614	1.0994	0.0242	0.0475

pos_1758	TG(20:2/22:6/22:6)	pos	C67 H102 O6 Na1	1.0076	1.1001	0.0139	0.0311
pos_1760	TG(20:3/22:6/22:6)	pos	C67 H101 O6	1.0593	1.1017	0.0068	0.0194
pos_1935	ZyE(20:4)	pos	C47 H75 O2	1.8443	1.3519	0.0006	0.0064
pos_1936	ZyE(20:5)	pos	C47 H73 O2	2.3564	1.4766	0.0000	0.0010
pos_1937	ZyE(22:4)	pos	C49 H82 O2 N1	1.7665	1.2805	0.0003	0.0063
pos_1938	ZyE(22:5)	pos	C49 H77 O2	1.6941	1.2915	0.0007	0.0064
pos_1939	ZyE(22:6)	pos	C49 H75 O2	2.2807	1.4357	0.0000	0.0017
neg_21	CL(23:1/14:0/16:0/24:1)	neg	C86 H162 O17 P2	0.9851	1.0472	0.0010	0.0083
neg_35	CL(23:0/18:1/18:1/22:3)	neg	C90 H164 O17 P2	2.1806	1.4290	0.0035	0.0161
neg_44	Cer(d16:2/19:1)	neg	C36 H66 O5 N1	1.0563	1.0776	0.0082	0.0290
neg_46	Cer(d16:1/21:0)	neg	C38 H74 O5 N1	1.0440	1.0590	0.0002	0.0049
neg_47	Cer(d16:2/21:1)	neg	C38 H70 O5 N1	0.9447	1.0476	0.0011	0.0087
neg_49	Cer(d16:1/22:0+O)	neg	C38 H74 O4 N1	1.5526	1.1595	0.0004	0.0067
neg_50	Cer(d16:2/23:1)	neg	C40 H74 O5 N1	1.0155	1.0521	0.0006	0.0072
neg_51	Cer(d18:0/22:0)	neg	C41 H82 O5 N1	1.7044	1.1899	0.0009	0.0083
neg_58	Cer(d18:0/24:1)	neg	C43 H84 O5 N1	1.8761	1.2105	0.0002	0.0047
neg_61	Cer(d19:1/24:0)	neg	C44 H86 O5 N1	1.3783	1.1893	0.0208	0.0561
neg_62	Cer(d19:1/24:1)	neg	C44 H84 O5 N1	1.1782	1.1541	0.0496	0.1051
neg_65	Cer(m18:0/18:1+O)	neg	C37 H72 O5 N1	1.0757	1.0565	0.0007	0.0080
neg_66	Cer(t16:1/24:1)	neg	C41 H78 O6 N1	1.3694	1.1124	0.0002	0.0060
neg_67	Cer(t18:0/24:1)	neg	C43 H84 O6 N1	1.1639	1.0817	0.0003	0.0062
neg_70	CerG2GNAc1(d20:2/17:0)	neg	C57 H102 O18 N2	1.1897	1.0922	0.0016	0.0107
neg_81	GM3(m22:0/24:0)	neg	C69 H128 O20 N2	1.2791	1.1556	0.0409	0.0934

neg_82	Hex1Cer(d17:0/16:1)	neg	C40 H76 O10 N1	1.2898	1.1462	0.0250	0.0637
neg_83	Hex1Cer(d16:0/18:1)	neg	C41 H78 O10 N1	1.3726	1.1157	0.0053	0.0216
neg_84	Hex1Cer(d21:0/17:1)	neg	C45 H86 O10 N1	1.0853	1.0678	0.0018	0.0112
neg_88	Hex1Cer(t20:1/18:1)	neg	C45 H84 O11 N1	0.9401	1.0497	0.0011	0.0086
neg_102	LPE(20:0)	neg	C25 H51 O7 N1 P1	1.2716	1.0991	0.0013	0.0095
neg_130	LdMePE(14:0e)	neg	C21 H45 O6 N1 P1	1.1354	1.0809	0.0008	0.0083
neg_133	LdMePE(16:0e)	neg	C23 H49 O6 N1 P1	1.4006	1.0976	0.0001	0.0033
neg_135	LdMePE(16:1e)	neg	C23 H47 O6 N1 P1	1.3382	1.1040	0.0004	0.0066
neg_136	LdMePE(17:0)	neg	C24 H49 O7 N1 P1	1.1077	1.0934	0.0093	0.0317
neg_138	LdMePE(18:0)	neg	C25 H51 O7 N1 P1	1.3573	1.1160	0.0005	0.0071
neg_139	LdMePE(18:0e)	neg	C25 H53 O6 N1 P1	1.6108	1.1351	0.0000	0.0009
neg_140	LdMePE(18:1e)	neg	C25 H51 O6 N1 P1	1.4087	1.1110	0.0003	0.0063
neg_143	LdMePE(19:0)	neg	C26 H53 O7 N1 P1	1.4270	1.1374	0.0019	0.0114
neg_146	LdMePE(20:0e)	neg	C27 H57 O6 N1 P1	2.0043	1.2752	0.0000	0.0006
neg_155	LdMePE(24:1)	neg	C31 H61 O7 N1 P1	1.6310	1.2328	0.0023	0.0125
neg_162	MGDG(12:0e/20:0)	neg	C43 H83 O11	1.0032	1.0413	0.0000	0.0015
neg_167	MGDG(18:1/16:0p)	neg	C45 H83 O11	1.0538	1.0617	0.0006	0.0072
neg_191	MLCL(14:2/18:0/18:0)	neg	C59 H110 O16 P2	1.2603	1.0921	0.0010	0.0083
neg_197	MLCL(14:2/20:0/20:0)	neg	C63 H118 O16 P2	1.4725	1.1725	0.0025	0.0131
neg_204	MLCL(14:2/22:0/22:0)	neg	C67 H126 O16 P2	1.5548	1.2101	0.0049	0.0207
neg_208	MLCL(14:2/24:1/24:1)	neg	C71 H130 O16 P2	1.6050	1.1829	0.0025	0.0132
neg_215	PC(14:1e/14:0)	neg	C37 H73 O9 N1 P1	0.9561	1.0668	0.0150	0.0449
neg_219	PC(16:1e/14:0)	neg	C39 H77 O9 N1 P1	0.9837	1.0493	0.0002	0.0060

neg_222	PC(16:0e/16:1)	neg	C41 H81 O9 N1 P1	0.9318	1.0422	0.0004	0.0067
neg_229	PC(17:0/18:1)	neg	C44 H85 O10 N1 P1	1.1179	1.0718	0.0038	0.0173
neg_231	PC(18:2e/17:1)	neg	C44 H83 O9 N1 P1	0.9958	1.0600	0.0016	0.0107
neg_232	PC(20:1e/16:0)	neg	C45 H89 O9 N1 P1	1.1728	1.0848	0.0021	0.0122
neg_233	PC(18:1e/18:1)	neg	C43 H83 O7 N1 P1	0.9329	1.0579	0.0087	0.0302
neg_242	PC(18:1e/20:5)	neg	C47 H83 O9 N1 P1	1.0016	1.0536	0.0012	0.0092
neg_244	PC(17:0/22:4)	neg	C48 H87 O10 N1 P1	0.9068	1.0465	0.0016	0.0107
neg_249	PC(20:0/20:5)	neg	C49 H87 O10 N1 P1	1.4222	1.1653	0.0102	0.0336
neg_259	PC(30:1/20:4)	neg	C57 H103 O8 N1 P1	1.1915	1.1220	0.0357	0.0840
neg_260	PC(30:1/20:5)	neg	C57 H101 O8 N1 P1	1.2609	1.1343	0.0428	0.0951
neg_269	PC(32:1/22:4)	neg	C61 H111 O8 N1 P1	1.2906	1.1247	0.0094	0.0318
neg_270	PC(34:1/20:4)	neg	C61 H111 O8 N1 P1	1.1595	1.1147	0.0260	0.0658
neg_275	PC(34:1/22:6)	neg	C63 H111 O8 N1 P1	1.3811	1.1555	0.0170	0.0481
neg_288	PE(18:0e/16:1)	neg	C39 H77 O7 N1 P1	0.9368	1.0513	0.0010	0.0083
neg_290	PE(16:0/18:2)	neg	C39 H73 O8 N1 P1	1.4772	1.1065	0.0234	0.0613
neg_303	PE(18:0/18:2)	neg	C41 H77 O8 N1 P1	1.0385	1.0914	0.0468	0.1008
neg_363	PE(20:0e/22:6)	neg	C47 H83 O7 N1 P1	0.9426	1.0606	0.0071	0.0264
neg_384	PEt(22:2/22:6)	neg	C49 H80 O8 N0 P1	1.1942	1.0906	0.0106	0.0344
neg_386	PG(15:0/15:0)	neg	C36 H70 O10 N0 P1	1.6351	1.1350	0.0460	0.0995
neg_388	PG(15:0/16:1)	neg	C37 H70 O10 N0 P1	3.3701	8.1772	0.0099	0.0330
neg_464	PS(18:1/18:1)	neg	C42 H77 O10 N1 P1	1.2415	1.1145	0.0459	0.0995
neg_475	PS(18:0/20:4)	neg	C44 H77 O10 N1 P1	1.3570	1.1084	0.0021	0.0119
neg_479	PS(18:0/20:5)	neg	C44 H75 O10 N1 P1	1.1563	1.0872	0.0287	0.0710

neg_490	PS(18:0/22:4)	neg	C46 H81 O10 N1 P1	1.1648	1.0854	0.0048	0.0207
neg_521	PS(20:3/22:6)	neg	C48 H75 O10 N1 P1	1.2497	1.1085	0.0134	0.0408
neg_532	SM(d16:1/20:0)	neg	C42 H84 O8 N2 P1	0.9265	1.0365	0.0001	0.0038
neg_545	dMePE(16:1e/14:0)	neg	C37 H73 O7 N1 P1	1.1848	1.0869	0.0010	0.0083
neg_546	dMePE(16:1/14:1)	neg	C37 H69 O8 N1 P1	1.6117	1.1203	0.0385	0.0894
neg_552	dMePE(16:0e/16:1)	neg	C39 H77 O7 N1 P1	0.9368	1.0513	0.0010	0.0083
neg_555	dMePE(16:1e/16:1)	neg	C39 H75 O7 N1 P1	1.2141	1.1046	0.0079	0.0286
neg_572	dMePE(16:1/20:4)	neg	C43 H75 O8 N1 P1	1.0185	1.1009	0.0393	0.0909
neg_583	dMePE(18:0e/20:4)	neg	C45 H83 O7 N1 P1	1.2539	1.0907	0.0004	0.0069
neg_595	dMePE(18:0e/22:6)	neg	C47 H83 O7 N1 P1	0.9571	1.0615	0.0060	0.0239
neg_604	phSM(d20:0/14:0)	neg	C40 H82 O9 N2 P1	1.0143	1.0606	0.0002	0.0047

1.4 Table S4. Lower abundant levels of lipid species in simple-packaged (SP) pike eels in comparison with fresh (FE) pike eels via LC-MS/MS analysis.

ID	Lipids	Mode	Formula	VIP	FC	*P* value	FDR
pos_4	BisMePA(10:1e/18:1)	pos	C33 H63 O7 N0 P1 Na1	1.4030	0.8426	0.0002	0.0060
pos_26	BisMePA(22:5/22:6)	pos	C49 H79 O8 N1 P1	0.9991	0.9008	0.0204	0.0416
pos_27	BisMePA(22:6/22:6)	pos	C49 H77 O8 N1 P1	1.0486	0.9178	0.0014	0.0084
pos_28	BisMePA(30:0/15:0)	pos	C50 H103 O8 N1 P1	1.3911	0.8425	0.0034	0.0131
pos_126	DG(18:4/12:0)	pos	C33 H56 O5 Na1	1.9383	0.6019	0.0471	0.0782
pos_133	DG(16:1e/16:0)	pos	C35 H68 O4 Na1	0.9529	0.9328	0.0137	0.0310
pos_134	DG(16:1/16:1)	pos	C35 H68 O5 N1	1.3031	0.8704	0.0074	0.0208
pos_136	DG(18:4/14:0)	pos	C35 H64 O5 N1	1.3683	0.8506	0.0068	0.0195
pos_137	DG(12:0/20:5)	pos	C35 H58 O5 Na1	1.4791	0.7266	0.0475	0.0788
pos_142	DG(17:1/16:1)	pos	C36 H70 O5 N1	1.1886	0.8863	0.0081	0.0219
pos_144	DG(18:4/15:0)	pos	C36 H66 O5 N1	1.1108	0.8945	0.0035	0.0135
pos_149	DG(16:1/18:1)	pos	C37 H72 O5 N1	0.9429	0.9369	0.0109	0.0267
pos_150	DG(16:1/18:2)	pos	C37 H70 O5 N1	1.2029	0.8958	0.0021	0.0099
pos_152	DG(16:1/18:3)	pos	C37 H68 O5 N1	1.1869	0.8938	0.0069	0.0195
pos_153	DG(18:4/16:0)	pos	C37 H65 O5	1.2271	0.8851	0.0027	0.0113
pos_154	DG(18:3e/16:1)	pos	C37 H67 O4	1.0633	0.8742	0.0186	0.0390
pos_155	DG(18:4/16:1)	pos	C37 H63 O5	1.2441	0.8812	0.0102	0.0254
pos_156	DG(14:0/20:5)	pos	C37 H63 O5	1.1430	0.9062	0.0014	0.0084
pos_157	DG(20:5/14:1)	pos	C37 H64 O5 N1	2.9119	0.2345	0.0008	0.0066
pos_158	DG(12:0/22:6)	pos	C37 H64 O5 N1	1.2813	0.8690	0.0011	0.0075
pos_162	DG(17:1/18:1)	pos	C38 H74 O5 N1	1.0515	0.9212	0.0010	0.0072

pos_165	DG(15:0/20:4)	pos	C38 H70 O5 N1	1.5102	0.8239	0.0008	0.0066
pos_167	DG(22:6/13:0)	pos	C38 H66 O5 N1	1.1299	0.9004	0.0039	0.0144
pos_171	DG(18:1/18:1)	pos	C39 H72 O5 Na1	1.0026	0.9247	0.0014	0.0085
pos_172	DG(18:0/18:2)	pos	C39 H76 O5 N1	0.9988	0.8975	0.0317	0.0580
pos_173	DG(18:1/18:2)	pos	C39 H74 O5 N1	1.1307	0.9125	0.0020	0.0099
pos_180	DG(16:0/20:5)	pos	C39 H70 O5 N1	1.2009	0.9051	0.0009	0.0070
pos_183	DG(22:5/14:1)	pos	C39 H64 O5 Na1	1.3335	0.8571	0.0021	0.0099
pos_184	DG(16:1/20:5)	pos	C39 H68 O5 N1	1.3761	0.8686	0.0013	0.0082
pos_185	DG(18:4/18:3)	pos	C39 H66 O5 N1	1.3765	0.8484	0.0020	0.0099
pos_186	DG(22:6/14:1)	pos	C39 H66 O5 N1	1.3200	0.8714	0.0012	0.0076
pos_187	DG(18:4/18:4)	pos	C39 H61 O5	1.3152	0.8462	0.0048	0.0158
pos_188	DG(22:6/14:2)	pos	C39 H64 O5 N1	1.1344	0.8602	0.0110	0.0268
pos_189	DG(22:6/14:3)	pos	C39 H62 O5 N1	1.4347	0.8542	0.0002	0.0061
pos_193	DG(17:1/20:2)	pos	C40 H76 O5 N1	1.0381	0.9036	0.0212	0.0429
pos_195	DG(15:0/22:4)	pos	C40 H74 O5 N1	1.0278	0.9045	0.0186	0.0390
pos_197	DG(17:1/20:4)	pos	C40 H72 O5 N1	1.3786	0.8538	0.0008	0.0067
pos_198	DG(15:0/22:5)	pos	C40 H69 O5	1.2044	0.8869	0.0012	0.0078
pos_199	DG(17:0/20:5)	pos	C40 H72 O5 N1	1.8041	0.8743	0.0001	0.0060
pos_200	DG(17:1/20:5)	pos	C40 H70 O5 N1	1.4254	0.8593	0.0001	0.0052
pos_203	DG(20:1/18:1)	pos	C41 H80 O5 N1	0.9417	0.9332	0.0041	0.0147
pos_205	DG(18:1/20:2)	pos	C41 H78 O5 N1	1.1625	0.9001	0.0018	0.0095
pos_207	DG(16:0/22:4)	pos	C41 H76 O5 N1	0.9468	0.9351	0.0019	0.0098
pos_210	DG(18:0/20:5)	pos	C41 H74 O5 N1	1.0281	0.9271	0.0012	0.0077

pos_214	DG(16:1/22:5)	pos	C41 H69 O5	1.3322	0.8687	0.0034	0.0132
pos_215	DG(16:0/22:6)	pos	C41 H72 O5 N1	1.0974	0.9266	0.0011	0.0073
pos_217	DG(16:1/22:6)	pos	C41 H67 O5	1.3778	0.8607	0.0040	0.0145
pos_219	DG(18:4/20:5)	pos	C41 H63 O5	1.3320	0.8573	0.0025	0.0110
pos_221	DG(17:1/22:2)	pos	C42 H80 O5 N1	1.0546	0.8728	0.0277	0.0524
pos_222	DG(19:0/20:3)	pos	C42 H80 O5 N1	1.0746	0.9026	0.0234	0.0463
pos_225	DG(17:0/22:5)	pos	C42 H76 O5 N1	1.1271	0.9033	0.0006	0.0064
pos_226	DG(17:1/22:4)	pos	C42 H73 O5	1.0436	0.9228	0.0005	0.0064
pos_227	DG(17:1/22:5)	pos	C42 H74 O5 N1	0.9654	0.9352	0.0008	0.0067
pos_228	DG(17:0/22:6)	pos	C42 H74 O5 N1	1.0631	0.9182	0.0081	0.0218
pos_229	DG(17:1/22:6)	pos	C42 H72 O5 N1	1.2269	0.8971	0.0011	0.0072
pos_231	DG(18:4/22:6)	pos	C43 H65 O5	1.3923	0.8538	0.0004	0.0063
pos_233	DG(18:1/22:2)	pos	C43 H82 O5 N1	0.9492	0.9235	0.0083	0.0221
pos_237	DG(18:1/22:4)	pos	C43 H78 O5 N1	1.2404	0.8932	0.0010	0.0071
pos_239	DG(18:0/22:5)	pos	C43 H78 O5 N1	0.9624	0.9348	0.0020	0.0099
pos_240	DG(18:1/22:5)	pos	C43 H73 O5	1.2469	0.8949	0.0010	0.0072
pos_241	DG(18:0/22:6)	pos	C43 H76 O5 N1	0.9601	0.9411	0.0012	0.0077
pos_242	DG(20:3/20:4)	pos	C43 H70 O5 Na1	1.3846	0.8523	0.0020	0.0099
pos_243	DG(22:5/18:2)	pos	C43 H71 O5	1.3508	0.8491	0.0052	0.0165
pos_244	DG(18:1/22:6)	pos	C43 H74 O5 N1	1.2251	0.9101	0.0007	0.0065
pos_245	DG(18:3/22:5)	pos	C43 H72 O5 N1	1.4849	0.8285	0.0020	0.0099
pos_246	DG(18:2/22:6)	pos	C43 H72 O5 N1	1.3221	0.8818	0.0010	0.0070
pos_248	DG(18:3/22:6)	pos	C43 H70 O5 N1	2.3756	0.7956	0.0000	0.0009

pos_249	DG(19:0/22:5)	pos	C44 H80 O5 N1	0.9739	0.9253	0.0007	0.0064
pos_250	DG(19:1/22:5)	pos	C44 H78 O5 N1	1.3012	0.8625	0.0040	0.0145
pos_252	DG(19:1/22:6)	pos	C44 H76 O5 N1	1.2735	0.8843	0.0010	0.0071
pos_254	DG(20:5/22:6)	pos	C45 H67 O5	1.2849	0.8895	0.0007	0.0066
pos_258	DG(20:1/22:4)	pos	C45 H82 O5 N1	0.9887	0.9238	0.0039	0.0144
pos_260	DG(20:0/22:5)	pos	C45 H82 O5 N1	1.2797	0.8844	0.0488	0.0805
pos_262	DG(20:0/22:6)	pos	C45 H80 O5 N1	1.1260	0.9172	0.0076	0.0210
pos_264	DG(20:1/22:6)	pos	C45 H78 O5 N1	1.1820	0.9071	0.0008	0.0067
pos_266	DG(20:2/22:6)	pos	C45 H76 O5 N1	1.2837	0.8856	0.0014	0.0085
pos_267	DG(22:5/20:4)	pos	C45 H74 O5 N1	1.1483	0.8829	0.0136	0.0309
pos_269	DG(22:5/21:1)	pos	C46 H82 O5 N1	1.2445	0.8709	0.0030	0.0123
pos_270	DG(22:6/21:0)	pos	C46 H82 O5 N1	1.1478	0.8894	0.0040	0.0145
pos_271	DG(22:6/21:1)	pos	C46 H80 O5 N1	1.2554	0.8591	0.0050	0.0161
pos_272	DG(22:5/22:5)	pos	C47 H73 O5	1.4611	0.8311	0.0008	0.0067
pos_274	DG(22:5/22:6)	pos	C47 H71 O5	1.3791	0.8673	0.0008	0.0067
pos_279	DG(22:0/22:6)	pos	C47 H84 O5 N1	1.1429	0.8894	0.0091	0.0233
pos_282	DG(22:4/22:4)	pos	C47 H80 O5 N1	1.0254	0.9075	0.0070	0.0198
pos_284	DG(22:4/22:5)	pos	C47 H78 O5 N1	1.2472	0.8792	0.0020	0.0099
pos_285	DG(22:6/23:1)	pos	C48 H84 O5 N1	1.1416	0.8935	0.0043	0.0149
pos_286	DG(24:1/22:5)	pos	C49 H88 O5 N1	1.0915	0.9027	0.0023	0.0103
pos_287	DG(24:1/22:6)	pos	C49 H86 O5 N1	1.1554	0.9041	0.0027	0.0114
pos_288	DG(24:2/22:6)	pos	C49 H84 O5 N1	1.2313	0.8527	0.0229	0.0456
pos_292	DG(37:0/16:0)	pos	C56 H110 O5 K1	0.9505	0.9086	0.0238	0.0469

pos_351	LPC(20:4)	pos	C28 H50 O7 N1 P1 Na1	1.1249	0.8808	0.0087	0.0225
pos_353	LPC(20:5)	pos	C28 H48 O7 N1 P1 Na1	1.1820	0.9243	0.0033	0.0129
pos_386	LPE(22:6)	pos	C27 H44 O7 N1 P1 Na1	0.9528	0.9259	0.0022	0.0103
pos_432	MePC(20:4/14:3)	pos	C43 H72 O8 N1 P1 Na1	1.1319	0.8935	0.0004	0.0063
pos_445	MePC(16:1/20:5)	pos	C45 H78 O8 N1 P1 Na1	1.0048	0.9101	0.0113	0.0273
pos_447	MePC(18:4/18:3)	pos	C45 H76 O8 N1 P1 Na1	1.5828	0.8478	0.0000	0.0013
pos_449	MePC(22:6/14:2)	pos	C45 H74 O8 N1 P1 Na1	1.1446	0.9047	0.0004	0.0063
pos_458	MePC(16:1/22:6)	pos	C47 H80 O8 N1 P1 Na1	1.1524	0.8870	0.0009	0.0067
pos_459	MePC(18:4/20:4)	pos	C47 H78 O8 N1 P1 Na1	1.6161	0.8321	0.0004	0.0063
pos_460	MePC(16:2e/22:6)	pos	C47 H80 O7 N1 P1 Na1	1.0766	0.8866	0.0035	0.0134
pos_461	MePC(18:4/20:5)	pos	C47 H76 O8 N1 P1 Na1	1.1281	0.9111	0.0005	0.0064
pos_468	MePC(18:2/22:6)	pos	C49 H82 O8 N1 P1 Na1	1.7305	0.8140	0.0001	0.0060
pos_469	MePC(18:3e/22:6)	pos	C49 H82 O7 N1 P1 Na1	1.1237	0.9150	0.0017	0.0095
pos_474	MePC(20:3/22:6)	pos	C51 H84 O8 N1 P1 Na1	0.9276	0.9222	0.0079	0.0216
pos_505	PC(16:0/12:0)	pos	C36 H73 O8 N1 P1	1.3236	0.8703	0.0017	0.0095
pos_512	PC(16:0/14:0)	pos	C38 H77 O8 N1 P1	0.8592	0.9266	0.0431	0.0732
pos_565	PC(11:0/22:6)	pos	C41 H71 O8 N1 P1	1.0541	0.9247	0.0003	0.0063
pos_632	PC(18:4/18:3)	pos	C44 H75 O8 N1 P1	1.1023	0.8730	0.0279	0.0528
pos_679	PC(20:5/18:2)	pos	C46 H78 O8 N1 P1 Na1	1.0129	0.9226	0.0083	0.0221
pos_684	PC(18:3/20:5)	pos	C46 H76 O8 N1 P1 Na1	1.3545	0.8307	0.0017	0.0093
pos_686	PC(18:4/20:5)	pos	C46 H74 O8 N1 P1 Na1	1.4873	0.8234	0.0063	0.0186
pos_703	PC(20:5/20:5)	pos	C48 H76 O8 N1 P1 Na1	1.0943	0.9027	0.0004	0.0063
pos_729	PC(20:5/20:4)	pos	C48 H78 O8 N1 P1 Na1	0.9805	0.8908	0.0174	0.0369

pos_743	PC(20:5/22:6)	pos	C50 H78 O8 N1 P1 Na1	1.0804	0.9184	0.0003	0.0063
pos_763	PC(22:4/22:6)	pos	C52 H85 O8 N1 P1	1.0817	0.8907	0.0135	0.0306
pos_764	PC(22:5/22:6)	pos	C52 H83 O8 N1 P1	0.9269	0.9256	0.0047	0.0156
pos_765	PC(22:6/22:6)	pos	C52 H80 O8 N1 P1 Na1	0.9559	0.9340	0.0009	0.0070
pos_790	PE(16:1/18:2)	pos	C39 H72 O8 N1 P1 Na1	1.0920	0.9131	0.0002	0.0061
pos_821	PE(16:1/22:6)	pos	C43 H73 O8 N1 P1	1.0276	0.9308	0.0002	0.0062
pos_835	PE(18:1/22:6)	pos	C45 H76 O8 N1 P1 Na1	0.8815	0.9440	0.0004	0.0063
pos_837	PE(18:2/22:6)	pos	C45 H74 O8 N1 P1 Na1	1.1446	0.9047	0.0004	0.0063
pos_839	PE(18:3/22:6)	pos	C45 H72 O8 N1 P1 Na1	1.0927	0.8885	0.0026	0.0112
pos_840	PE(20:4/22:6)	pos	C47 H75 O8 N1 P1	1.0754	0.9204	0.0008	0.0067
pos_841	PE(20:5/22:6)	pos	C47 H73 O8 N1 P1	1.1439	0.9050	0.0004	0.0063
pos_842	PE(20:3/22:5)	pos	C47 H78 O8 N1 P1 Na1	1.5457	0.8404	0.0003	0.0063
pos_843	PE(20:3/22:6)	pos	C47 H76 O8 N1 P1 Na1	1.1281	0.9111	0.0005	0.0064
pos_844	PE(22:4/22:6)	pos	C49 H79 O8 N1 P1	0.9119	0.9099	0.0382	0.0669
pos_845	PE(22:5/22:6)	pos	C49 H77 O8 N1 P1	1.0539	0.9170	0.0013	0.0082
pos_846	PE(22:6/22:6)	pos	C49 H75 O8 N1 P1	1.1261	0.9114	0.0005	0.0064
pos_847	PEt(15:0/12:0)	pos	C32 H64 O8 N0 P1	1.2455	0.8881	0.0009	0.0070
pos_848	PEt(15:0/13:0)	pos	C33 H66 O8 N0 P1	1.2124	0.8829	0.0037	0.0140
pos_849	PEt(15:0/14:0)	pos	C34 H68 O8 N0 P1	1.0902	0.9177	0.0019	0.0099
pos_850	PEt(15:0/14:1)	pos	C34 H66 O8 N0 P1	1.1984	0.8952	0.0041	0.0145
pos_852	PEt(15:0/16:1)	pos	C36 H70 O8 N0 P1	1.1539	0.9072	0.0022	0.0103
pos_853	PEt(15:0/17:0)	pos	C37 H74 O8 N0 P1	1.1041	0.9159	0.0007	0.0065
pos_854	PEt(17:1/15:0)	pos	C37 H72 O8 N0 P1	1.2347	0.8946	0.0006	0.0064

pos_855	PEt(18:0/15:0)	pos	C38 H76 O8 N0 P1	1.3537	0.8768	0.0003	0.0063
pos_857	PEt(15:0/18:2)	pos	C38 H72 O8 N0 P1	1.3860	0.8497	0.0025	0.0109
pos_858	PEt(15:0/19:0)	pos	C39 H78 O8 N0 P1	1.1166	0.9113	0.0004	0.0063
pos_859	PEt(19:1/15:0)	pos	C39 H76 O8 N0 P1	1.2100	0.8927	0.0009	0.0068
pos_860	PEt(20:0/15:0)	pos	C40 H80 O8 N0 P1	1.0679	0.9209	0.0033	0.0129
pos_862	PEt(15:0/20:1)	pos	C40 H78 O8 N0 P1	1.0626	0.9243	0.0005	0.0064
pos_863	PEt(15:0/20:2)	pos	C40 H76 O8 N0 P1	1.0778	0.9277	0.0001	0.0047
pos_864	PEt(15:0/21:0)	pos	C41 H82 O8 N0 P1	1.2101	0.8850	0.0004	0.0063
pos_865	PEt(15:0/22:0)	pos	C42 H84 O8 N0 P1	0.9865	0.9215	0.0020	0.0099
pos_866	PEt(15:0/22:1)	pos	C42 H82 O8 N0 P1	1.0665	0.9159	0.0026	0.0112
pos_868	PEt(15:0/22:5)	pos	C42 H74 O8 N0 P1	1.1849	0.9019	0.0015	0.0086
pos_869	PEt(15:0/22:6)	pos	C42 H72 O8 N0 P1	1.1979	0.9015	0.0029	0.0122
pos_870	PEt(15:0/24:1)	pos	C44 H86 O8 N0 P1	1.0531	0.9186	0.0006	0.0064
pos_887	PMe(16:0/12:0)	pos	C32 H64 O8 N0 P1	1.2455	0.8881	0.0009	0.0070
pos_889	PMe(16:0/14:0)	pos	C34 H68 O8 N0 P1	1.0902	0.9178	0.0019	0.0099
pos_890	PMe(16:0/14:1)	pos	C34 H66 O8 N0 P1	1.1978	0.8951	0.0041	0.0146
pos_892	PMe(16:0/16:1)	pos	C36 H70 O8 N0 P1	1.1572	0.9073	0.0022	0.0103
pos_893	PMe(16:0/17:0)	pos	C37 H74 O8 N0 P1	0.9453	0.9310	0.0012	0.0077
pos_894	PMe(17:1/16:0)	pos	C37 H72 O8 N0 P1	1.2388	0.8941	0.0006	0.0064
pos_895	PMe(18:0/16:0)	pos	C38 H76 O8 N0 P1	1.3537	0.8768	0.0003	0.0063
pos_897	PMe(16:0/18:2)	pos	C38 H72 O8 N0 P1	1.3761	0.8572	0.0031	0.0125
pos_899	PMe(19:1/16:0)	pos	C39 H76 O8 N0 P1	1.2271	0.8764	0.0059	0.0179
pos_900	PMe(20:0/16:0)	pos	C40 H80 O8 N0 P1	1.2087	0.8960	0.0003	0.0063

pos_901	PMe(18:0/18:1)	pos	C40 H78 O8 N0 P1	1.3961	0.8440	0.0037	0.0140
pos_902	PMe(16:0/20:1)	pos	C40 H78 O8 N0 P1	1.0626	0.9243	0.0005	0.0064
pos_903	PMe(16:0/20:2)	pos	C40 H76 O8 N0 P1	1.0778	0.9277	0.0001	0.0047
pos_904	PMe(16:0/20:5)	pos	C40 H70 O8 N0 P1	1.2459	0.8987	0.0006	0.0064
pos_905	PMe(16:0/21:0)	pos	C41 H82 O8 N0 P1	1.2343	0.8810	0.0004	0.0063
pos_906	PMe(16:0/22:0)	pos	C42 H84 O8 N0 P1	0.9865	0.9216	0.0020	0.0099
pos_907	PMe(16:0/22:1)	pos	C42 H82 O8 N0 P1	1.1637	0.9043	0.0010	0.0072
pos_909	PMe(16:0/22:6)	pos	C42 H72 O8 N0 P1	1.1979	0.9015	0.0029	0.0122
pos_910	PMe(16:0/24:1)	pos	C44 H86 O8 N0 P1	1.0531	0.9187	0.0006	0.0064
pos_1033	TG(9:0/10:0/10:0)	pos	C32 H60 O6 K1	1.5083	0.7993	0.0012	0.0077
pos_1035	TG(6:0/13:0/13:0)	pos	C35 H66 O6 K1	1.1788	0.8889	0.0044	0.0151
pos_1036	TG(8:0/10:2/14:0)	pos	C35 H63 O6	1.0653	0.8974	0.0093	0.0238
pos_1037	TG(8:0/11:2/14:0)	pos	C36 H65 O6	1.2899	0.8640	0.0011	0.0075
pos_1038	TG(4:0/15:0/15:0)	pos	C37 H70 O6 K1	1.1884	0.8996	0.0019	0.0099
pos_1040	TG(16:1/6:0/12:2)	pos	C37 H65 O6	0.9975	0.9084	0.0098	0.0248
pos_1044	TG(16:0/8:0/11:2)	pos	C38 H69 O6	0.9481	0.9321	0.0010	0.0072
pos_1046	TG(16:1/8:0/11:2)	pos	C38 H67 O6	1.1317	0.9003	0.0006	0.0064
pos_1047	TG(14:0/10:3/11:2)	pos	C38 H63 O6	0.9568	0.9239	0.0018	0.0095
pos_1049	TG(16:0/10:0/10:2)	pos	C39 H71 O6	1.1003	0.8965	0.0153	0.0337
pos_1051	TG(12:1e/6:0/18:1)	pos	C39 H73 O5	1.0247	0.9198	0.0027	0.0114
pos_1053	TG(18:3e/8:0/10:0)	pos	C39 H71 O5	1.2613	0.8697	0.0025	0.0109
pos_1061	TG(14:1e/6:0/17:1)	pos	C40 H74 O5 Na1	1.0430	0.9228	0.0005	0.0064
pos_1062	TG(8:0/11:2/18:1)	pos	C40 H71 O6	1.1330	0.9068	0.0004	0.0063

pos_1064	TG(14:1e/11:3/12:3)	pos	C40 H65 O5	2.7656	0.3360	0.0002	0.0061
pos_1066	TG(16:0/11:1/11:1)	pos	C41 H75 O6	1.0348	0.9164	0.0074	0.0207
pos_1068	TG(12:1e/8:0/18:1)	pos	C41 H77 O5	1.0379	0.9072	0.0038	0.0141
pos_1069	TG(18:1/10:1/10:1)	pos	C41 H73 O6	1.1025	0.9001	0.0099	0.0249
pos_1072	TG(12:1e/10:4/16:0)	pos	C41 H74 O5 N1	1.1203	0.9197	0.0022	0.0103
pos_1073	TG(12:0e/6:0/20:5)	pos	C41 H71 O5	1.0026	0.9247	0.0014	0.0085
pos_1079	TG(20:4e/8:0/10:4)	pos	C41 H65 O5	1.5174	0.7393	0.0381	0.0668
pos_1080	TG(15:0/6:0/18:1)	pos	C42 H78 O6 K1	1.2590	0.8768	0.0017	0.0094
pos_1081	TG(9:0/13:0/17:1)	pos	C42 H78 O6 K1	1.0626	0.9243	0.0005	0.0064
pos_1084	TG(4:0/17:1/18:1)	pos	C42 H76 O6 K1	1.4540	0.8286	0.0007	0.0065
pos_1085	TG(20:1/8:0/11:2)	pos	C42 H75 O6	1.1790	0.8878	0.0008	0.0066
pos_1086	TG(6:0/13:0/20:4)	pos	C42 H72 O6 K1	1.1174	0.9071	0.0030	0.0124
pos_1087	TG(18:1/10:1/11:3)	pos	C42 H71 O6	0.8976	0.9115	0.0397	0.0689
pos_1089	TG(8:0/11:2/20:5)	pos	C42 H67 O6	1.1148	0.8978	0.0009	0.0070
pos_1092	TG(16:1/10:0/14:0)	pos	C43 H84 O6 N1	1.2613	0.8433	0.0332	0.0600
pos_1096	TG(18:1/11:1/11:1)	pos	C43 H77 O6	1.2340	0.8783	0.0064	0.0188
pos_1097	TG(12:0e/6:0/22:4)	pos	C43 H77 O5	1.1068	0.9097	0.0460	0.0769
pos_1110	TG(8:0/11:2/22:5)	pos	C44 H71 O6	1.3755	0.8543	0.0002	0.0062
pos_1111	TG(8:0/11:2/22:6)	pos	C44 H69 O6	1.8033	0.8059	0.0000	0.0029
pos_1115	TG(10:0/14:0/18:1)	pos	C45 H84 O6 K1	2.0898	0.8351	0.0000	0.0045
pos_1121	TG(10:0/12:2/20:4)	pos	C45 H75 O6	1.4798	0.8331	0.0003	0.0063
pos_1123	TG(12:1e/10:0/20:5)	pos	C45 H77 O5	1.1195	0.8830	0.0100	0.0250
pos_1124	TG(14:0e/6:0/22:6)	pos	C45 H77 O5	1.2059	0.8824	0.0054	0.0169

pos_1127	TG(12:1e/8:0/22:6)	pos	C45 H75 O5	1.4395	0.8380	0.0008	0.0067
pos_1128	TG(10:0/10:2/22:6)	pos	C45 H71 O6	0.9222	0.9295	0.0032	0.0129
pos_1131	TG(9:0/10:0/24:1)	pos	C46 H86 O6 K1	1.0531	0.9186	0.0006	0.0064
pos_1134	TG(22:6/10:1/11:4)	pos	C46 H67 O6	1.1411	0.8910	0.0015	0.0086
pos_1152	TG(16:0/14:0/14:3)	pos	C47 H88 O6 N1	1.0760	0.8936	0.0201	0.0414
pos_1153	TG(16:1/14:0/14:3)	pos	C47 H86 O6 N1	1.2590	0.8341	0.0180	0.0380
pos_1156	TG(16:1/14:0/14:4)	pos	C53 H96 O6 N2	0.9542	0.9225	0.0125	0.0292
pos_1157	TG(16:0/10:4/18:2)	pos	C53 H94 O6 N2	1.2907	0.8484	0.0160	0.0349
pos_1158	TG(12:1e/10:4/22:1)	pos	C47 H81 O5	1.3801	0.8398	0.0025	0.0109
pos_1159	TG(22:5/11:1/11:1)	pos	C47 H77 O6	1.3064	0.8733	0.0002	0.0062
pos_1161	TG(22:6/11:1/11:1)	pos	C47 H75 O6	1.2357	0.8867	0.0009	0.0068
pos_1168	TG(16:1/12:1/17:1)	pos	C48 H90 O6 N1	1.1221	0.8890	0.0302	0.0557
pos_1171	TG(18:4/13:0/14:0)	pos	C48 H88 O6 N1	1.9544	0.7941	0.0001	0.0060
pos_1172	TG(15:0/14:3/16:1)	pos	C48 H88 O6 N1	1.1414	0.8865	0.0062	0.0185
pos_1183	TG(16:0/14:2/16:1)	pos	C49 H92 O6 N1	0.9664	0.9037	0.0325	0.0590
pos_1187	TG(12:0/14:0/20:5)	pos	C49 H84 O6 Na1	0.9697	0.9178	0.0132	0.0302
pos_1189	TG(16:1/10:0/20:5)	pos	C49 H82 O6 Na1	1.1824	0.8759	0.0097	0.0246
pos_1193	TG(16:1/8:0/22:6)	pos	C49 H84 O6 N1	1.5008	0.8177	0.0017	0.0094
pos_1202	TG(16:1/14:1/17:1)	pos	C50 H94 O6 N1	1.9350	0.8376	0.0006	0.0064
pos_1204	TG(16:1/13:0/18:3)	pos	C50 H92 O6 N1	1.2434	0.8620	0.0020	0.0099
pos_1206	TG(18:4/13:0/16:1)	pos	C50 H90 O6 N1	1.0547	0.8729	0.0324	0.0590
pos_1207	TG(14:0/13:0/20:5)	pos	C50 H90 O6 N1	1.2076	0.8935	0.0017	0.0095
pos_1235	TG(16:1/12:0/20:5)	pos	C51 H86 O6 Na1	1.0233	0.9102	0.0137	0.0310

pos_1236	TG(12:0/14:0/22:6)	pos	C51 H86 O6 Na1	1.4403	0.8719	0.0002	0.0062
pos_1239	TG(16:1/10:0/22:6)	pos	C51 H88 O6 N1	1.4403	0.8073	0.0224	0.0450
pos_1240	TG(16:0/10:1/22:6)	pos	C51 H88 O6 N1	1.2130	0.8812	0.0411	0.0706
pos_1242	TG(14:0/14:3/20:5)	pos	C51 H86 O6 N1	1.5325	0.7913	0.0018	0.0097
pos_1243	TG(16:0/10:2/22:6)	pos	C51 H86 O6 N1	1.2654	0.8204	0.0145	0.0324
pos_1244	TG(18:4/14:3/16:1)	pos	C51 H83 O6	0.9697	0.9180	0.0132	0.0302
pos_1245	TG(15:0/11:2/22:6)	pos	C57 H98 O6 N2	1.0145	0.9131	0.0186	0.0390
pos_1264	TG(16:1/13:0/20:5)	pos	C52 H92 O6 N1	1.1301	0.8821	0.0464	0.0772
pos_1276	TG(18:1/10:3/22:6)	pos	C59 H98 O6 N2	1.1061	0.9165	0.0203	0.0416
pos_1298	TG(16:1/14:1/20:5)	pos	C53 H88 O6 Na1	1.1822	0.8789	0.0235	0.0465
pos_1302	TG(12:0/18:3/20:5)	pos	C53 H90 O6 N1	1.3541	0.8765	0.0055	0.0170
pos_1303	TG(16:0/14:3/20:5)	pos	C53 H90 O6 N1	1.4928	0.8130	0.0043	0.0150
pos_1306	TG(16:0/12:3/22:6)	pos	C59 H100 O6 N2	1.4376	0.7901	0.0052	0.0165
pos_1330	TG(20:5/14:2/17:1)	pos	C54 H92 O6 N1	1.0054	0.9023	0.0223	0.0449
pos_1336	TG(18:4/14:1/20:5)	pos	C55 H86 O6 Na1	1.4253	0.8550	0.0015	0.0086
pos_1337	TG(16:0/14:4/22:6)	pos	C55 H90 O6 N1	1.2317	0.8512	0.0362	0.0644
pos_1340	TG(16:0/18:1/18:1)	pos	C55 H106 O6 N1	2.1183	0.8735	0.0292	0.0544
pos_1362	TG(16:1/14:1/22:6)	pos	C55 H90 O6 Na1	0.9916	0.9082	0.0256	0.0494
pos_1365	TG(18:4/16:1/18:4)	pos	C55 H92 O6 N1	1.1895	0.8867	0.0229	0.0456
pos_1371	TG(20:5/13:0/20:5)	pos	C56 H88 O6 Na1	1.5498	0.8301	0.0186	0.0390
pos_1374	TG(20:4/11:2/22:6)	pos	C62 H100 O6 N2	1.0699	0.9257	0.0062	0.0184
pos_1375	TG(20:5/11:3/22:5)	pos	C62 H98 O6 N2	1.2557	0.8922	0.0055	0.0170
pos_1388	TG(14:0/17:1/22:5)	pos	C56 H100 O6 N1	1.0425	0.9074	0.0138	0.0310

pos_1392	TG(16:1/17:1/20:5)	pos	C56 H94 O6 Li1	1.6770	0.8475	0.0100	0.0250
pos_1394	TG(18:4/17:1/18:3)	pos	C56 H96 O6 N1	1.0371	0.8839	0.0441	0.0743
pos_1396	TG(18:4/15:0/20:5)	pos	C56 H90 O6 Na1	1.1917	0.8684	0.0118	0.0281
pos_1398	TG(11:0/20:4/22:5)	pos	C62 H106 O6 N2	1.0985	0.9243	0.0017	0.0094
pos_1405	TG(12:0/20:5/22:6)	pos	C57 H88 O6 Na1	1.3677	0.8694	0.0008	0.0067
pos_1409	TG(20:5/12:2/22:6)	pos	C57 H85 O6	1.4253	0.8550	0.0015	0.0086
pos_1417	TG(16:0/16:0/22:4)	pos	C57 H102 O6 K1	1.7416	0.7438	0.0055	0.0170
pos_1426	TG(16:0/16:0/22:6)	pos	C57 H102 O6 N1	2.0784	0.8254	0.0051	0.0163
pos_1433	TG(16:1/16:1/22:5)	pos	C57 H97 O6	0.9715	0.8930	0.0467	0.0777
pos_1439	TG(16:2e/16:1/22:6)	pos	C57 H98 O5 N1	1.4170	0.8350	0.0028	0.0118
pos_1450	TG(20:5/13:0/22:6)	pos	C58 H90 O6 Li1	1.3312	0.8718	0.0023	0.0106
pos_1471	TG(18:3/17:1/20:5)	pos	C58 H98 O6 N1	1.0148	0.9014	0.0126	0.0293
pos_1483	TG(18:3e/16:1/22:6)	pos	C59 H100 O5 N1	1.3034	0.8455	0.0066	0.0191
pos_1487	TG(20:5/14:1/22:6)	pos	C59 H90 O6 Na1	1.3941	0.8580	0.0036	0.0136
pos_1491	TG(20:5/14:3/22:6)	pos	C59 H90 O6 N1	1.5051	0.8090	0.0056	0.0171
pos_1492	TG(22:5/12:3/22:6)	pos	C59 H87 O6	1.3677	0.8694	0.0008	0.0067
pos_1505	TG(16:1/18:1/22:3)	pos	C59 H104 O6 K1	1.5270	0.7896	0.0125	0.0292
pos_1522	TG(20:5/18:2/18:2)	pos	C59 H96 O6 Na1	1.0824	0.8860	0.0189	0.0393
pos_1523	TG(18:3/18:2/20:4)	pos	C59 H100 O6 N1	1.1742	0.8942	0.0382	0.0669
pos_1561	TG(16:1/20:5/22:5)	pos	C61 H100 O6 N1	1.2061	0.9036	0.0143	0.0319
pos_1567	TG(22:6/14:2/22:6)	pos	C61 H94 O6 N1	1.2886	0.8802	0.0172	0.0367
pos_1568	TG(22:6/14:3/22:6)	pos	C61 H92 O6 N1	1.6430	0.7986	0.0061	0.0183
pos_1610	TG(16:0/21:0/22:4)	pos	C62 H116 O6 N1	1.4347	0.8958	0.0067	0.0194

pos_1627	TG(16:0/22:4/22:6)	pos	C63 H103 O6	1.5492	0.8971	0.0001	0.0060
pos_1637	TG(18:3/20:4/22:6)	pos	C63 H96 O6 Na1	1.4035	0.8359	0.0018	0.0096
pos_1638	TG(18:3/20:5/22:5)	pos	C63 H96 O6 Na1	0.9444	0.9236	0.0024	0.0108
pos_1640	TG(18:3/20:5/22:6)	pos	C63 H95 O6	1.4240	0.8341	0.0030	0.0122
pos_1641	TG(16:2e/22:6/22:6)	pos	C63 H100 O5 N1	1.3851	0.8510	0.0066	0.0190
pos_1642	TG(18:4/20:5/22:6)	pos	C63 H92 O6 Na1	1.1464	0.9043	0.0086	0.0225
pos_1643	TG(20:5/20:5/20:5)	pos	C63 H92 O6 Li1	1.3783	0.8395	0.0033	0.0129
pos_1667	TG(19:0/20:4/22:6)	pos	C64 H108 O6 N1	1.1348	0.8917	0.0095	0.0243
pos_1683	TG(16:0/22:6/23:1)	pos	C64 H114 O6 N1	0.8879	0.9311	0.0244	0.0477
pos_1700	TG(18:3/22:5/22:6)	pos	C65 H102 O6 N1	1.0915	0.9177	0.0211	0.0428
pos_1702	TG(20:5/20:4/22:6)	pos	C65 H97 O6	1.3035	0.8651	0.0078	0.0213
pos_1704	TG(18:3e/22:6/22:6)	pos	C65 H102 O5 N1	1.2097	0.8672	0.0101	0.0252
pos_1705	TG(20:5/20:5/22:6)	pos	C65 H95 O6	1.2136	0.8615	0.0154	0.0338
pos_1714	TG(16:0/22:5/24:1)	pos	C65 H115 O6	1.0901	0.9102	0.0020	0.0099
pos_1716	TG(18:1/22:1/22:5)	pos	C65 H113 O6	1.1849	0.8860	0.0006	0.0064
pos_1732	TG(24:1/17:1/22:2)	pos	C66 H124 O6 N1	1.0653	0.8941	0.0291	0.0543
pos_1738	TG(18:1/22:5/23:0)	pos	C66 H120 O6 N1	1.1702	0.8863	0.0270	0.0517
pos_1742	TG(18:1/22:6/23:0)	pos	C66 H118 O6 N1	1.1575	0.8957	0.0377	0.0663
pos_1761	TG(20:5/22:5/22:6)	pos	C67 H99 O6	0.9718	0.9022	0.0238	0.0469
pos_1773	TG(18:1/22:5/24:1)	pos	C67 H117 O6	1.1856	0.8830	0.0015	0.0086
pos_1800	TG(25:0/18:1/22:5)	pos	C68 H124 O6 N1	1.0865	0.9004	0.0114	0.0275
pos_1802	TG(25:0/18:1/22:6)	pos	C68 H122 O6 N1	0.9848	0.9217	0.0103	0.0255
pos_1806	TG(25:1/18:2/22:6)	pos	C68 H118 O6 N1	1.5369	0.8784	0.0160	0.0349

pos_1831	TG(22:0/22:0/22:6)	pos	C69 H126 O6 N1	0.9909	0.9234	0.0056	0.0172
pos_1839	TG(24:1/20:3/22:5)	pos	C69 H120 O6 N1	1.1904	0.9011	0.0009	0.0070
pos_1859	TG(22:1/22:6/23:0)	pos	C70 H126 O6 N1	1.0377	0.9175	0.0065	0.0189
pos_1861	TG(22:1/22:6/23:1)	pos	C70 H124 O6 N1	1.0196	0.9211	0.0048	0.0158
pos_1881	TG(22:0/22:6/24:1)	pos	C71 H124 O6 Na1	1.0846	0.8932	0.0100	0.0252
pos_1882	TG(24:1/22:1/22:6)	pos	C71 H126 O6 N1	1.1086	0.8973	0.0077	0.0211
pos_1883	TG(24:1/22:4/22:4)	pos	C71 H124 O6 N1	1.2926	0.8632	0.0003	0.0063
pos_1884	TG(24:1/22:2/22:6)	pos	C71 H124 O6 N1	1.0295	0.9112	0.0272	0.0519
pos_1892	TG(24:1/22:6/23:0)	pos	C72 H130 O6 N1	1.0839	0.9032	0.0061	0.0183
pos_1897	TG(26:0/22:6/22:6)	pos	C73 H122 O6 N1	1.1590	0.8749	0.0064	0.0188
pos_1906	TG(24:0/22:6/24:1)	pos	C73 H128 O6 Na1	1.0963	0.8928	0.0127	0.0294
pos_1910	TG(24:1/22:6/24:2)	pos	C73 H128 O6 N1	1.2987	0.8669	0.0065	0.0189
pos_1913	TG(25:0/22:6/24:1)	pos	C74 H134 O6 N1	1.5229	0.8729	0.0018	0.0097
pos_1914	TG(25:1/22:6/24:1)	pos	C74 H132 O6 N1	1.2300	0.8576	0.0057	0.0174
pos_1915	TG(28:0/22:5/22:5)	pos	C75 H130 O6 N1	1.3315	0.8569	0.0438	0.0742
pos_1917	TG(28:0/22:6/22:6)	pos	C75 H126 O6 N1	1.0225	0.8972	0.0376	0.0662
pos_1921	TG(26:0/22:6/24:1)	pos	C75 H136 O6 N1	0.9793	0.9057	0.0047	0.0156
pos_1929	WE(3:0/16:1)	pos	H40 C19 O2 N1	1.7208	0.8868	0.0291	0.0543
neg_1	CL(14:0/16:0/16:0/18:0)	neg	C73 H140 O17 P2	1.7249	0.8317	0.0012	0.0092
neg_2	CL(16:0/16:0/16:0/16:0)	neg	C73 H140 O17 P2	1.9237	0.7796	0.0017	0.0111
neg_3	CL(14:0/16:0/16:1/18:1)	neg	C73 H136 O17 P2	1.7526	0.8382	0.0020	0.0118
neg_4	CL(16:0/16:0/18:0/18:0)	neg	C77 H148 O17 P2	2.0150	0.7716	0.0010	0.0083
neg_5	CL(16:0/16:0/16:0/20:0)	neg	C77 H148 O17 P2	1.2420	0.9194	0.0017	0.0109

neg_6	CL(18:1/16:0/16:0/18:1)	neg	C77 H144 O17 P2	1.5235	0.8872	0.0009	0.0083
neg_7	CL(18:1/16:1/16:1/18:1)	neg	C77 H140 O17 P2	1.9679	0.7817	0.0008	0.0083
neg_10	CL(20:5/16:0/16:1/20:4)	neg	C81 H136 O17 P2	1.7086	0.8630	0.0010	0.0083
neg_12	CL(14:0/18:0/20:4/20:4)	neg	C81 H140 O17 P2	1.8582	0.8114	0.0016	0.0107
neg_17	CL(21:0/16:0/16:0/22:6)	neg	C84 H150 O17 P2	1.1224	0.9480	0.0026	0.0133
neg_18	CL(19:0/16:1/18:1/22:6)	neg	C84 H146 O17 P2	1.0346	0.9427	0.0099	0.0330
neg_23	CL(23:0/16:0/16:0/22:6)	neg	C86 H154 O17 P2	1.2090	0.9328	0.0010	0.0083
neg_24	CL(21:0/16:1/18:1/22:6)	neg	C86 H150 O17 P2	1.1993	0.9174	0.0043	0.0191
neg_29	CL(22:6/15:0/20:0/22:0)	neg	C88 H158 O17 P2	1.0363	0.9440	0.0009	0.0083
neg_31	CL(23:0/16:0/18:0/22:6)	neg	C88 H159 O17 P2	1.0948	0.9400	0.0002	0.0050
neg_34	CL(21:0/16:0/22:5/22:6)	neg	C90 H153 O17 P2	1.2719	0.9074	0.0012	0.0092
neg_38	CL(17:0/22:5/22:6/22:6)	neg	C92 H145 O17 P2	1.6344	0.8433	0.0034	0.0161
neg_78	GD2(d19:0/22:6)	neg	C83 H136 O34 N4	1.5599	0.8581	0.0003	0.0063
neg_91	Hex1Cer(t18:0/22:6)	neg	C47 H80 O11 N1	0.9345	0.9584	0.0013	0.0092
neg_92	Hex2Cer(d12:0/16:0)	neg	C40 H76 O13 N1	1.2012	0.9078	0.0445	0.0977
neg_104	LPE(20:3)	neg	C25 H45 O7 N1 P1	1.4828	0.8542	0.0076	0.0276
neg_105	LPE(20:5)	neg	C25 H41 O7 N1 P1	1.6004	0.8872	0.0000	0.0012
neg_107	LPEt(18:1)	neg	C23 H44 O7 N0 P1	1.3940	0.8551	0.0160	0.0461
neg_109	LPEt(22:6)	neg	C27 H42 O7 N0 P1	1.4132	0.8499	0.0154	0.0455
neg_113	LPG(20:4)	neg	C26 H44 O9 N0 P1	0.9400	0.9315	0.0204	0.0553
neg_114	LPG(20:5)	neg	C26 H42 O9 N0 P1	1.2358	0.8951	0.0029	0.0142
neg_115	LPG(22:6)	neg	C28 H44 O9 N0 P1	1.0608	0.9230	0.0211	0.0567
neg_116	LPI(16:0)	neg	C25 H48 O12 N0 P1	1.4773	0.8663	0.0096	0.0323

neg_118	LPI(18:1)	neg	C27 H50 O12 N0 P1	1.7265	0.8287	0.0026	0.0133
neg_119	LPI(20:1)	neg	C29 H54 O12 N0 P1	3.4673	0.1883	0.0006	0.0072
neg_122	LPI(20:4)	neg	C29 H48 O12 N0 P1	1.3948	0.8926	0.0020	0.0115
neg_123	LPI(20:5)	neg	C29 H46 O12 N0 P1	1.5652	0.8509	0.0012	0.0092
neg_124	LPI(22:5)	neg	C31 H50 O12 N0 P1	1.7518	0.8282	0.0029	0.0141
neg_125	LPI(22:6)	neg	C31 H48 O12 N0 P1	1.5606	0.8765	0.0023	0.0125
neg_141	LdMePE(18:2)	neg	C25 H47 O7 N1 P1	1.0538	0.9320	0.0061	0.0240
neg_142	LdMePE(18:3)	neg	C25 H45 O7 N1 P1	1.7015	0.8490	0.0004	0.0066
neg_150	LdMePE(20:4)	neg	C27 H47 O7 N1 P1	1.1778	0.9261	0.0047	0.0203
neg_151	LdMePE(20:5)	neg	C27 H45 O7 N1 P1	1.5167	0.8954	0.0001	0.0043
neg_153	LdMePE(22:5)	neg	C29 H49 O7 N1 P1	1.1727	0.9246	0.0082	0.0290
neg_154	LdMePE(22:6)	neg	C29 H47 O7 N1 P1	1.3260	0.9216	0.0005	0.0072
neg_196	MLCL(14:2/20:4/20:4)	neg	C63 H102 O16 P2	1.4708	0.9171	0.0001	0.0033
neg_203	MLCL(14:2/22:6/22:6)	neg	C67 H102 O16 P2	1.2764	0.9315	0.0008	0.0083
neg_206	MLCL(18:4/20:4/22:6)	neg	C69 H106 O16 P2	1.4379	0.8632	0.0113	0.0363
neg_255	PC(22:4/22:5)	neg	C53 H87 O10 N1 P1	1.3948	0.8635	0.0408	0.0934
neg_293	PE(14:0/20:4)	neg	C39 H69 O8 N1 P1	1.2648	0.9200	0.0000	0.0030
neg_309	PE(16:1/20:4)	neg	C41 H71 O8 N1 P1	1.2838	0.9152	0.0001	0.0044
neg_310	PE(14:0/22:6)	neg	C41 H69 O8 N1 P1	1.4904	0.8949	0.0000	0.0015
neg_321	PE(15:0/22:6)	neg	C42 H71 O8 N1 P1	1.2415	0.9172	0.0019	0.0113
neg_339	PE(16:2e/22:6)	neg	C43 H71 O7 N1 P1	1.1208	0.9339	0.0052	0.0215
neg_344	PE(17:1/22:6)	neg	C44 H73 O8 N1 P1	1.4502	0.8849	0.0022	0.0122
neg_345	PE(18:4/22:6)	neg	C45 H69 O8 N1 P1	1.6655	0.8475	0.0002	0.0050

neg_352	PE(18:1/22:5)	neg	C45 H77 O8 N1 P1	1.1445	0.9139	0.0128	0.0398
neg_356	PE(20:4/20:4)	neg	C45 H73 O8 N1 P1	1.4082	0.9078	0.0000	0.0016
neg_360	PE(19:1/22:6)	neg	C46 H77 O8 N1 P1	1.2497	0.9147	0.0004	0.0067
neg_364	PE(20:1/22:6)	neg	C47 H79 O8 N1 P1	1.2097	0.9336	0.0000	0.0015
neg_368	PE(22:5/20:4)	neg	C47 H75 O8 N1 P1	1.3108	0.8982	0.0247	0.0633
neg_372	PEt(18:0/18:1)	neg	C41 H78 O8 N0 P1	1.4077	0.8477	0.0187	0.0513
neg_373	PEt(18:1/18:1)	neg	C41 H76 O8 N0 P1	1.4216	0.8438	0.0177	0.0495
neg_375	PEt(16:0/20:5)	neg	C41 H70 O8 N0 P1	1.4159	0.8415	0.0151	0.0451
neg_378	PEt(16:1/22:6)	neg	C43 H70 O8 N0 P1	2.9070	0.2174	0.0126	0.0396
neg_380	PEt(18:1/22:6)	neg	C45 H74 O8 N0 P1	1.1751	0.8762	0.0362	0.0847
neg_404	PG(16:2e/20:4)	neg	C42 H72 O9 N0 P1	1.8699	0.7981	0.0006	0.0072
neg_418	PG(20:5/22:6)	neg	C48 H72 O10 N0 P1	1.2305	0.8761	0.0269	0.0675
neg_420	PG(22:5/22:6)	neg	C50 H76 O10 N0 P1	0.9939	0.9346	0.0176	0.0495
neg_421	PG(22:6/22:6)	neg	C50 H74 O10 N0 P1	0.9856	0.9354	0.0159	0.0460
neg_424	PI(16:0/18:1)	neg	C43 H80 O13 N0 P1	1.3564	0.8953	0.0105	0.0344
neg_427	PI(18:0/18:1)	neg	C45 H84 O13 N0 P1	1.4345	0.8859	0.0061	0.0240
neg_429	PI(16:0/20:4)	neg	C45 H78 O13 N0 P1	0.9971	0.9481	0.0008	0.0083
neg_430	PI(16:1/20:4)	neg	C45 H76 O13 N0 P1	1.2761	0.9007	0.0009	0.0083
neg_433	PI(16:1/20:5)	neg	C45 H74 O13 N0 P1	1.4335	0.8568	0.0023	0.0125
neg_434	PI(14:0/22:6)	neg	C45 H74 O13 N0 P1	1.3905	0.8431	0.0134	0.0408
neg_440	PI(16:1/22:5)	neg	C47 H78 O13 N0 P1	1.5651	0.8451	0.0043	0.0191
neg_442	PI(16:0e/22:6)	neg	C47 H80 O12 N0 P1	1.2105	0.9128	0.0058	0.0235
neg_443	PI(19:0/20:4)	neg	C48 H84 O13 N0 P1	1.3491	0.9148	0.0003	0.0063

neg_444	PI(17:0/22:6)	neg	C48 H80 O13 N0 P1	1.1377	0.9303	0.0017	0.0111
neg_445	PI(17:1/22:6)	neg	C48 H78 O13 N0 P1	1.1482	0.8978	0.0158	0.0458
neg_447	PI(18:0/22:5)	neg	C49 H84 O13 N0 P1	1.0393	0.9425	0.0142	0.0431
neg_450	PI(18:3/22:6)	neg	C49 H76 O13 N0 P1	1.2270	0.8699	0.0127	0.0398
neg_451	PI(19:0/22:6)	neg	C50 H84 O13 N0 P1	0.9399	0.9406	0.0157	0.0458
neg_452	PI(20:5/22:6)	neg	C51 H76 O13 N0 P1	3.0587	0.3151	0.0044	0.0194
neg_453	PI(22:5/20:4)	neg	C51 H80 O13 N0 P1	1.1121	0.8857	0.0294	0.0724
neg_454	PI(22:4/22:6)	neg	C53 H82 O13 N0 P1	2.2126	0.0463	0.0495	0.1051
neg_457	PIP2(20:4e/19:0)	neg	C48 H88 O18 N0 P3	1.3480	0.8950	0.0063	0.0242
neg_467	PS(14:0/22:6)	neg	C42 H69 O10 N1 P1	1.0088	0.9107	0.0310	0.0750
neg_484	PS(18:3/20:5)	neg	C44 H69 O10 N1 P1	2.7791	0.2109	0.0123	0.0390
neg_486	PS(19:0/20:4)	neg	C45 H79 O10 N1 P1	1.2656	0.9125	0.0066	0.0251
neg_487	PS(19:0/20:5)	neg	C45 H77 O10 N1 P1	1.1893	0.9214	0.0082	0.0290
neg_492	PS(20:0/20:5)	neg	C46 H79 O10 N1 P1	1.1156	0.9174	0.0299	0.0734
neg_501	PS(18:3/22:6)	neg	C46 H71 O10 N1 P1	1.4103	0.8659	0.0130	0.0402
neg_502	PS(18:3e/22:6)	neg	C46 H73 O9 N1 P1	1.2607	0.9035	0.0016	0.0107
neg_506	PS(19:0/22:6)	neg	C47 H79 O10 N1 P1	1.0060	0.9461	0.0082	0.0290
neg_507	PS(20:4/22:6)	neg	C48 H73 O10 N1 P1	0.9317	0.9355	0.0457	0.0995
neg_509	PS(20:5/22:6)	neg	C48 H71 O10 N1 P1	1.3128	0.8755	0.0092	0.0315
neg_513	PS(20:1/22:6)	neg	C48 H79 O10 N1 P1	1.1030	0.9359	0.0014	0.0097
neg_517	PS(20:2/22:6)	neg	C48 H77 O10 N1 P1	1.0789	0.9405	0.0010	0.0083
neg_520	PS(20:5/22:4)	neg	C48 H75 O10 N1 P1	1.5228	0.8612	0.0092	0.0315
neg_538	SM(d20:0/24:1)	neg	C50 H100 O8 N2 P1	1.4104	0.8660	0.0261	0.0658

neg_592	dMePE(20:5/20:5)	neg	C47 H73 O8 N1 P1	1.6279	0.8338	0.0010	0.0083
neg_597	dMePE(18:2/22:6)	neg	C47 H77 O8 N1 P1	1.0886	0.9059	0.0352	0.0834
neg_598	dMePE(20:5/20:4)	neg	C47 H75 O8 N1 P1	1.4016	0.8896	0.0006	0.0072
neg_599	dMePE(20:5/22:5)	neg	C49 H77 O8 N1 P1	2.3461	0.5277	0.0217	0.0576
neg_600	dMePE(20:4/22:6)	neg	C49 H77 O8 N1 P1	1.2706	0.9116	0.0005	0.0071
neg_601	dMePE(20:5/22:6)	neg	C49 H75 O8 N1 P1	1.5555	0.8744	0.0003	0.0062
neg_603	dMePE(22:6/22:6)	neg	C51 H77 O8 N1 P1	2.0327	0.8135	0.0000	0.0010
neg_608	phSM(t18:1/18:0)	neg	C41 H83 O8 N2 P1 Cl1	1.2259	0.8958	0.0165	0.470

1.5 Table S5. Higher abundant levels of lipid species in vacuum-packaged (VP) pike eels in comparison with fresh (FE) pike eels via LC-MS/MS analysis.

ID	Lipids	Mode	Formula	VIP	FC	*P* value	FDR
pos_8	BisMePA(12:0e/22:6)	pos	C39 H67 O7 N0 P1 Na1	1.2249	1.0347	0.0026	0.0850
pos_37	Cer(d17:1/16:0)	pos	C33 H64 O2 N1	1.3929	1.0609	0.0495	0.2974
pos_41	Cer(d18:1/16:0)	pos	C34 H68 O3 N1	1.1422	1.0292	0.0038	0.0968
pos_79	Cer(t17:0/16:0)	pos	C33 H64 O2 N1	1.6452	1.0825	0.0343	0.2501
pos_90	Cer(t16:0/23:5)	pos	C39 H68 O3 N1	1.5132	1.0525	0.0028	0.0886
pos_120	Co(Q8)	pos	C49 H75 O4	4.1390	1.3775	0.0000	0.0005
pos_121	Co(Q9)	pos	C54 H83 O4	3.5927	1.2947	0.0001	0.0094
pos_122	DG(16:1/12:0)	pos	C31 H58 O5 Na1	1.4001	1.0455	0.0103	0.1578
pos_124	DG(16:1/14:0)	pos	C33 H66 O5 N1	0.9137	1.0199	0.0246	0.2195
pos_130	DG(16:0/16:0)	pos	C35 H72 O5 N1	1.0945	1.0289	0.0258	0.2256
pos_144	DG(18:4/15:0)	pos	C36 H66 O5 N1	1.1905	1.0401	0.0413	0.2767
pos_151	DG(16:0/18:3)	pos	C37 H67 O5	1.3659	1.0462	0.0268	0.2258
pos_153	DG(18:4/16:0)	pos	C37 H65 O5	1.1334	1.0304	0.0210	0.2036
pos_156	DG(14:0/20:5)	pos	C37 H63 O5	1.2578	1.0324	0.0053	0.1094
pos_180	DG(16:0/20:5)	pos	C39 H70 O5 N1	1.2485	1.0297	0.0073	0.1347
pos_184	DG(16:1/20:5)	pos	C39 H68 O5 N1	1.2765	1.0357	0.0193	0.1993
pos_197	DG(17:1/20:4)	pos	C40 H72 O5 N1	1.2910	1.0359	0.0061	0.1197
pos_198	DG(15:0/22:5)	pos	C40 H69 O5	1.2340	1.0325	0.0050	0.1094
pos_200	DG(17:1/20:5)	pos	C40 H70 O5 N1	1.1882	1.0290	0.0034	0.0931
pos_201	DG(15:0/22:6)	pos	C40 H70 O5 N1	1.9174	1.1040	0.0374	0.2648

pos_207	DG(16:0/22:4)	pos	C41 H76 O5 N1	1.0576	1.0248	0.0161	0.1916
pos_214	DG(16:1/22:5)	pos	C41 H69 O5	0.9615	1.0222	0.0341	0.2501
pos_225	DG(17:0/22:5)	pos	C42 H76 O5 N1	1.6344	1.0492	0.0004	0.0258
pos_235	DG(18:0/22:4)	pos	C43 H80 O5 N1	1.0524	1.0273	0.0223	0.2099
pos_236	DG(20:0/20:4)	pos	C43 H80 O5 N1	1.0051	1.0304	0.0500	0.2993
pos_242	DG(20:3/20:4)	pos	C43 H70 O5 Na1	1.1123	1.0288	0.0199	0.1993
pos_272	DG(22:5/22:5)	pos	C47 H73 O5	0.8831	1.0214	0.0465	0.2905
pos_274	DG(22:5/22:6)	pos	C47 H71 O5	1.0180	1.0218	0.0128	0.1765
pos_284	DG(22:4/22:5)	pos	C47 H78 O5 N1	0.9641	1.0222	0.0225	0.2099
pos_285	DG(22:6/23:1)	pos	C48 H84 O5 N1	1.0586	1.0319	0.0371	0.2648
pos_320	Hex2Cer(d16:1/26:1)	pos	C54 H102 O13 N1	2.4754	1.1664	0.0049	0.1093
pos_378	LPE(16:1)	pos	C21 H42 O7 N1 P1 Na1	3.5775	1.2897	0.0000	0.0001
pos_379	LPE(16:1e)	pos	C21 H44 O6 N1 P1 Na1	1.3907	1.0376	0.0108	0.1580
pos_387	LPG(18:1)	pos	C24 H47 O9 N0 P1 Na1	3.0188	1.4114	0.0341	0.2501
pos_404	MePC(8:0/22:5)	pos	C39 H68 O8 N1 P1 Na1	3.9241	1.3656	0.0000	0.0000
pos_435	MePC(22:6/12:3)	pos	C43 H68 O8 N1 P1 Na1	3.0620	1.2234	0.0000	0.0009
pos_502	PC(11:0/16:1)	pos	C35 H69 O8 N1 P1	3.0466	1.2352	0.0002	0.0152
pos_503	PC(9:0/18:2)	pos	C35 H67 O8 N1 P1	1.7938	1.0951	0.0280	0.2301
pos_511	PC(11:0/18:2)	pos	C37 H71 O8 N1 P1	4.5489	1.4542	0.0000	0.0000
pos_519	PC(16:1/14:1)	pos	C38 H73 O8 N1 P1	3.2323	1.2424	0.0001	0.0116
pos_524	PC(17:0/14:0)	pos	C39 H79 O8 N1 P1	1.4182	1.0483	0.0028	0.0886
pos_530	PC(17:1/14:1)	pos	C39 H75 O8 N1 P1	3.0716	1.1645	0.0000	0.0004
pos_563	PC(11:0/22:5)	pos	C41 H73 O8 N1 P1	1.4253	1.0525	0.0480	0.2928

pos_565	PC(11:0/22:6)	pos	C41 H71 O8 N1 P1	2.2490	1.0998	0.0003	0.0249
pos_614	PC(18:1/18:2)	pos	C44 H83 O8 N1 P1	3.3151	1.5391	0.0304	0.2391
pos_666	PC(18:1/20:4)	pos	C46 H83 O8 N1 P1	2.1186	1.1331	0.0075	0.1368
pos_778	PE(16:1/14:1)	pos	C35 H67 O8 N1 P1	2.0650	1.1311	0.0288	0.2319
pos_779	PE(16:0/16:1)	pos	C37 H73 O8 N1 P1	4.5421	1.4653	0.0000	0.0004
pos_781	PE(16:1/16:1)	pos	C37 H70 O8 N1 P1 Na1	3.4960	1.3013	0.0001	0.0127
pos_783	PE(17:1/16:1)	pos	C38 H73 O8 N1 P1	3.7100	1.3068	0.0000	0.0001
pos_787	PE(16:1/18:1)	pos	C39 H75 O8 N1 P1	3.0716	1.1645	0.0000	0.0004
pos_790	PE(16:1/18:2)	pos	C39 H72 O8 N1 P1 Na1	2.2394	1.0989	0.0003	0.0255
pos_808	PE(16:1/20:5)	pos	C41 H71 O8 N1 P1	2.3976	1.1096	0.0000	0.0071
pos_849	PEt(15:0/14:0)	pos	C34 H68 O8 N0 P1	1.0901	1.0217	0.0032	0.0912
pos_850	PEt(15:0/14:1)	pos	C34 H66 O8 N0 P1	1.0448	1.0265	0.0493	0.2974
pos_854	PEt(17:1/15:0)	pos	C37 H72 O8 N0 P1	1.0747	1.0220	0.0053	0.1094
pos_855	PEt(18:0/15:0)	pos	C38 H76 O8 N0 P1	0.9126	1.0169	0.0129	0.1765
pos_869	PEt(15:0/22:6)	pos	C42 H72 O8 N0 P1	1.2352	1.0284	0.0053	0.1094
pos_872	PG(16:1/14:0)	pos	C36 H70 O10 N0 P1	1.8713	1.1264	0.0047	0.1064
pos_873	PG(16:1/16:1)	pos	C38 H71 O10 N0 P1 Na1	3.2965	1.2678	0.0001	0.0127
pos_875	PG(16:0/18:2)	pos	C40 H75 O10 N0 P1 Na1	2.5756	1.1567	0.0032	0.0912
pos_878	PG(30:0/16:0)	pos	C52 H107 O10 N1 P1	2.0453	1.1090	0.0293	0.2345
pos_882	PI(16:1/22:6)	pos	C47 H81 O13 N1 P1	2.4553	1.1814	0.0080	0.1380
pos_886	PI(22:6/22:6)	pos	C53 H83 O13 N1 P1	2.2671	1.1912	0.0263	0.2258
pos_889	PMe(16:0/14:0)	pos	C34 H68 O8 N0 P1	1.0901	1.0217	0.0032	0.0912
pos_890	PMe(16:0/14:1)	pos	C34 H66 O8 N0 P1	1.0572	1.0268	0.0455	0.2889

pos_893	PMe(16:0/17:0)	pos	C37 H74 O8 N0 P1	1.0629	1.0226	0.0029	0.0912
pos_894	PMe(17:1/16:0)	pos	C37 H72 O8 N0 P1	1.0555	1.0215	0.0065	0.1256
pos_909	PMe(16:0/22:6)	pos	C42 H72 O8 N0 P1	1.2352	1.0284	0.0053	0.1094
pos_1025	SPH(t20:0)	pos	C20 H44 O3 N1	1.5321	1.0654	0.0106	0.1580
pos_1036	TG(8:0/10:2/14:0)	pos	C35 H63 O6	1.0971	1.0347	0.0375	0.2648
pos_1045	TG(16:0/8:0/11:3)	pos	C38 H67 O6	1.6418	1.0959	0.0240	0.2168
pos_1067	TG(14:0/10:2/14:0)	pos	C41 H74 O6 Li1	2.0195	1.1270	0.0061	0.1197
pos_1072	TG(12:1e/10:4/16:0)	pos	C41 H74 O5 N1	0.9772	1.0179	0.0089	0.1397
pos_1083	TG(18:3/10:3/11:4)	pos	C42 H60 O6 Li1	1.6387	1.1222	0.0425	0.2799
pos_1084	TG(4:0/17:1/18:1)	pos	C42 H76 O6 K1	1.5172	1.0439	0.0001	0.0127
pos_1100	TG(12:0e/6:0/22:6)	pos	C43 H73 O5	2.3239	1.1208	0.0034	0.0931
pos_1102	TG(18:4/10:2/12:2)	pos	C43 H67 O6	1.5010	1.0607	0.0265	0.2258
pos_1106	TG(20:4/10:4/11:3)	pos	C44 H62 O6 Li1	1.8713	1.1264	0.0047	0.1064
pos_1112	TG(17:1/10:4/14:4)	pos	C44 H67 O6	1.6304	1.1014	0.0488	0.2970
pos_1115	TG(10:0/14:0/18:1)	pos	C45 H84 O6 K1	1.1827	1.0363	0.0287	0.2319
pos_1124	TG(14:0e/6:0/22:6)	pos	C45 H77 O5	1.2469	1.0387	0.0197	0.1993
pos_1126	TG(10:0/12:2/20:5)	pos	C45 H73 O6	1.2973	1.0420	0.0166	0.1916
pos_1128	TG(10:0/10:2/22:6)	pos	C45 H71 O6	0.9741	1.0226	0.0183	0.1992
pos_1134	TG(22:6/10:1/11:4)	pos	C46 H67 O6	1.1683	1.0376	0.0405	0.2767
pos_1144	TG(18:2/11:2/14:4)	pos	C46 H73 O6	1.7118	1.0629	0.0010	0.0565
pos_1162	TG(12:0e/10:2/22:6)	pos	C47 H77 O5	1.4283	1.0496	0.0089	0.1397
pos_1320	TG(15:0/14:0/22:5)	pos	C54 H95 O6	1.7183	1.0823	0.0195	0.1993
pos_1459	TG(16:0/17:0/22:5)	pos	C58 H106 O6 N1	1.0592	1.0261	0.0243	0.2185

pos_1482	TG(14:0/20:4/22:6)	pos	C59 H95 O6	1.8718	1.0956	0.0233	0.2139
pos_1519	TG(16:1/20:4/20:4)	pos	C59 H97 O6	1.4921	1.0564	0.0082	0.1384
pos_1529	TG(15:0/20:4/22:6)	pos	C60 H96 O6 Na1	1.7790	1.0753	0.0194	0.1993
pos_1563	TG(14:0/22:6/22:6)	pos	C61 H95 O6	1.5502	1.0877	0.0183	0.1992
pos_1591	TG(16:1/20:4/22:4)	pos	C61 H101 O6	2.0916	1.1315	0.0193	0.1993
pos_1625	TG(18:1/20:3/22:6)	pos	C63 H103 O6	2.1188	1.1301	0.0109	0.1580
pos_1836	TG(24:1/20:2/22:5)	pos	C69 H122 O6 N1	2.0386	1.0885	0.0168	0.1929
pos_1936	ZyE(20:5)	pos	C47 H73 O2	2.0333	1.1275	0.0056	0.1123
pos_1939	ZyE(22:6)	pos	C49 H75 O2	1.9877	1.1135	0.0066	0.1256
neg_2	CL(16:0/16:0/16:0/16:0)	neg	C73 H140 O17 P2	1.5816	1.1048	0.0010	0.0148
neg_3	CL(14:0/16:0/16:1/18:1)	neg	C73 H136 O17 P2	1.0938	1.0463	0.0040	0.0325
neg_4	CL(16:0/16:0/18:0/18:0)	neg	C77 H148 O17 P2	1.0954	1.0525	0.0110	0.0631
neg_5	CL(16:0/16:0/16:0/20:0)	neg	C77 H148 O17 P2	0.9840	1.0360	0.0020	0.0217
neg_7	CL(18:1/16:1/16:1/18:1)	neg	C77 H140 O17 P2	1.1358	1.0606	0.0161	0.0781
neg_10	CL(20:5/16:0/16:1/20:4)	neg	C81 H136 O17 P2	1.0323	1.0409	0.0135	0.0711
neg_12	CL(14:0/18:0/20:4/20:4)	neg	C81 H140 O17 P2	1.2261	1.0593	0.0020	0.0217
neg_44	Cer(d16:2/19:1)	neg	C36 H66 O5 N1	1.1433	1.0680	0.0285	0.1132
neg_46	Cer(d16:1/21:0)	neg	C38 H74 O5 N1	0.9860	1.0410	0.0040	0.0325
neg_47	Cer(d16:2/21:1)	neg	C38 H70 O5 N1	1.0063	1.0400	0.0076	0.0508
neg_49	Cer(d16:1/22:0+O)	neg	C38 H74 O4 N1	0.9762	1.0620	0.0365	0.1362
neg_75	FA(22:5)	neg	O2 H33 C22	1.2543	1.0484	0.0010	0.0143
neg_77	GD1a(m19:1/20:4)	neg	C87 H144 O38 N4	0.9308	1.0391	0.0093	0.0575
neg_92	Hex2Cer(d12:0/16:0)	neg	C40 H76 O13 N1	1.8670	1.1690	0.0014	0.0174

neg_95	Hex2Cer(d24:0/22:6)	neg	C60 H104 O15 N1	1.8722	1.1911	0.0142	0.0732
neg_96	Hex3Cer(m21:1/22:6)	neg	C61 H102 O17 N1	1.9874	1.2181	0.0054	0.0398
neg_97	LPE(15:0)	neg	C20 H41 O7 N1 P1	3.0589	1.4538	0.0000	0.0001
neg_110	LPG(15:0)	neg	C21 H42 O9 N0 P1	2.1452	1.2147	0.0006	0.0113
neg_112	LPG(16:1)	neg	C22 H42 O9 N0 P1	3.0458	1.4016	0.0000	0.0000
neg_209	OAHFA(18:1/18:0)	neg	C36 H67 O4	1.2754	1.0676	0.0019	0.0211
neg_210	OAHFA(18:1/20:3)	neg	C38 H65 O4	1.2207	1.0642	0.0025	0.0252
neg_211	OAHFA(20:4/20:3)	neg	C40 H63 O4	1.0191	1.0463	0.0156	0.0764
neg_213	OAHFA(22:5/22:4)	neg	C44 H67 O4	1.3433	1.0792	0.0039	0.0325
neg_259	PC(30:1/20:4)	neg	C57 H103 O8 N1 P1	1.6905	1.1480	0.0341	0.1292
neg_260	PC(30:1/20:5)	neg	C57 H101 O8 N1 P1	1.9059	1.2005	0.0126	0.0675
neg_261	PC(28:1/22:6)	neg	C57 H99 O8 N1 P1	1.9446	1.2027	0.0016	0.0196
neg_262	PC(29:1/22:6)	neg	C58 H101 O8 N1 P1	1.8616	1.2263	0.0119	0.0663
neg_267	PC(30:1/22:6)	neg	C59 H103 O8 N1 P1	1.9016	1.1538	0.0027	0.0262
neg_268	PC(31:1/22:6)	neg	C60 H105 O8 N1 P1	1.5269	1.1376	0.0104	0.0613
neg_273	PC(32:1/22:6)	neg	C61 H107 O8 N1 P1	1.7198	1.1278	0.0078	0.0510
neg_275	PC(34:1/22:6)	neg	C63 H111 O8 N1 P1	1.7732	1.1689	0.0173	0.0825
neg_278	PE(16:1/14:0)	neg	C35 H67 O8 N1 P1	2.7438	1.3211	0.0000	0.0000
neg_287	PE(18:0/16:0)	neg	C39 H77 O8 N1 P1	1.0747	1.0515	0.0041	0.0327
neg_290	PE(16:0/18:2)	neg	C39 H73 O8 N1 P1	2.9303	1.3096	0.0000	0.0003
neg_297	PE(17:1/18:1)	neg	C40 H75 O8 N1 P1	1.6968	1.1060	0.0000	0.0015
neg_302	PE(18:1/18:1)	neg	C41 H77 O8 N1 P1	1.7984	1.1038	0.0000	0.0003
neg_304	PE(18:1/18:2)	neg	C41 H75 O8 N1 P1	1.6893	1.1263	0.0006	0.0112

neg_309	PE(16:1/20:4)	neg	C41 H71 O8 N1 P1	1.4155	1.0732	0.0002	0.0054
neg_371	PEt(12:0e/22:6)	neg	C39 H66 O7 N0 P1	1.2088	1.0516	0.0015	0.0184
neg_385	PG(15:0/13:0)	neg	C34 H66 O10 N0 P1	1.8987	1.2273	0.0101	0.0605
neg_386	PG(15:0/15:0)	neg	C36 H70 O10 N0 P1	1.8066	1.1773	0.0077	0.0510
neg_388	PG(15:0/16:1)	neg	C37 H70 O10 N0 P1	5.0485	14.0898	0.0000	0.0000
neg_389	PG(16:0/16:1)	neg	C38 H72 O10 N0 P1	2.7107	1.3570	0.0000	0.0005
neg_390	PG(12:1e/20:4)	neg	C38 H66 O9 N0 P1	1.0138	1.0461	0.0049	0.0368
neg_392	PG(17:1/16:1)	neg	C39 H72 O10 N0 P1	3.2174	1.8002	0.0080	0.0518
neg_396	PG(18:0/18:1)	neg	C42 H80 O10 N0 P1	0.9689	1.0305	0.0000	0.0011
neg_397	PG(18:1/18:1)	neg	C42 H78 O10 N0 P1	2.4988	1.3314	0.0006	0.0112
neg_400	PG(16:0/20:5)	neg	C42 H72 O10 N0 P1	2.1828	1.4694	0.0488	0.1609
neg_401	PG(16:1/20:4)	neg	C42 H72 O10 N0 P1	1.7216	1.1271	0.0006	0.0112
neg_402	PG(16:1/20:5)	neg	C42 H70 O10 N0 P1	2.0066	1.1751	0.0002	0.0056
neg_407	PG(18:1/20:4)	neg	C44 H76 O10 N0 P1	1.0742	1.0538	0.0126	0.0675
neg_408	PG(16:1/22:5)	neg	C44 H74 O10 N0 P1	2.1898	1.2565	0.0007	0.0114
neg_409	PG(18:1/20:5)	neg	C44 H74 O10 N0 P1	1.5369	1.1048	0.0025	0.0252
neg_432	PI(16:1e/20:4)	neg	C45 H78 O12 N0 P1	1.0376	1.0428	0.0010	0.0148
neg_434	PI(14:0/22:6)	neg	C45 H74 O13 N0 P1	1.6363	1.1649	0.0238	0.1049
neg_455	PI(22:5/22:6)	neg	C53 H80 O13 N0 P1	1.6784	1.1686	0.0182	0.0853
neg_458	PMe(16:1/16:1)	neg	C36 H66 O8 N0 P1	2.8475	1.4050	0.0000	0.0000
neg_459	PMe(16:1/18:1)	neg	C38 H70 O8 N0 P1	3.1707	1.4927	0.0000	0.0000
neg_461	PS(16:1/18:2)	neg	C40 H71 O10 N1 P1	2.9759	1.3780	0.0000	0.0000
neg_462	PS(16:1/18:3)	neg	C40 H69 O10 N1 P1	3.3426	1.5192	0.0000	0.0000

neg_465	PS(18:1/18:2)	neg	C42 H75 O10 N1 P1	1.7740	1.1112	0.0000	0.0003
neg_484	PS(18:3/20:5)	neg	C44 H69 O10 N1 P1	2.3912	1.4796	0.0329	0.1261
neg_498	PS(18:2/22:6)	neg	C46 H73 O10 N1 P1	1.3548	1.1150	0.0353	0.1328
neg_501	PS(18:3/22:6)	neg	C46 H71 O10 N1 P1	1.1545	1.0754	0.0409	0.1455
neg_545	dMePE(16:1e/14:0)	neg	C37 H73 O7 N1 P1	1.6508	1.1153	0.0012	0.0156
neg_546	dMePE(16:1/14:1)	neg	C37 H69 O8 N1 P1	3.3704	1.4639	0.0000	0.0000

1.6 Table S6. Lower abundant levels of lipid species in vacuum-packaged (VP) pike eels in comparison with fresh (FE) pike eels via LC-MS/MS analysis.

ID	Lipids	Mode	Formula	VIP	FC	*P* value	FDR
pos_16	BisMePA(18:0/20:5)	pos	C43 H79 O8 N1 P1	1.6024	0.9535	0.0016	0.0722
pos_27	BisMePA(22:6/22:6)	pos	C49 H77 O8 N1 P1	1.7568	0.9406	0.0024	0.0850
pos_29	CL(20:5/20:4/22:4/24:1)	pos	C95 H159 O17 P2	3.1060	0.8246	0.0000	0.0001
pos_327	LPC(16:1)	pos	C24 H49 O7 N1 P1	1.8296	0.9288	0.0345	0.2501
pos_331	LPC(17:1)	pos	C25 H51 O7 N1 P1	1.6180	0.9365	0.0459	0.2889
pos_340	LPC(18:4)	pos	C26 H47 O7 N1 P1	1.8919	0.9104	0.0109	0.1580
pos_347	LPC(20:2)	pos	C28 H54 O7 N1 P1 Na1	1.8589	0.9222	0.0311	0.2418
pos_349	LPC(20:3)	pos	C28 H53 O7 N1 P1	1.7114	0.9329	0.0198	0.1993
pos_351	LPC(20:4)	pos	C28 H50 O7 N1 P1 Na1	2.8179	0.8169	0.0003	0.0219
pos_353	LPC(20:5)	pos	C28 H48 O7 N1 P1 Na1	2.0274	0.9060	0.0198	0.1993
pos_359	LPC(22:4)	pos	C30 H54 O7 N1 P1 Na1	1.8210	0.9152	0.0344	0.2501
pos_360	LPC(22:5)	pos	C30 H52 O7 N1 P1 Na1	2.3264	0.8878	0.0042	0.1022
pos_370	LPC(30:0)	pos	C38 H78 O7 N1 P1 Na1	1.1229	0.9685	0.0369	0.2648
pos_373	LPC(32:0)	pos	C40 H82 O7 N1 P1 Na1	1.2869	0.9608	0.0239	0.2168
pos_384	LPE(20:4)	pos	C25 H44 O7 N1 P1 Na1	0.9854	0.9716	0.0467	0.2905
pos_390	LdMePE(18:1)	pos	C25 H51 O7 N1 P1	1.6185	0.9365	0.0457	0.2889
pos_409	MePC(16:0/16:1)	pos	C41 H84 O8 N2 P1	1.5641	0.9545	0.0047	0.1064
pos_412	MePC(10:0/22:3)	pos	C41 H80 O8 N2 P1	1.6162	0.9142	0.0268	0.2258
pos_440	MePC(18:1/18:2)	pos	C45 H84 O8 N1 P1 Na1	1.7892	0.9427	0.0009	0.0546
pos_445	MePC(16:1/20:5)	pos	C45 H78 O8 N1 P1 Na1	2.1927	0.8659	0.0080	0.1380

pos_447	MePC(18:4/18:3)	pos	C45 H76 O8 N1 P1 Na1	1.9320	0.9040	0.0018	0.0730
pos_449	MePC(22:6/14:2)	pos	C45 H74 O8 N1 P1 Na1	1.4793	0.9506	0.0139	0.1787
pos_450	MePC(19:0/18:2)	pos	C46 H88 O8 N1 P1 Na1	1.4243	0.9629	0.0018	0.0730
pos_458	MePC(16:1/22:6)	pos	C47 H80 O8 N1 P1 Na1	1.9371	0.9093	0.0080	0.1380
pos_468	MePC(18:2/22:6)	pos	C49 H82 O8 N1 P1 Na1	1.6638	0.9226	0.0202	0.2005
pos_481	PC(8:0e/11:4)	pos	C27 H49 O7 N1 P1	2.0205	0.8837	0.0177	0.1953
pos_493	PC(4:0/18:2)	pos	C30 H57 O8 N1 P1	1.2708	0.9520	0.0491	0.2974
pos_501	PC(11:0/16:0)	pos	C35 H71 O8 N1 P1	2.0600	0.8900	0.0037	0.0954
pos_504	PC(14:0/14:0)	pos	C36 H72 O8 N1 P1 Na1	2.1034	0.8843	0.0210	0.2036
pos_506	PC(16:0/13:0)	pos	C37 H75 O8 N1 P1	1.5902	0.9211	0.0324	0.2469
pos_509	PC(16:1/13:0)	pos	C37 H73 O8 N1 P1	2.4536	0.8435	0.0014	0.0722
pos_513	PC(14:0e/16:0)	pos	C38 H79 O7 N1 P1	0.9387	0.9811	0.0294	0.2345
pos_514	PC(16:1/14:0)	pos	C38 H74 O8 N1 P1 Na1	2.1179	0.9042	0.0035	0.0931
pos_518	PC(12:0/18:2)	pos	C38 H73 O8 N1 P1	1.8585	0.8853	0.0337	0.2501
pos_520	PC(14:1e/16:1)	pos	C38 H75 O7 N1 P1	1.6014	0.9280	0.0416	0.2767
pos_522	PC(8:0/22:3)	pos	C38 H71 O8 N1 P1	2.1034	0.8843	0.0210	0.2036
pos_534	PC(16:0/16:0)	pos	C40 H81 O8 N1 P1	1.0214	0.9724	0.0431	0.2802
pos_536	PC(16:0/16:1)	pos	C40 H78 O8 N1 P1 Na1	1.3841	0.9562	0.0345	0.2501
pos_540	PC(16:1/16:1)	pos	C40 H76 O8 N1 P1 Na1	1.7167	0.9346	0.0106	0.1580
pos_543	PC(10:0/22:3)	pos	C40 H75 O8 N1 P1	2.4206	0.8730	0.0026	0.0850
pos_554	PC(17:1/16:0)	pos	C41 H80 O8 N1 P1 Na1	1.9588	0.9235	0.0037	0.0954
pos_557	PC(15:0/18:2)	pos	C41 H79 O8 N1 P1	1.5871	0.9519	0.0045	0.1064
pos_569	PC(16:1e/18:0)	pos	C42 H85 O7 N1 P1	0.9981	0.9797	0.0442	0.2827

pos_572	PC(16:0/18:2)	pos	C42 H80 O8 N1 P1 Na1	2.2908	0.9082	0.0002	0.0162
pos_573	PC(16:0e/18:2)	pos	C42 H83 O7 N1 P1	1.3694	0.9603	0.0135	0.1787
pos_576	PC(16:0/18:3)	pos	C42 H78 O8 N1 P1 Na1	1.8048	0.9337	0.0017	0.0722
pos_579	PC(16:1/18:3)	pos	C42 H77 O8 N1 P1	1.9553	0.8759	0.0322	0.2469
pos_583	PC(14:0/20:5)	pos	C42 H74 O8 N1 P1 Na1	2.2288	0.8957	0.0012	0.0624
pos_601	PC(17:1/18:3)	pos	C43 H79 O8 N1 P1	1.5486	0.9374	0.0280	0.2301
pos_610	PC(18:1/18:1)	pos	C44 H84 O8 N1 P1 Na1	1.6724	0.9555	0.0006	0.0436
pos_617	PC(18:1e/18:2)	pos	C44 H85 O7 N1 P1	1.1841	0.9710	0.0137	0.1787
pos_619	PC(18:4/18:0)	pos	C44 H81 O8 N1 P1	1.7112	0.9409	0.0172	0.1941
pos_623	PC(16:0/20:5)	pos	C44 H78 O8 N1 P1 Na1	2.0737	0.9241	0.0021	0.0800
pos_627	PC(14:1e/22:4)	pos	C44 H81 O7 N1 P1	1.1302	0.9709	0.0148	0.1864
pos_629	PC(14:0/22:6)	pos	C44 H76 O8 N1 P1 Na1	1.9005	0.9286	0.0025	0.0850
pos_643	PC(19:1/18:3)	pos	C45 H83 O8 N1 P1	1.7693	0.9340	0.0069	0.1280
pos_644	PC(15:0/22:4)	pos	C45 H83 O8 N1 P1	0.9762	0.9766	0.0373	0.2648
pos_648	PC(17:0/20:5)	pos	C45 H80 O8 N1 P1 Na1	1.7165	0.9288	0.0086	0.1397
pos_651	PC(17:1/20:5)	pos	C45 H78 O8 N1 P1 Na1	1.8633	0.9308	0.0015	0.0722
pos_656	PC(20:1/18:1)	pos	C46 H88 O8 N1 P1 Na1	1.2467	0.9612	0.0336	0.2501
pos_662	PC(16:0/22:4)	pos	C46 H84 O8 N1 P1 Na1	1.7755	0.9280	0.0177	0.1953
pos_668	PC(18:0/20:5)	pos	C46 H82 O8 N1 P1 Na1	1.8481	0.9364	0.0016	0.0722
pos_674	PC(16:1/22:5)	pos	C46 H81 O8 N1 P1	1.8022	0.9238	0.0130	0.1765
pos_675	PC(18:1/20:5)	pos	C46 H80 O8 N1 P1 Na1	1.6822	0.9419	0.0084	0.1397
pos_679	PC(20:5/18:2)	pos	C46 H78 O8 N1 P1 Na1	1.4981	0.9431	0.0200	0.1993
pos_684	PC(18:3/20:5)	pos	C46 H76 O8 N1 P1 Na1	2.2184	0.8831	0.0032	0.0912

pos_686	PC(18:4/20:5)	pos	C46 H74 O8 N1 P1 Na1	2.2969	0.8340	0.0198	0.1993
pos_689	PC(17:0/22:3)	pos	C47 H89 O8 N1 P1	0.9275	0.9790	0.0418	0.2774
pos_690	PC(19:1/20:2)	pos	C47 H89 O8 N1 P1	1.2943	0.9541	0.0386	0.2682
pos_692	PC(19:1/20:3)	pos	C47 H87 O8 N1 P1	1.5472	0.9522	0.0018	0.0730
pos_699	PC(17:1/22:5)	pos	C47 H82 O8 N1 P1 Na1	2.4979	0.8554	0.0133	0.1787
pos_703	PC(20:5/20:5)	pos	C48 H76 O8 N1 P1 Na1	1.9528	0.9160	0.0019	0.0730
pos_705	PC(16:0/24:2)	pos	C48 H93 O8 N1 P1	1.0457	0.9704	0.0473	0.2918
pos_729	PC(20:5/20:4)	pos	C48 H78 O8 N1 P1 Na1	2.0851	0.8728	0.0253	0.2239
pos_730	PC(18:3/22:6)	pos	C48 H79 O8 N1 P1	1.7252	0.9289	0.0153	0.1907
pos_732	PC(18:3e/22:6)	pos	C48 H81 O7 N1 P1	1.9779	0.9030	0.0082	0.1384
pos_743	PC(20:5/22:6)	pos	C50 H78 O8 N1 P1 Na1	1.8410	0.9285	0.0061	0.1197
pos_744	PC(26:1/16:1)	pos	C50 H97 O8 N1 P1	0.9777	0.9748	0.0269	0.2258
pos_745	PC(20:0/22:4)	pos	C50 H93 O8 N1 P1	1.6432	0.9359	0.0044	0.1056
pos_747	PC(20:0/22:5)	pos	C50 H91 O8 N1 P1	1.6662	0.9387	0.0041	0.1010
pos_748	PC(31:1/11:4)	pos	C50 H91 O8 N1 P1	1.6413	0.9352	0.0101	0.1561
pos_752	PC(20:2e/22:4)	pos	C50 H90 O7 N1 P1 Na1	1.1088	0.9652	0.0431	0.2802
pos_761	PC(22:6/21:0)	pos	C51 H91 O8 N1 P1	1.9211	0.9229	0.0021	0.0800
pos_762	PC(22:6/21:1)	pos	C51 H89 O8 N1 P1	1.6239	0.9195	0.0191	0.1993
pos_765	PC(22:6/22:6)	pos	C52 H80 O8 N1 P1 Na1	1.2835	0.9596	0.0386	0.2682
pos_771	PC(22:1/22:6)	pos	C52 H91 O8 N1 P1	1.9939	0.9167	0.0036	0.0954
pos_774	PC(24:1/22:6)	pos	C54 H95 O8 N1 P1	2.1158	0.9163	0.0002	0.0175
pos_798	PE(18:0/18:1)	pos	C41 H80 O8 N1 P1 Na1	1.6459	0.9523	0.0008	0.0486
pos_812	PE(18:0/20:4)	pos	C43 H79 O8 N1 P1	1.6024	0.9533	0.0016	0.0722

pos_827	PE(18:0/22:5)	pos	C45 H81 O8 N1 P1	1.5152	0.9576	0.0007	0.0453
pos_831	PE(18:0/22:6)	pos	C45 H79 O8 N1 P1	1.2657	0.9721	0.0015	0.0722
pos_833	PE(18:1p/22:5)	pos	C45 H79 O7 N1 P1	0.8965	0.9808	0.0273	0.2266
pos_835	PE(18:1/22:6)	pos	C45 H76 O8 N1 P1 Na1	1.1475	0.9681	0.0222	0.2099
pos_837	PE(18:2/22:6)	pos	C45 H74 O8 N1 P1 Na1	1.4793	0.9506	0.0139	0.1787
pos_840	PE(20:4/22:6)	pos	C47 H75 O8 N1 P1	1.5664	0.9339	0.0337	0.2501
pos_841	PE(20:5/22:6)	pos	C47 H73 O8 N1 P1	1.4832	0.9503	0.0135	0.1787
pos_845	PE(22:5/22:6)	pos	C49 H77 O8 N1 P1	1.7571	0.9408	0.0024	0.0850
pos_880	PI(18:0/20:4)	pos	C47 H87 O13 N1 P1	2.2107	0.8846	0.0122	0.1737
pos_883	PI(18:0/22:6)	pos	C49 H87 O13 N1 P1	2.0629	0.9034	0.0266	0.2258
pos_911	PS(18:0/18:1)	pos	C42 H81 O10 N1 P1	2.4629	0.8535	0.0030	0.0912
pos_914	PS(18:0/22:5)	pos	C46 H81 O10 N1 P1	2.2446	0.8855	0.0023	0.0831
pos_915	PS(18:0/22:6)	pos	C46 H79 O10 N1 P1	2.0543	0.9018	0.0253	0.2239
pos_925	SM(d16:1/16:1)	pos	C37 H74 O6 N2 P1	1.1560	0.9630	0.0329	0.2486
pos_934	SM(d18:2/16:0)	pos	C39 H78 O6 N2 P1	1.2288	0.9618	0.0266	0.2258
pos_946	SM(d18:2/18:3)	pos	C41 H76 O6 N2 P1	1.9075	0.8916	0.0310	0.2418
pos_949	SM(d18:2/19:1)	pos	C42 H82 O6 N2 P1	1.3083	0.9667	0.0126	0.1765
pos_951	SM(d16:1/21:3)	pos	C42 H80 O6 N2 P1	1.2861	0.9496	0.0477	0.2928
pos_955	SM(d16:1/22:0)	pos	C43 H87 O6 N2 P1 Na1	1.4669	0.9558	0.0100	0.1561
pos_958	SM(d16:1/22:2)	pos	C43 H84 O6 N2 P1	1.1337	0.9675	0.0427	0.2799
pos_963	SM(d18:0/21:2)	pos	C44 H88 O6 N2 P1	1.1723	0.9677	0.0479	0.2928
pos_966	SM(d18:0/21:3)	pos	C44 H86 O6 N2 P1	1.2251	0.9646	0.0235	0.2147
pos_968	SM(d18:1/21:5)	pos	C44 H80 O6 N2 P1	1.5245	0.9474	0.0165	0.1916

pos_971	SM(d16:1/24:1)	pos	C45 H89 O6 N2 P1 Na1	1.5872	0.9541	0.0068	0.1273
pos_975	SM(d16:1/24:3)	pos	C45 H86 O6 N2 P1	1.4886	0.9548	0.0087	0.1397
pos_980	SM(d18:1/22:6)	pos	C45 H80 O6 N2 P1	1.6427	0.9207	0.0282	0.2301
pos_982	SM(d18:1/23:1)	pos	C46 H92 O6 N2 P1	1.2316	0.9661	0.0435	0.2802
pos_984	SM(d20:0/21:3)	pos	C46 H90 O6 N2 P1	1.2454	0.9617	0.0412	0.2767
pos_986	SM(d18:1/23:4)	pos	C46 H86 O6 N2 P1	1.5649	0.9379	0.0231	0.2138
pos_987	SM(d18:1/23:5)	pos	C46 H84 O6 N2 P1	1.7048	0.9275	0.0047	0.1064
pos_988	SM(d18:1/24:0)	pos	C47 H96 O6 N2 P1	1.2665	0.9642	0.0213	0.2054
pos_989	SM(d18:1/24:1)	pos	C47 H94 O6 N2 P1	1.9704	0.8980	0.0381	0.2673
pos_991	SM(d18:1/24:3)	pos	C47 H90 O6 N2 P1	1.4771	0.9440	0.0442	0.2827
pos_992	SM(d19:1/23:3)	pos	C47 H90 O6 N2 P1	1.2169	0.9685	0.0109	0.1580
pos_993	SM(d18:2/24:3)	pos	C47 H88 O6 N2 P1	1.5568	0.9551	0.0086	0.1397
pos_997	SM(d19:0/24:4)	pos	C48 H92 O6 N2 P1	1.2460	0.9648	0.0434	0.2802
pos_1006	SM(d20:0/24:6)	pos	C49 H90 O6 N2 P1	1.1952	0.9625	0.0461	0.2889
pos_1017	SM(t18:1/24:0)	pos	C47 H96 O7 N2 P1	1.6176	0.9338	0.0164	0.1916
pos_1256	TG(15:0/16:1/18:3)	pos	C52 H96 O6 N1	0.9542	0.9829	0.0089	0.1397
pos_1398	TG(11:0/20:4/22:5)	pos	C62 H106 O6 N2	1.4156	0.9620	0.0079	0.1380
pos_1432	TG(16:0/18:3/20:4)	pos	C57 H96 O6 Li1	2.6488	0.8359	0.0053	0.1094
pos_1523	TG(18:3/18:2/20:4)	pos	C59 H100 O6 N1	1.4433	0.9599	0.0169	0.1929
pos_1803	TG(19:1/22:6/24:1)	pos	C68 H120 O6 N1	1.6445	0.9328	0.0210	0.2036
neg_9	CL(24:0/14:0/16:0/18:0)	neg	C81 H156 O17 P2	1.2031	0.9385	0.0039	0.0325
neg_14	CL(23:0/15:0/16:0/20:0)	neg	C83 H160 O17 P2	1.1014	0.9561	0.0038	0.0325
neg_19	CL(24:0/16:0/16:0/20:0)	neg	C85 H164 O17 P2	0.9602	0.9677	0.0000	0.0005

neg_31	CL(23:0/16:0/18:0/22:6)	neg	C88 H159 O17 P2	1.4172	0.9282	0.0003	0.0076
neg_34	CL(21:0/16:0/22:5/22:6)	neg	C90 H153 O17 P2	1.2878	0.9323	0.0017	0.0199
neg_141	LdMePE(18:2)	neg	C25 H47 O7 N1 P1	1.2311	0.9336	0.0098	0.0592
neg_142	LdMePE(18:3)	neg	C25 H45 O7 N1 P1	1.2837	0.9116	0.0085	0.0541
neg_150	LdMePE(20:4)	neg	C27 H47 O7 N1 P1	1.0046	0.9524	0.0482	0.1607
neg_151	LdMePE(20:5)	neg	C27 H45 O7 N1 P1	1.2776	0.9338	0.0145	0.0732
neg_153	LdMePE(22:5)	neg	C29 H49 O7 N1 P1	1.3939	0.9124	0.0369	0.1371
neg_154	LdMePE(22:6)	neg	C29 H47 O7 N1 P1	1.1659	0.9490	0.0146	0.0732
neg_184	MLCL(14:2/15:0/15:0)	neg	C53 H98 O16 P2	1.0286	0.9410	0.0410	0.1455
neg_190	MLCL(11:2/16:1/22:4)	neg	C58 H98 O16 P2	1.5427	0.8860	0.0285	0.1132
neg_193	MLCL(14:2/18:2/18:2)	neg	C59 H102 O16 P2	1.0769	0.9438	0.0478	0.1605
neg_195	MLCL(11:3/20:4/22:6)	neg	C62 H94 O16 P2	1.0403	0.9368	0.0461	0.1581
neg_196	MLCL(14:2/20:4/20:4)	neg	C63 H102 O16 P2	1.1366	0.9517	0.0191	0.0868
neg_200	MLCL(14:2/20:3/20:3)	neg	C63 H106 O16 P2	1.0945	0.9411	0.0468	0.1585
neg_203	MLCL(14:2/22:6/22:6)	neg	C67 H102 O16 P2	1.1135	0.9561	0.0188	0.0866
neg_206	MLCL(18:4/20:4/22:6)	neg	C69 H106 O16 P2	1.6014	0.8820	0.0059	0.0430
neg_224	PC(17:1/16:1)	neg	C42 H79 O10 N1 P1	1.0097	0.9624	0.0006	0.0112
neg_227	PC(14:0/20:4)	neg	C43 H77 O10 N1 P1	1.3118	0.9412	0.0002	0.0056
neg_241	PC(18:2e/20:4)	neg	C46 H82 O7 N1 P1 Cl1	1.4262	0.9333	0.0004	0.0078
neg_256	PC(24:0/22:6)	neg	C55 H97 O10 N1 P1	3.5298	0.2071	0.0110	0.0631
neg_333	PE(16:1/22:5)	neg	C43 H73 O8 N1 P1	1.1700	0.9319	0.0298	0.1167
neg_342	PE(19:0/20:4)	neg	C44 H79 O8 N1 P1	1.4169	0.9010	0.0262	0.1092
neg_360	PE(19:1/22:6)	neg	C46 H77 O8 N1 P1	1.3762	0.9171	0.0070	0.0496

neg_403	PG(14:0/22:6)	neg	C42 H70 O10 N0 P1	1.4787	0.9008	0.0032	0.0291
neg_417	PG(20:4/22:6)	neg	C48 H74 O10 N0 P1	1.0354	0.9478	0.0073	0.0498
neg_422	PG(28:1/20:5)	neg	C54 H94 O10 N0 P1	1.0511	0.9398	0.0372	0.1372
neg_435	PI(17:0/20:4)	neg	C46 H80 O13 N0 P1	1.0028	0.9544	0.0094	0.0577
neg_439	PI(18:0/20:5)	neg	C47 H80 O13 N0 P1	1.2336	0.9400	0.0103	0.0609
neg_443	PI(19:0/20:4)	neg	C48 H84 O13 N0 P1	1.1578	0.9487	0.0072	0.0498
neg_444	PI(17:0/22:6)	neg	C48 H80 O13 N0 P1	1.2271	0.9387	0.0069	0.0490
neg_446	PI(18:0/22:4)	neg	C49 H86 O13 N0 P1	1.3736	0.9068	0.0121	0.0670
neg_447	PI(18:0/22:5)	neg	C49 H84 O13 N0 P1	1.3993	0.9208	0.0111	0.0631
neg_457	PIP2(20:4e/19:0)	neg	C48 H88 O18 N0 P3	1.2016	0.9306	0.0298	0.1167
neg_468	PS(18:0/19:0)	neg	C43 H83 O10 N1 P1	1.1159	0.9627	0.0022	0.0230
neg_472	PS(18:0/20:0)	neg	C44 H85 O10 N1 P1	1.3272	0.9313	0.0024	0.0245
neg_475	PS(18:0/20:4)	neg	C44 H77 O10 N1 P1	1.5603	0.8937	0.0044	0.0348
neg_487	PS(19:0/20:5)	neg	C45 H77 O10 N1 P1	1.0496	0.9478	0.0384	0.1408
neg_488	PS(17:0/22:6)	neg	C45 H75 O10 N1 P1	1.1477	0.9317	0.0145	0.0732
neg_492	PS(20:0/20:5)	neg	C46 H79 O10 N1 P1	1.1970	0.9479	0.0017	0.0199
neg_505	PS(20:5/21:0)	neg	C47 H81 O10 N1 P1	1.0865	0.9583	0.0029	0.0268
neg_510	PS(20:0/22:6)	neg	C48 H81 O10 N1 P1	1.1187	0.9585	0.0007	0.0113
neg_513	PS(20:1/22:6)	neg	C48 H79 O10 N1 P1	1.3297	0.9328	0.0008	0.0122
neg_514	PS(20:2/22:5)	neg	C48 H79 O10 N1 P1	1.3264	0.9138	0.0242	0.1049
neg_526	PS(22:6/23:0)	neg	C51 H87 O10 N1 P1	1.1775	0.9531	0.0002	0.0046
neg_527	PS(25:0/22:6)	neg	C53 H91 O10 N1 P1	1.2879	0.9058	0.0263	0.1092
neg_528	PS(27:0/22:6)	neg	C55 H95 O10 N1 P1	1.7556	0.8750	0.0259	0.1092

neg_540	dMePE(14:0/14:0)	neg	C35 H69 O8 N1 P1	1.8515	0.8609	0.0003	0.0064
neg_542	dMePE(16:0/14:0)	neg	C37 H73 O8 N1 P1	1.2186	0.9339	0.0122	0.0670
neg_544	dMePE(16:1/14:0)	neg	C37 H71 O8 N1 P1	1.7018	0.8650	0.0048	0.0367
neg_547	dMePE(15:0/16:0)	neg	C38 H75 O8 N1 P1	1.8021	0.8599	0.0048	0.0367
neg_557	dMePE(16:0/18:1)	neg	C41 H79 O8 N1 P1	1.1191	0.9571	0.0031	0.0283
neg_561	dMePE(14:0/20:4)	neg	C41 H73 O8 N1 P1	1.6275	0.8916	0.0009	0.0143
neg_562	dMePE(14:0/20:5)	neg	C41 H71 O8 N1 P1	1.6224	0.8900	0.0006	0.0113
neg_573	dMePE(16:0/20:5)	neg	C43 H75 O8 N1 P1	1.4212	0.9337	0.0001	0.0046
neg_577	dMePE(14:0/22:6)	neg	C43 H73 O8 N1 P1	1.1901	0.9475	0.0008	0.0132
neg_586	dMePE(16:0/22:6)	neg	C45 H77 O8 N1 P1	1.0950	0.9634	0.0000	0.0015
neg_598	dMePE(20:5/20:4)	neg	C47 H75 O8 N1 P1	0.9896	0.9496	0.0245	0.1054
neg_601	dMePE(20:5/22:6)	neg	C49 H75 O8 N1 P1	1.1175	0.9496	0.0041	0.0329

1.7 Table S7. Higher abundant levels of lipid species in simple-packaged (SP) pike eels in comparison with vacuum-packaged (VP) pike eels via LC-MS/MS analysis.

ID	Lipids	Mode	Formula	VIP	FC	*P* value	FDR
pos_16	BisMePA(18:0/20:5)	pos	C43 H79 O8 N1 P1	1.0659	1.0851	0.0001	0.0024
pos_29	CL(20:5/20:4/22:4/24:1)	pos	C95 H159 O17 P2	1.4678	1.2322	0.0002	0.0026
pos_36	Cer(d16:1/16:0)	pos	C32 H62 O2 N1	1.2487	1.1945	0.0015	0.0061
pos_54	Cer(d16:0/24:1)	pos	C40 H80 O3 N1	1.0659	1.1132	0.0007	0.0041
pos_59	Cer(d19:1/22:0)	pos	C41 H82 O3 N1	1.0921	1.1906	0.0253	0.0453
pos_62	Cer(d19:1/23:0)	pos	C42 H82 O2 N1	1.2093	1.2062	0.0114	0.0251
pos_75	Cer(m18:0/24:0)	pos	C42 H86 O2 N1	1.1589	1.1324	0.0003	0.0027
pos_99	ChE(16:0)	pos	C43 H80 O2 N1	1.7889	1.3135	0.0001	0.0022
pos_102	ChE(18:0)	pos	C45 H84 O2 N1	1.7419	1.3108	0.0001	0.0023
pos_103	ChE(18:1)	pos	C45 H82 O2 N1	2.1141	1.4211	0.0000	0.0010
pos_105	ChE(19:1)	pos	C46 H84 O2 N1	1.5627	1.2595	0.0005	0.0035
pos_106	ChE(20:1)	pos	C47 H86 O2 N1	1.8639	1.3943	0.0005	0.0034
pos_107	ChE(20:2)	pos	C47 H84 O2 N1	1.5282	1.2301	0.0005	0.0035
pos_110	ChE(20:5)	pos	C47 H75 O2	1.7581	1.3388	0.0008	0.0043
pos_111	ChE(22:1)	pos	C49 H90 O2 N1	1.8417	1.3788	0.0001	0.0023
pos_112	ChE(22:2)	pos	C49 H88 O2 N1	1.4481	1.2212	0.0006	0.0035
pos_113	ChE(22:4)	pos	C49 H84 O2 N1	1.7177	1.3079	0.0004	0.0031
pos_114	ChE(22:5)	pos	C49 H82 O2 N1	1.8289	1.3202	0.0001	0.0023
pos_115	ChE(22:6)	pos	C49 H77 O2	1.6430	1.2826	0.0007	0.0038
pos_116	ChE(24:1)	pos	C51 H94 O2 N1	1.3822	1.2216	0.0010	0.0048
pos_117	ChE(24:2)	pos	C51 H92 O2 N1	1.5822	1.3106	0.0005	0.0033

pos_118	ChE(30:5)	pos	C57 H98 O2 N1	0.9612	1.1067	0.0031	0.0097
pos_119	Co(Q10)	pos	C59 H91 O4	1.0550	1.1307	0.0126	0.0271
pos_213	DG(18:1/20:5)	pos	C41 H69 O5	1.0670	1.1510	0.0258	0.0460
pos_290	DG(33:0/18:1)	pos	C54 H108 O5 N1	1.1598	1.1318	0.0048	0.0131
pos_299	Hex1Cer(d16:1/16:0)	pos	C38 H74 O8 N1	1.1540	1.1505	0.0021	0.0077
pos_301	Hex1Cer(d16:1/22:0)	pos	C44 H86 O8 N1	0.9915	1.0899	0.0004	0.0029
pos_302	Hex1Cer(d18:1/22:0)	pos	C46 H90 O8 N1	1.2986	1.1536	0.0148	0.0302
pos_305	Hex1Cer(d17:1/24:1)	pos	C47 H88 O7 N1	1.0749	1.1458	0.0142	0.0293
pos_322	LPC(14:0e)	pos	C22 H49 O6 N1 P1	1.0781	1.1081	0.0016	0.0064
pos_326	LPC(16:0e)	pos	C24 H53 O6 N1 P1	1.1760	1.1079	0.0006	0.0036
pos_328	LPC(16:1e)	pos	C24 H51 O6 N1 P1	1.1041	1.1137	0.0012	0.0053
pos_329	LPC(16:2e)	pos	C24 H49 O6 N1 P1	1.1000	1.1631	0.0080	0.0194
pos_330	LPC(17:0)	pos	C25 H53 O7 N1 P1	1.1740	1.1306	0.0016	0.0064
pos_333	LPC(18:0e)	pos	C26 H57 O6 N1 P1	1.4129	1.1920	0.0005	0.0034
pos_335	LPC(18:1e)	pos	C26 H55 O6 N1 P1	1.2383	1.1234	0.0006	0.0035
pos_337	LPC(18:2e)	pos	C26 H53 O6 N1 P1	1.0980	1.1249	0.0060	0.0155
pos_341	LPC(19:0)	pos	C27 H57 O7 N1 P1	1.1694	1.1603	0.0033	0.0102
pos_343	LPC(20:0)	pos	C28 H59 O7 N1 P1	0.9994	1.1042	0.0042	0.0121
pos_344	LPC(20:0e)	pos	C28 H61 O6 N1 P1	1.4493	1.1894	0.0001	0.0024
pos_346	LPC(20:1e)	pos	C28 H59 O6 N1 P1	1.2198	1.1511	0.0005	0.0035
pos_350	LPC(20:3e)	pos	C28 H55 O6 N1 P1	1.2435	1.1989	0.0033	0.0101
pos_352	LPC(20:4e)	pos	C28 H53 O6 N1 P1	1.1913	1.1609	0.0012	0.0053
pos_355	LPC(22:0)	pos	C30 H63 O7 N1 P1	1.3982	1.2193	0.0010	0.0047

pos_362	LPC(23:0)	pos	C31 H65 O7 N1 P1	1.3661	1.2375	0.0042	0.0122
pos_363	LPC(23:1)	pos	C31 H63 O7 N1 P1	1.4193	1.2342	0.0003	0.0028
pos_364	LPC(24:0)	pos	C32 H67 O7 N1 P1	1.4700	1.2367	0.0018	0.0069
pos_366	LPC(24:2)	pos	C32 H63 O7 N1 P1	1.1468	1.1609	0.0017	0.0066
pos_367	LPC(26:1)	pos	C34 H69 O7 N1 P1	1.3210	1.1951	0.0026	0.0088
pos_368	LPC(28:0)	pos	C36 H75 O7 N1 P1	0.9583	1.0752	0.0004	0.0031
pos_369	LPC(28:1)	pos	C36 H73 O7 N1 P1	0.9028	1.0883	0.0083	0.0199
pos_370	LPC(30:0)	pos	C38 H78 O7 N1 P1 Na1	1.1064	1.1029	0.0002	0.0024
pos_371	LPC(30:1)	pos	C38 H76 O7 N1 P1 Na1	0.8939	1.0941	0.0125	0.0269
pos_373	LPC(32:0)	pos	C40 H82 O7 N1 P1 Na1	1.1635	1.1146	0.0002	0.0025
pos_374	LPC(32:1)	pos	C40 H80 O7 N1 P1 Na1	1.1827	1.1150	0.0001	0.0024
pos_375	LPC(33:1)	pos	C41 H82 O7 N1 P1 Na1	1.2936	1.1598	0.0014	0.0058
pos_376	LPC(34:1)	pos	C42 H84 O7 N1 P1 Na1	1.1907	1.1091	0.0001	0.0024
pos_394	MePC(8:0/11:2)	pos	C28 H52 O8 N1 P1 Na1	1.2862	1.2040	0.0121	0.0262
pos_396	MePC(8:0e/11:4)	pos	C28 H50 O7 N1 P1 Na1	1.1469	1.1790	0.0235	0.0429
pos_400	MePC(11:0/18:2)	pos	C40 H80 O8 N2 P1	1.5295	1.2773	0.0003	0.0028
pos_401	MePC(18:0e/11:4)	pos	C40 H78 O7 N2 P1	1.4179	1.2651	0.0016	0.0065
pos_402	MePC(12:0/18:2)	pos	C41 H82 O8 N2 P1	1.4962	1.3163	0.0011	0.0052
pos_405	MePC(11:0/20:2)	pos	C42 H84 O8 N2 P1	1.5468	1.2492	0.0004	0.0030
pos_406	MePC(11:0/20:3)	pos	C42 H82 O8 N2 P1	1.6363	1.3397	0.0004	0.0031
pos_407	MePC(11:0/20:4)	pos	C42 H80 O8 N2 P1	1.3141	1.2035	0.0004	0.0029
pos_408	MePC(20:0e/11:4)	pos	C42 H82 O7 N2 P1	1.8398	1.4502	0.0002	0.0026
pos_409	MePC(16:0/16:1)	pos	C41 H84 O8 N2 P1	1.0318	1.0785	0.0002	0.0026

pos_410	MePC(16:1/16:1)	pos	C43 H86 O8 N2 P1	1.5864	1.3555	0.0010	0.0048
pos_418	MePC(15:0/18:2)	pos	C42 H84 O8 N2 P1	1.5010	1.2291	0.0006	0.0036
pos_419	MePC(15:0/18:3)	pos	C42 H82 O8 N2 P1	1.6366	1.3390	0.0004	0.0031
pos_420	MePC(20:4e/13:0)	pos	C42 H82 O7 N2 P1	1.8391	1.5054	0.0007	0.0039
pos_421	MePC(11:0/22:5)	pos	C44 H82 O8 N2 P1	1.3529	1.2136	0.0010	0.0048
pos_424	MePC(16:1/18:2)	pos	C45 H88 O8 N2 P1	1.8620	1.8662	0.0233	0.0427
pos_437	MePC(17:1/18:2)	pos	C46 H90 O8 N2 P1	1.5374	1.3042	0.0004	0.0032
pos_438	MePC(15:0/20:5)	pos	C44 H82 O8 N2 P1	1.3529	1.2135	0.0010	0.0048
pos_440	MePC(18:1/18:2)	pos	C45 H84 O8 N1 P1 Na1	0.9987	1.0898	0.0039	0.0115
pos_442	MePC(16:0e/20:4)	pos	C45 H84 O7 N1 P1 Na1	3.7678	110.2739	0.0000	0.0000
pos_450	MePC(19:0/18:2)	pos	C46 H88 O8 N1 P1 Na1	0.9955	1.0814	0.0033	0.0101
pos_451	MePC(19:1/18:2)	pos	C48 H94 O8 N2 P1	1.6468	1.3055	0.0004	0.0032
pos_457	MePC(16:0e/22:6)	pos	C47 H84 O7 N1 P1 Na1	0.8828	1.1027	0.0255	0.0456
pos_463	MePC(17:0/22:6)	pos	C50 H92 O8 N2 P1	1.1594	1.1619	0.0043	0.0124
pos_470	MePC(20:4e/21:0)	pos	C50 H94 O7 N1 P1 Na1	1.1138	1.1176	0.0003	0.0029
pos_486	PC(6:0/14:3)	pos	C28 H51 O8 N1 P1	1.1018	1.1693	0.0163	0.0324
pos_487	PC(8:0e/13:0)	pos	C29 H61 O7 N1 P1	1.4415	1.1871	0.0253	0.0453
pos_489	PC(10:0e/11:3)	pos	C29 H55 O7 N1 P1	1.2110	1.1423	0.0009	0.0045
pos_495	PC(8:1e/15:0)	pos	C31 H63 O7 N1 P1	1.4193	1.2342	0.0003	0.0028
pos_496	PC(12:0e/11:4)	pos	C31 H57 O7 N1 P1	0.9616	1.0959	0.0106	0.0237
pos_497	PC(12:1e/11:4)	pos	C31 H55 O7 N1 P1	0.8837	1.0743	0.0095	0.0219
pos_498	PC(12:0e/12:0)	pos	C32 H67 O7 N1 P1	1.4700	1.2367	0.0018	0.0069
pos_500	PC(8:1e/18:2)	pos	C34 H65 O7 N1 P1	1.2522	1.2023	0.0035	0.0106

pos_501	PC(11:0/16:0)	pos	C35 H71 O8 N1 P1	1.2365	1.1850	0.0005	0.0032
pos_504	PC(14:0/14:0)	pos	C36 H72 O8 N1 P1 Na1	1.4471	1.2115	0.0016	0.0063
pos_506	PC(16:0/13:0)	pos	C37 H75 O8 N1 P1	1.6099	1.2255	0.0000	0.0006
pos_508	PC(8:0e/21:0)	pos	C37 H77 O7 N1 P1	1.2628	1.1473	0.0009	0.0045
pos_509	PC(16:1/13:0)	pos	C37 H73 O8 N1 P1	1.3231	1.2409	0.0016	0.0063
pos_510	PC(8:1e/21:0)	pos	C37 H75 O7 N1 P1	1.0078	1.0997	0.0037	0.0110
pos_513	PC(14:0e/16:0)	pos	C38 H79 O7 N1 P1	1.0002	1.0700	0.0001	0.0023
pos_514	PC(16:1/14:0)	pos	C38 H74 O8 N1 P1 Na1	1.2876	1.1569	0.0006	0.0035
pos_516	PC(12:0e/18:1)	pos	C38 H77 O7 N1 P1	1.1047	1.1075	0.0019	0.0071
pos_520	PC(14:1e/16:1)	pos	C38 H75 O7 N1 P1	1.1740	1.1410	0.0024	0.0082
pos_522	PC(8:0/22:3)	pos	C38 H71 O8 N1 P1	1.4471	1.2115	0.0016	0.0063
pos_523	PC(15:0/16:0)	pos	C39 H79 O8 N1 P1	0.9821	1.0852	0.0006	0.0035
pos_525	PC(8:0e/23:0)	pos	C39 H81 O7 N1 P1	1.0611	1.1145	0.0073	0.0181
pos_526	PC(15:0/16:1)	pos	C39 H77 O8 N1 P1	0.9898	1.1231	0.0237	0.0431
pos_528	PC(14:0e/17:1)	pos	C39 H79 O7 N1 P1	1.2282	1.0942	0.0000	0.0010
pos_533	PC(20:5/11:4)	pos	C39 H61 O8 N1 P1	1.4677	1.2127	0.0005	0.0032
pos_534	PC(16:0/16:0)	pos	C40 H81 O8 N1 P1	1.1065	1.1065	0.0001	0.0023
pos_536	PC(16:0/16:1)	pos	C40 H78 O8 N1 P1 Na1	1.0024	1.0924	0.0053	0.0141
pos_538	PC(14:0e/18:1)	pos	C40 H81 O7 N1 P1	0.9895	1.0850	0.0024	0.0084
pos_542	PC(16:1e/16:1)	pos	C40 H79 O7 N1 P1	0.9668	1.0735	0.0003	0.0027
pos_543	PC(10:0/22:3)	pos	C40 H75 O8 N1 P1	1.1673	1.1487	0.0045	0.0128
pos_544	PC(14:1e/18:2)	pos	C40 H77 O7 N1 P1	0.9828	1.1159	0.0473	0.0758
pos_550	PC(18:0/15:0)	pos	C41 H83 O8 N1 P1	1.0247	1.0868	0.0003	0.0028

pos_551	PC(16:0/17:0)	pos	C41 H83 O8 N1 P1	1.1261	1.0924	0.0001	0.0023
pos_554	PC(17:1/16:0)	pos	C41 H80 O8 N1 P1 Na1	1.3211	1.1436	0.0002	0.0026
pos_556	PC(16:0e/17:1)	pos	C41 H83 O7 N1 P1	1.0252	1.0788	0.0002	0.0026
pos_557	PC(15:0/18:2)	pos	C41 H79 O8 N1 P1	1.0541	1.0912	0.0008	0.0044
pos_558	PC(16:2e/17:0)	pos	C41 H81 O7 N1 P1	1.0853	1.1436	0.0485	0.0774
pos_559	PC(15:0/18:3)	pos	C41 H77 O8 N1 P1	1.0983	1.1574	0.0192	0.0361
pos_567	PC(18:0e/16:0)	pos	C42 H87 O7 N1 P1	1.0108	1.0906	0.0006	0.0035
pos_569	PC(16:1e/18:0)	pos	C42 H85 O7 N1 P1	1.0385	1.0714	0.0001	0.0024
pos_572	PC(16:0/18:2)	pos	C42 H80 O8 N1 P1 Na1	1.0950	1.1056	0.0027	0.0089
pos_573	PC(16:0e/18:2)	pos	C42 H83 O7 N1 P1	1.1910	1.1080	0.0001	0.0024
pos_574	PC(16:1e/18:1)	pos	C42 H83 O7 N1 P1	0.9412	1.0670	0.0008	0.0043
pos_576	PC(16:0/18:3)	pos	C42 H78 O8 N1 P1 Na1	1.1402	1.1127	0.0003	0.0029
pos_578	PC(16:1e/18:2)	pos	C42 H81 O7 N1 P1	0.9808	1.0775	0.0003	0.0028
pos_579	PC(16:1/18:3)	pos	C42 H77 O8 N1 P1	0.9292	1.1364	0.0251	0.0452
pos_581	PC(14:0e/20:4)	pos	C42 H79 O7 N1 P1	0.8951	1.0624	0.0008	0.0043
pos_583	PC(14:0/20:5)	pos	C42 H74 O8 N1 P1 Na1	0.9051	1.0962	0.0104	0.0234
pos_591	PC(18:0/17:0)	pos	C43 H87 O8 N1 P1	0.9473	1.0704	0.0008	0.0044
pos_595	PC(17:0/18:2)	pos	C43 H83 O8 N1 P1	1.0324	1.1310	0.0421	0.0687
pos_596	PC(16:2e/19:0)	pos	C43 H85 O7 N1 P1	1.3405	1.1119	0.0165	0.0327
pos_599	PC(16:2e/19:1)	pos	C43 H83 O7 N1 P1	1.0589	1.0946	0.0004	0.0032
pos_602	PC(20:4e/15:0)	pos	C43 H81 O7 N1 P1	0.9707	1.0867	0.0014	0.0058
pos_605	PC(20:0/16:0)	pos	C44 H89 O8 N1 P1	1.3234	1.2046	0.0030	0.0095
pos_606	PC(18:0/18:0)	pos	C44 H89 O8 N1 P1	1.1183	1.1363	0.0144	0.0295

pos_607	PC(18:0/18:1)	pos	C44 H87 O8 N1 P1	1.1332	1.1331	0.0045	0.0128
pos_608	PC(16:0/20:1)	pos	C44 H87 O8 N1 P1	1.3833	1.1710	0.0165	0.0327
pos_609	PC(18:0e/18:1)	pos	C44 H89 O7 N1 P1	1.1295	1.1124	0.0007	0.0038
pos_610	PC(18:1/18:1)	pos	C44 H84 O8 N1 P1 Na1	1.0188	1.0755	0.0007	0.0041
pos_612	PC(18:0e/18:2)	pos	C44 H87 O7 N1 P1	1.1029	1.0912	0.0005	0.0032
pos_613	PC(16:0e/20:2)	pos	C44 H86 O7 N1 P1 Na1	0.9181	1.0748	0.0003	0.0028
pos_614	PC(18:1/18:2)	pos	C44 H83 O8 N1 P1	1.5661	1.3010	0.0026	0.0088
pos_615	PC(16:0/20:3)	pos	C44 H83 O8 N1 P1	1.6241	1.2868	0.0002	0.0024
pos_616	PC(18:3e/18:0)	pos	C44 H85 O7 N1 P1	0.9077	1.0761	0.0010	0.0047
pos_617	PC(18:1e/18:2)	pos	C44 H85 O7 N1 P1	1.0835	1.0897	0.0002	0.0024
pos_619	PC(18:4/18:0)	pos	C44 H81 O8 N1 P1	1.0658	1.0870	0.0032	0.0099
pos_620	PC(16:0e/20:4)	pos	C44 H83 O7 N1 P1	1.2538	1.1514	0.0011	0.0052
pos_621	PC(16:2e/20:2)	pos	C44 H82 O7 N1 P1 Na1	1.0677	1.0879	0.0003	0.0029
pos_622	PC(16:1/20:4)	pos	C44 H79 O8 N1 P1	1.4743	1.2493	0.0010	0.0048
pos_623	PC(16:0/20:5)	pos	C44 H78 O8 N1 P1 Na1	1.0881	1.0943	0.0022	0.0077
pos_624	PC(16:1e/20:4)	pos	C44 H81 O7 N1 P1	1.9209	1.5157	0.0001	0.0023
pos_625	PC(16:2e/20:3)	pos	C44 H80 O7 N1 P1 Na1	0.9983	1.0842	0.0016	0.0065
pos_628	PC(16:1/20:5)	pos	C44 H77 O8 N1 P1	1.5244	1.2935	0.0149	0.0303
pos_629	PC(14:0/22:6)	pos	C44 H76 O8 N1 P1 Na1	1.0064	1.0963	0.0037	0.0111
pos_636	PC(16:0/21:0)	pos	C45 H91 O8 N1 P1	1.0687	1.1099	0.0120	0.0262
pos_639	PC(19:1/18:2)	pos	C45 H85 O8 N1 P1	1.0198	1.0789	0.0003	0.0027
pos_642	PC(17:0/20:4)	pos	C45 H83 O8 N1 P1	1.7642	1.4424	0.0062	0.0158
pos_643	PC(19:1/18:3)	pos	C45 H83 O8 N1 P1	1.2311	1.1243	0.0002	0.0024

pos_646	PC(17:1/20:4)	pos	C45 H81 O8 N1 P1	1.3557	1.2687	0.0071	0.0176
pos_648	PC(17:0/20:5)	pos	C45 H80 O8 N1 P1 Na1	1.1316	1.1266	0.0006	0.0038
pos_650	PC(15:0/22:6)	pos	C45 H79 O8 N1 P1	1.0725	1.1790	0.0097	0.0223
pos_655	PC(20:0e/18:1)	pos	C46 H93 O7 N1 P1	0.9638	1.0903	0.0014	0.0059
pos_659	PC(20:1/18:2)	pos	C46 H87 O8 N1 P1	1.6698	1.5103	0.0102	0.0232
pos_662	PC(16:0/22:4)	pos	C46 H84 O8 N1 P1 Na1	1.1790	1.1188	0.0004	0.0031
pos_663	PC(16:0e/22:4)	pos	C46 H87 O7 N1 P1	1.1631	1.1013	0.0001	0.0024
pos_664	PC(18:2e/20:2)	pos	C46 H86 O7 N1 P1 Na1	1.1715	1.1215	0.0012	0.0053
pos_665	PC(18:0e/20:4)	pos	C46 H87 O7 N1 P1	1.0437	1.0986	0.0008	0.0043
pos_666	PC(18:1/20:4)	pos	C46 H83 O8 N1 P1	1.4464	1.2201	0.0025	0.0085
pos_668	PC(18:0/20:5)	pos	C46 H82 O8 N1 P1 Na1	1.1485	1.1079	0.0003	0.0028
pos_669	PC(16:2e/22:3)	pos	C46 H84 O7 N1 P1 Na1	1.0225	1.0880	0.0012	0.0055
pos_670	PC(18:2e/20:3)	pos	C46 H84 O7 N1 P1 Na1	1.2231	1.1329	0.0007	0.0038
pos_671	PC(16:0e/22:5)	pos	C46 H84 O7 N1 P1 Na1	0.9763	1.1070	0.0123	0.0266
pos_673	PC(16:0/22:6)	pos	C46 H81 O8 N1 P1	1.0837	1.1799	0.0370	0.0621
pos_676	PC(16:2e/22:4)	pos	C46 H82 O7 N1 P1 Na1	1.2057	1.1304	0.0005	0.0034
pos_689	PC(17:0/22:3)	pos	C47 H89 O8 N1 P1	1.0955	1.0947	0.0003	0.0027
pos_690	PC(19:1/20:2)	pos	C47 H89 O8 N1 P1	1.0768	1.1078	0.0006	0.0038
pos_691	PC(19:0/20:4)	pos	C47 H87 O8 N1 P1	1.0164	1.0800	0.0003	0.0028
pos_692	PC(19:1/20:3)	pos	C47 H87 O8 N1 P1	1.3225	1.1434	0.0001	0.0024
pos_695	PC(17:1/22:4)	pos	C47 H84 O8 N1 P1 Na1	1.7696	1.1716	0.0013	0.0056
pos_698	PC(17:0/22:6)	pos	C47 H83 O8 N1 P1	1.4075	1.2936	0.0014	0.0060
pos_709	PC(20:0e/20:4)	pos	C48 H91 O7 N1 P1	1.1959	1.1183	0.0002	0.0026

pos_710	PC(20:2e/20:2)	pos	C48 H90 O7 N1 P1 Na1	1.3516	1.1748	0.0003	0.0027
pos_711	PC(20:1/20:4)	pos	C48 H87 O8 N1 P1	1.5324	1.2648	0.0005	0.0034
pos_714	PC(18:3e/22:2)	pos	C48 H88 O7 N1 P1 Na1	1.0674	1.1233	0.0010	0.0048
pos_717	PC(18:0/22:6)	pos	C48 H85 O8 N1 P1	1.7016	1.3686	0.0010	0.0046
pos_718	PC(18:1/22:5)	pos	C48 H85 O8 N1 P1	0.8902	1.0741	0.0026	0.0088
pos_719	PC(18:0e/22:6)	pos	C48 H87 O7 N1 P1	1.0161	1.0869	0.0007	0.0039
pos_720	PC(18:2e/22:4)	pos	C48 H86 O7 N1 P1 Na1	1.3081	1.1395	0.0001	0.0024
pos_721	PC(18:1/22:6)	pos	C48 H83 O8 N1 P1	1.8297	1.4277	0.0002	0.0025
pos_722	PC(18:3e/22:4)	pos	C48 H84 O7 N1 P1 Na1	1.0361	1.0923	0.0009	0.0045
pos_724	PC(18:2/22:6)	pos	C48 H81 O8 N1 P1	1.2369	1.2240	0.0084	0.0201
pos_728	PC(18:3e/22:5)	pos	C48 H82 O7 N1 P1 Na1	1.0163	1.1197	0.0088	0.0207
pos_733	PC(17:1/24:2)	pos	C49 H93 O8 N1 P1	1.3278	1.1655	0.0151	0.0305
pos_745	PC(20:0/22:4)	pos	C50 H93 O8 N1 P1	1.1474	1.1300	0.0005	0.0033
pos_747	PC(20:0/22:5)	pos	C50 H91 O8 N1 P1	1.1865	1.1089	0.0002	0.0024
pos_748	PC(31:1/11:4)	pos	C50 H91 O8 N1 P1	1.1135	1.1276	0.0030	0.0096
pos_751	PC(20:0e/22:6)	pos	C50 H91 O7 N1 P1	1.0891	1.1362	0.0055	0.0145
pos_752	PC(20:2e/22:4)	pos	C50 H90 O7 N1 P1 Na1	1.1842	1.1331	0.0001	0.0024
pos_760	PC(20:3e/22:6)	pos	C50 H85 O7 N1 P1	1.3073	1.1392	0.0001	0.0024
pos_761	PC(22:6/21:0)	pos	C51 H91 O8 N1 P1	1.0881	1.1292	0.0042	0.0121
pos_762	PC(22:6/21:1)	pos	C51 H89 O8 N1 P1	1.4228	1.2500	0.0027	0.0089
pos_766	PC(24:0/20:4)	pos	C52 H97 O8 N1 P1	2.3368	2.0186	0.0077	0.0189
pos_768	PC(20:3e/24:2)	pos	C52 H97 O7 N1 P1	1.0613	1.1467	0.0063	0.0160
pos_769	PC(22:0/22:6)	pos	C52 H93 O8 N1 P1	1.5667	1.4193	0.0442	0.0712

pos_770	PC(20:4e/24:2)	pos	C52 H95 O7 N1 P1	1.2387	1.1733	0.0044	0.0127
pos_771	PC(22:1/22:6)	pos	C52 H91 O8 N1 P1	1.1474	1.1291	0.0027	0.0090
pos_774	PC(24:1/22:6)	pos	C54 H95 O8 N1 P1	1.2408	1.1458	0.0009	0.0045
pos_788	PE(16:1e/18:1)	pos	C39 H76 O7 N1 P1 Na1	1.0532	1.1244	0.0137	0.0288
pos_798	PE(18:0/18:1)	pos	C41 H80 O8 N1 P1 Na1	1.0875	1.0872	0.0001	0.0023
pos_812	PE(18:0/20:4)	pos	C43 H79 O8 N1 P1	1.0659	1.0851	0.0001	0.0024
pos_872	PG(16:1/14:0)	pos	C36 H70 O10 N0 P1	1.4310	1.2746	0.0027	0.0089
pos_911	PS(18:0/18:1)	pos	C42 H81 O10 N1 P1	1.9664	1.3601	0.0000	0.0008
pos_914	PS(18:0/22:5)	pos	C46 H81 O10 N1 P1	1.6244	1.2534	0.0000	0.0017
pos_915	PS(18:0/22:6)	pos	C46 H79 O10 N1 P1	1.3048	1.1662	0.0027	0.0089
pos_920	SM(d18:1/12:0)	pos	C35 H72 O6 N2 P1	1.1584	1.1192	0.0005	0.0034
pos_921	SM(d18:1/13:0)	pos	C36 H74 O6 N2 P1	1.1098	1.1155	0.0009	0.0045
pos_923	SM(d16:1/16:0)	pos	C37 H75 O6 N2 P1 Na1	1.0745	1.0909	0.0017	0.0067
pos_924	SM(d14:0/18:1)	pos	C37 H76 O6 N2 P1	1.1369	1.0889	0.0003	0.0028
pos_925	SM(d16:1/16:1)	pos	C37 H74 O6 N2 P1	1.2370	1.1407	0.0001	0.0024
pos_926	SM(d14:0/18:4)	pos	C37 H70 O6 N2 P1	1.1941	1.1623	0.0020	0.0074
pos_928	SM(d17:1/16:0)	pos	C38 H78 O6 N2 P1	1.0307	1.0856	0.0019	0.0071
pos_930	SM(d16:1/17:1)	pos	C38 H76 O6 N2 P1	1.0535	1.1391	0.0123	0.0265
pos_931	SM(d18:2/15:1)	pos	C38 H74 O6 N2 P1	1.1170	1.1484	0.0062	0.0158
pos_934	SM(d18:2/16:0)	pos	C39 H78 O6 N2 P1	1.1975	1.1147	0.0001	0.0024
pos_936	SM(d16:1/18:3)	pos	C39 H74 O6 N2 P1	1.0745	1.0909	0.0017	0.0067
pos_939	SM(d18:2/17:1)	pos	C40 H78 O6 N2 P1	1.0704	1.0960	0.0015	0.0061
pos_940	SM(d14:0/21:4)	pos	C40 H76 O6 N2 P1	1.1087	1.1073	0.0014	0.0060

pos_941	SM(d18:0/18:0)	pos	C41 H86 O6 N2 P1	1.1789	1.1227	0.0162	0.0324
pos_944	SM(d18:2/18:1)	pos	C41 H80 O6 N2 P1	1.2300	1.1563	0.0026	0.0088
pos_945	SM(d18:1/18:3)	pos	C41 H78 O6 N2 P1	1.0537	1.0780	0.0004	0.0031
pos_946	SM(d18:2/18:3)	pos	C41 H76 O6 N2 P1	1.4871	1.2150	0.0006	0.0035
pos_948	SM(d19:1/18:1)	pos	C42 H84 O6 N2 P1	1.0230	1.1117	0.0222	0.0408
pos_949	SM(d18:2/19:1)	pos	C42 H82 O6 N2 P1	1.2070	1.1037	0.0001	0.0024
pos_950	SM(d14:0/23:4)	pos	C42 H80 O6 N2 P1	1.2296	1.1404	0.0018	0.0069
pos_951	SM(d16:1/21:3)	pos	C42 H80 O6 N2 P1	1.4276	1.1821	0.0002	0.0024
pos_952	SM(d14:0/23:6)	pos	C42 H76 O6 N2 P1	1.0865	1.1119	0.0019	0.0070
pos_953	SM(d20:0/18:0)	pos	C43 H90 O6 N2 P1	1.3577	1.1722	0.0271	0.0480
pos_954	SM(d20:0/18:1)	pos	C43 H88 O6 N2 P1	1.5606	1.2053	0.0009	0.0044
pos_955	SM(d16:1/22:0)	pos	C43 H87 O6 N2 P1 Na1	1.2007	1.1030	0.0001	0.0023
pos_957	SM(d18:1/20:2)	pos	C43 H84 O6 N2 P1	1.0543	1.1078	0.0081	0.0196
pos_958	SM(d16:1/22:2)	pos	C43 H84 O6 N2 P1	1.1526	1.1122	0.0003	0.0029
pos_962	SM(d18:1/21:0)	pos	C44 H90 O6 N2 P1	0.9698	1.0740	0.0013	0.0055
pos_963	SM(d18:0/21:2)	pos	C44 H88 O6 N2 P1	1.0104	1.0844	0.0031	0.0098
pos_965	SM(d18:1/21:2)	pos	C44 H86 O6 N2 P1	1.0032	1.0953	0.0164	0.0326
pos_966	SM(d18:0/21:3)	pos	C44 H86 O6 N2 P1	1.2254	1.1095	0.0000	0.0020
pos_968	SM(d18:1/21:5)	pos	C44 H80 O6 N2 P1	1.4273	1.1652	0.0001	0.0023
pos_971	SM(d16:1/24:1)	pos	C45 H89 O6 N2 P1 Na1	1.1203	1.0883	0.0003	0.0027
pos_973	SM(d18:1/22:2)	pos	C45 H88 O6 N2 P1	0.9929	1.0790	0.0013	0.0056
pos_974	SM(d18:1/22:3)	pos	C45 H86 O6 N2 P1	1.1645	1.1372	0.0058	0.0152
pos_975	SM(d16:1/24:3)	pos	C45 H86 O6 N2 P1	1.2017	1.1031	0.0001	0.0023

pos_977	SM(d16:1/24:4)	pos	C45 H84 O6 N2 P1	1.0055	1.0878	0.0017	0.0066
pos_978	SM(d18:1/22:5)	pos	C45 H82 O6 N2 P1	2.0911	1.9676	0.0252	0.0453
pos_979	SM(d20:1/20:5)	pos	C45 H82 O6 N2 P1	1.6940	1.2855	0.0000	0.0015
pos_980	SM(d18:1/22:6)	pos	C45 H80 O6 N2 P1	1.3578	1.2078	0.0024	0.0082
pos_982	SM(d18:1/23:1)	pos	C46 H92 O6 N2 P1	1.0533	1.0863	0.0014	0.0058
pos_983	SM(d18:1/23:2)	pos	C46 H90 O6 N2 P1	1.0484	1.1044	0.0036	0.0108
pos_984	SM(d20:0/21:3)	pos	C46 H90 O6 N2 P1	1.1213	1.0951	0.0003	0.0028
pos_985	SM(d18:1/23:3)	pos	C46 H88 O6 N2 P1	1.1455	1.1168	0.0056	0.0148
pos_986	SM(d18:1/23:4)	pos	C46 H86 O6 N2 P1	1.4297	1.1843	0.0002	0.0025
pos_987	SM(d18:1/23:5)	pos	C46 H84 O6 N2 P1	1.7090	1.2502	0.0000	0.0003
pos_989	SM(d18:1/24:1)	pos	C47 H94 O6 N2 P1	1.3069	1.1651	0.0057	0.0150
pos_990	SM(d18:1/24:2)	pos	C47 H92 O6 N2 P1	1.0562	1.0856	0.0006	0.0036
pos_991	SM(d18:1/24:3)	pos	C47 H90 O6 N2 P1	1.2318	1.1347	0.0008	0.0043
pos_992	SM(d19:1/23:3)	pos	C47 H90 O6 N2 P1	0.9751	1.0700	0.0001	0.0024
pos_993	SM(d18:2/24:3)	pos	C47 H88 O6 N2 P1	1.1136	1.0874	0.0003	0.0028
pos_994	SM(d18:1/24:5)	pos	C47 H86 O6 N2 P1	0.9729	1.0875	0.0021	0.0075
pos_995	SM(d19:0/24:2)	pos	C48 H96 O6 N2 P1	1.0724	1.1669	0.0224	0.0411
pos_997	SM(d19:0/24:4)	pos	C48 H92 O6 N2 P1	1.0360	1.0840	0.0014	0.0058
pos_998	SM(d19:0/24:5)	pos	C48 H90 O6 N2 P1	0.9939	1.1055	0.0106	0.0237
pos_999	SM(d19:0/24:6)	pos	C48 H88 O6 N2 P1	1.0126	1.1448	0.0395	0.0654
pos_1000	SM(d19:1/24:6)	pos	C48 H86 O6 N2 P1	1.6526	1.2434	0.0000	0.0020
pos_1001	SM(d20:0/24:2)	pos	C49 H98 O6 N2 P1	1.4094	1.1293	0.0022	0.0077
pos_1002	SM(d20:0/24:3)	pos	C49 H96 O6 N2 P1	1.3556	1.1538	0.0093	0.0214

pos_1005	SM(d20:0/24:5)	pos	C49 H92 O6 N2 P1	1.2611	1.1604	0.0080	0.0195
pos_1006	SM(d20:0/24:6)	pos	C49 H90 O6 N2 P1	1.2005	1.1225	0.0002	0.0024
pos_1007	SM(t18:0/14:0)	pos	C37 H78 O7 N2 P1	1.2514	1.1770	0.0021	0.0077
pos_1008	SM(t18:1/14:0)	pos	C37 H76 O7 N2 P1	1.3310	1.1970	0.0033	0.0101
pos_1009	SM(t18:0/16:0)	pos	C39 H82 O7 N2 P1	1.2591	1.1612	0.0012	0.0053
pos_1010	SM(t18:1/21:5)	pos	C44 H80 O7 N2 P1	1.2460	1.2458	0.0387	0.0644
pos_1011	SM(t18:1/22:0)	pos	C45 H92 O7 N2 P1	1.1512	1.1385	0.0031	0.0098
pos_1012	SM(t18:1/22:1)	pos	C45 H90 O7 N2 P1	1.5371	1.3037	0.0002	0.0026
pos_1013	SM(t18:1/22:2)	pos	C45 H88 O7 N2 P1	2.7685	5.8097	0.0006	0.0035
pos_1014	SM(t18:1/22:3)	pos	C45 H86 O7 N2 P1	0.9706	1.0806	0.0028	0.0092
pos_1015	SM(t18:1/22:6)	pos	C45 H79 O7 N2 P1 Li1	1.9247	1.4552	0.0000	0.0021
pos_1017	SM(t18:1/24:0)	pos	C47 H96 O7 N2 P1	0.8792	1.1053	0.0365	0.0614
pos_1027	SPHP(d20:0)	pos	C20 H43 O4 N1 P1	1.0150	1.0992	0.0015	0.0061
pos_1028	StE(22:3)	pos	C51 H88 O2 N1	1.4476	1.2451	0.0025	0.0085
pos_1029	StE(22:4)	pos	C51 H86 O2 N1	1.6211	1.2802	0.0007	0.0038
pos_1067	TG(14:0/10:2/14:0)	pos	C41 H74 O6 Li1	1.7904	1.3122	0.0002	0.0025
pos_1083	TG(18:3/10:3/11:4)	pos	C42 H60 O6 Li1	1.1797	1.2079	0.0202	0.0378
pos_1095	TG(16:0/6:0/18:2)	pos	C43 H78 O6 Li1	1.6430	1.2817	0.0007	0.0038
pos_1105	TG(20:3/10:3/11:4)	pos	C44 H64 O6 Li1	1.2303	1.2002	0.0119	0.0260
pos_1106	TG(20:4/10:4/11:3)	pos	C44 H62 O6 Li1	1.4310	1.2746	0.0027	0.0089
pos_1119	TG(20:4e/6:0/16:0)	pos	C45 H81 O5	1.4371	1.1421	0.0046	0.0130
pos_1178	TG(16:0e/14:0/16:0)	pos	C49 H100 O5 N1	1.4566	1.2142	0.0006	0.0035
pos_1179	TG(16:0/14:0/16:1)	pos	C49 H92 O6 Na1	0.9637	1.0812	0.0038	0.0113

pos_1198	TG(16:0/14:0/17:1)	pos	C50 H94 O6 Na1	1.0864	1.1045	0.0030	0.0095
pos_1200	TG(16:1/14:0/17:1)	pos	C50 H92 O6 Na1	1.0668	1.1219	0.0050	0.0135
pos_1210	TG(16:0/11:3/20:2)	pos	C50 H86 O6 Li1	2.1702	1.3409	0.0000	0.0000
pos_1218	TG(18:0e/14:0/16:0)	pos	C51 H104 O5 N1	1.3639	1.1638	0.0002	0.0025
pos_1220	TG(16:0/16:0/16:1)	pos	C51 H96 O6 Na1	1.1052	1.0995	0.0008	0.0041
pos_1222	TG(16:1/14:0/18:1)	pos	C51 H94 O6 Na1	0.9825	1.1061	0.0269	0.0476
pos_1224	TG(16:0e/16:1/16:1)	pos	C51 H100 O5 N1	0.9826	1.1449	0.0368	0.0618
pos_1246	TG(16:0/16:0/17:0)	pos	C52 H104 O6 N1	1.3460	1.1749	0.0006	0.0038
pos_1249	TG(15:0/16:0/18:1)	pos	C52 H98 O6 Na1	1.0283	1.0872	0.0038	0.0114
pos_1252	TG(16:0/16:1/17:1)	pos	C52 H96 O6 Na1	1.0310	1.0922	0.0041	0.0120
pos_1254	TG(16:1/16:1/17:1)	pos	C52 H94 O6 Na1	0.9885	1.1211	0.0164	0.0326
pos_1267	TG(11:0/18:1/20:5)	pos	C52 H88 O6 Li1	1.9083	1.2603	0.0000	0.0002
pos_1281	TG(16:1e/16:0/18:1)	pos	C53 H104 O5 N1	1.3785	1.1839	0.0018	0.0070
pos_1282	TG(18:1e/16:0/16:1)	pos	C53 H104 O5 N1	1.2697	1.1749	0.0028	0.0092
pos_1294	TG(18:4/16:0/16:1)	pos	C53 H93 O6	0.9492	1.0849	0.0083	0.0199
pos_1313	TG(16:0/17:1/18:1)	pos	C54 H100 O6 Na1	0.9843	1.0807	0.0025	0.0084
pos_1315	TG(16:1/17:1/18:1)	pos	C54 H98 O6 Na1	1.0166	1.1107	0.0063	0.0160
pos_1319	TG(16:1/17:1/18:3)	pos	C54 H94 O6 Li1	1.3169	1.1974	0.0067	0.0168
pos_1333	TG(18:0/16:0/18:0)	pos	C55 H110 O6 N1	1.1397	1.1312	0.0043	0.0124
pos_1334	TG(18:0e/16:0/18:0)	pos	C55 H112 O5 N1	1.2790	1.1652	0.0012	0.0054
pos_1339	TG(18:0e/16:0/18:1)	pos	C55 H107 O5	1.0334	1.0892	0.0002	0.0026
pos_1343	TG(18:1e/16:0/18:1)	pos	C55 H108 O5 N1	1.3300	1.1433	0.0002	0.0024
pos_1348	TG(16:1e/18:1/18:1)	pos	C55 H106 O5 N1	1.0293	1.1332	0.0207	0.0385

pos_1350	TG(16:0/16:0/20:4)	pos	C55 H98 O6 Na1	1.0193	1.1469	0.0281	0.0495
pos_1352	TG(16:0e/16:0/20:4)	pos	C55 H104 O5 N1	1.1001	1.1147	0.0031	0.0098
pos_1353	TG(16:0/16:1/20:4)	pos	C55 H96 O6 Na1	0.9573	1.1082	0.0291	0.0509
pos_1367	TG(18:0/17:0/18:0)	pos	C56 H112 O6 N1	0.8637	1.0822	0.0447	0.0720
pos_1381	TG(18:1/17:1/18:3)	pos	C56 H98 O6 Li1	1.2011	1.1616	0.0003	0.0027
pos_1410	TG(18:0e/18:0/18:1)	pos	C57 H111 O5	0.9450	1.0838	0.0013	0.0055
pos_1412	TG(16:0e/18:1/20:1)	pos	C57 H112 O5 N1	0.9235	1.0664	0.0003	0.0029
pos_1415	TG(18:1e/18:1/18:1)	pos	C57 H110 O5 N1	1.0171	1.1084	0.0021	0.0077
pos_1495	TG(18:0e/16:0/22:1)	pos	C59 H115 O5	0.8875	1.0819	0.0025	0.0085
pos_1499	TG(16:0e/18:1/22:1)	pos	C59 H116 O5 N1	1.0434	1.0735	0.0000	0.0009
pos_1622	TG(20:0/16:0/24:0)	pos	C63 H126 O6 N1	1.1742	1.1636	0.0439	0.0710
pos_1636	TG(16:1/22:6/22:6)	pos	C63 H97 O6	1.7415	1.2910	0.0007	0.0040
pos_1662	TG(20:1/18:1/22:6)	pos	C63 H106 O6 Na1	0.9732	1.0829	0.0015	0.0061
pos_1669	TG(22:5/17:1/22:6)	pos	C64 H100 O6 Na1	0.9655	1.0998	0.0085	0.0202
pos_1670	TG(17:0/22:6/22:6)	pos	C64 H100 O6 Na1	1.1298	1.1252	0.0009	0.0045
pos_1695	TG(18:1/22:5/22:5)	pos	C65 H105 O6	0.9738	1.0829	0.0015	0.0061
pos_1728	TG(15:0/24:1/24:1)	pos	C66 H124 O6 Li1	1.8987	1.3831	0.0004	0.0031
pos_1737	TG(24:1/17:1/22:4)	pos	C66 H120 O6 N1	0.9197	1.1027	0.0358	0.0605
pos_1758	TG(20:2/22:6/22:6)	pos	C67 H102 O6 Na1	1.0076	1.1106	0.0066	0.0166
pos_1760	TG(20:3/22:6/22:6)	pos	C67 H101 O6	0.9908	1.0805	0.0002	0.0025
pos_1935	ZyE(20:4)	pos	C47 H75 O2	1.7581	1.3389	0.0008	0.0043
pos_1936	ZyE(20:5)	pos	C47 H73 O2	1.7835	1.3096	0.0002	0.0025
pos_1937	ZyE(22:4)	pos	C49 H82 O2 N1	1.8289	1.3202	0.0001	0.0023

pos_1938	ZyE(22:5)	pos	C49 H77 O2	1.6426	1.2921	0.0007	0.0038
pos_1939	ZyE(22:6)	pos	C49 H75 O2	1.7540	1.2893	0.0002	0.0026
neg_9	CL(24:0/14:0/16:0/18:0)	neg	C81 H156 O17 P2	1.1892	1.1022	0.0010	0.0074
neg_14	CL(23:0/15:0/16:0/20:0)	neg	C83 H160 O17 P2	0.9361	1.0536	0.0011	0.0075
neg_35	CL(23:0/18:1/18:1/22:3)	neg	C90 H164 O17 P2	1.8562	1.3701	0.0067	0.0247
neg_49	Cer(d16:1/22:0+O)	neg	C38 H74 O4 N1	1.0952	1.0918	0.0032	0.0151
neg_51	Cer(d18:0/22:0)	neg	C41 H82 O5 N1	1.1098	1.1156	0.0096	0.0307
neg_58	Cer(d18:0/24:1)	neg	C43 H84 O5 N1	1.5320	1.1812	0.0001	0.0020
neg_66	Cer(t16:1/24:1)	neg	C41 H78 O6 N1	1.0394	1.0767	0.0050	0.0199
neg_81	GM3(m22:0/24:0)	neg	C69 H128 O20 N2	1.8407	1.3024	0.0003	0.0036
neg_133	LdMePE(16:0e)	neg	C23 H49 O6 N1 P1	1.0035	1.0711	0.0047	0.0192
neg_135	LdMePE(16:1e)	neg	C23 H47 O6 N1 P1	1.0233	1.1031	0.0419	0.0894
neg_136	LdMePE(17:0)	neg	C24 H49 O7 N1 P1	1.0740	1.1047	0.0110	0.0337
neg_139	LdMePE(18:0e)	neg	C25 H53 O6 N1 P1	1.3959	1.1393	0.0003	0.0037
neg_140	LdMePE(18:1e)	neg	C25 H51 O6 N1 P1	1.1976	1.1058	0.0039	0.0169
neg_143	LdMePE(19:0)	neg	C26 H53 O7 N1 P1	1.4150	1.1674	0.0024	0.0126
neg_146	LdMePE(20:0e)	neg	C27 H57 O6 N1 P1	1.7196	1.2399	0.0000	0.0000
neg_155	LdMePE(24:1)	neg	C31 H61 O7 N1 P1	1.2306	1.1786	0.0168	0.0456
neg_164	MGDG(14:1e/18:1)	neg	C43 H79 O11	1.0883	1.0983	0.0415	0.0891
neg_167	MGDG(18:1/16:0p)	neg	C45 H83 O11	1.1000	1.0847	0.0014	0.0091
neg_184	MLCL(14:2/15:0/15:0)	neg	C53 H98 O16 P2	0.9547	1.0843	0.0153	0.0428
neg_191	MLCL(14:2/18:0/18:0)	neg	C59 H110 O16 P2	1.1860	1.0955	0.0002	0.0032
neg_197	MLCL(14:2/20:0/20:0)	neg	C63 H118 O16 P2	1.1967	1.1467	0.0082	0.0279

neg_204	MLCL(14:2/22:0/22:0)	neg	C67 H126 O16 P2	1.3358	1.1994	0.0110	0.0337
neg_208	MLCL(14:2/24:1/24:1)	neg	C71 H130 O16 P2	1.4159	1.1827	0.0031	0.0148
neg_229	PC(17:0/18:1)	neg	C44 H85 O10 N1 P1	0.9398	1.0645	0.0053	0.0206
neg_232	PC(20:1e/16:0)	neg	C45 H89 O9 N1 P1	1.0455	1.0850	0.0021	0.0114
neg_233	PC(18:1e/18:1)	neg	C43 H83 O7 N1 P1	0.9104	1.0647	0.0038	0.0169
neg_251	PC(18:1e/22:5)	neg	C49 H87 O9 N1 P1	1.0700	1.1030	0.0137	0.0395
neg_253	PC(24:0/20:5)	neg	C53 H95 O10 N1 P1	1.3325	1.2078	0.0279	0.0673
neg_256	PC(24:0/22:6)	neg	C55 H97 O10 N1 P1	2.8364	5.2651	0.0088	0.0294
neg_333	PE(16:1/22:5)	neg	C43 H73 O8 N1 P1	1.0334	1.0952	0.0158	0.0439
neg_342	PE(19:0/20:4)	neg	C44 H79 O8 N1 P1	1.1225	1.1167	0.0214	0.0561
neg_349	PE(20:0/20:4)	neg	C45 H81 O8 N1 P1	1.0791	1.0877	0.0018	0.0103
neg_361	PE(20:0/22:5)	neg	C47 H83 O8 N1 P1	1.0140	1.0900	0.0106	0.0331
neg_403	PG(14:0/22:6)	neg	C42 H70 O10 N0 P1	1.3175	1.1504	0.0016	0.0096
neg_446	PI(18:0/22:4)	neg	C49 H86 O13 N0 P1	1.1121	1.0911	0.0236	0.0599
neg_468	PS(18:0/19:0)	neg	C43 H83 O10 N1 P1	0.9812	1.0482	0.0004	0.0041
neg_472	PS(18:0/20:0)	neg	C44 H85 O10 N1 P1	1.0960	1.0805	0.0010	0.0074
neg_475	PS(18:0/20:4)	neg	C44 H77 O10 N1 P1	1.8388	1.2403	0.0000	0.0011
neg_479	PS(18:0/20:5)	neg	C44 H75 O10 N1 P1	1.4103	1.1533	0.0008	0.0064
neg_488	PS(17:0/22:6)	neg	C45 H75 O10 N1 P1	1.1829	1.1196	0.0026	0.0136
neg_521	PS(20:3/22:6)	neg	C48 H75 O10 N1 P1	1.1872	1.1180	0.0130	0.0379
neg_540	dMePE(14:0/14:0)	neg	C35 H69 O8 N1 P1	1.7115	1.2273	0.0000	0.0005
neg_542	dMePE(16:0/14:0)	neg	C37 H73 O8 N1 P1	1.2221	1.1072	0.0011	0.0075
neg_544	dMePE(16:1/14:0)	neg	C37 H71 O8 N1 P1	1.3755	1.1615	0.0292	0.0691

neg_547	dMePE(15:0/16:0)	neg	C38 H75 O8 N1 P1	1.4279	1.1790	0.0038	0.0169
neg_551	dMePE(16:0/16:1)	neg	C39 H75 O8 N1 P1	1.0299	1.0647	0.0003	0.0033
neg_556	dMePE(17:1/16:0)	neg	C40 H77 O8 N1 P1	1.1380	1.0849	0.0004	0.0038
neg_557	dMePE(16:0/18:1)	neg	C41 H79 O8 N1 P1	0.9357	1.0512	0.0010	0.0073
neg_561	dMePE(14:0/20:4)	neg	C41 H73 O8 N1 P1	1.2439	1.1264	0.0016	0.0094
neg_583	dMePE(18:0e/20:4)	neg	C45 H83 O7 N1 P1	1.4000	1.1371	0.0021	0.0115
neg_604	phSM(d20:0/14:0)	neg	C40 H82 O9 N2 P1	1.1081	1.0914	0.0002	0.0032

1.8 Table S8. Lower abundant levels of lipid species in simple-packaged (SP) pike eels in comparison with vacuum-packaged (VP) pike eels via LC-MS/MS analysis.

ID	Lipids	Mode	Formula	VIP	FC	*P* value	FDR
pos_4	BisMePA(10:1e/18:1)	pos	C33 H63 O7 N0 P1 Na1	1.4957	0.8192	0.0000	0.0019
pos_28	BisMePA(30:0/15:0)	pos	C50 H103 O8 N1 P1	1.3715	0.8386	0.0031	0.0097
pos_120	Co(Q8)	pos	C49 H75 O4	1.7703	0.8843	0.0030	0.0096
pos_121	Co(Q9)	pos	C54 H83 O4	2.4884	0.7315	0.0000	0.0000
pos_122	DG(16:1/12:0)	pos	C31 H58 O5 Na1	1.1582	0.8780	0.0035	0.0106
pos_124	DG(16:1/14:0)	pos	C33 H66 O5 N1	1.0279	0.9125	0.0027	0.0089
pos_126	DG(18:4/12:0)	pos	C33 H56 O5 Na1	1.8980	0.5893	0.0361	0.0610
pos_133	DG(16:1e/16:0)	pos	C35 H68 O4 Na1	1.0087	0.9225	0.0058	0.0151
pos_134	DG(16:1/16:1)	pos	C35 H68 O5 N1	1.3711	0.8525	0.0022	0.0077
pos_135	DG(14:0/18:3)	pos	C35 H63 O5	1.1953	0.8701	0.0018	0.0070
pos_136	DG(18:4/14:0)	pos	C35 H64 O5 N1	1.1828	0.8672	0.0278	0.0491
pos_137	DG(12:0/20:5)	pos	C35 H58 O5 Na1	1.5159	0.7103	0.0330	0.0565
pos_140	DG(15:0/18:1)	pos	C36 H72 O5 N1	1.0227	0.9033	0.0051	0.0138
pos_141	DG(17:1/16:0)	pos	C36 H72 O5 N1	1.0423	0.9226	0.0001	0.0024
pos_142	DG(17:1/16:1)	pos	C36 H70 O5 N1	1.0997	0.8927	0.0087	0.0205
pos_143	DG(15:0/18:3)	pos	C36 H68 O5 N1	1.2384	0.8705	0.0049	0.0133
pos_144	DG(18:4/15:0)	pos	C36 H66 O5 N1	1.2883	0.8600	0.0001	0.0024
pos_149	DG(16:1/18:1)	pos	C37 H72 O5 N1	1.0847	0.9178	0.0012	0.0053
pos_150	DG(16:1/18:2)	pos	C37 H70 O5 N1	1.2850	0.8776	0.0004	0.0032
pos_151	DG(16:0/18:3)	pos	C37 H67 O5	1.6047	0.7333	0.0496	0.0788
pos_152	DG(16:1/18:3)	pos	C37 H68 O5 N1	1.2457	0.8756	0.0052	0.0140

pos_153	DG(18:4/16:0)	pos	C37 H65 O5	1.3397	0.8590	0.0004	0.0032
pos_154	DG(18:3e/16:1)	pos	C37 H67 O4	1.0954	0.8607	0.0168	0.0331
pos_155	DG(18:4/16:1)	pos	C37 H63 O5	1.2340	0.8653	0.0039	0.0114
pos_156	DG(14:0/20:5)	pos	C37 H63 O5	1.2893	0.8778	0.0001	0.0024
pos_157	DG(20:5/14:1)	pos	C37 H64 O5 N1	2.9372	0.2214	0.0004	0.0032
pos_158	DG(12:0/22:6)	pos	C37 H64 O5 N1	1.4147	0.8420	0.0002	0.0024
pos_162	DG(17:1/18:1)	pos	C38 H74 O5 N1	1.1823	0.9025	0.0001	0.0023
pos_165	DG(15:0/20:4)	pos	C38 H70 O5 N1	1.3861	0.8209	0.0049	0.0133
pos_167	DG(22:6/13:0)	pos	C38 H66 O5 N1	1.2044	0.8833	0.0008	0.0044
pos_171	DG(18:1/18:1)	pos	C39 H72 O5 Na1	1.1967	0.8982	0.0000	0.0019
pos_172	DG(18:0/18:2)	pos	C39 H76 O5 N1	1.0595	0.8894	0.0166	0.0328
pos_173	DG(18:1/18:2)	pos	C39 H74 O5 N1	1.2062	0.8968	0.0003	0.0027
pos_180	DG(16:0/20:5)	pos	C39 H70 O5 N1	1.3466	0.8790	0.0001	0.0024
pos_181	DG(18:3e/18:2)	pos	C39 H69 O4	0.9900	0.9282	0.0002	0.0026
pos_183	DG(22:5/14:1)	pos	C39 H64 O5 Na1	1.4098	0.8361	0.0002	0.0027
pos_184	DG(16:1/20:5)	pos	C39 H68 O5 N1	1.5123	0.8387	0.0002	0.0026
pos_185	DG(18:4/18:3)	pos	C39 H66 O5 N1	1.3926	0.8387	0.0004	0.0029
pos_186	DG(22:6/14:1)	pos	C39 H66 O5 N1	1.3446	0.8582	0.0005	0.0034
pos_187	DG(18:4/18:4)	pos	C39 H61 O5	1.4236	0.8146	0.0004	0.0029
pos_188	DG(22:6/14:2)	pos	C39 H64 O5 N1	1.1905	0.8518	0.0018	0.0069
pos_189	DG(22:6/14:3)	pos	C39 H62 O5 N1	1.5006	0.8476	0.0000	0.0008
pos_193	DG(17:1/20:2)	pos	C40 H76 O5 N1	1.2547	0.8535	0.0019	0.0071
pos_197	DG(17:1/20:4)	pos	C40 H72 O5 N1	1.5116	0.8242	0.0001	0.0024

pos_198	DG(15:0/22:5)	pos	C40 H69 O5	1.3443	0.8590	0.0002	0.0025
pos_199	DG(17:0/20:5)	pos	C40 H72 O5 N1	1.3804	0.8777	0.0084	0.0201
pos_200	DG(17:1/20:5)	pos	C40 H70 O5 N1	1.5295	0.8351	0.0000	0.0010
pos_201	DG(15:0/22:6)	pos	C40 H70 O5 N1	1.3102	0.8503	0.0043	0.0123
pos_203	DG(20:1/18:1)	pos	C41 H80 O5 N1	1.0550	0.9188	0.0004	0.0029
pos_205	DG(18:1/20:2)	pos	C41 H78 O5 N1	1.2452	0.8840	0.0002	0.0027
pos_206	DG(18:1/20:3)	pos	C41 H73 O5	1.4057	0.7636	0.0361	0.0610
pos_207	DG(16:0/22:4)	pos	C41 H76 O5 N1	1.1042	0.9124	0.0001	0.0024
pos_210	DG(18:0/20:5)	pos	C41 H74 O5 N1	1.1791	0.9060	0.0001	0.0023
pos_214	DG(16:1/22:5)	pos	C41 H69 O5	1.4094	0.8498	0.0012	0.0053
pos_215	DG(16:0/22:6)	pos	C41 H72 O5 N1	1.1651	0.9141	0.0002	0.0027
pos_217	DG(16:1/22:6)	pos	C41 H67 O5	1.4324	0.8456	0.0018	0.0069
pos_218	DG(18:3/20:5)	pos	C41 H65 O5	1.0648	0.8830	0.0082	0.0198
pos_219	DG(18:4/20:5)	pos	C41 H63 O5	1.2193	0.8609	0.0039	0.0115
pos_220	DG(17:1/22:1)	pos	C42 H82 O5 N1	0.9889	0.9146	0.0011	0.0050
pos_221	DG(17:1/22:2)	pos	C42 H80 O5 N1	1.1384	0.8581	0.0221	0.0408
pos_222	DG(19:0/20:3)	pos	C42 H80 O5 N1	1.1897	0.8645	0.0030	0.0097
pos_225	DG(17:0/22:5)	pos	C42 H76 O5 N1	1.4065	0.8609	0.0000	0.0017
pos_226	DG(17:1/22:4)	pos	C42 H73 O5	1.0682	0.9162	0.0001	0.0024
pos_227	DG(17:1/22:5)	pos	C42 H74 O5 N1	1.0380	0.9226	0.0002	0.0025
pos_228	DG(17:0/22:6)	pos	C42 H74 O5 N1	1.1705	0.8953	0.0016	0.0063
pos_229	DG(17:1/22:6)	pos	C42 H72 O5 N1	1.3395	0.8760	0.0002	0.0024
pos_231	DG(18:4/22:6)	pos	C43 H65 O5	1.4788	0.8368	0.0001	0.0023

pos_232	DG(18:1/22:1)	pos	C43 H84 O5 N1	1.0201	0.9204	0.0005	0.0034
pos_233	DG(18:1/22:2)	pos	C43 H82 O5 N1	1.0809	0.9021	0.0010	0.0047
pos_237	DG(18:1/22:4)	pos	C43 H78 O5 N1	1.3169	0.8765	0.0002	0.0027
pos_239	DG(18:0/22:5)	pos	C43 H78 O5 N1	1.0490	0.9205	0.0004	0.0031
pos_240	DG(18:1/22:5)	pos	C43 H73 O5	1.3108	0.8798	0.0003	0.0028
pos_241	DG(18:0/22:6)	pos	C43 H76 O5 N1	1.0293	0.9298	0.0003	0.0027
pos_242	DG(20:3/20:4)	pos	C43 H70 O5 Na1	1.4930	0.8285	0.0004	0.0032
pos_243	DG(22:5/18:2)	pos	C43 H71 O5	1.5143	0.8121	0.0004	0.0032
pos_244	DG(18:1/22:6)	pos	C43 H74 O5 N1	1.2645	0.8996	0.0003	0.0027
pos_245	DG(18:3/22:5)	pos	C43 H72 O5 N1	1.6312	0.7951	0.0001	0.0024
pos_246	DG(18:2/22:6)	pos	C43 H72 O5 N1	1.4073	0.8622	0.0002	0.0026
pos_248	DG(18:3/22:6)	pos	C43 H70 O5 N1	1.8284	0.8063	0.0023	0.0079
pos_249	DG(19:0/22:5)	pos	C44 H80 O5 N1	1.0157	0.9161	0.0002	0.0026
pos_250	DG(19:1/22:5)	pos	C44 H78 O5 N1	1.2154	0.8635	0.0046	0.0129
pos_252	DG(19:1/22:6)	pos	C44 H76 O5 N1	1.3246	0.8697	0.0003	0.0027
pos_254	DG(20:5/22:6)	pos	C45 H67 O5	1.3242	0.8774	0.0003	0.0027
pos_258	DG(20:1/22:4)	pos	C45 H82 O5 N1	1.0999	0.9013	0.0004	0.0031
pos_260	DG(20:0/22:5)	pos	C45 H82 O5 N1	1.2166	0.8714	0.0351	0.0597
pos_262	DG(20:0/22:6)	pos	C45 H80 O5 N1	1.1662	0.8984	0.0015	0.0061
pos_264	DG(20:1/22:6)	pos	C45 H78 O5 N1	1.2424	0.8926	0.0002	0.0027
pos_266	DG(20:2/22:6)	pos	C45 H76 O5 N1	1.3556	0.8668	0.0003	0.0028
pos_267	DG(22:5/20:4)	pos	C45 H74 O5 N1	1.2204	0.8667	0.0010	0.0048
pos_269	DG(22:5/21:1)	pos	C46 H82 O5 N1	1.2782	0.8561	0.0011	0.0049

pos_270	DG(22:6/21:0)	pos	C46 H82 O5 N1	1.2315	0.8691	0.0008	0.0042
pos_271	DG(22:6/21:1)	pos	C46 H80 O5 N1	1.4144	0.8268	0.0003	0.0028
pos_272	DG(22:5/22:5)	pos	C47 H73 O5	1.5146	0.8137	0.0003	0.0029
pos_274	DG(22:5/22:6)	pos	C47 H71 O5	1.4503	0.8488	0.0002	0.0026
pos_276	DG(22:1/22:4)	pos	C47 H86 O5 N1	1.0407	0.9041	0.0010	0.0048
pos_279	DG(22:0/22:6)	pos	C47 H84 O5 N1	1.2293	0.8683	0.0028	0.0092
pos_282	DG(22:4/22:4)	pos	C47 H80 O5 N1	1.1798	0.8750	0.0009	0.0046
pos_284	DG(22:4/22:5)	pos	C47 H78 O5 N1	1.3314	0.8601	0.0006	0.0036
pos_285	DG(22:6/23:1)	pos	C48 H84 O5 N1	1.2436	0.8659	0.0008	0.0043
pos_286	DG(24:1/22:5)	pos	C49 H88 O5 N1	1.1804	0.8826	0.0005	0.0034
pos_287	DG(24:1/22:6)	pos	C49 H86 O5 N1	1.2335	0.8841	0.0005	0.0034
pos_288	DG(24:2/22:6)	pos	C49 H84 O5 N1	1.5945	0.7948	0.0003	0.0027
pos_292	DG(37:0/16:0)	pos	C56 H110 O5 K1	1.0295	0.9012	0.0080	0.0195
pos_378	LPE(16:1)	pos	C21 H42 O7 N1 P1 Na1	1.9573	0.8345	0.0002	0.0024
pos_404	MePC(8:0/22:5)	pos	C39 H68 O8 N1 P1 Na1	2.2232	0.7728	0.0000	0.0001
pos_435	MePC(22:6/12:3)	pos	C43 H68 O8 N1 P1 Na1	1.9422	0.8190	0.0000	0.0002
pos_459	MePC(18:4/20:4)	pos	C47 H78 O8 N1 P1 Na1	1.1625	0.8743	0.0086	0.0204
pos_502	PC(11:0/16:1)	pos	C35 H69 O8 N1 P1	1.5763	0.8802	0.0187	0.0357
pos_505	PC(16:0/12:0)	pos	C36 H73 O8 N1 P1	1.3663	0.8589	0.0005	0.0033
pos_511	PC(11:0/18:2)	pos	C37 H71 O8 N1 P1	1.8830	0.8194	0.0001	0.0024
pos_512	PC(16:0/14:0)	pos	C38 H77 O8 N1 P1	0.9453	0.9151	0.0210	0.0390
pos_519	PC(16:1/14:1)	pos	C38 H73 O8 N1 P1	1.9908	0.8690	0.0056	0.0148
pos_530	PC(17:1/14:1)	pos	C39 H75 O8 N1 P1	1.5606	0.8641	0.0001	0.0024

pos_565	PC(11:0/22:6)	pos	C41 H71 O8 N1 P1	1.7015	0.8407	0.0000	0.0002
pos_779	PE(16:0/16:1)	pos	C37 H73 O8 N1 P1	1.8490	0.8411	0.0000	0.0016
pos_781	PE(16:1/16:1)	pos	C37 H70 O8 N1 P1 Na1	1.9830	0.8219	0.0020	0.0072
pos_783	PE(17:1/16:1)	pos	C38 H73 O8 N1 P1	1.9795	0.8635	0.0009	0.0045
pos_787	PE(16:1/18:1)	pos	C39 H75 O8 N1 P1	1.5606	0.8640	0.0001	0.0024
pos_790	PE(16:1/18:2)	pos	C39 H72 O8 N1 P1 Na1	1.6996	0.8309	0.0000	0.0002
pos_808	PE(16:1/20:5)	pos	C41 H71 O8 N1 P1	1.6904	0.8408	0.0000	0.0002
pos_821	PE(16:1/22:6)	pos	C43 H73 O8 N1 P1	1.1916	0.9064	0.0001	0.0024
pos_842	PE(20:3/22:5)	pos	C47 H78 O8 N1 P1 Na1	1.1350	0.8744	0.0059	0.0153
pos_847	PEt(15:0/12:0)	pos	C32 H64 O8 N0 P1	1.3134	0.8711	0.0002	0.0026
pos_848	PEt(15:0/13:0)	pos	C33 H66 O8 N0 P1	1.3348	0.8559	0.0005	0.0034
pos_849	PEt(15:0/14:0)	pos	C34 H68 O8 N0 P1	1.2039	0.8982	0.0003	0.0028
pos_850	PEt(15:0/14:1)	pos	C34 H66 O8 N0 P1	1.2945	0.8721	0.0011	0.0049
pos_852	PEt(15:0/16:1)	pos	C36 H70 O8 N0 P1	1.1570	0.9028	0.0015	0.0061
pos_853	PEt(15:0/17:0)	pos	C37 H74 O8 N0 P1	1.0942	0.9119	0.0005	0.0034
pos_854	PEt(17:1/15:0)	pos	C37 H72 O8 N0 P1	1.3315	0.8754	0.0001	0.0024
pos_855	PEt(18:0/15:0)	pos	C38 H76 O8 N0 P1	1.4060	0.8622	0.0001	0.0024
pos_857	PEt(15:0/18:2)	pos	C38 H72 O8 N0 P1	1.4816	0.8281	0.0008	0.0041
pos_858	PEt(15:0/19:0)	pos	C39 H78 O8 N0 P1	1.1263	0.9049	0.0002	0.0024
pos_859	PEt(19:1/15:0)	pos	C39 H76 O8 N0 P1	1.2105	0.8867	0.0005	0.0035
pos_860	PEt(20:0/15:0)	pos	C40 H80 O8 N0 P1	1.0823	0.9127	0.0015	0.0062
pos_862	PEt(15:0/20:1)	pos	C40 H78 O8 N0 P1	1.0516	0.9212	0.0003	0.0028
pos_863	PEt(15:0/20:2)	pos	C40 H76 O8 N0 P1	1.0339	0.9237	0.0001	0.0022

pos_864	PEt(15:0/21:0)	pos	C41 H82 O8 N0 P1	1.2539	0.8715	0.0002	0.0024
pos_865	PEt(15:0/22:0)	pos	C42 H84 O8 N0 P1	1.0547	0.9085	0.0005	0.0035
pos_866	PEt(15:0/22:1)	pos	C42 H82 O8 N0 P1	1.0958	0.9077	0.0009	0.0046
pos_868	PEt(15:0/22:5)	pos	C42 H74 O8 N0 P1	1.2438	0.8891	0.0006	0.0035
pos_869	PEt(15:0/22:6)	pos	C42 H72 O8 N0 P1	1.3328	0.8766	0.0004	0.0031
pos_870	PEt(15:0/24:1)	pos	C44 H86 O8 N0 P1	1.1291	0.9040	0.0002	0.0024
pos_873	PG(16:1/16:1)	pos	C38 H71 O10 N0 P1 Na1	1.8446	0.8312	0.0007	0.0041
pos_882	PI(16:1/22:6)	pos	C47 H81 O13 N1 P1	1.8725	0.8162	0.0004	0.0031
pos_886	PI(22:6/22:6)	pos	C53 H83 O13 N1 P1	1.5324	0.8170	0.0022	0.0078
pos_887	PMe(16:0/12:0)	pos	C32 H64 O8 N0 P1	1.3134	0.8710	0.0002	0.0026
pos_889	PMe(16:0/14:0)	pos	C34 H68 O8 N0 P1	1.2039	0.8982	0.0003	0.0028
pos_890	PMe(16:0/14:1)	pos	C34 H66 O8 N0 P1	1.2959	0.8717	0.0010	0.0048
pos_892	PMe(16:0/16:1)	pos	C36 H70 O8 N0 P1	1.2011	0.8978	0.0010	0.0048
pos_893	PMe(16:0/17:0)	pos	C37 H74 O8 N0 P1	1.0839	0.9104	0.0002	0.0024
pos_894	PMe(17:1/16:0)	pos	C37 H72 O8 N0 P1	1.3325	0.8752	0.0001	0.0024
pos_895	PMe(18:0/16:0)	pos	C38 H76 O8 N0 P1	1.3564	0.8688	0.0002	0.0024
pos_897	PMe(16:0/18:2)	pos	C38 H72 O8 N0 P1	1.4157	0.8455	0.0018	0.0069
pos_899	PMe(19:1/16:0)	pos	C39 H76 O8 N0 P1	1.2243	0.8706	0.0043	0.0123
pos_900	PMe(20:0/16:0)	pos	C40 H80 O8 N0 P1	1.2178	0.8902	0.0001	0.0024
pos_901	PMe(18:0/18:1)	pos	C40 H78 O8 N0 P1	1.4223	0.8330	0.0022	0.0077
pos_902	PMe(16:0/20:1)	pos	C40 H78 O8 N0 P1	1.0516	0.9213	0.0003	0.0028
pos_903	PMe(16:0/20:2)	pos	C40 H76 O8 N0 P1	1.0339	0.9236	0.0001	0.0022
pos_904	PMe(16:0/20:5)	pos	C40 H70 O8 N0 P1	1.2860	0.8873	0.0002	0.0026

pos_905	PMe(16:0/21:0)	pos	C41 H82 O8 N0 P1	1.2641	0.8705	0.0001	0.0024
pos_906	PMe(16:0/22:0)	pos	C42 H84 O8 N0 P1	1.0547	0.9085	0.0005	0.0035
pos_907	PMe(16:0/22:1)	pos	C42 H82 O8 N0 P1	1.1824	0.8964	0.0004	0.0030
pos_909	PMe(16:0/22:6)	pos	C42 H72 O8 N0 P1	1.3328	0.8766	0.0004	0.0031
pos_910	PMe(16:0/24:1)	pos	C44 H86 O8 N0 P1	1.1291	0.9041	0.0002	0.0024
pos_913	PS(16:1/22:6)	pos	C44 H73 O10 N1 P1	1.7470	0.8474	0.0068	0.0171
pos_1033	TG(9:0/10:0/10:0)	pos	C32 H60 O6 K1	1.5364	0.7858	0.0004	0.0029
pos_1035	TG(6:0/13:0/13:0)	pos	C35 H66 O6 K1	1.3008	0.8624	0.0006	0.0037
pos_1036	TG(8:0/10:2/14:0)	pos	C35 H63 O6	1.2078	0.8673	0.0012	0.0054
pos_1037	TG(8:0/11:2/14:0)	pos	C36 H65 O6	1.4056	0.8394	0.0002	0.0025
pos_1038	TG(4:0/15:0/15:0)	pos	C37 H70 O6 K1	1.1664	0.8975	0.0020	0.0072
pos_1040	TG(16:1/6:0/12:2)	pos	C37 H65 O6	1.1247	0.8846	0.0022	0.0078
pos_1044	TG(16:0/8:0/11:2)	pos	C38 H69 O6	1.0556	0.9163	0.0001	0.0024
pos_1046	TG(16:1/8:0/11:2)	pos	C38 H67 O6	1.2109	0.8845	0.0001	0.0024
pos_1047	TG(14:0/10:3/11:2)	pos	C38 H63 O6	1.2043	0.8978	0.0000	0.0010
pos_1049	TG(16:0/10:0/10:2)	pos	C39 H71 O6	1.1265	0.8924	0.0136	0.0286
pos_1051	TG(12:1e/6:0/18:1)	pos	C39 H73 O5	1.0839	0.9083	0.0007	0.0039
pos_1052	TG(16:1/10:0/10:2)	pos	C39 H69 O6	1.3103	0.8495	0.0034	0.0105
pos_1053	TG(18:3e/8:0/10:0)	pos	C39 H71 O5	1.3405	0.8523	0.0007	0.0039
pos_1059	TG(16:0/10:0/11:2)	pos	C40 H73 O6	1.0695	0.8808	0.0191	0.0360
pos_1061	TG(14:1e/6:0/17:1)	pos	C40 H74 O5 Na1	1.0659	0.9164	0.0001	0.0024
pos_1062	TG(8:0/11:2/18:1)	pos	C40 H71 O6	1.2033	0.8929	0.0001	0.0023
pos_1064	TG(14:1e/11:3/12:3)	pos	C40 H65 O5	2.8438	0.3119	0.0001	0.0023

pos_1066	TG(16:0/11:1/11:1)	pos	C41 H75 O6	1.0825	0.9064	0.0035	0.0106
pos_1068	TG(12:1e/8:0/18:1)	pos	C41 H77 O5	1.1489	0.8878	0.0005	0.0032
pos_1070	TG(12:1e/6:0/20:2)	pos	C41 H75 O5	1.2619	0.8466	0.0033	0.0101
pos_1072	TG(12:1e/10:4/16:0)	pos	C41 H74 O5 N1	1.2038	0.9035	0.0005	0.0033
pos_1073	TG(12:0e/6:0/20:5)	pos	C41 H71 O5	1.1967	0.8982	0.0000	0.0019
pos_1079	TG(20:4e/8:0/10:4)	pos	C41 H65 O5	1.9896	0.6500	0.0001	0.0024
pos_1080	TG(15:0/6:0/18:1)	pos	C42 H78 O6 K1	1.1362	0.8823	0.0052	0.0139
pos_1081	TG(9:0/13:0/17:1)	pos	C42 H78 O6 K1	1.0516	0.9212	0.0003	0.0028
pos_1084	TG(4:0/17:1/18:1)	pos	C42 H76 O6 K1	1.6067	0.7938	0.0001	0.0024
pos_1085	TG(20:1/8:0/11:2)	pos	C42 H75 O6	1.3211	0.8631	0.0001	0.0023
pos_1086	TG(6:0/13:0/20:4)	pos	C42 H72 O6 K1	1.1855	0.8933	0.0010	0.0049
pos_1087	TG(18:1/10:1/11:3)	pos	C42 H71 O6	0.9314	0.9047	0.0258	0.0460
pos_1089	TG(8:0/11:2/20:5)	pos	C42 H67 O6	1.2535	0.8750	0.0001	0.0023
pos_1092	TG(16:1/10:0/14:0)	pos	C43 H84 O6 N1	1.4500	0.8134	0.0056	0.0148
pos_1093	TG(18:0/11:1/11:1)	pos	C43 H79 O6	1.0415	0.9097	0.0005	0.0032
pos_1096	TG(18:1/11:1/11:1)	pos	C43 H77 O6	1.2149	0.8757	0.0056	0.0147
pos_1097	TG(12:0e/6:0/22:4)	pos	C43 H77 O5	1.1348	0.8881	0.0117	0.0256
pos_1100	TG(12:0e/6:0/22:6)	pos	C43 H73 O5	1.4501	0.8298	0.0013	0.0057
pos_1110	TG(8:0/11:2/22:5)	pos	C44 H71 O6	1.5585	0.8173	0.0000	0.0008
pos_1111	TG(8:0/11:2/22:6)	pos	C44 H69 O6	1.8954	0.7828	0.0000	0.0002
pos_1115	TG(10:0/14:0/18:1)	pos	C45 H84 O6 K1	2.1036	0.8058	0.0000	0.0005
pos_1120	TG(18:0/10:3/14:2)	pos	C45 H77 O6	0.9356	0.9161	0.0036	0.0108
pos_1121	TG(10:0/12:2/20:4)	pos	C45 H75 O6	1.2666	0.8511	0.0017	0.0068

pos_1122	TG(12:1e/8:0/22:5)	pos	C45 H77 O5	1.0118	0.9158	0.0009	0.0046
pos_1123	TG(12:1e/10:0/20:5)	pos	C45 H77 O5	1.1944	0.8665	0.0036	0.0108
pos_1124	TG(14:0e/6:0/22:6)	pos	C45 H77 O5	1.3533	0.8495	0.0007	0.0041
pos_1127	TG(12:1e/8:0/22:6)	pos	C45 H75 O5	1.3933	0.8337	0.0013	0.0055
pos_1128	TG(10:0/10:2/22:6)	pos	C45 H71 O6	1.0564	0.9090	0.0005	0.0034
pos_1131	TG(9:0/10:0/24:1)	pos	C46 H86 O6 K1	1.1291	0.9040	0.0002	0.0024
pos_1134	TG(22:6/10:1/11:4)	pos	C46 H67 O6	1.3208	0.8587	0.0001	0.0023
pos_1139	TG(15:0/10:2/18:1)	pos	C46 H82 O6 K1	1.0151	0.9062	0.0034	0.0103
pos_1144	TG(18:2/11:2/14:4)	pos	C46 H73 O6	1.0156	0.9197	0.0001	0.0023
pos_1153	TG(16:1/14:0/14:3)	pos	C47 H86 O6 N1	1.7959	0.7628	0.0001	0.0022
pos_1157	TG(16:0/10:4/18:2)	pos	C53 H94 O6 N2	1.1190	0.8639	0.0467	0.0750
pos_1158	TG(12:1e/10:4/22:1)	pos	C47 H81 O5	1.1484	0.8481	0.0190	0.0360
pos_1159	TG(22:5/11:1/11:1)	pos	C47 H77 O6	1.0768	0.8816	0.0080	0.0195
pos_1161	TG(22:6/11:1/11:1)	pos	C47 H75 O6	1.2749	0.8752	0.0004	0.0031
pos_1168	TG(16:1/12:1/17:1)	pos	C48 H90 O6 N1	1.1985	0.8728	0.0187	0.0357
pos_1171	TG(18:4/13:0/14:0)	pos	C48 H88 O6 N1	1.8313	0.7980	0.0001	0.0024
pos_1172	TG(15:0/14:3/16:1)	pos	C48 H88 O6 N1	1.0015	0.8878	0.0074	0.0183
pos_1193	TG(16:1/8:0/22:6)	pos	C49 H84 O6 N1	1.3290	0.8392	0.0115	0.0253
pos_1202	TG(16:1/14:1/17:1)	pos	C50 H94 O6 N1	1.6506	0.8447	0.0013	0.0056
pos_1204	TG(16:1/13:0/18:3)	pos	C50 H92 O6 N1	1.2609	0.8566	0.0004	0.0029
pos_1207	TG(14:0/13:0/20:5)	pos	C50 H90 O6 N1	1.1660	0.8948	0.0011	0.0051
pos_1211	TG(11:0/14:0/22:6)	pos	C50 H88 O6 N1	1.3426	0.8489	0.0327	0.0562
pos_1231	TG(18:4/14:0/16:1)	pos	C51 H88 O6 Na1	1.0453	0.8825	0.0138	0.0288

pos_1235	TG(16:1/12:0/20:5)	pos	C51 H86 O6 Na1	1.0346	0.9055	0.0123	0.0266
pos_1236	TG(12:0/14:0/22:6)	pos	C51 H86 O6 Na1	1.1803	0.8830	0.0014	0.0061
pos_1239	TG(16:1/10:0/22:6)	pos	C51 H88 O6 N1	1.6197	0.7803	0.0047	0.0130
pos_1240	TG(16:0/10:1/22:6)	pos	C51 H88 O6 N1	1.1329	0.8621	0.0149	0.0302
pos_1242	TG(14:0/14:3/20:5)	pos	C51 H86 O6 N1	1.2691	0.8167	0.0151	0.0305
pos_1243	TG(16:0/10:2/22:6)	pos	C51 H86 O6 N1	1.2912	0.8179	0.0052	0.0140
pos_1264	TG(16:1/13:0/20:5)	pos	C52 H92 O6 N1	1.0981	0.8567	0.0306	0.0531
pos_1268	TG(11:0/16:1/22:6)	pos	C52 H90 O6 N1	1.3476	0.8459	0.0205	0.0382
pos_1298	TG(16:1/14:1/20:5)	pos	C53 H88 O6 Na1	1.2509	0.8650	0.0112	0.0248
pos_1302	TG(12:0/18:3/20:5)	pos	C53 H90 O6 N1	1.3240	0.8721	0.0021	0.0077
pos_1303	TG(16:0/14:3/20:5)	pos	C53 H90 O6 N1	1.4576	0.8086	0.0041	0.0120
pos_1306	TG(16:0/12:3/22:6)	pos	C59 H100 O6 N2	1.3820	0.7899	0.0058	0.0151
pos_1320	TG(15:0/14:0/22:5)	pos	C54 H95 O6	1.3504	0.8968	0.0165	0.0327
pos_1336	TG(18:4/14:1/20:5)	pos	C55 H86 O6 Na1	1.2792	0.8765	0.0127	0.0271
pos_1337	TG(16:0/14:4/22:6)	pos	C55 H90 O6 N1	1.3457	0.8313	0.0077	0.0189
pos_1362	TG(16:1/14:1/22:6)	pos	C55 H90 O6 Na1	0.9889	0.9062	0.0236	0.0429
pos_1365	TG(18:4/16:1/18:4)	pos	C55 H92 O6 N1	1.0466	0.8920	0.0257	0.0460
pos_1371	TG(20:5/13:0/20:5)	pos	C56 H88 O6 Na1	1.4274	0.8419	0.0244	0.0442
pos_1374	TG(20:4/11:2/22:6)	pos	C62 H100 O6 N2	1.0465	0.9235	0.0049	0.0134
pos_1375	TG(20:5/11:3/22:5)	pos	C62 H98 O6 N2	1.2542	0.8835	0.0020	0.0074
pos_1388	TG(14:0/17:1/22:5)	pos	C56 H100 O6 N1	0.9603	0.9121	0.0185	0.0355
pos_1395	TG(22:6/14:1/17:1)	pos	C56 H96 O6 N1	1.2281	0.8745	0.0118	0.0258
pos_1396	TG(18:4/15:0/20:5)	pos	C56 H90 O6 Na1	0.9974	0.8794	0.0256	0.0458

pos_1405	TG(12:0/20:5/22:6)	pos	C57 H88 O6 Na1	1.3042	0.8718	0.0008	0.0042
pos_1409	TG(20:5/12:2/22:6)	pos	C57 H85 O6	1.2792	0.8765	0.0127	0.0271
pos_1417	TG(16:0/16:0/22:4)	pos	C57 H102 O6 K1	1.7216	0.7373	0.0043	0.0123
pos_1439	TG(16:2e/16:1/22:6)	pos	C57 H98 O5 N1	1.3937	0.8318	0.0027	0.0089
pos_1450	TG(20:5/13:0/22:6)	pos	C58 H90 O6 Li1	1.6662	0.8341	0.0000	0.0006
pos_1454	TG(19:1/18:0/18:1)	pos	C58 H112 O6 N1	1.0818	0.8404	0.0220	0.0406
pos_1483	TG(18:3e/16:1/22:6)	pos	C59 H100 O5 N1	1.2417	0.8508	0.0101	0.0229
pos_1487	TG(20:5/14:1/22:6)	pos	C59 H90 O6 Na1	1.3537	0.8566	0.0034	0.0103
pos_1491	TG(20:5/14:3/22:6)	pos	C59 H90 O6 N1	1.5047	0.8013	0.0023	0.0081
pos_1492	TG(22:5/12:3/22:6)	pos	C59 H87 O6	1.3042	0.8718	0.0008	0.0042
pos_1505	TG(16:1/18:1/22:3)	pos	C59 H104 O6 K1	1.6413	0.7685	0.0042	0.0121
pos_1522	TG(20:5/18:2/18:2)	pos	C59 H96 O6 Na1	1.0446	0.8843	0.0203	0.0378
pos_1539	TG(16:0/19:0/22:4)	pos	C60 H112 O6 N1	1.1348	0.9121	0.0159	0.0319
pos_1561	TG(16:1/20:5/22:5)	pos	C61 H100 O6 N1	1.1561	0.9055	0.0196	0.0369
pos_1567	TG(22:6/14:2/22:6)	pos	C61 H94 O6 N1	1.2836	0.8704	0.0047	0.0130
pos_1568	TG(22:6/14:3/22:6)	pos	C61 H92 O6 N1	1.6154	0.7848	0.0027	0.0089
pos_1637	TG(18:3/20:4/22:6)	pos	C63 H96 O6 Na1	1.1916	0.8620	0.0138	0.0288
pos_1640	TG(18:3/20:5/22:6)	pos	C63 H95 O6	1.3103	0.8457	0.0081	0.0195
pos_1641	TG(16:2e/22:6/22:6)	pos	C63 H100 O5 N1	1.3550	0.8424	0.0040	0.0118
pos_1642	TG(18:4/20:5/22:6)	pos	C63 H92 O6 Na1	1.1916	0.8902	0.0022	0.0077
pos_1643	TG(20:5/20:5/20:5)	pos	C63 H92 O6 Li1	1.3938	0.8300	0.0009	0.0044
pos_1646	TG(26:1/16:0/18:1)	pos	C63 H122 O6 N1	1.3025	0.8861	0.0077	0.0189
pos_1667	TG(19:0/20:4/22:6)	pos	C64 H108 O6 N1	1.1106	0.8947	0.0111	0.0248

pos_1700	TG(18:3/22:5/22:6)	pos	C65 H102 O6 N1	1.1089	0.9062	0.0093	0.0214
pos_1702	TG(20:5/20:4/22:6)	pos	C65 H97 O6	1.2149	0.8685	0.0064	0.0162
pos_1704	TG(18:3e/22:6/22:6)	pos	C65 H102 O5 N1	1.1594	0.8673	0.0102	0.0232
pos_1705	TG(20:5/20:5/22:6)	pos	C65 H95 O6	1.1799	0.8599	0.0102	0.0232
pos_1714	TG(16:0/22:5/24:1)	pos	C65 H115 O6	1.0892	0.9053	0.0013	0.0057
pos_1716	TG(18:1/22:1/22:5)	pos	C65 H113 O6	1.1391	0.8878	0.0004	0.0029
pos_1720	TG(20:0/20:3/22:6)	pos	C65 H112 O6 N1	1.0632	0.9000	0.0076	0.0188
pos_1738	TG(18:1/22:5/23:0)	pos	C66 H120 O6 N1	1.0902	0.8896	0.0414	0.0679
pos_1742	TG(18:1/22:6/23:0)	pos	C66 H118 O6 N1	1.1520	0.8850	0.0173	0.0338
pos_1773	TG(18:1/22:5/24:1)	pos	C67 H117 O6	1.1262	0.8892	0.0018	0.0069
pos_1800	TG(25:0/18:1/22:5)	pos	C68 H124 O6 N1	1.0366	0.9029	0.0139	0.0288
pos_1802	TG(25:0/18:1/22:6)	pos	C68 H122 O6 N1	0.9597	0.9201	0.0090	0.0211
pos_1827	TG(20:1/22:4/24:0)	pos	C69 H128 O6 N1	1.0801	0.8847	0.0191	0.0360
pos_1830	TG(26:1/18:0/22:5)	pos	C69 H126 O6 N1	1.1615	0.8721	0.0415	0.0679
pos_1841	TG(24:0/20:4/22:5)	pos	C69 H120 O6 N1	1.0311	0.9158	0.0019	0.0071
pos_1859	TG(22:1/22:6/23:0)	pos	C70 H126 O6 N1	1.0039	0.9156	0.0054	0.0143
pos_1881	TG(22:0/22:6/24:1)	pos	C71 H124 O6 Na1	1.0410	0.8980	0.0134	0.0282
pos_1883	TG(24:1/22:4/22:4)	pos	C71 H124 O6 N1	0.9670	0.8908	0.0133	0.0281
pos_1892	TG(24:1/22:6/23:0)	pos	C72 H130 O6 N1	1.0314	0.9032	0.0058	0.0151
pos_1894	TG(26:1/22:4/22:5)	pos	C73 H126 O6 N1	1.1526	0.8845	0.0025	0.0086
pos_1897	TG(26:0/22:6/22:6)	pos	C73 H122 O6 N1	1.1438	0.8686	0.0048	0.0131
pos_1906	TG(24:0/22:6/24:1)	pos	C73 H128 O6 Na1	1.0664	0.8862	0.0091	0.0212
pos_1910	TG(24:1/22:6/24:2)	pos	C73 H128 O6 N1	1.2468	0.8671	0.0069	0.0173

pos_1913	TG(25:0/22:6/24:1)	pos	C74 H134 O6 N1	1.5454	0.8699	0.0009	0.0044
pos_1914	TG(25:1/22:6/24:1)	pos	C74 H132 O6 N1	1.1071	0.8764	0.0138	0.0288
pos_1925	TG(30:0/22:6/22:6)	pos	C77 H130 O6 N1	1.1752	0.8513	0.0119	0.0259
neg_1	CL(14:0/16:0/16:0/18:0)	neg	C73 H140 O17 P2	1.7695	0.7986	0.0004	0.0037
neg_2	CL(16:0/16:0/16:0/16:0)	neg	C73 H140 O17 P2	2.2016	0.7057	0.0001	0.0025
neg_3	CL(14:0/16:0/16:1/18:1)	neg	C73 H136 O17 P2	1.8430	0.8012	0.0004	0.0041
neg_4	CL(16:0/16:0/18:0/18:0)	neg	C77 H148 O17 P2	2.0668	0.7331	0.0002	0.0033
neg_5	CL(16:0/16:0/16:0/20:0)	neg	C77 H148 O17 P2	1.4019	0.8875	0.0002	0.0030
neg_6	CL(18:1/16:0/16:0/18:1)	neg	C77 H144 O17 P2	1.6189	0.8569	0.0002	0.0029
neg_7	CL(18:1/16:1/16:1/18:1)	neg	C77 H140 O17 P2	2.0655	0.7370	0.0002	0.0029
neg_10	CL(20:5/16:0/16:1/20:4)	neg	C81 H136 O17 P2	1.8151	0.8290	0.0001	0.0029
neg_12	CL(14:0/18:0/20:4/20:4)	neg	C81 H140 O17 P2	1.9768	0.7660	0.0003	0.0033
neg_17	CL(21:0/16:0/16:0/22:6)	neg	C84 H150 O17 P2	1.1340	0.9388	0.0018	0.0105
neg_18	CL(19:0/16:1/18:1/22:6)	neg	C84 H146 O17 P2	1.0317	0.9320	0.0097	0.0308
neg_23	CL(23:0/16:0/16:0/22:6)	neg	C86 H154 O17 P2	1.1357	0.9293	0.0029	0.0145
neg_24	CL(21:0/16:1/18:1/22:6)	neg	C86 H150 O17 P2	1.3882	0.8848	0.0004	0.0038
neg_29	CL(22:6/15:0/20:0/22:0)	neg	C88 H158 O17 P2	0.9437	0.9436	0.0006	0.0056
neg_38	CL(17:0/22:5/22:6/22:6)	neg	C92 H145 O17 P2	1.6512	0.8203	0.0017	0.0102
neg_71	FA(18:4)	neg	O2 H27 C18	1.1670	0.9225	0.0009	0.0070
neg_75	FA(22:5)	neg	O2 H33 C22	1.2643	0.9245	0.0001	0.0023
neg_78	GD2(d19:0/22:6)	neg	C83 H136 O34 N4	1.2483	0.8847	0.0043	0.0180
neg_92	Hex2Cer(d12:0/16:0)	neg	C40 H76 O13 N1	1.9586	0.7766	0.0001	0.0019
neg_95	Hex2Cer(d24:0/22:6)	neg	C60 H104 O15 N1	1.2601	0.8777	0.0468	0.0966

neg_96	Hex3Cer(m21:1/22:6)	neg	C61 H102 O17 N1	1.4179	0.8615	0.0413	0.0891
neg_97	LPE(15:0)	neg	C20 H41 O7 N1 P1	1.6679	0.7856	0.0048	0.0193
neg_100	LPE(18:2)	neg	C23 H43 O7 N1 P1	1.3886	0.8667	0.0041	0.0176
neg_105	LPE(20:5)	neg	C25 H41 O7 N1 P1	1.3013	0.9005	0.0002	0.0029
neg_109	LPEt(22:6)	neg	C27 H42 O7 N0 P1	1.0906	0.8843	0.0433	0.0918
neg_110	LPG(15:0)	neg	C21 H42 O9 N0 P1	1.3352	0.8560	0.0077	0.0271
neg_112	LPG(16:1)	neg	C22 H42 O9 N0 P1	2.1730	0.7264	0.0000	0.0010
neg_113	LPG(20:4)	neg	C26 H44 O9 N0 P1	1.0544	0.9113	0.0046	0.0190
neg_114	LPG(20:5)	neg	C26 H42 O9 N0 P1	1.4210	0.8518	0.0009	0.0070
neg_116	LPI(16:0)	neg	C25 H48 O12 N0 P1	1.3211	0.8590	0.0376	0.0840
neg_118	LPI(18:1)	neg	C27 H50 O12 N0 P1	1.4871	0.8245	0.0118	0.0355
neg_119	LPI(20:1)	neg	C29 H54 O12 N0 P1	2.7025	0.2044	0.0117	0.0353
neg_123	LPI(20:5)	neg	C29 H46 O12 N0 P1	1.4700	0.8365	0.0021	0.0114
neg_124	LPI(22:5)	neg	C31 H50 O12 N0 P1	1.4958	0.8260	0.0134	0.0388
neg_125	LPI(22:6)	neg	C31 H48 O12 N0 P1	1.4501	0.8613	0.0066	0.0247
neg_209	OAHFA(18:1/18:0)	neg	C36 H67 O4	1.2036	0.9104	0.0002	0.0032
neg_210	OAHFA(18:1/20:3)	neg	C38 H65 O4	1.1324	0.9154	0.0005	0.0046
neg_213	OAHFA(22:5/22:4)	neg	C44 H67 O4	1.5821	0.8313	0.0020	0.0113
neg_261	PC(28:1/22:6)	neg	C57 H99 O8 N1 P1	1.4712	0.8554	0.0067	0.0247
neg_262	PC(29:1/22:6)	neg	C58 H101 O8 N1 P1	1.2851	0.8704	0.0374	0.0839
neg_267	PC(30:1/22:6)	neg	C59 H103 O8 N1 P1	1.0713	0.9176	0.0315	0.0720
neg_278	PE(16:1/14:0)	neg	C35 H67 O8 N1 P1	1.9284	0.7992	0.0000	0.0006
neg_290	PE(16:0/18:2)	neg	C39 H73 O8 N1 P1	1.8096	0.8449	0.0000	0.0006

neg_293	PE(14:0/20:4)	neg	C39 H69 O8 N1 P1	1.3040	0.9022	0.0000	0.0005
neg_297	PE(17:1/18:1)	neg	C40 H75 O8 N1 P1	1.1578	0.9363	0.0010	0.0074
neg_302	PE(18:1/18:1)	neg	C41 H77 O8 N1 P1	1.4995	0.8964	0.0000	0.0003
neg_304	PE(18:1/18:2)	neg	C41 H75 O8 N1 P1	1.2004	0.8975	0.0020	0.0112
neg_309	PE(16:1/20:4)	neg	C41 H71 O8 N1 P1	1.6753	0.8528	0.0000	0.0001
neg_310	PE(14:0/22:6)	neg	C41 H69 O8 N1 P1	1.4647	0.8808	0.0000	0.0002
neg_321	PE(15:0/22:6)	neg	C42 H71 O8 N1 P1	1.0868	0.9208	0.0019	0.0112
neg_339	PE(16:2e/22:6)	neg	C43 H71 O7 N1 P1	1.1425	0.9196	0.0031	0.0148
neg_344	PE(17:1/22:6)	neg	C44 H73 O8 N1 P1	1.4490	0.8688	0.0012	0.0080
neg_345	PE(18:4/22:6)	neg	C45 H69 O8 N1 P1	1.4835	0.8520	0.0003	0.0033
neg_352	PE(18:1/22:5)	neg	C45 H77 O8 N1 P1	1.1372	0.9023	0.0092	0.0299
neg_356	PE(20:4/20:4)	neg	C45 H73 O8 N1 P1	1.1364	0.9218	0.0003	0.0037
neg_368	PE(22:5/20:4)	neg	C47 H75 O8 N1 P1	1.2177	0.9045	0.0064	0.0245
neg_371	PEt(12:0e/22:6)	neg	C39 H66 O7 N0 P1	1.1811	0.9239	0.0002	0.0033
neg_372	PEt(18:0/18:1)	neg	C41 H78 O8 N0 P1	1.1679	0.8687	0.0143	0.0410
neg_374	PEt(16:0e/20:4)	neg	C41 H74 O7 N0 P1	2.1788	0.4567	0.0219	0.0564
neg_378	PEt(16:1/22:6)	neg	C43 H70 O8 N0 P1	3.1446	0.1904	0.0003	0.0033
neg_380	PEt(18:1/22:6)	neg	C45 H74 O8 N0 P1	1.0345	0.8992	0.0209	0.0555
neg_383	PEt(22:6/22:6)	neg	C49 H72 O8 N0 P1	2.9725	0.3280	0.0037	0.0169
neg_388	PG(15:0/16:1)	neg	C37 H70 O10 N0 P1	2.0170	0.5804	0.0286	0.0680
neg_389	PG(16:0/16:1)	neg	C38 H72 O10 N0 P1	1.9125	0.7643	0.0001	0.0023
neg_390	PG(12:1e/20:4)	neg	C38 H66 O9 N0 P1	0.9633	0.9385	0.0004	0.0041
neg_392	PG(17:1/16:1)	neg	C39 H72 O10 N0 P1	2.0619	0.6620	0.0111	0.0337

neg_397	PG(18:1/18:1)	neg	C42 H78 O10 N0 P1	1.5098	0.8497	0.0003	0.0033
neg_400	PG(16:0/20:5)	neg	C42 H72 O10 N0 P1	1.8116	0.6379	0.0377	0.0840
neg_401	PG(16:1/20:4)	neg	C42 H72 O10 N0 P1	1.5270	0.8563	0.0002	0.0033
neg_402	PG(16:1/20:5)	neg	C42 H70 O10 N0 P1	1.6851	0.8103	0.0004	0.0039
neg_404	PG(16:2e/20:4)	neg	C42 H72 O9 N0 P1	1.9128	0.7647	0.0001	0.0027
neg_408	PG(16:1/22:5)	neg	C44 H74 O10 N0 P1	1.8614	0.7666	0.0000	0.0006
neg_409	PG(18:1/20:5)	neg	C44 H74 O10 N0 P1	1.4494	0.8649	0.0011	0.0075
neg_412	PG(16:1/22:6)	neg	C44 H72 O10 N0 P1	0.9102	0.8997	0.0393	0.0856
neg_426	PI(14:0/20:4)	neg	C43 H74 O13 N0 P1	1.8132	0.7633	0.0003	0.0034
neg_430	PI(16:1/20:4)	neg	C45 H76 O13 N0 P1	1.5423	0.8403	0.0009	0.0070
neg_433	PI(16:1/20:5)	neg	C45 H74 O13 N0 P1	1.3473	0.8320	0.0167	0.0455
neg_434	PI(14:0/22:6)	neg	C45 H74 O13 N0 P1	2.1772	0.7237	0.0000	0.0002
neg_436	PI(17:1/20:4)	neg	C46 H78 O13 N0 P1	1.1412	0.8589	0.0365	0.0824
neg_437	PI(15:0/22:6)	neg	C46 H76 O13 N0 P1	1.4668	0.7986	0.0081	0.0278
neg_440	PI(16:1/22:5)	neg	C47 H78 O13 N0 P1	1.3279	0.8525	0.0183	0.0493
neg_445	PI(17:1/22:6)	neg	C48 H78 O13 N0 P1	1.5656	0.8229	0.0015	0.0094
neg_450	PI(18:3/22:6)	neg	C49 H76 O13 N0 P1	1.4759	0.8135	0.0021	0.0114
neg_452	PI(20:5/22:6)	neg	C51 H76 O13 N0 P1	3.0065	0.2898	0.0023	0.0123
neg_453	PI(22:5/20:4)	neg	C51 H80 O13 N0 P1	1.2006	0.8527	0.0192	0.0514
neg_454	PI(22:4/22:6)	neg	C53 H82 O13 N0 P1	3.3077	0.0253	0.0006	0.0051
neg_455	PI(22:5/22:6)	neg	C53 H80 O13 N0 P1	1.8827	0.7586	0.0002	0.0032
neg_458	PMe(16:1/16:1)	neg	C36 H66 O8 N0 P1	2.2066	0.7134	0.0000	0.0001
neg_459	PMe(16:1/18:1)	neg	C38 H70 O8 N0 P1	2.4725	0.6715	0.0000	0.0000

neg_461	PS(16:1/18:2)	neg	C40 H71 O10 N1 P1	2.0266	0.7738	0.0000	0.0006
neg_462	PS(16:1/18:3)	neg	C40 H69 O10 N1 P1	2.4417	0.6908	0.0000	0.0000
neg_465	PS(18:1/18:2)	neg	C42 H75 O10 N1 P1	1.3684	0.8947	0.0001	0.0017
neg_467	PS(14:0/22:6)	neg	C42 H69 O10 N1 P1	1.5410	0.8380	0.0001	0.0027
neg_484	PS(18:3/20:5)	neg	C44 H69 O10 N1 P1	3.5823	0.1426	0.0000	0.0009
neg_498	PS(18:2/22:6)	neg	C46 H73 O10 N1 P1	1.2274	0.8652	0.0107	0.0331
neg_501	PS(18:3/22:6)	neg	C46 H71 O10 N1 P1	1.7189	0.8052	0.0010	0.0073
neg_502	PS(18:3e/22:6)	neg	C46 H73 O9 N1 P1	1.0566	0.9125	0.0040	0.0172
neg_507	PS(20:4/22:6)	neg	C48 H73 O10 N1 P1	1.0995	0.9174	0.0014	0.0089
neg_517	PS(20:2/22:6)	neg	C48 H77 O10 N1 P1	0.9974	0.9396	0.0012	0.0080
neg_520	PS(20:5/22:4)	neg	C48 H75 O10 N1 P1	1.4092	0.8605	0.0107	0.0331
neg_546	dMePE(16:1/14:1)	neg	C37 H69 O8 N1 P1	2.1353	0.7653	0.0000	0.0008
neg_592	dMePE(20:5/20:5)	neg	C47 H73 O8 N1 P1	1.0653	0.8818	0.0299	0.0697
neg_601	dMePE(20:5/22:6)	neg	C49 H75 O8 N1 P1	1.0824	0.9208	0.0149	0.0423
neg_603	dMePE(22:6/22:6)	neg	C51 H77 O8 N1 P1	1.6366	0.8320	0.0001	0.0025
